# Author Correction: The copy number and mutational landscape of recurrent ovarian high-grade serous carcinoma

**DOI:** 10.1038/s41467-023-41611-0

**Published:** 2023-09-26

**Authors:** Philip Smith, Thomas Bradley, Lena Morrill Gavarró, Teodora Goranova, Darren P. Ennis, Hasan B. Mirza, Dilrini De Silva, Anna M. Piskorz, Carolin M. Sauer, Sarwah Al-Khalidi, Ionut-Gabriel Funingana, Marika A. V. Reinius, Gaia Giannone, Liz-Anne Lewsley, Jamie Stobo, John McQueen, Gareth Bryson, Matthew Eldridge, The BriTROC Investigators, R. M. Glasspool, C. Gourley, R. Kennedy, G. Hall, R. Edmondson, A. Clamp, S. Sundar, A. Walter, M. Hall, H. Gabra, C. Fotopoulou, E. Brockbank, A. Montes, M. Lockley, Geoff Macintyre, Florian Markowetz, James D. Brenton, Iain A. McNeish

**Affiliations:** 1 CRUK Cambridge Institute, University of Cambridge, Cambridge, UK; 2 Ovarian Cancer Action Research Centre, Department of Surgery and Cancer, Imperial College London, London, UK; 3 Cambridge University Hospitals NHS Foundation Trust, Cambridge, UK; 4 CRUK Glasgow Clinical Trials Unit, Institute of Cancer Sciences, University of Glasgow, Glasgow, UK; 5 Department of Histopathology, Queen Elizabeth University Hospital, Glasgow, UK; 6 Centro Nacional de Investigaciones Oncológicas, Madrid, Spain; 7 Beatson West of Scotland Cancer Centre, Glasgow, UK; 8 University of Edinburgh, Edinburgh, UK; 9 Queen’s University, Belfast, UK; 10 University of Leeds, Leeds, UK; 11 University of Manchester, Manchester, UK; 12 Christie Hospital Foundation NHS Trust, Manchester, UK; 13 University of Birmingham, Birmingham, UK; 14 Bristol Oncology Centre, Bristol, UK; 15 East and North Hertfordshire NHS Trust, Stevenage, UK; 16 Barts and the London NHS Trust, London, UK; 17 Guy’s and St Thomas’ NHS Trust, London, UK; 18 Barts Cancer Institute, Queen Mary University of London, London, UK

**Keywords:** Ovarian cancer, Ovarian cancer, Cancer genomics

Correction to: *Nature Communications* 10.1038/s41467-023-39867-7, published online 20 July 2023

The original version of this Article mistakenly misspelled “Carolin M. Sauer” from the author list as “Carolyn Sauer,” who is from the ‘CRUK Cambridge Institute, University of Cambridge, Cambridge, UK.’ This has been corrected in both the PDF and HTML versions of the Article.

The original version of this Article mistakenly misspelled “Ionut-Gabriel Funingana” from the author list as “Ionat-Gabriel Funingana,” who is from the ‘CRUK Cambridge Institute, University of Cambridge, Cambridge, UK’ and ‘Cambridge University Hospitals NHS Foundation Trust, Cambridge, UK.’ This has been corrected in both the PDF and HTML versions of the Article.

The original version of this Article contained an error in Fig. 1a, in which a panel indicating copy number event analysis was missing from the study workflow diagram. This has been corrected in both the PDF and HTML versions of the Article.

The original version of this Article contained an error in the Methods section which incorrectly read ‘Fastq sequencing reads were aligned to GRCh37 (g1kp2) using bwa samse.’ The correct version states ‘hs37d5’ in place of ‘glkp2.’ This has been corrected in both the PDF and HTML versions of the Article.

The original version of this Article contained an error in the Data Availability Statement stating ‘The pre-processed single nucleotide variant and copy number data are available through a Zenodo data repository and can be downloaded here’ which linked to an incorrect version of the Zenodo repository. The correct link is as follows: https://zenodo.org/record/7573784. This has been corrected in both the PDF and HTML versions of the Article.

