# Supplementary material for: The copy number and mutational landscape of recurrent ovarian high-grade serous carcinoma
Source: Nat Commun. 2023 Jul 20;14:4387. doi: 10.1038/s41467-023-39867-7 (PMC10359414; doi:10.1038/s41467-023-39867-7)
Supplement: Supplementary file 1 — Supplementary Information [file 41467_2023_39867_MOESM1_ESM.pdf]

Supplementary information

Supplementary methods

Patient inclusion and exclusion criteria

Inclusion criteria

1. Patients with recurrent histologically-proven ovarian cancer, primary peritoneal carcinoma or fallopian tube cancer of high grade serous and high grade endometrioid subtypes. Patients who have a diagnosis of ovarian cancer with a known germline mutation in *BRCA1* or *BRCA2* will also be eligible for inclusion regardless of histological subtype. Patients who are having a diagnostic image-guided biopsy may be consented and study biopsy taken while awaiting pathological review. Eligible patients who have had samples collected under generic research consent may be registered retrospectively only after full discussion between the site, Chief Investigator and Cancer Research UK Clinical Trials Unit (and BriTROC-1 specific consent obtained).
2. Patients must have received at least one line of platinum-containing chemotherapy
3. Availability of formalin-fixed, paraffin-embedded tissue taken at the time of original diagnosis of high grade serous ovarian cancer. This may be primary surgical debulking specimen OR core biopsy. For those with only a core biopsy from time of diagnosis, availability of specimens taken at interval debulking surgery is desirable, but not essential.
4. Patients must have disease deemed suitable for imaging-guided biopsy (ultrasound or CT) by an experienced radiologist or suitable for intra-operative biopsy during secondary debulking surgery as determined by an experienced gynaecological oncology surgeon. Other biopsies, such as skin deposits, are also acceptable. However, this must be confirmed with the Cancer Research UK Clinical Trials Unit prior to patient registration.
5. Age ≥ 18 years.
6. Written informed consent.
7. Able to apply with study procedures.
8. Life expectancy > 3 months
9. No contraindication to biopsy as appropriate.

Exclusion criteria

1. Ovarian, primary peritoneal or fallopian tube cancer of low grade serous, grades 1 or 2 endometrioid, clear cell or carcinosarcoma/MMMT subtypes unless associated with known germline mutation in BRCA1 or BRCA2.
2. Borderline/low malignant potential tumours
3. Any non-epithelial ovarian malignancy
4. Patients with asymptomatic rising CA125 with no radiological evidence of recurrent ovarian cancer.
5. Original diagnosis of high grade serous cancer made on cytology only

Patient & sample subsetting

Across multiple analyses performed in this publication, the number of available samples and patients changes depending on the analysis and methodology being used due to various filtering criteria and sample availability. Provided is a reference table describing the sample numbers for the predominant analyses in this publication (Table S8).

Tagged Amplicon Sequencing

Read alignment

Sequenced reads were aligned to the human reference genome (GRCh37 - g1kp2 *i.e.* 'hs37d5'), using the bwa-mem algorithm (version: 0.7.17-r1188) in paired-end mode. Duplicate reads (*i.e.* paired-end reads with the same orientation position and start and end positions) were left unmarked and were not removed during the alignment process.

Read alignment post-processing and QC

Samtools (v1.10) was used to fill in mate co-ordinates and insert sizes fields using the *fixmate* utility after the reads had been sorted by name using samtools *sort*. The same utility was used to resort the aligned reads this time by position so that the bam files could be cleaned by the Picard (v2.25.7) *CleanSam* utility. Mate information in the alignments were further cleaned using the Picard *FixMateInformtion* utility. Picard's *AddOrReplaceReadGroup* was then used to annotate bam headers with information relating to library, barcode and sample identifiers as well as sequencing platform and centre information. Bam files were then indexed using samtools *index* and finally validated using Picard's *ValidateSamFile* utility to ensure that bam files were valid before any downstream processing occurred.

The following steps were implemented using code developed by CRUK Cambridge Institute's bioinformatics core (v0.7.2; https://github.com/crukci-bioinformatics/ampliconseq):

Alignments were further cleaned to retain only those aligned reads whose alignment began within 1 base pair of the start or end position of any pre-specified amplicon genomic interval. Reads in which the corresponding mate pair read did also not align to the corresponding end of the amplicon were also removed. Each alignment file was split into a minimal set of non-overlapping amplicon alignment files, such that no subsequent alignment file contained any overlapping amplicons. This precludes the opportunity of errors in which the primer regions of some amplicons overlap with the targeted region of other amplicons, and therefore creating erroneous mutant allele fractions.

Germline Variant Calling

Germline short variants were called on non-tumour whole blood samples with the aid of CRUK-CI's *ampliconseq* pipeline (https://github.com/crukci-bioinformatics/ampliconseq; v0.7.2) and the *individual* calling mode of octopus. Processing using each option is described sequentially.

HaplotypeCaller/ampliconseq

The ampliconseq pipeline wraps around core variant calling algorithms with additional QC, preprocessing and post-processing steps. Relevant pipeline steps were as followed:

All variant calling was performed using GATK's HaplotypeCaller (GATK version 3.8-0-ge9d806836)[1, 2]. Variant calling was performed on individual library read alignments. Within each library, variant calling was performed on the targeted regions of individual amplicons, and therefore excluded amplicon primer and non-amplicon genomic regions.

In instances in which the same variant was called from multiple amplicons covering the same locus, the variant record with highest variant quality score was selected, whilst the remaining variant record was discarded. For instances in which paired end reads overlapped, both reads were discarded if the base calls were discordant, otherwise the read with the highest mapping quality was selected for the computation of downstream read count and SNV metrics.

HaplotypeCaller hard filters implemented were as followed for SNVs:
QD < 2.0; FS > 60.0; MQ < 40.0; MQRankSum < -12.5

And for short indels:
QD < 2.0; FS > 200.0

In addition to HaplotypeCaller filters, further filtering was performed by two unique functions within the ampliconseq pipeline which models dataset noise: The first models substitution specific noise at a specific locus for all libraries within a single sequencing run. The second models noise within individual libraries. Thresholds are determined based on modelled beta distributions using quantiles corresponding to a probability of 0.9999. All called variants below these two library and position specific noise thresholds are discarded. Variants which were not detected in both technical replicate libraries, or did not pass all quality controls filters for both technical replicates were discarded.

Octopus

Germline variants were called with octopus using the *individual* calling mode. Variants were called in this mode using default parameter thresholds with the *–disable-downsampling*, *–allow-marked-duplicates* and *–allow-octopus-duplicates* flags all activated during variant calling. Called variants which did not pass any of the default parameter hard thresholds were discarded.

A second round of filtering using version 0.7.2 of the octopus germline random forest model was also implemented. Variants with a random forest predicted genotype quality score below the default threshold (*i.e.* 3) were discarded. Variants in which the called predicted genotypes for both technical replicates were discordant were also discarded.

Germline variant post-processing

Variants called from either the ampliconseq pipeline or octopus were functionally annotated and filtered as described below (see *Variant annotation* section). Variants identified through clinical testing (even for some patients without germline TAm-Seq sequencing) are detailed in the supplementary data 1, containing a table of short variants. By default, variants called using the octopus algorithm were accepted. Variants called using HaplotypeCaller required additional evidence in order to be accepted into the final call set. This could be either concordance with octopus variant calling or concordance with the results of clinical testing. All instances of the *BRCA2:p.T3033Lfs*29* were removed from the final call set for the following reasons: generally low mutant allele fraction, frequent failing of QC filters when all putative variants were considered together, and additionally the failure of this variant to pass QC filters when variants were called jointly between normal and tumour samples.

Tumour Sample Variant Calling
All variant calling on tumour samples were performed using the *cancer* calling mode of Octopus (v0.7.2)[3] with the exception of the *TP53* variants which were used to guide copy number calling, which were as reported previously[4, 5, 6]), although *TP53* variants were also called independently for this study:

Variant calling was performed on tumour samples in two primary modes which will be described here individually - a *TP53* and a non-*TP53* calling mode:

TP53 somatic variant calling

TP53 variants are called separately due to their unique role as necessary and ubiquitous drivers of tumourigenesis in high grade serous carcinoma[7], and the presence therefore of an identical *TP53* mutation being expected in all tumour samples from the same patient. For *TP53,* variants were called on each tumour sample individually.

Octopus was called in *cancer* calling mode with the following options: Downsampling of reads was disabled using the –*disable-downsampling* flag and both aligner and Octopus recognised read duplicates were not removed using the –*allow-marked-duplicates* and *–allow-octopus-duplicates* flags respectively. Expected somatic mutation frequencies were set at 0.03 and 0.01 using the *–min-expected-somatic-frequency* and *–min-credible-somatic-frequency* flags respectively. Octopus will attempt to classify called variants as germline or somatic, and only variants classified as somatic were retained using the *–somatics-only* command line flag. Somatic variants were further filtered using default hard filter parameter value thresholds with the exception of the *AF* parameter being set to '*AF < 0.03*' for the –*somatic-filter-expression* flag. Variants which were not detected in both technical replicates for a given sample were

discarded.

The set of amplicon regions for *TP53* variant calling were set to the union of genomic ranges specified in amplicon panels 1, 10 and 28 (as described in supplementary data 5).

Due to their often being multiple *TP53* variants detected per patient, all *TP53* mutations identified per patient were classified as suspected *driver* or *non-driver* mutations. A combined ranking and scoring process was conducted for each patient's set of identified *TP53* variants. The mutation designated as the likely driver mutation for each patient was selected as being the representative *TP53* mutation for the construction of oncoprints reported in this study (*i.e.* Figure 2, Figures S26 and S27).

More precisely and formally, the scoring process for each patients set of *TP53* variants were as followed:

$$v_i^{score} = v_i^{maf} + 3v_i^{nsample} + v_i^{qual} + v_i^{penality}$$

Where v is a unique list of *TP53* mutations identified per patient indexed from $1, ..., n$ for n unique identified *TP53* mutations per patient. v^{maf}, $v^{nsample}$ and v^{qual} are all descending rankings of v by the mutant allele fraction, number of tumour samples in which the variant was located, and the variant quality score respectively such that the set of elements of v^{maf}, $v^{nsample}$ and v^{qual} are all equal to $\{1, ..., n\}$. $v^{penalty}$ accounts for potential batch effects by penalising variants which do not appear in both the diagnosis and relapse tumour classes such that $v^{penalty} \in \{0,12\}$. The suspected *TP53* driver mutation is labelled using the variant corresponding to $min(v^{score})$.

Non-*TP53* somatic variant calling

Non-*TP53* variants were called using four different methods: *unmatched and paired, matched and paired, matched and unpaired* and *unmatched and unpaired* analysis modes. In this context, *matching* refers to analyses supported with a matched normal, non-tumour whole blood sample for the same patient. Pairing (Figure 2) refers to the use of both *diagnosis* and *relapse* tumour types for a particular analysis mode. *Matched* analyses are of increased confidence and can be used to confidently classify variants as germline or somatic (Figures S26 and S27), however matched analyses occur with restricted sample sizes due to the extra requirement of available sequenced non-tumour samples.

Tumour samples which were suspected of low cellularity (either from the copy number analysis or from pathology reported predictions of sample cellularity) were not assessed for somatic variants. Samples with failed sequencing in any amplicons of the *TP53* gene (*i.e.* coverage <100 for either technical replicate) were also not assessed for variants as these samples were not judged to have met quality control standards.

For all four non-*TP53* somatic variant calling modes, joint variant calling was performed for all available tumour samples (and non-tumour samples where applicable) for a given patient. After core variant calling and filtering using octopus, a *post-hoc* quality score of 500 was applied. All variants in which the variant did not appear in both technical replicates for a sample were discarded. All variants were passed through additional fixation artefact and variant functional annotation filters as described in below sections (fixation artefact correction and variant annotation).

Unmatched and unpaired analyses

Octopus was executed in *cancer* calling mode with *--min-expected-somatic-frequency* set to 0.03 and *--min-credible-somatic-frequency* set to 0.01. Additional flags passed were as follows: *–disable-downsampling, –allow-marked-duplicates* and *–allow-octopus-duplicates.*

The first set of variant filtering implemented both germline and somatic random forest models (v0.7.2), in which all variants not passing the random forest filter were discarded. A second round of variant filtering was applied using default threshold values with the exception of 'QUAL < 10' for *–filter expression* and 'AF < 0.01' for *–somatic-filter-expression.*

Variants which appeared at high frequencies in the study group were cross-referenced against the results of the corresponding *matched and unpaired* analysis. If the variant appeared at a much reduced frequency in the matched and unpaired analysis, then the putative recurrent mutation was regarded to be spurious. This was the case for two variants: *BRCA1:p.Lys654SerfsTer47* and *BRCA2:p.Thr3033LeufsTer29.*

A known limitation of the octopus algorithm is that when calling variants in an unmatched analysis, germline variants with inflated MAFs due to events inducing loss of heterozygosity erroneously fail some hard filter thresholds (in particular the *AF* and *AFB* filters). To counteract this limitation of the algorithm, known germline mutations from the *germline* analysis were added *post hoc* to the set of variants called in *unmatched* analyses.

Unmatched and paired analyses

The aim of this analysis is to determine which variants are shared or exclusive to the given *diagnosis* and *relapse* tumour classes. This analysis mode occurs downstream of all analysis steps described for the *unmatched and unpaired* analyses described above:

Targeted variant calling (also referred to as *specific variant calling*) was performed on all variants discovered as part of the *unmatched and unpaired* analysis, although this time with relaxed thresholds for some hard filters, more specifically: '*AF < 0.001*' and '*AFB < 0.50*' for the *–filter-expression* command-line flag. Relaxing parameter thresholds at this stage increases sensitivity for putative shared variants between the *diagnosis* and *relapse* tumour classes without substantially reducing specificity more generally for all assessed genomic loci. Variants which were discordant between diagnosis and relapse tumour samples were manually assessed using IGV in order to determine if variant detection, or lack of detection occurred due to algorithm error.

Matched and unpaired analyses

For matched analyses, non-tumour bam files were supplied via the -N flag when calling octopus. As in previous analysis modes, *--min-expected-somatic-frequency* was set to 0.03 and *--min-credible-somatic-frequency* was set to *0.01.*

The first round of variant filtering was conducted using both the germline and somatic random forest models (v0.7.2) supplied as part of octopus. The default threshold of 3 for the *RFGQ_ALL* parameter was applied. A subsequent round of filtering was applied using default hard filter thresholds except for '*QUAL < 10'* for *–filter-expression* and 'AF < 0.03' for the *–somatic-filter-expression* flag.

Matched and paired analyses

Targeted analyses were performed as described in the *unmatched and paired* analysis section. The results of this analysis are reported below:

Due to differences in methodology, the results reported in Figure 2 are not expected to be perfectly aligned/concordant with the results reported in Figures S26 and S27. The main differences in methodology that explain these differences are as follows:

i) Figures S26 and S27 are matched for available normal tissue whilst Figure 2 is not. Not all cases had available whole blood DNA libraries, which limits/decreases the number of cases examined *(i.e.* the number of columns in the oncoprint) for Figures S26 and S27. This explains why the *RAD51D* diagnosis only mutation for case 101 does not appear in Figures S26 and S27.

ii) Normal whole blood samples were assessed using two different amplicon panels (panel 6 and panel 28) whilst only amplicon panel 28 was used to sequence tumour samples, the matched analysis reported in Figures S26 and S27 is

limited to those genes in the intersection of panels 6 and 28. Thus, the diagnosis only mutation for *NF1* for case 51 does not appear in Figures S26 and S27.

iii) There was a slight difference in the mutant allele fraction threshold used for Fig 2 and Figures S26 and S27. This explains the discrepancy for *PALB2* for case 139.

Fixation artefact correction

In order to identify any potential substitution-specific artefacts, suspected artefacts in which a mutation appeared in only one of two technical duplicates were counted, and MAF density estimates were produced. As previously reported[8], a large enrichment of C>T transitions were identified in formalin fixed tissue compared to tissues preserved using different methods (*e.g.* UMFIX fixation in this study). Additionally, MAF density estimates for C>T substitutions from formalin fixed samples were shifted to the right compared to those not fixed with formalin indicating that when such artifactual transitions were detected, they were present to a greater extent in formalin fixed samples. The total number of artifactual mutations of this type detected in formalin fixed samples were also greater.

An additional MAF threshold of 0.23 was implemented for C>T substitutions (and correspondingly cognate G>A substitutions) as a result. In DNA samples with particularly poor quality DNA due to formalin fixation, artefact MAFs are inflated[9] leading to highly discordant MAFs between technical replicates. As a result, an additional C>T/G>A filter was also implemented for variants in which MAFs differed by more than 0.30 in order to remove further artefacts.

Variant annotation

All non-*TP53* variants were functionally annotated using Ensembl's *variant effect prediction* (VEP) pipeline[10] (v102.0). VEP was executed using the *–everything* and *–check_existing* flags.

Only annotations for a gene's canonical/representative transcript (as determined by VEP) were considered. Variants which were detected within genes within a short range upstream or downstream of the target gene were also discarded. Variants were further refined using the molecular tumour board portal (MTBP)[11]. More specifically, variants labelled as benign or likely benign by MBTP were discarded.

Inference of sample mislabelling events

Two different approaches were used and combined in order to identify putative sample mislabelling events. Firstly, the concordance between libraries belonging to normal and tumour samples deriving from the same patient were tested using a modified version of the HaveYouSwappedYourSamples method[12].. Namely, pairwise concordance scores were calculated for all normal-tumour library pairs by determining the proportion of high MAF variants (as determined by HaplotypeCaller) which were shared between each library pair. A threshold was then applied in order to classify library pairs as being either concordant or discordant. Normal-tumour sample pairs were classified as potentially discordant if they did not contain any expected concordant library pairs. Given the sparsity of the genomic information obtained from the amplicon sequencing data, no sample swap events could be determined with high confidence.

Secondly, a sample was identified as potentially being mislabelled if it contained what appeared to be a high MAF *TP53* mutation that was discordant for other *TP53* mutations classified as 'driver' for that patient.

Copy number fitting

Modified QDNAseq implementation

QDNAseq was modified to allow for read counts to be corrected for GC and mappability whilst being able to transform data back into read count space. To do this, several modifications were made to QDNAseq. In the correctBins() function the following line was altered. After bin correction is performed using QDNAseq correctBins() function, the

following transformation is applied to the binned copy number data to correct by the estimated bin correction generated by the estimateCorrection() function.

```
readCountsFiltered<-applyFilters(readCounts,residual=TRUE,blacklist=TRUE)
readCountsFiltered <- estimateCorrection(readCountsFiltered)
copyNumbers <- correctBins(readCountsFiltered)

assayDataElement(copyNumbers,"copynumber") <- sweep(assayDataElement(
copyNumbers,"copynumber"),2,apply(assayDataElement(readCountsFiltered,"fit"),2,Median,na.rm=T),FUN='*')

copyNumbersSmooth <- smoothOutlierBins(copyNumbers)
copyNumbersSegmented <- segmentBins(copyNumbersSmooth,transformFun="sqrt")
```

Additionally, the sqrtadhoc() function which is utilised in the segmentBins() function was modified to adjust the anscombe transformation to be utilised with non-log data, in accordance with the previous alterations to the bin correction.

```
## Original
sqrtadhoc <- function(x, factor=sqrtfactor(), offset=sqrtoffset(), inv=FALSE){
  if (!inv){
    x <- x + offset
    sqrt(x * factor)
  } else{
    x <- x^2 * (factor^-1)
    x - offset
  }
}
```

```
## Modified
sqrtadhoc <- function(x, factor=sqrtfactor(), offset=sqrtoffset(), inv=FALSE){
  if (!inv){
    x <- x + offset
    2*sqrt(x + factor)
  } else{
    x <- (x/2)^2 - (factor)
    x - offset
  }
}
```

This modified version of QDNAseq is available at https://github.com/markowetzlab/QDNAseqmod.

Profile fitting

A grid search was performed across ploidy and purity ranges to estimate absolute copy number profile fitting. A range of quantitative and qualitative metrics were used to select the correct ploidy-purity combination from a single or set of best absolute fits for a given copy number profile. Samples with multiple equally likely fits were assessed independently by two investigators to select the best fit or exclude a sample from downstream analysis. Discordant assessments were discussed until a consensus was reached.

Sample fits were also subject to fit 'power' calculations, in which fits with insufficient reads to support the selected ploidy-purity combination were excluded from selection. Sufficiently 'powered' fits after quality control underwent downsampling to a fixed read depth of 15 reads per bin per tumour copy which acts to normalise inter-sample and

intra-patient variance caused by varying coverage between samples. This manifests as an increased or decreased standard deviation in the bins associated with a given segment. Downsampled absolute copy number profiles were then generated and selected in the same manner as described previously, with selected fits used for downstream analysis.

Lastly, selected downsampled fits were subject to profile variance filtering. While the downsampling process seeks to reduce the amount of intra-patient and inter-sample bin variance, some absolute copy number profiles still retain a high degree of bin variance across their selected copy number fit. The standard deviation for bin values across copy number states 1, 2, 3, and 4 where calculated and samples with standard deviations of all bin values across all copy number states exceeding 3 standard deviations above the mean were removed, excluding four samples from downstream analysis (IM_159, IM_181, JBLAB-19324, JBLAB-4121).

Copy number analysis

Copy number event calling

Copy number events were defined as a given segment in a copy number profile, under the assumption that each segment called as a CN event is independent of other neighbouring segment changes. For the analysis of CNA (both focal/gene-level and broad) copy number thresholds were used as defined by COSMIC and Allele-specific copy number analysis of tumours[13].

Average genome ploidy ≤2.7

- Amplification: total copy number ≥5
- Deletion: total copy number = 0

Average genome ploidy >2.7

- Amplification: total copy number ≥9
- Deletion: total copy number < (average genome ploidy - 2.7)

For gene-level extending across more than one 30 kb genome bin, a mean was taken of all intersected bins. Broad events are defined on the basis of the proportion of affected cytoband with a threshold of 80% called as either amplified or deleted. For arm-level events a threshold of 50% of a chromosome arm (as a proportion of supporting bins) was selected. Copy number events which were equal to plus or minus one from a sample ploidy, but not called as amplification or deletions, were termed gains and losses, respectively.

Ploidy changes

Ploidy changes are difficult to assess due to the nature of absolute copy number fitting, where an incorrectly selected ploidy-purity combination would appear as change in ploidy between the diagnosis and relapse samples. Here, the patients with suspected ploidy changes underwent a scoring methodology to assess the likelihood of a true ploidy change versus a technical error during absolute profile fitting. Change in ploidy between samples was defined as;

$$ploidy\ change\ =\ |\ median(r_i)\ -median(d_i)\ |\ \geq\ 1$$

Where i is the patient, r is the ploidy of relapse samples for patient i, and d is the ploidy of diagnosis samples. Where multiple samples occur for a given sample group, a median value is taken. An absolute value of one or greater is defined as a patient with a ploidy change.

Ploidy change patients were assessed on the basis of three criteria to determine the confidence to which a ploidy change is likely to be true rather than as a consequence of erroneous or poor quality copy number fitting. These criteria are;

1) Selected fits have the highest scoring quantitative quality metrics compared to other sufficiently powered copy number fits (clonality error, TP53 estimate).
2) No underpowered fits with otherwise acceptable quality metrics are available which would contradict the selected copy number fit.
3) Additional samples, attributed to either diagnosis or relapse groups, support the ploidy change by also conforming to criteria 1 and/or criteria 2.

Meeting any of these criteria provides a given patient ploidy change with one star, with a maximum of three stars for a ploidy change with the maximum confidence. Patients with ploidy change and the assigned rating are detailed in Table S8.

For patient-specific analyses (patient loci clustering & gene correlation and heatmap), these ploidy change samples were excluded from the analysis due to the impact on patient clustering. This can be visualised in Figure S28, where ploidy change patients constitute a large proportion of the more extreme copy number changes between diagnosis and relapse.

Purity differences

As expected, we observed differences in tumour purity values for absolute fitted copy number profiles across different biopsy sites and fixation methods, but purity was still consistent between diagnosis and relapse ($p=0.61$; Mann-Whitney U test), including stratification for platinum-based treatment response (resistant $p=0.57$, sensitive $p=0.80$; Mann-Whitney U test)(supplementary information - Figures S5A & S5C). Sample purity remained stable at the cohort-level when only including paired patients between diagnosis and relapse ($p=0.73$; Wilcoxon signed-rank test), including stratification for platinum-based treatment response (resistant $p=1.0$, sensitive $p=0.62$; Wilcoxon signed-rank test)(supplementary information - Figures S5B & S5D).

Intra-tumour heterogeneity

As implemented by van Dijk *et al.*[14], copy number heterogeneity (CNH) is calculated as the minimisation of segment distance from integer state using a ploidy-purity grid search over segment i, where;

$$CNH = min_{\alpha,\tau}\left(\frac{\sum_i \quad d_i w_i}{\sum_i \quad w_i}\right) \qquad (1)$$

Where d_i is the absolute distance of a segment from an integer defined as;

$$d_i = |\ q_i - round(q_i)\ | \qquad (2)$$

Where q_i is the absolute copy number of a segment, α is the sample purity, τ is the average sample ploidy, and w_i is the segment width.

Our implementation forgoes performing a ploidy-purity grid search to determine the lowest chromosomal copy number heterogeneity across as ploidy and purity values for a given sample have already been determined during absolute fitting. As such we calculate CNH (hereto referred to as intra-tumour heterogeneity; ITH) as;

$$ITH = \frac{\sum_i \quad d_i w_i}{\sum_i \quad w_i} \ (3)$$

Where d_i is the absolute distance of a segment from an integer defined in equation 2. Noisy segments were excluded as described by van Dijk *et al.*[14] using the standard deviation of the mean (σ_μ) bin distributions across each segment.

Noise thresholds were set at 2 standard deviations greater than the mean noise, where cutoffs were set to a threshold of $\sigma_\mu > 1.48$, and $\sigma_\mu > 0.875$, for segments and samples, respectively. Noise thresholds removed nine samples (3.4%)

and 685 segments (1.37%, mean and median of 2.59 and 1.00 segments per sample, respectively). After sample exclusion, segment filtering removed 467 segments (0.97%, mean and median of 1.82 and 1.00 segments per sample, respectively). (Figure S29).

Copy number signature abundance modelling

Partial ILR-Bernoulli model

Compositional data are defined by their sum constraint (exposures add up to one) and positivity (exposures are equal or larger than zero), therefore, any regression methods used to analyse them have to be appropriate for a multivariate compositional response. The basis of compositional data analysis[15] is that a compositional vector of length d can be transformed to an unconstrained vector in R^{d-1} without loss of information, and removing the sum-constraint. Here, as we have described previously[16], we use the Isometric Log-Ratio (ILR) transformation[17], in which we use an orthonormal basis to transform the data.

A further challenge in copy number exposure data is the presence of zero values. We address it by using a variant of the ILR transformation, the partial ILR, in which only non-zero values are taken into account. The presence or absence of signatures is analysed using a Bernoulli model. We introduce mixed effects in both models to capture the information about paired diagnosis and relapse samples. The models are implemented in Template Model Builder[18] and run through R.

Model interpretability

The transformation of compositional data is adequately explained as follows; instead of analysing signatures s1 through s7, we analyse the following signature comparisons after ILR transformation of s1 vs s2, s3 vs the mean of s1 and s2, s4 vs the mean of s1-s3, and so forth. This leads to a total of six pairwise comparisons (Figure S30). The means of comparison is by taking the log-ratio of signatures (or groups of signatures), and we use the geometric mean to group signatures.

The two parameters of interest in the model are the intercept and the slope. Both are vectors of length 6 (i.e. as many as comparisons). The intercept indicates, in transformed space, the abundance of signatures in the first group of samples. This intercept can be transformed back to compositional data (using the inverse ILR transformation) to get the mean abundance of signatures in the first group. The slope is the difference in signature abundance between the groups, in transformed space. Therefore, the sum of the intercept and the slope gives us the mean abundance of signatures in the second group, in transformed space. A slope of zero indicates that the exposures are not different between groups.

As an example, Figure S31 shows the intercept and slope for a scenario in which there are three mutational signatures (therefore, they are both vectors of length two). For the intercept, the first ILR is close to zero, indicating that the log-ratio between s1 and s2 is close to zero, and that therefore the mean abundance of s1 and s2 in the first group is roughly the same. ILR2 is negative, indicating that the abundance of S3 is lower than the geometric mean of s1 and s2. For the beta slopes, that of ILR1 is slightly negative, indicating that the ratio between s1 and s2 is a bit lower in the second group than in the first group. The slope for ILR2 is positive, indicating that s3 is more prevalent in the second group.

Immune environment from copy number signatures

Sample preparation

Tissue microarrays (TMA) were created using 1mm cores from viable archival formalin-fixed paraffin-embedded blocks of archival samples of the BriTROC study. Three representative cores were taken from each block. 3µm sections of the TMA blocks were cut using a Leica microtome, transferred to a water bath pre-heated to 60°C and collected on

SuperFrost Plus glass slides. The sections were dried overnight at 37°C then baked at 60°C for 1h to remove excess wax.

Automated Staining

Automated staining was carried out using the Ventana Discovery Ultra platform. All bulk reagents used were purchased from Roche. Sections were rehydrated by incubating them in EZ prep solution for 32min at 69°C, then antigen retrieval was performed by incubating the sections for 1h in Ventana Cell Conditioning buffer 1 (CC1) at 96°C (pH 8.5). After 4min incubation in hydrogen peroxide anti-human primary antibodies were applied and incubated for 1h at 37°C (CD3, Roche 790-4341, prediluted; CD8, Spring Bioscience M5394, 0.5ug/ml; Pan-Keratin, Roche 760-2135, prediluted). After a 16 min incubation with an HRP-conjugated secondary antibody, the chromogenic signal was developed in either 3, 3'-Diaminobenzidine (DAB) chromogen for 8 min, in Purple chromogen for 40 min, in Yellow chromogen for 28 min or in Teal chromogen for 8 mins. For chromogenic counter-staining, the slides were incubated for 4 min in Copper, 8 min in haematoxylin and 4 min in Bluing Reagent. Stained slides were dehydrated using the Leica Autostainer ST020, manually cover-slipped and digitally scanned by Aperio Scanscope XT. Antibodies were validated in the Cancer Research UK Cambridge Institute Pathology Core by staining human lymphoid tissue and confirming appropriate staining location with a pathologist. As the markers are CD8 and CD3. A non-lymphoid tissue was used as a negative control. The antibodies were further validated by multiplex staining, where the overlap between CD3 and CD8 was confirmed.

Image analyses

Analysis of IHC images was done using the HALO Image Analysis Platform (Indica labs). Nuclear segmentation was performed using haematoxylin staining intensity, and cell margins were defined using watershed. The number of cells positive for CD8, CD3, FOXP3 or CD20 was quantified by setting a minimum intensity threshold for each chromogen, and the density was then calculated per area of tissue (Global density). Immune cell subtypes were obtained defined as: CD8+ (all CD8+ T cells); CD8- (CD3+ CD8− T cells); CD3 (The total density of CD3+ cells, inferred from the sum of CD8+ and CD8− cell densities). Images were classified based on pan-keratin staining pattern into tumour (panK+) and stroma (panK-), and each cell density was calculated per region of interest. To test the accuracy of the tissue classifiers, 237 classified images were scored by a pathologist; a score of 1 was given for >90% accuracy, 2 for 70–90% and 3 for < 70% accuracy. 76% (n=179) of images scored 1, 16% (n=39) scored 2 and 8% (n=19) scored 3, indicating fair accuracy of the classifiers. Global (GD), tumour (TD) and stromal (SD) density of each cell type were obtained by dividing the cell count in that location by the area of the same location.

Quantification of tumour immunohistochemistry markers

After quantification of marker positive cells across fixed image areas, grouped by stromal, tumour, and all tissue, cell counts were normalised to a marker-positive cells per micrometre squared (cells/μm^2) and were further rescaled into log(1+ X) where X is cells/μm^2 to account for extreme positive counts and zero counts for each image.

Supplementary Figures

A

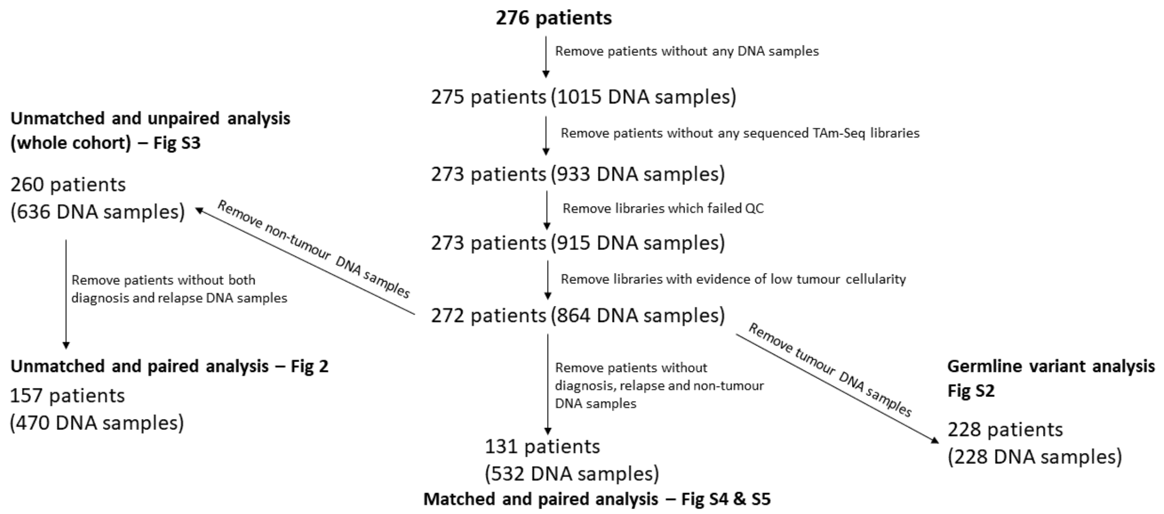

B

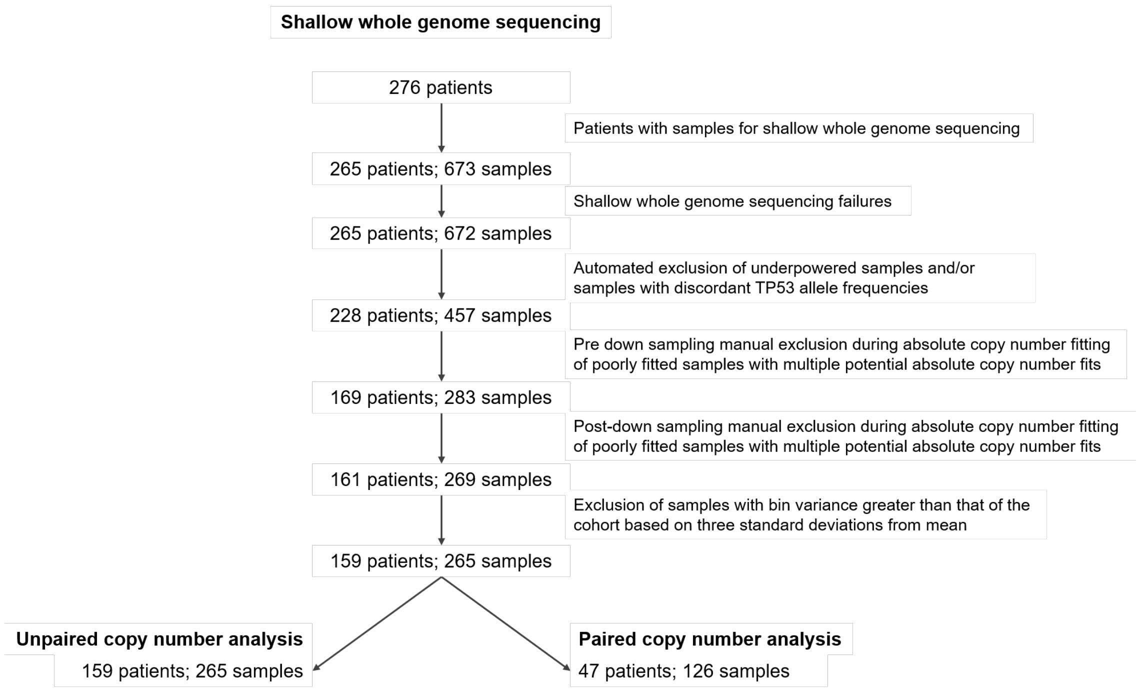

Figure S1. BriTROC study REMARK diagrams

***A** - Patient/sample flow through the single nucleotide variant analysis pipeline, including patient/sample exclusion rationale and pipeline end points.*

***B** - Patient/sample flow through the shallow whole genome sequencing pipeline, including patient/sample exclusion rationale and pipeline en*

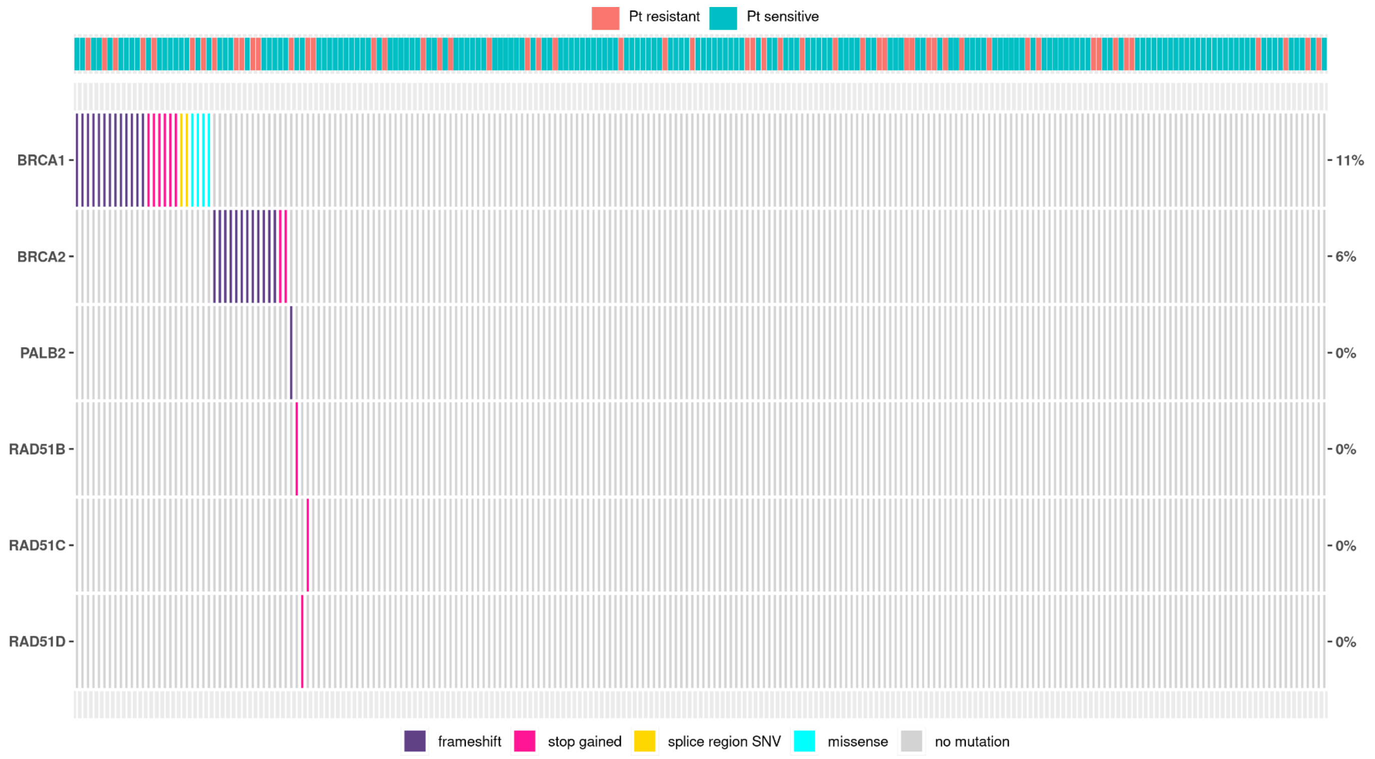

Figure S2. Germline SNVs and short indels identified in key homologous recombination pathway genes

Germline DNA extracted from whole blood samples from 228 BriTROC-1 patients was tested for short variants in key HR genes. Each column represents one patient, colour coded to denote patient platinum sensitivity status at study entry. The lower legend denotes variant type. FANCM and BARD1 were also tested, but no mutations were identified for any patient

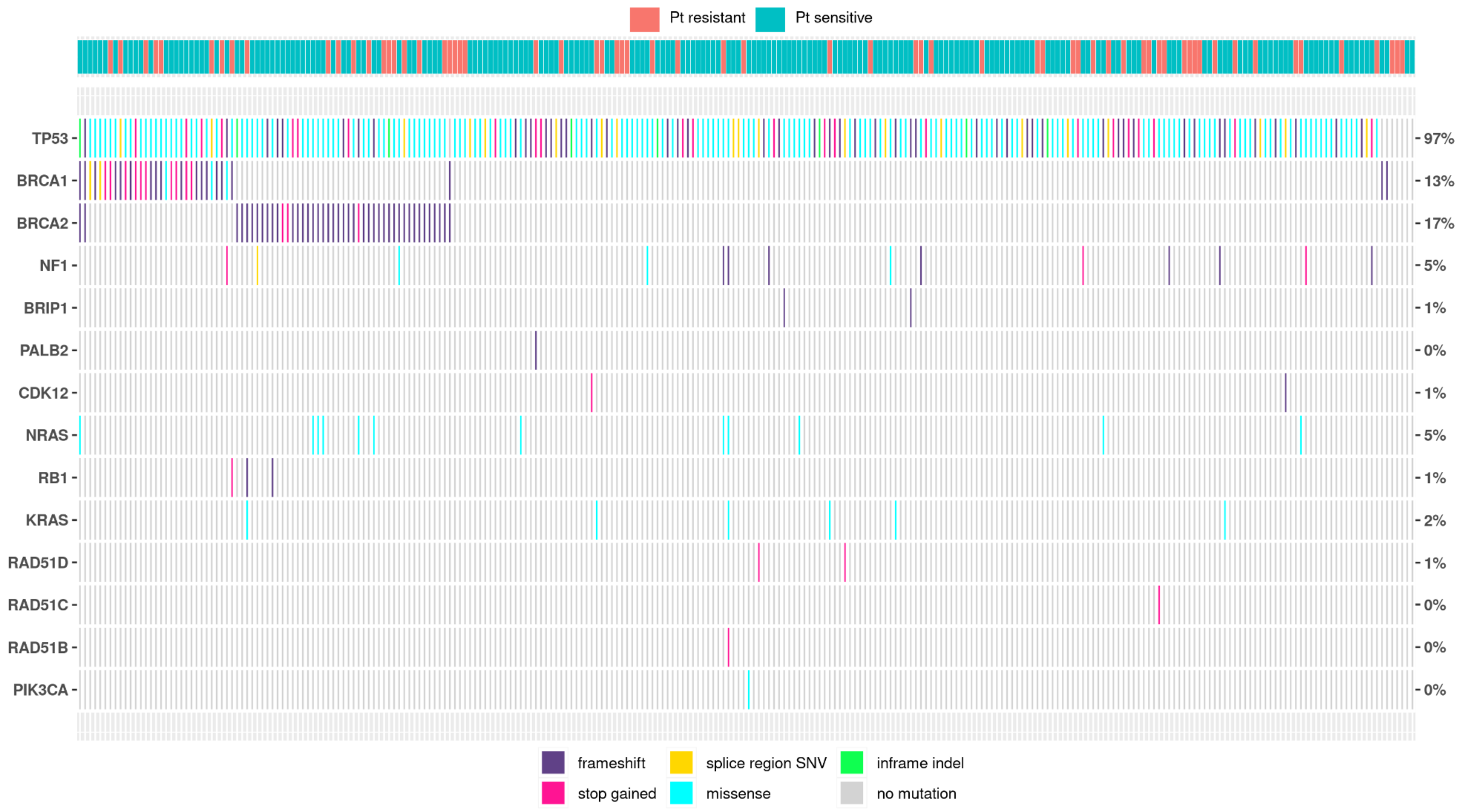

Figure S3. Whole cohort-level detection of SNVs and short indels in key cancer related genes (unpaired)
DNA samples extracted from all tumour samples (both diagnosis and relapse) from 265 patients were tested for short variants in 20 relevant cancer genes. Mutations were not classified as somatic or germline in this analysis nor classified by relapse status (diagnosis vs relapse). Samples were not matched with corresponding normal DNA for each patient. The lower legend denotes variant type. EGFR, FANCM, RAD51C, PALB2, BRAF and CTNNB1 were also targeted, but no mutations were identified.

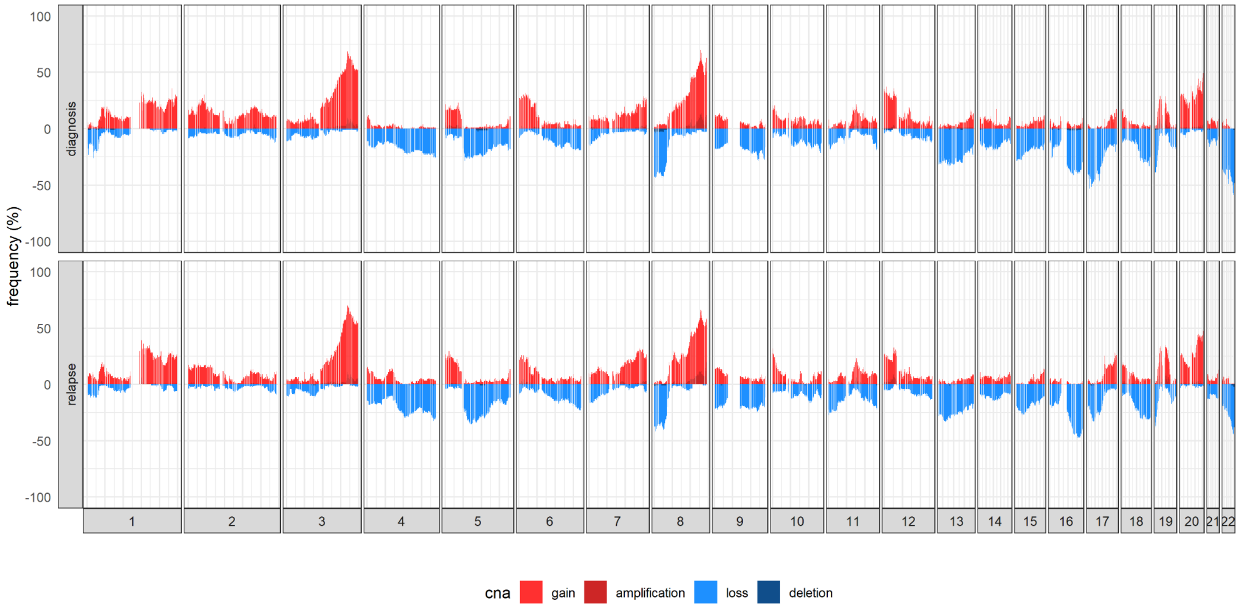

Figure S4. Genome-wide copy number alteration frequency plot

A summary plot of the genome-wide frequency of absolute copy number alterations across diagnosis and relapse samples. Red indicates an increase in genomic copies (defined as either gains or amplifications) and blue indicates a decrease in genomic copies (defined as either losses or deletions). This plot demonstrates the genomic similarities between the alteration frequency of diagnosis and relapse cohorts (n = 126 & n = 139, diagnosis and relapse samples, respectively).

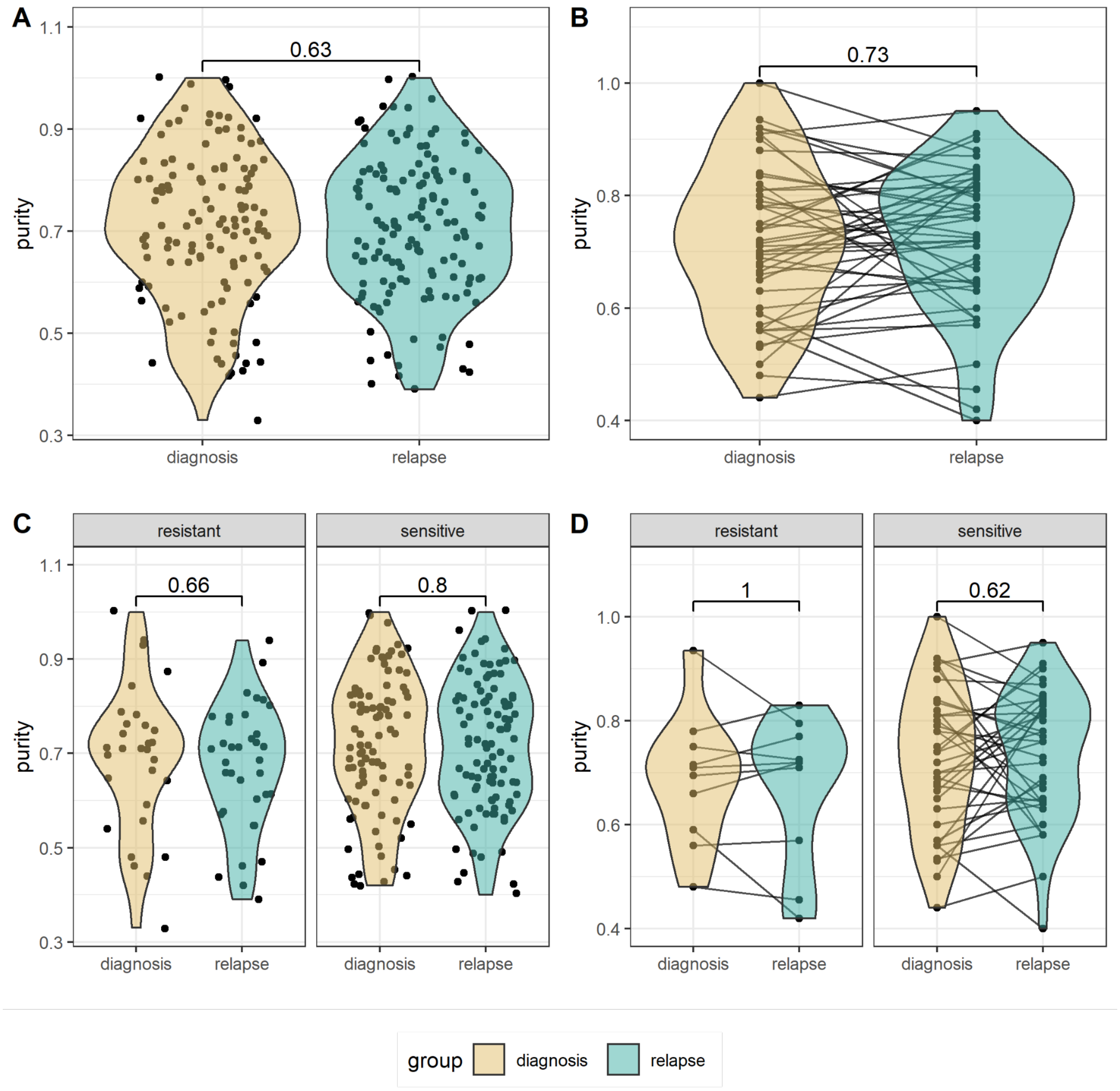

Figure S5: Purity distributions for diagnosis and relapse tumours

Distribution of fitted purity values for each sample identified during absolute copy number fitting (tested using two-sided Mann-Whitney U and Wilcoxon signed-rank for unpaired and paired groupings, respectively).

***A** - All samples between diagnosis and relapse (n = 126 & n = 139, respectively).*

***B** - Paired samples between diagnosis and relapse (n = 47 pairs).*

***C** - All samples between diagnosis and relapse, stratified by platinum sensitivity (n = 30, n = 36, n = 96, n = 103, resistant-diagnosis, resistant-relapse, sensitive-diagnosis, sensitive-relapse).*

***D** - Paired samples between diagnosis and relapse, stratified by platinum sensitivity (n = 10 & n = 37, resistant and sensitive, respectively).*

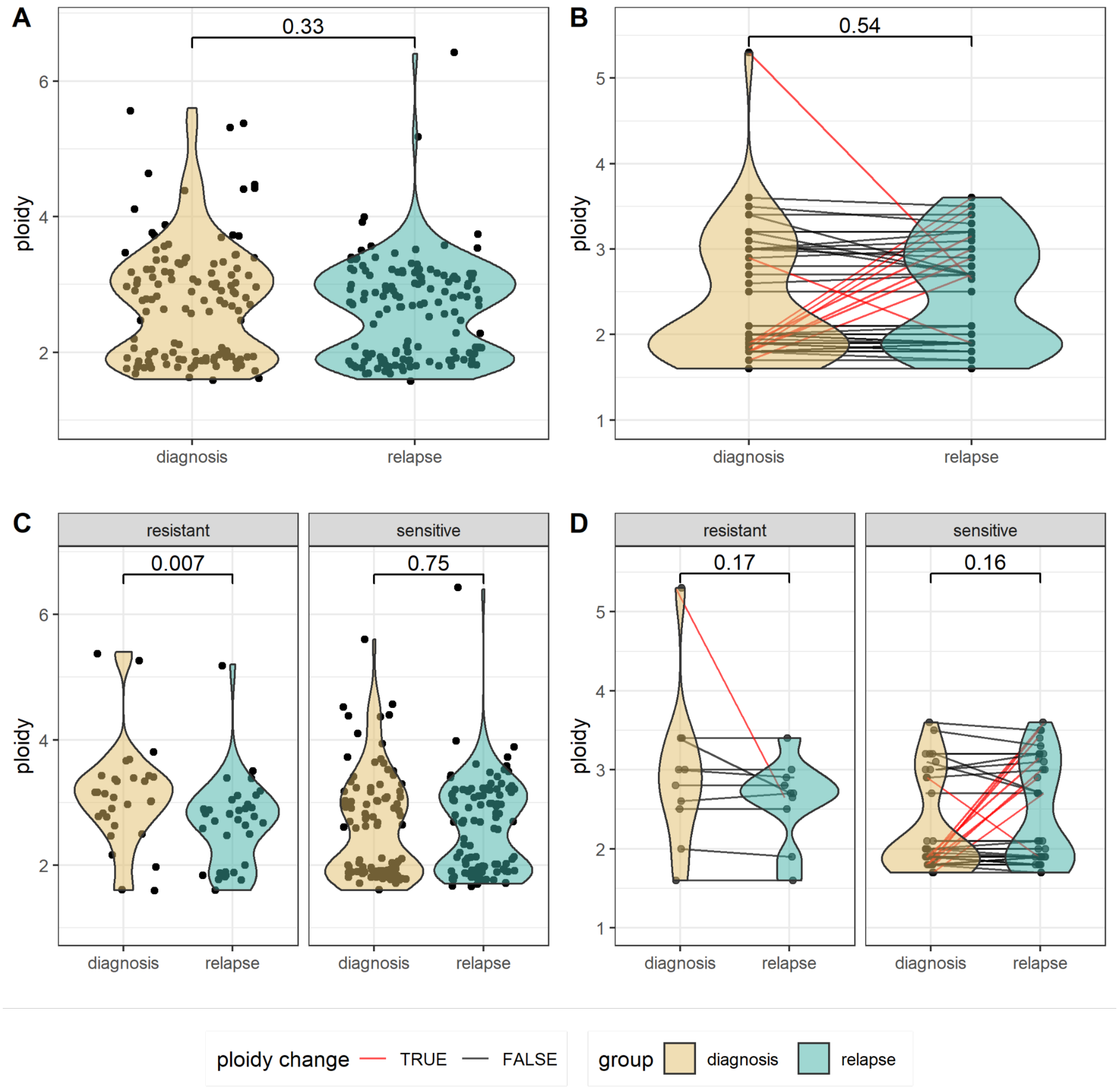

Figure S6. Ploidy distributions for diagnosis and relapse tumours

Distribution of fitted ploidy values for each sample identified during absolute copy number fitting (tested using two-sided Mann-Whitney U and Wilcoxon signed-rank for unpaired and paired groupings, respectively). Samples with ploidy change are marked in red.

***A** - All diagnosis and relapse samples (n = 126 & n = 139, respectively).*

***B** - Diagnosis and relapse samples in paired patients (n = 47 patients).*

***C** - All samples stratified by platinum sensitivity (n = 30, n = 36, n = 96, n = 103, resistant-diagnosis, resistant-relapse, sensitive-diagnosis, sensitive-relapse).*

***D** - Diagnosis and relapse samples in paired patients, stratified by platinum sensitivity (n = 10 & n = 37, resistant and sensitive, respectively).*

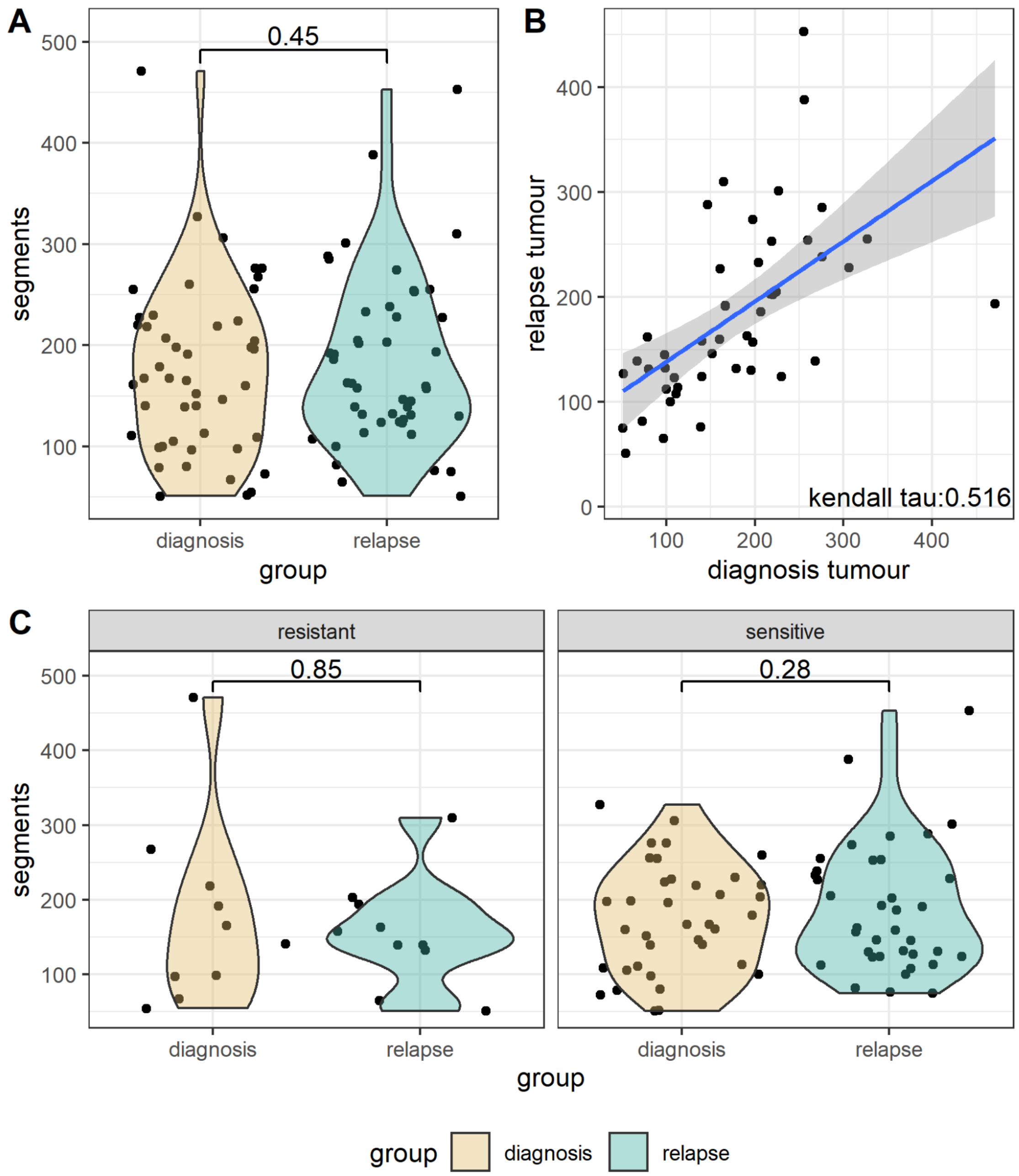

Figure S7. Segment distributions for primary and relapse tumours

A *- Comparison of segment counts per sample between primary and relapse samples across paired samples. No statistically significant difference was found between the number of segments in paired primary and relapse samples across patients (n = 47 patients) (p-values are calculated using a two-sided Wilcoxon signed rank test).*

B *- Scatter plot of segment counts for each sample in diagnosis and relapse tumours in paired patients. Segments were averaged across samples where multiple samples were available for any patient in either the diagnosis or relapse group. Blue line indicates the linear regression line and the shaded portion is the upper and lower 95% confidence interval of the standard error of the mean. Correlation was calculated using Kendall rank correlation (n = 47 patients).*

C *- Comparison of segment counts per sample between paired primary and relapse samples stratified by patient platinum-based treatment sensitivity (resistant; n = 10 & sensitive; n = 37) (p-values are calculated using a two-sided Wilcoxon signed rank test).*

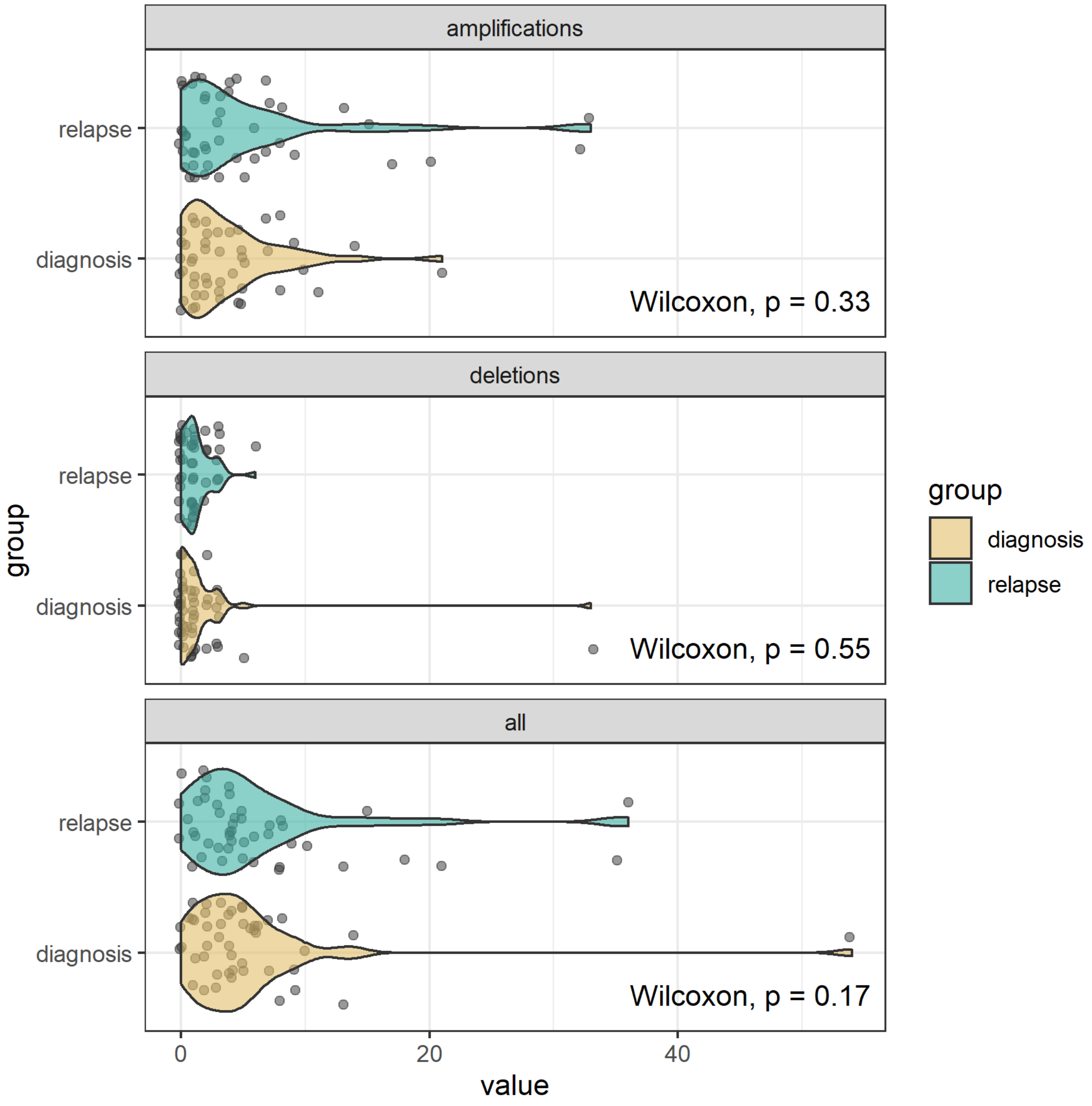

Figure S8. Copy number events

Copy number event distributions calculated using segments as a proxy for a copy number event change between diagnosis and relapse sample groups. Summary plots of copy number events are stratified by event type (all, amplification, and deletion; diagnosis and relapse n = 47 paired patients). Differences in event distributions were tested using a two-sided Wilcoxon ranked-sign.

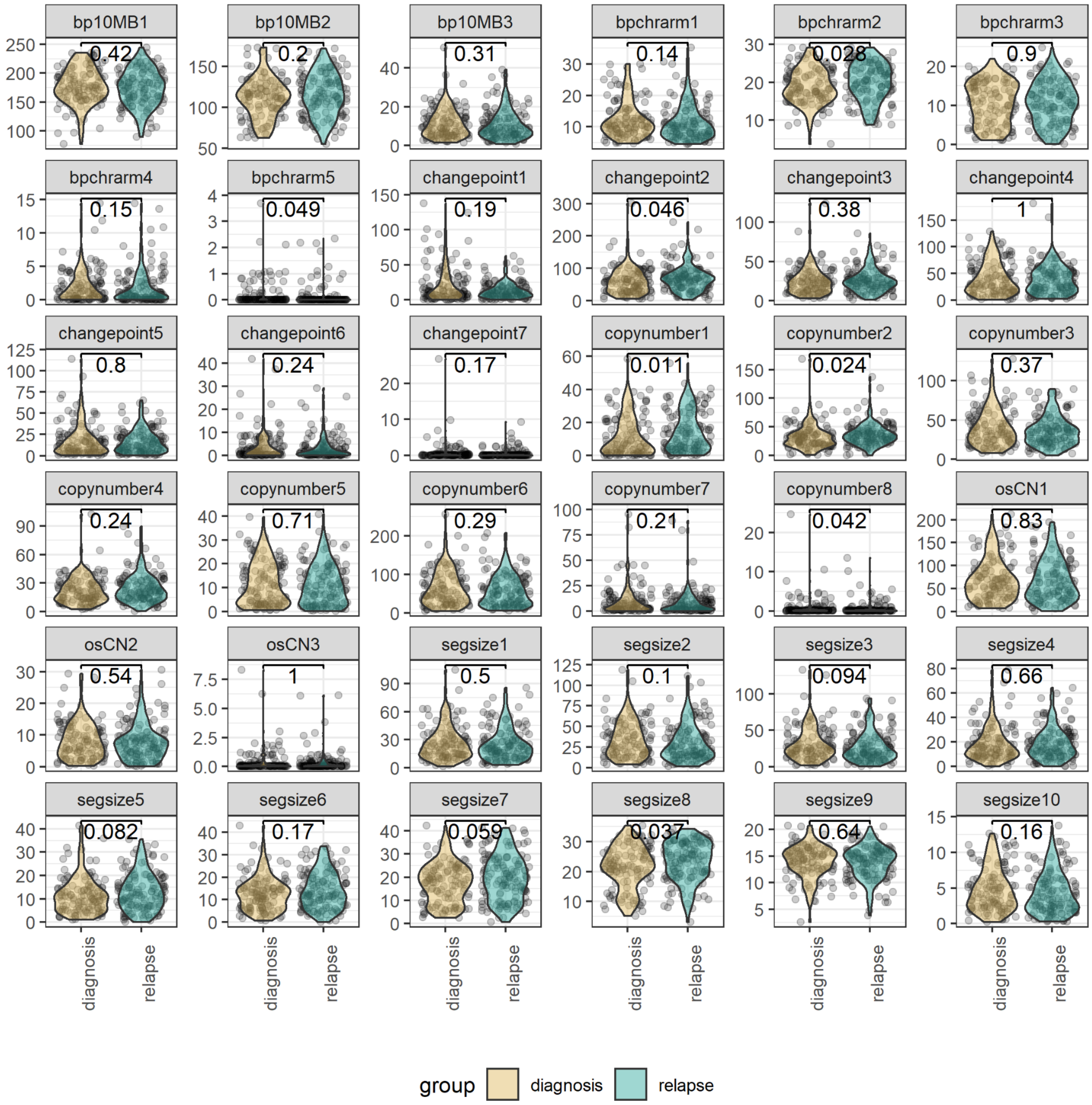

Figure S9. Copy number features

Faceted plot of copy number feature distributions calculated during copy number signature extraction. These are the same copy number features utilised in the derivation of copy number signatures[4] and should therefore provide a robust comparison of the differing copy number processes between diagnosis and relapse samples (n = 126 & n = 139, respectively). Distributions were tested using a two-sided Mann-Whitney U test, without adjustment for multiple comparisons. Of all tested, 7/36 copy number features were determined to be significantly different between diagnosis and relapse but none was statistically different after false discovery rate correction.

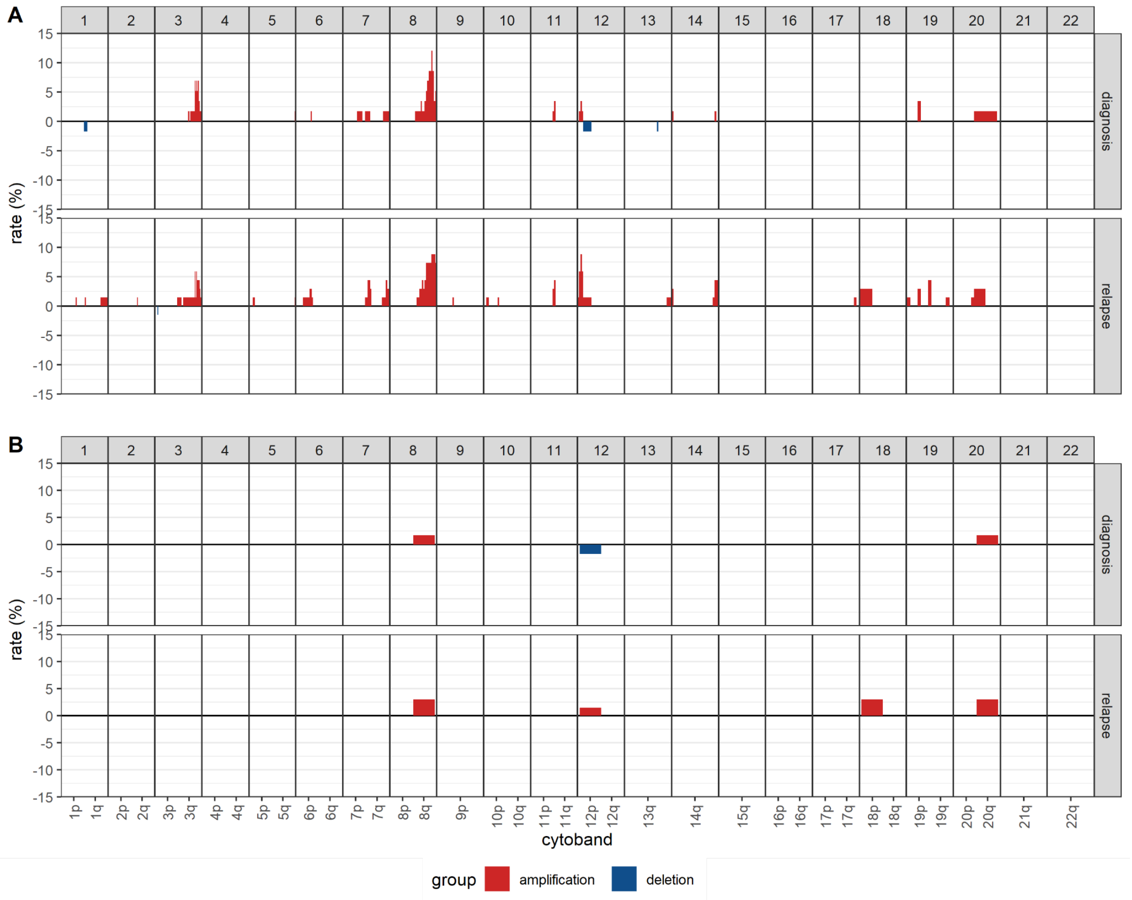

Figure S10: Cytoband and chromosome arm alteration rates

***A** - Copy number alteration rates for cytoband-resolution where each cytoband is assessed independently between diagnosis and relapse tumour groups. X-axis denotes each cytoband across each chromosome (noted by axis facets) where 80% of bins supported a CNA call (n = 126 & n = 139, diagnosis and relapse samples, respectively).*

***B** - Copy number alteration rates for arm-resolution where each arm is assessed independently between diagnosis and relapse tumour groups. X-axis denotes each cytoband across each chromosome (noted by axis facets) where 50% of bins supported a CNA call (n = 126 & n = 139, diagnosis and relapse samples, respectively).*

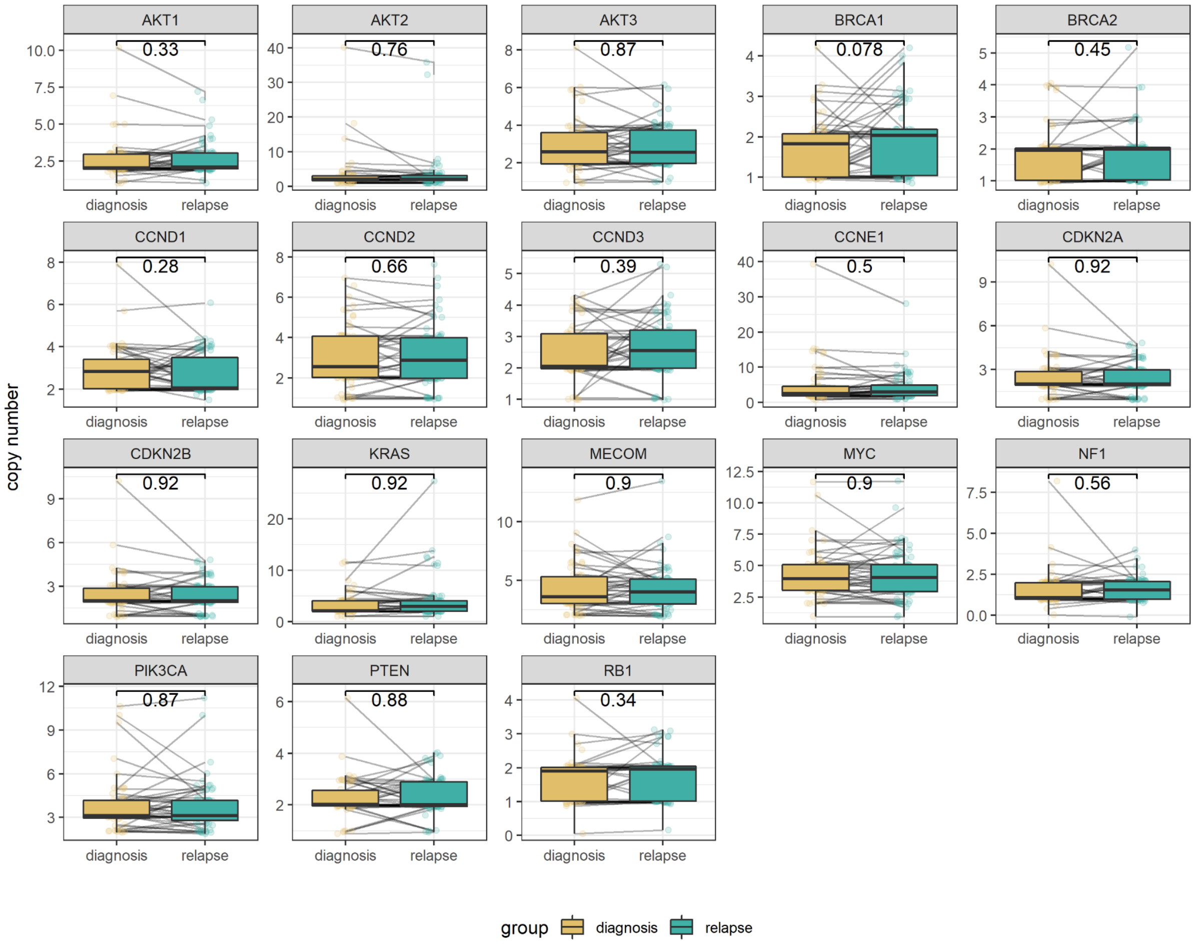

Figure S11. Total gene copies

Absolute copy number state distributions for the 18 frequently altered genes between paired diagnosis and relapse samples (n = 58 & n = 68, diagnosis and relapse, respectively). No statistically significant difference was found between the diagnosis and relapse group when comparing the distributions of copy number states over each gene locus (two-sided Mann-Whitney U test, without adjustment for multiple comparisons). Boxplots show the lower and upper hinges corresponding to the first and third quartiles (the 25th and 75th percentiles). The whiskers extend from the hinge to the largest value no further than 1.5 × interquartile range from the hinge.

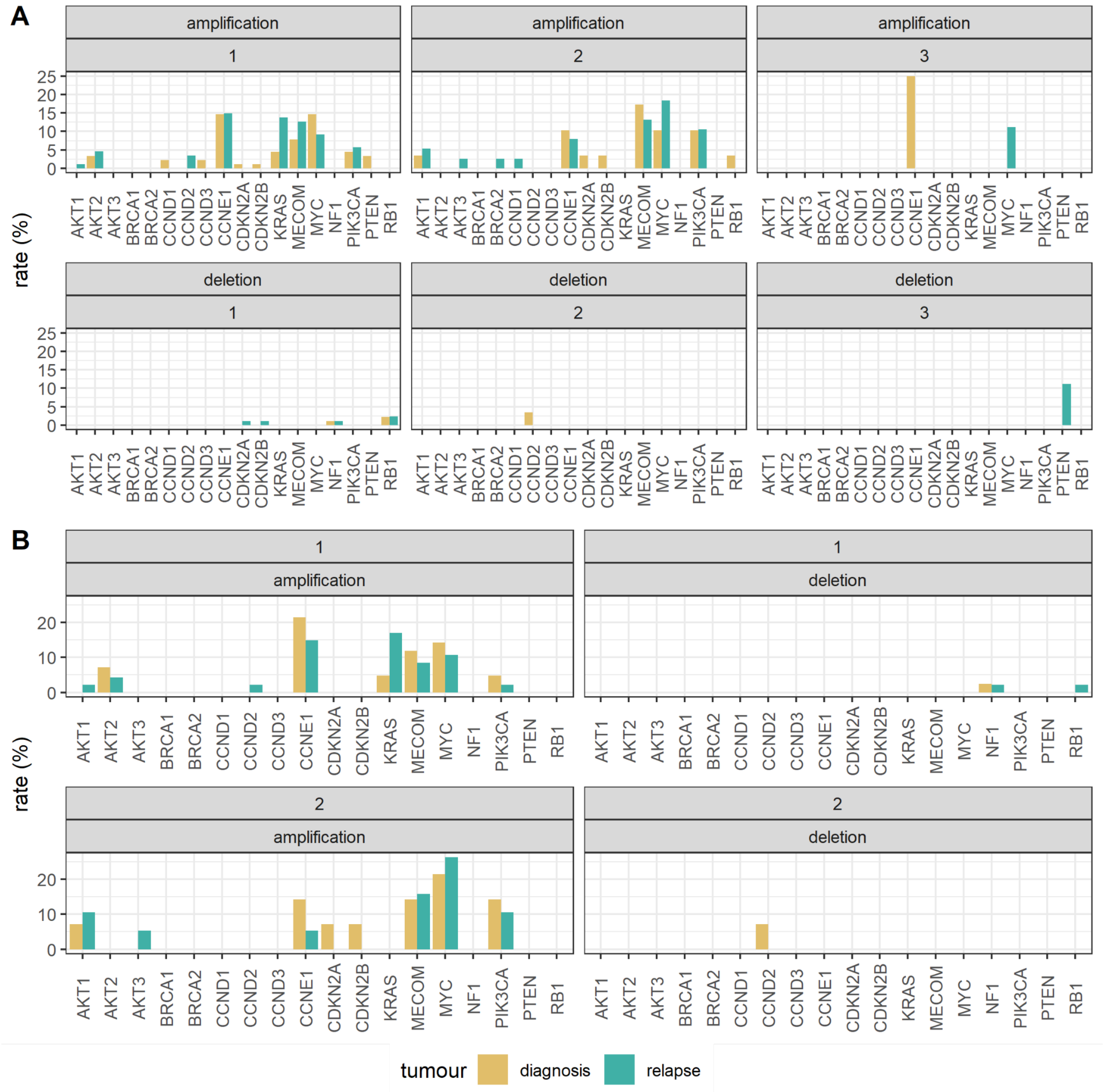

Figure S12. Copy number focal changes in frequently altered genes stratified by prior lines of therapy

***A** - Frequency of focal amplification and deletions in frequently altered genes stratified by diagnosis or relapse and number of prior lines of chemotherapy at study registration in all samples (n = 89 & n = 87, prior lines = 1 diagnosis & relapse, respectively; n = 29 & n = 38, prior lines = 2 diagnosis & relapse, respectively; n = 4 & n = 9, prior lines = 3 diagnosis & relapse, respectively).*

***B** - Frequency of focal amplification and deletions in frequently altered genes stratified by diagnosis or relapse and number of prior lines of chemotherapy at study registration in paired samples (n = 42 & n = 47, prior lines = 1 diagnosis & relapse, respectively; n = 14 & n = 19, prior lines = 2 diagnosis & relapse, respectively).*

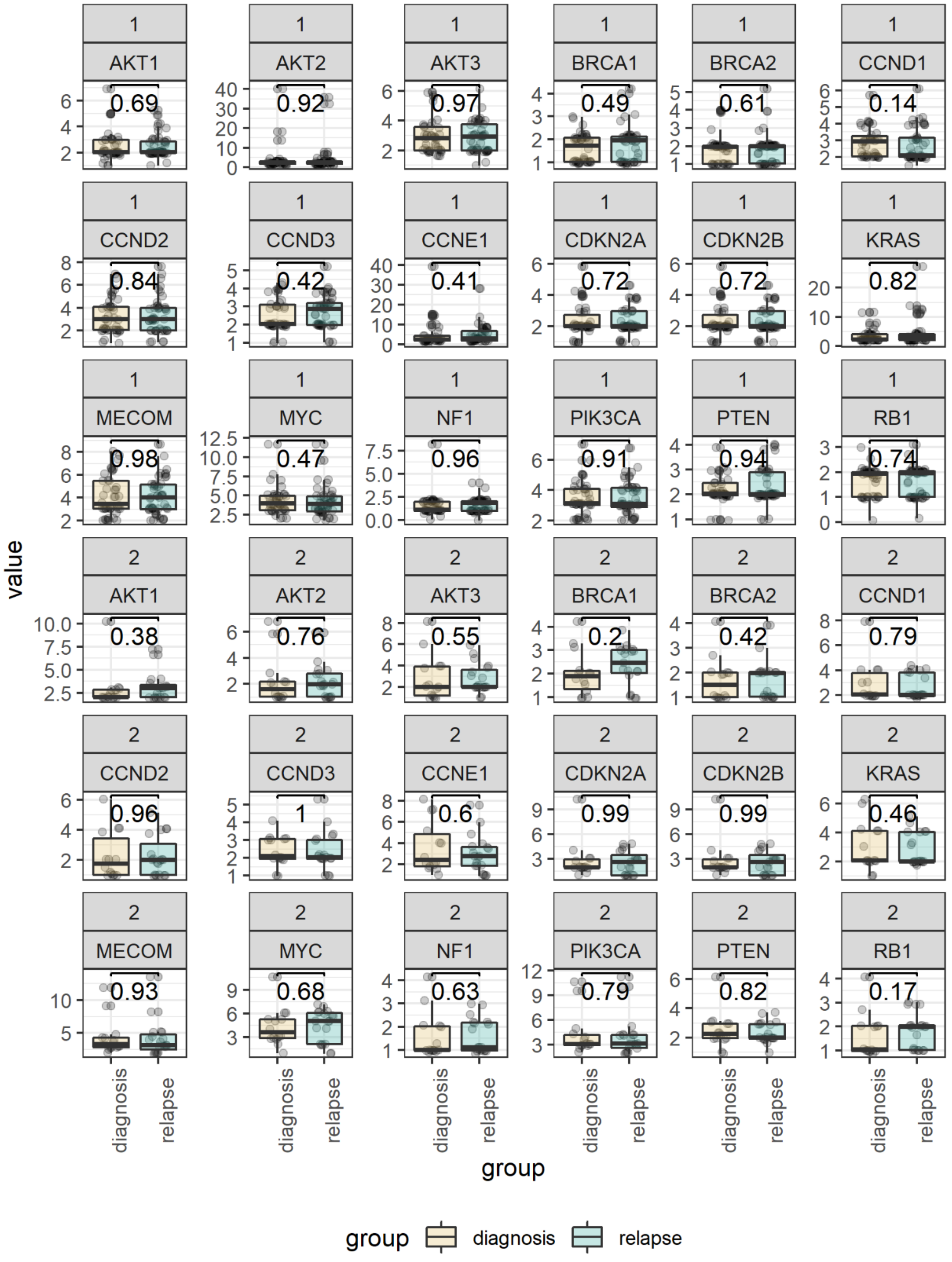

Figure S13. Stratified copy number count by prior lines and tumour timepoint

Faceted boxplot of copy number state distributions for 18 frequently altered genes comparing diagnosis and relapse tumours, stratified by either one or two prior lines of therapy. Displayed p-values are the uncorrected statistical outcome of a two-sided Mann-Whitney U test (n = 42 & n = 47, prior lines = 1 diagnosis & relapse, respectively; n = 14 & n = 19, prior lines = 2 diagnosis & relapse, respectively), without adjustment for multiple comparisons. Boxplots show the lower and upper hinges corresponding to the first and third quartiles (the 25th and 75th percentiles). The whiskers extend from the hinge to the largest value no further than 1.5 × interquartile range from the hinge.

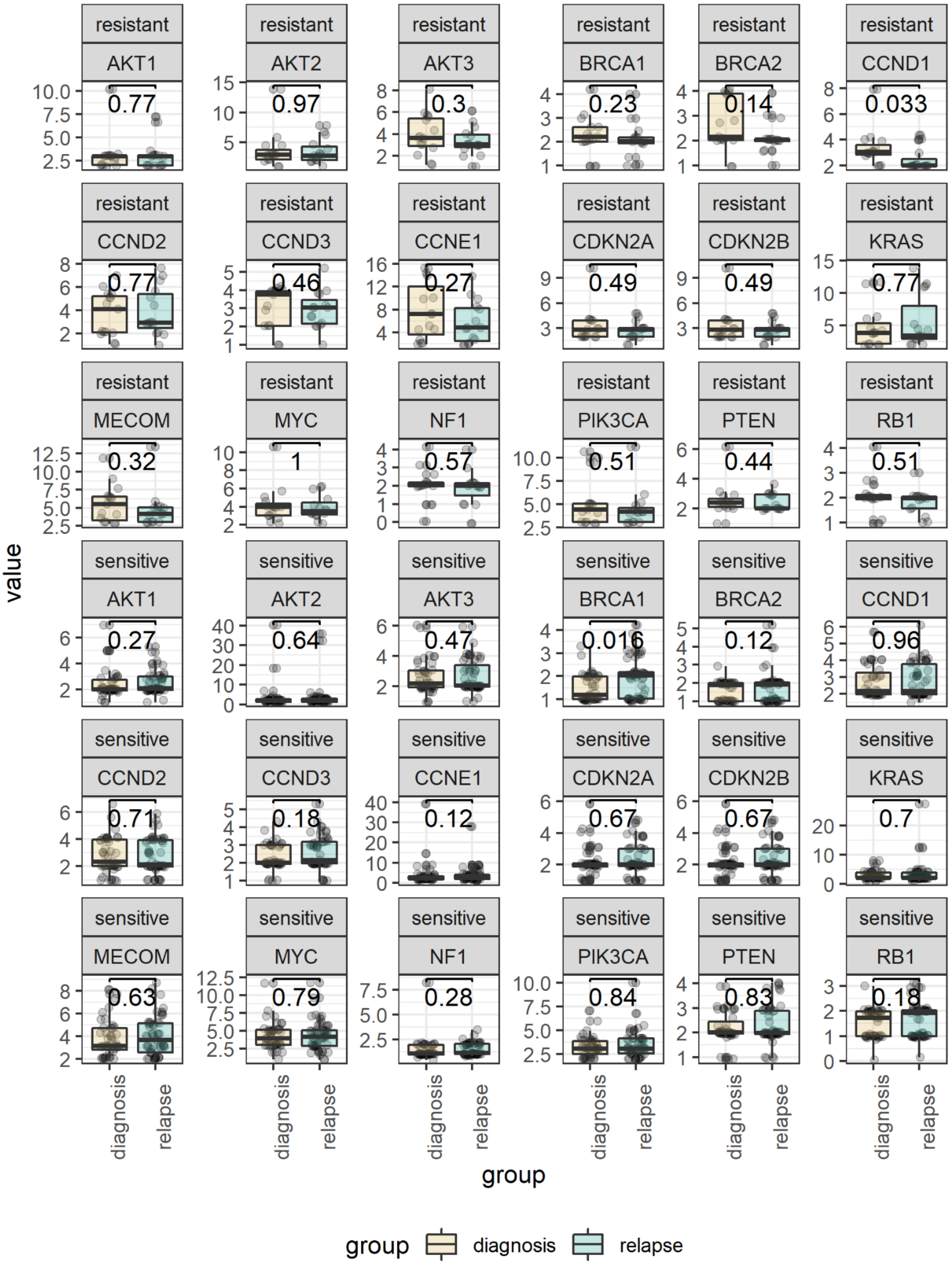

Figure S14. Stratified copy number count by platinum status and tumour timepoint

Faceted boxplot of copy number state distributions for 18 frequently altered genes comparing diagnosis and relapse tumours, stratified by platinum status. Displayed p-values are the uncorrected statistical outcome of a two-sided Mann-Whitney U test (n = 15, n = 15, platinum resistant, diagnosis & relapse, respectively; n = 43, n = 53, platinum sensitive, diagnosis & relapse, respectively), without adjustment for multiple comparisons. Boxplots show the lower and upper hinges corresponding to the first and third quartiles (the 25th and 75th percentiles). The whiskers extend from the hinge to the largest value no further than 1.5 × interquartile range from the hinge.

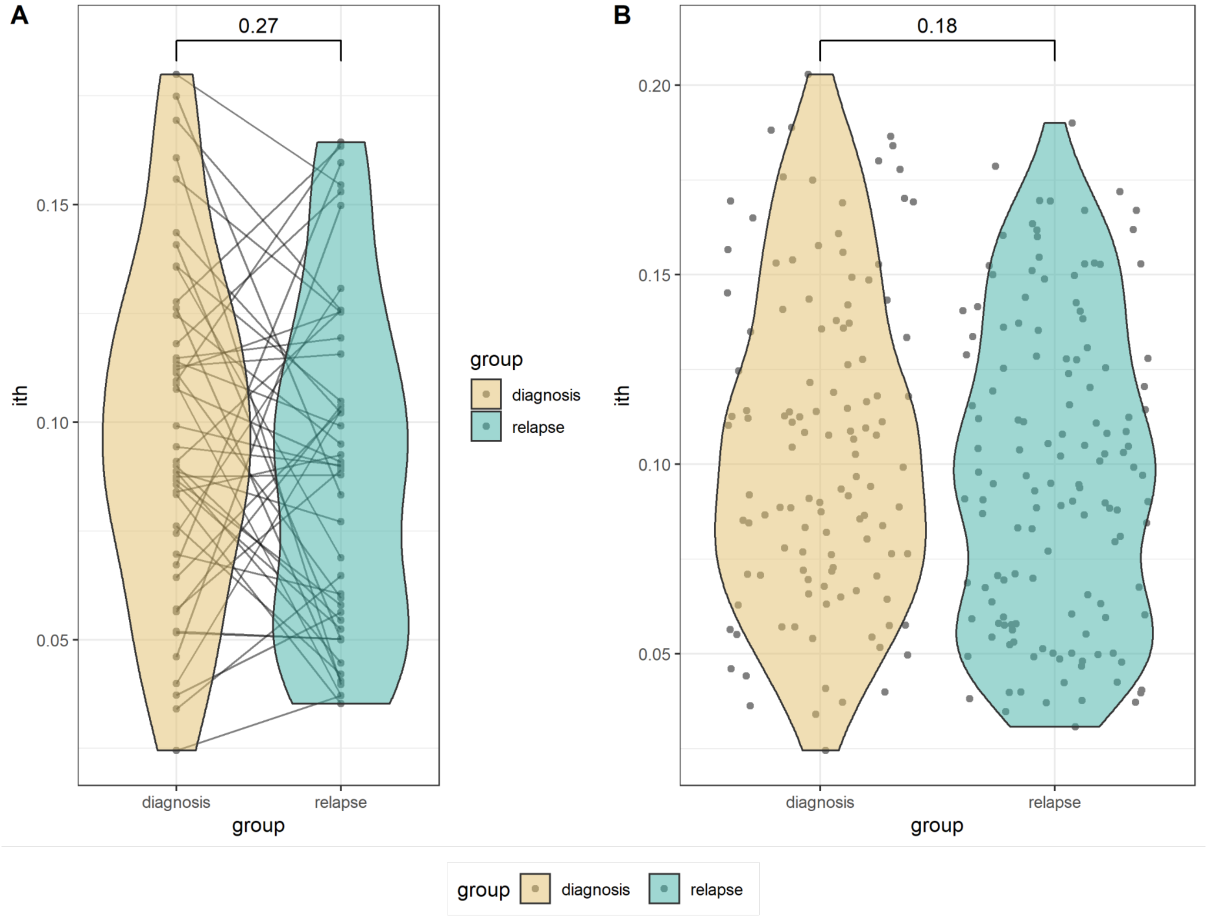

Figure S15. Intra-tumoural heterogeneity

***A** - Violin plot comparing the patient-level comparison of estimated ITH as calculated from integer copy number segment deviations stratified by paired diagnosis and relapse samples (n = 47). Distributions were not shown to be non-statistically significant by two-sided Wilcoxon signed-rank test utilising paired comparison.*

***B** - Violin plot comparing the unpaired sample-level comparison of estimated ITH as calculated from integer copy number segment deviations stratified by diagnosis and relapse samples (n = 119 & 137, diagnosis and relapse, respectively). Distributions were not shown to be non-statistically significant by two-sided Wilcoxon signed-rank test utilising paired comparison.*

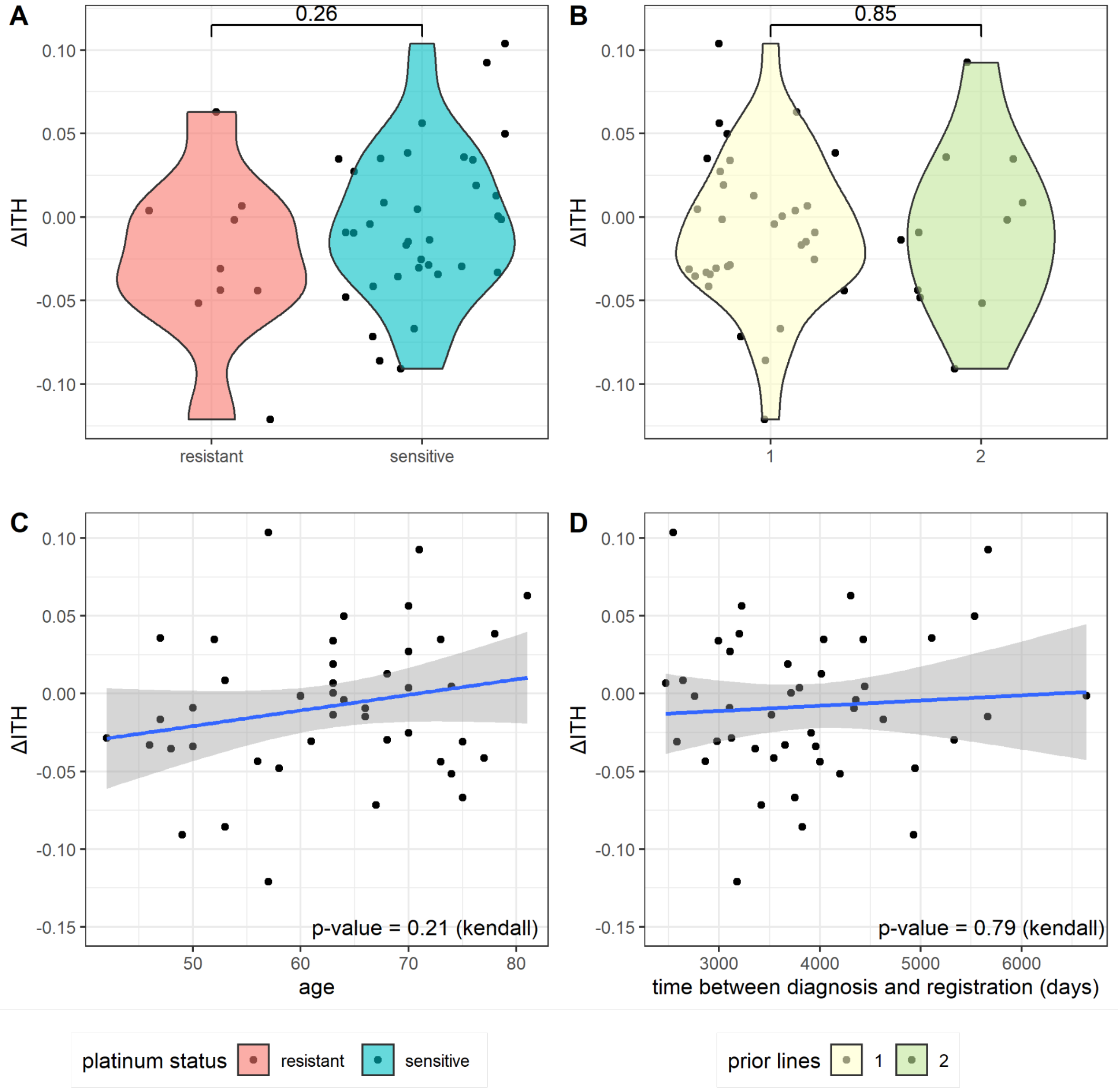

Figure S16. Intra-tumoural heterogeneity change

***A** - Violin plot comparing the patient-level comparison of estimated ΔITH as calculated from integer copy number segment deviations stratified by platinum-based therapeutic resistance or sensitivity (n = 9 & n = 35). Distributions were not shown to be non-statistically significant by a two-sided Mann-Whitney U test.*

***B** - Violin plot comparing the patient-level comparison of estimated ΔITH as calculated from integer copy number segment deviations stratified by prior lines of therapy before study entry (n = 33 & n = 11), two patients had three and four prior lines of therapy, respectively, so were dropped from statistical testing. Statistics shown is a two-sided Mann-Whitney U test.*

***C** - Scatter plot of estimated ΔITH to patient age demonstrating limited correlation of changing tumour heterogeneity with age at diagnosis (n = 44). Blue line indicates the linear regression line and the shaded portion is the upper and lower 95% confidence interval of the standard error of the mean. Statistics shown is a two-sided Kendall rank correlation coefficient test.*

***D** - Scatter plot of estimated ΔITH to distance from diagnosis, as a proxy for sample age. The linear regression demonstrates limited correlation of changing tumour heterogeneity with age at diagnosis (n = 44). Blue line indicates the linear regression line and the shaded portion is the upper and lower 95% confidence interval of the standard error of the mean. Statistics shown is a two-sided Kendall rank correlation coefficient test.*

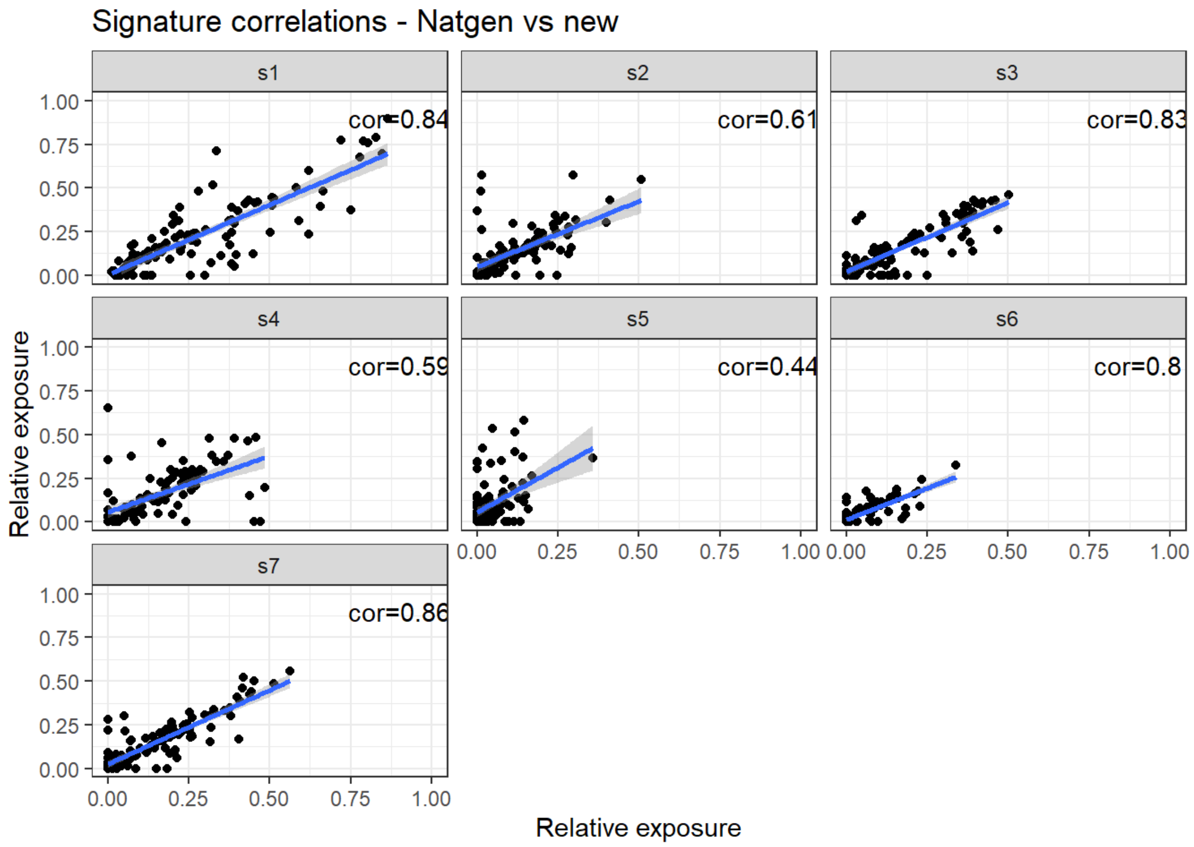

Figure S17. Copy number signature correlations

Correlation between previously generated HGSC copy number signature exposures[4] and HGSC copy number signature exposures generated in this study in samples utilised in both studies. Copy number signatures are broadly correlated, though exposures for any given sample are variable due to different absolute copy number fitting methodologies between this and the previous study[4] (n = 104 samples). Blue line indicates the linear regression line and the shaded portion is the upper and lower 95% confidence interval of the standard error of the mean.

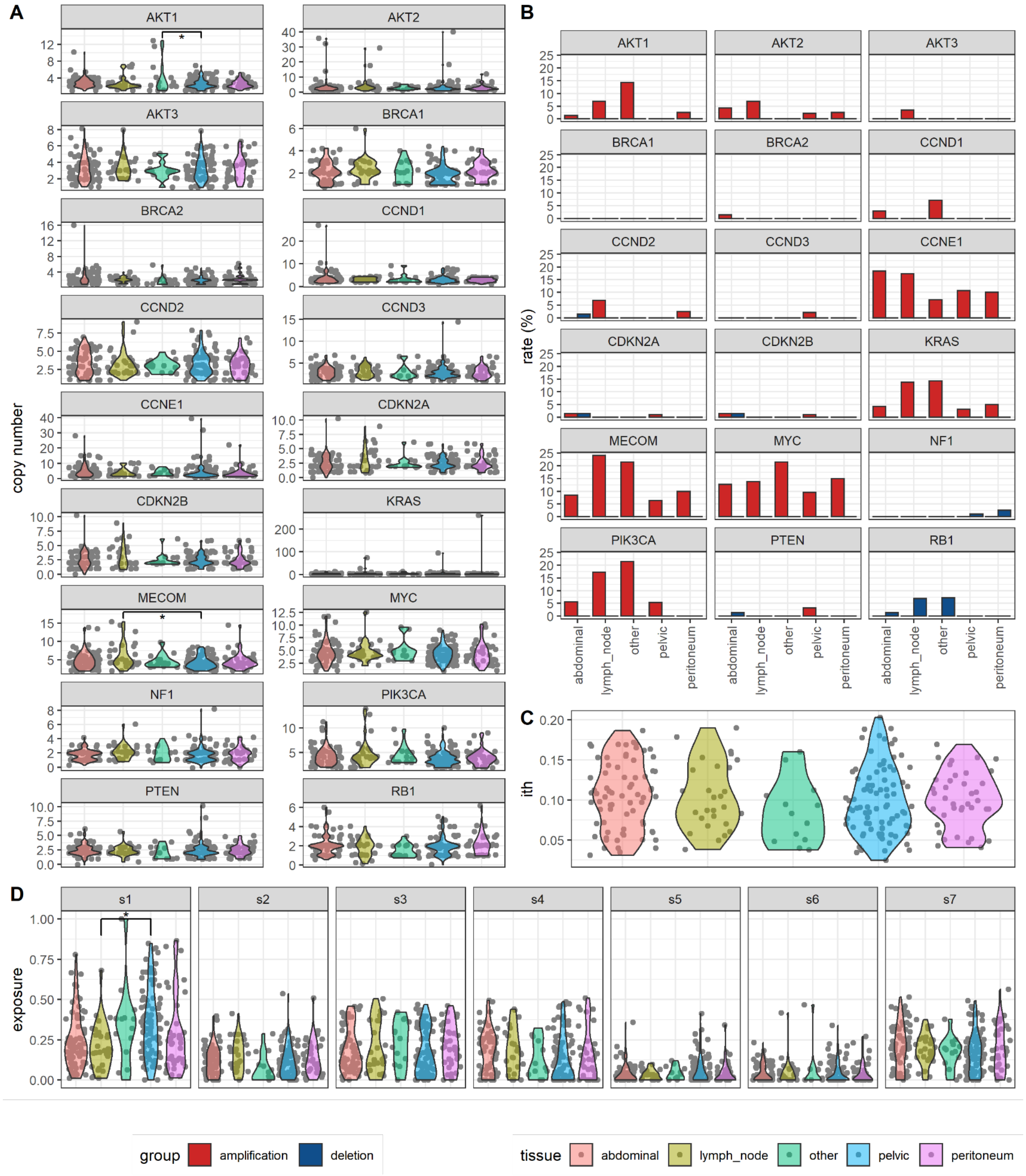

Figure S18. Copy number features by tissue site of origin

***A** - Copy number count summaries of 18 frequently altered genes by tissue site. Violin plots are coloured by tissue and represent the distribution of copy number value for a given gene across all available samples (sample n; intra-abdominal = 71, lymph node = 29, other = 14, pelvic = 94, and peritoneum = 40). The * symbol indicates a p-value less than 0.05 after adjustment multiple comparisons from a one-sided* Tukey HSD test. *Exact p-values are* p=0.006 & p=0.006 for *ATK1 and MECOM*, respectively.

***B** - Copy number alteration rates across 18 clinically frequently altered genes across tumour tissue sites. Bar plots are colour by amplification and deletion rate for a given gene across all available samples (sample n; intra-abdominal = 71, lymph node = 29, other = 14, pelvic = 94, & peritoneum = 40).*

***C** - Distribution of ITH for each sample stratified by available tumour tissue type (sample n; intra-abdominal = 71, lymph node = 28, other = 13, pelvic = 88, and peritoneum = 39).*

***D** - Distributions for each copy number signature. Violin plots are colour by tissue and represent the distribution of copy number signature across all available samples by tumour tissue site (sample n; intra-abdominal = 71, lymph = 29, other = 14, pelvic = 94, and peritoneum = 40). The * symbol indicates a p-value less than 0.05 after adjustment multiple comparisons one-sided* Tukey HSD test. *Exact p-value* p=0.04 for s1.

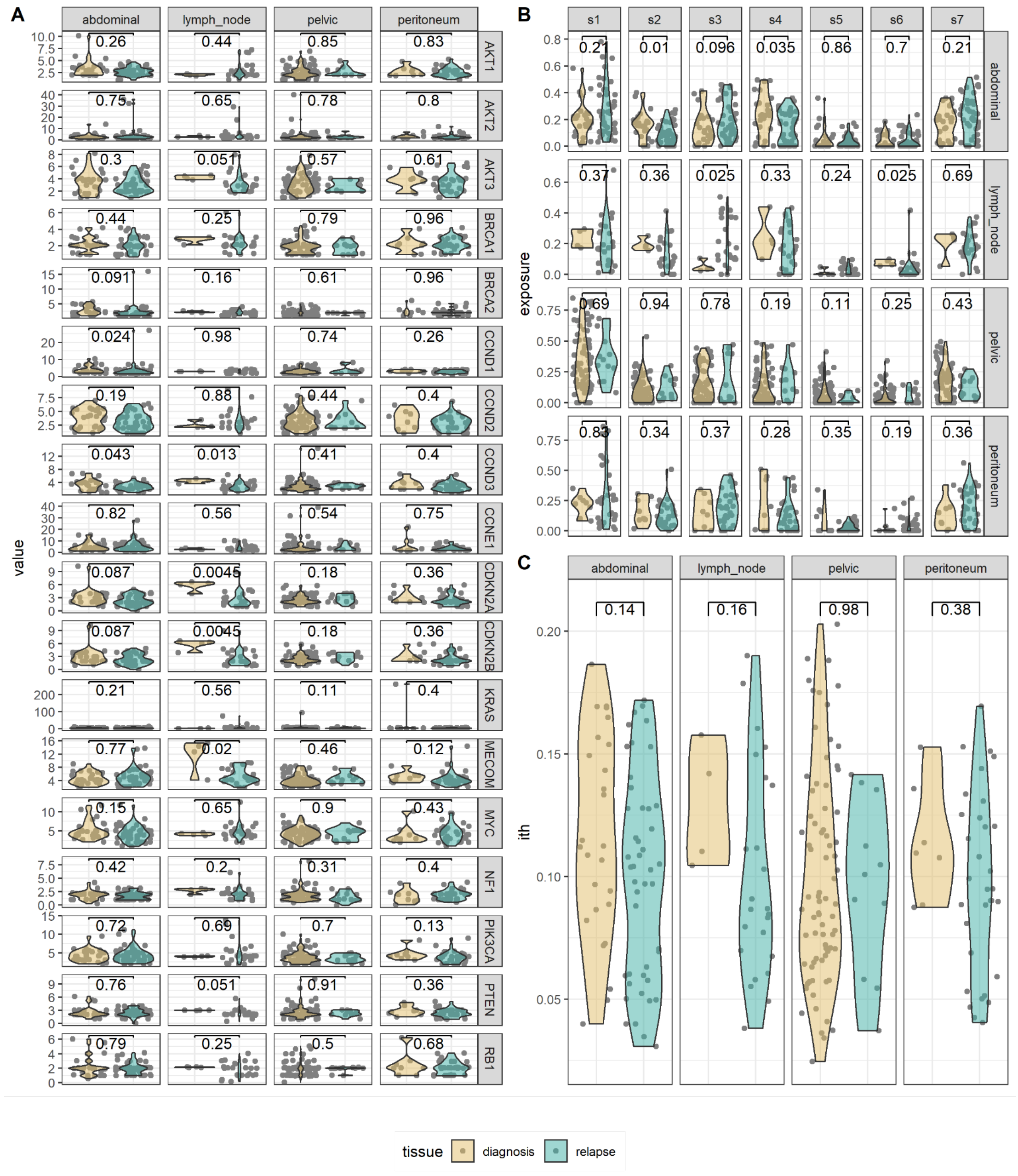

Figure S19. Copy number features stratified by diagnosis and relapse across tissue site of origin

A *- Copy number count summaries across 18 frequently altered genes across tumour tissue site. Box plots are colour by diagnosis and relapse sample status and represent the distribution of copy number value for a given gene (sample n; intra-abdominal = 26 & 45, lymph node = 4 & 25, pelvic = 82 & 12, and peritoneum = 8 & 32, diagnosis and relapse, respectively). Statistics shown is a two-sided Mann-Whitney U test, without adjustments for multiple comparisons.*

B *- Distributions for each copy number signature. Violin plots are colour by diagnosis and relapse sample status and represent the distribution of copy number signature across tumour tissue sites (sample n; intra-abdominal = 26 & 45, lymph node = 4 & 25, pelvic = 82 & 12, and peritoneum = 8 & 32, diagnosis and relapse, respectively). Statistics shown is a two-sided Mann-Whitney U test, without adjustments for multiple comparisons.*

C *- Distribution of ITH for each sample stratified by available tumour tissue type (sample n; intra-abdominal = 26 & 45, lymph node = 4 & 24, pelvic = 77 & 11, peritoneum = 7 & 32, diagnosis and relapse, respectively). Statistics shown is a two-sided Mann-Whitney U test, without adjustments for multiple comparisons.*

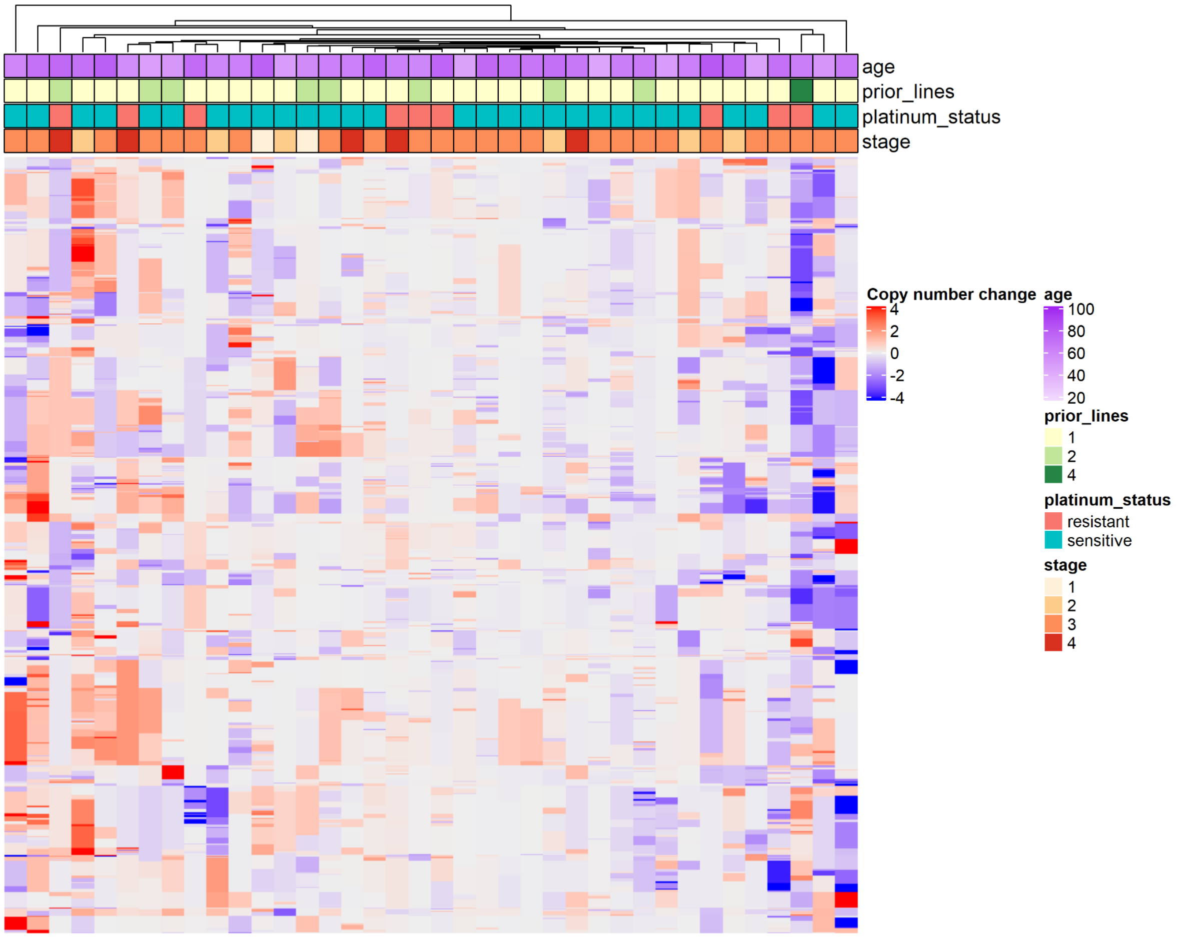

Figure S20. Gene change heatmap and correlation matrix

Heatmap showing the direction of copy number change for gene loci between 47 paired diagnosis and relapse samples at patient level. Rows correspond to the top 20% most variable loci (corresponding to 3623 genes) and columns correspond to patients. Cell colouration represents the change in copy number for a given locus (mean value in the relapse sample minus mean value in the diagnosis sample) (n = 38 patients).

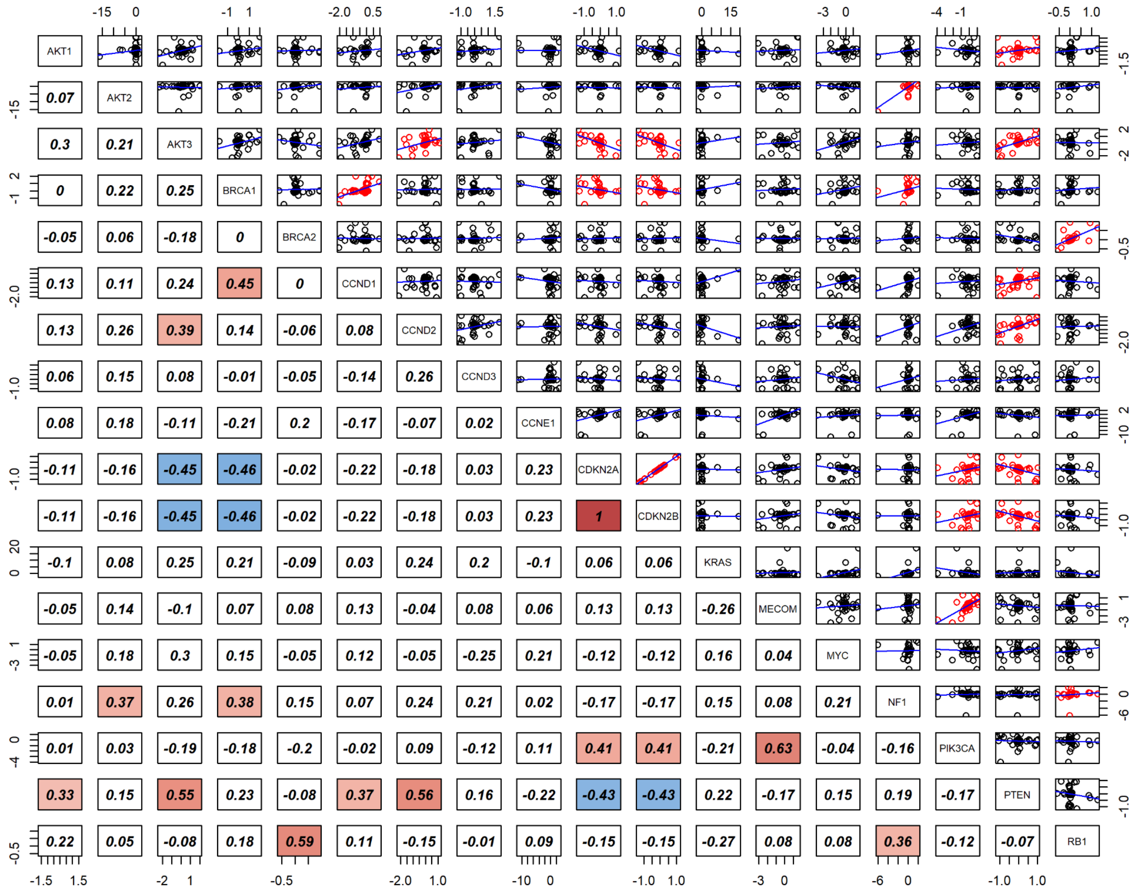

Figure S21. Copy number change correlations in frequently altered genes

Correlation and scatter plot matrix of copy number change correlation between different frequently altered genes. Scatter plots show the point distribution for any given pair of genes labelled in the diagonal. The blue line for each scatter plot in the linear fit for the given set of points; points in red are significantly correlation (spearman rank correlation). Numerical values in the lower portion state the correlation coefficient for a given gene pairing using the same colouration ascribed in Figure 6 (n = 38 patients).

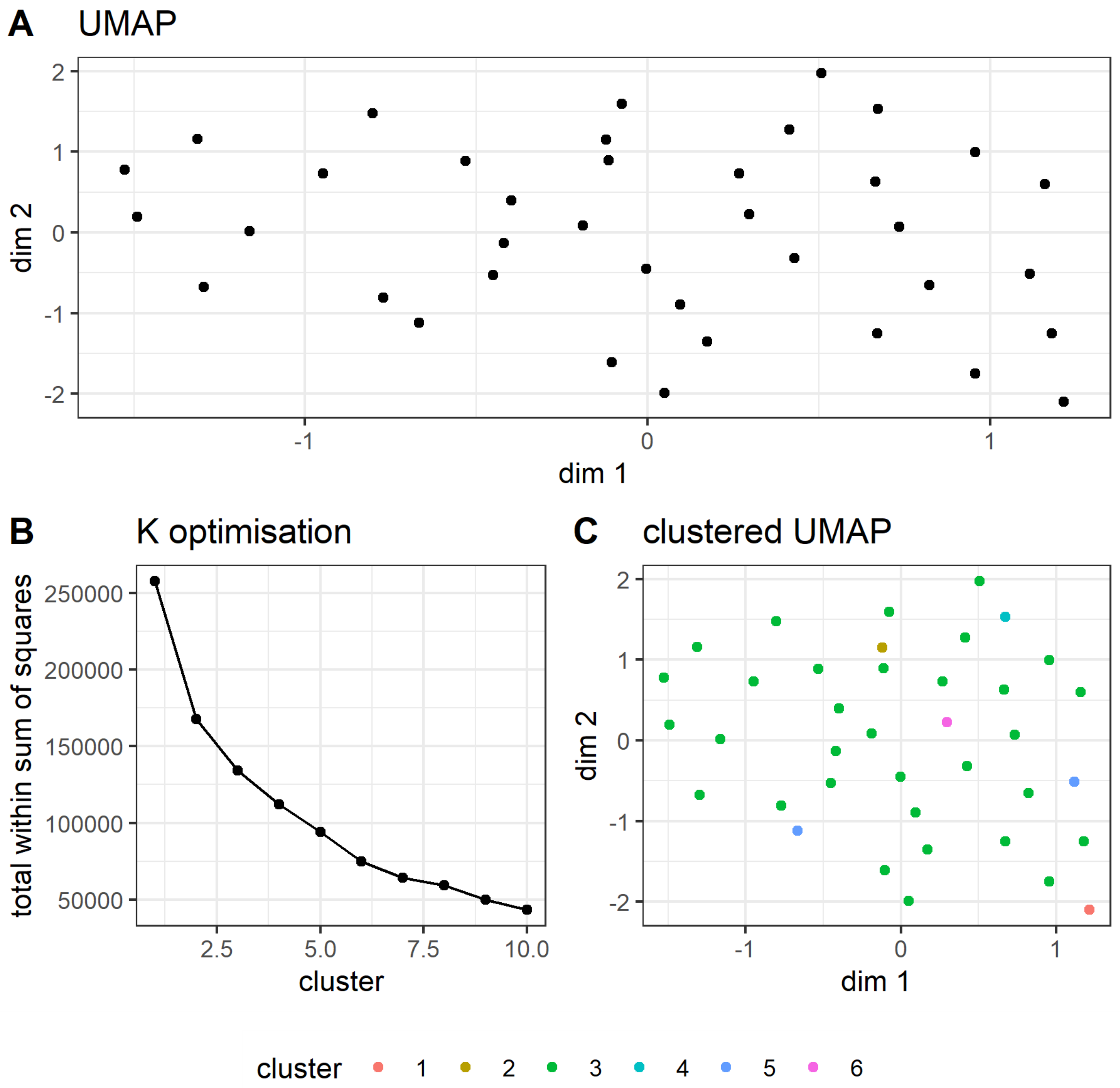

Figure S22. Copy number change matrix clustering

A *- UMAP dimensional reduction of the copy number change matrix shown in figure 6A. No obvious patterns of patient clustering can be identified.*

B *- Visualisation total within sum of squares calculation for cluster numbers 1 through 10 for k-means clustering. This process should typically identify an "elbow" to select as the optimal number of clusters.*

C *- Re-visualisation of the UMAP dimensional reduction with the purported optimal k-means clusters which demonstrated little to no clustering of patients.*

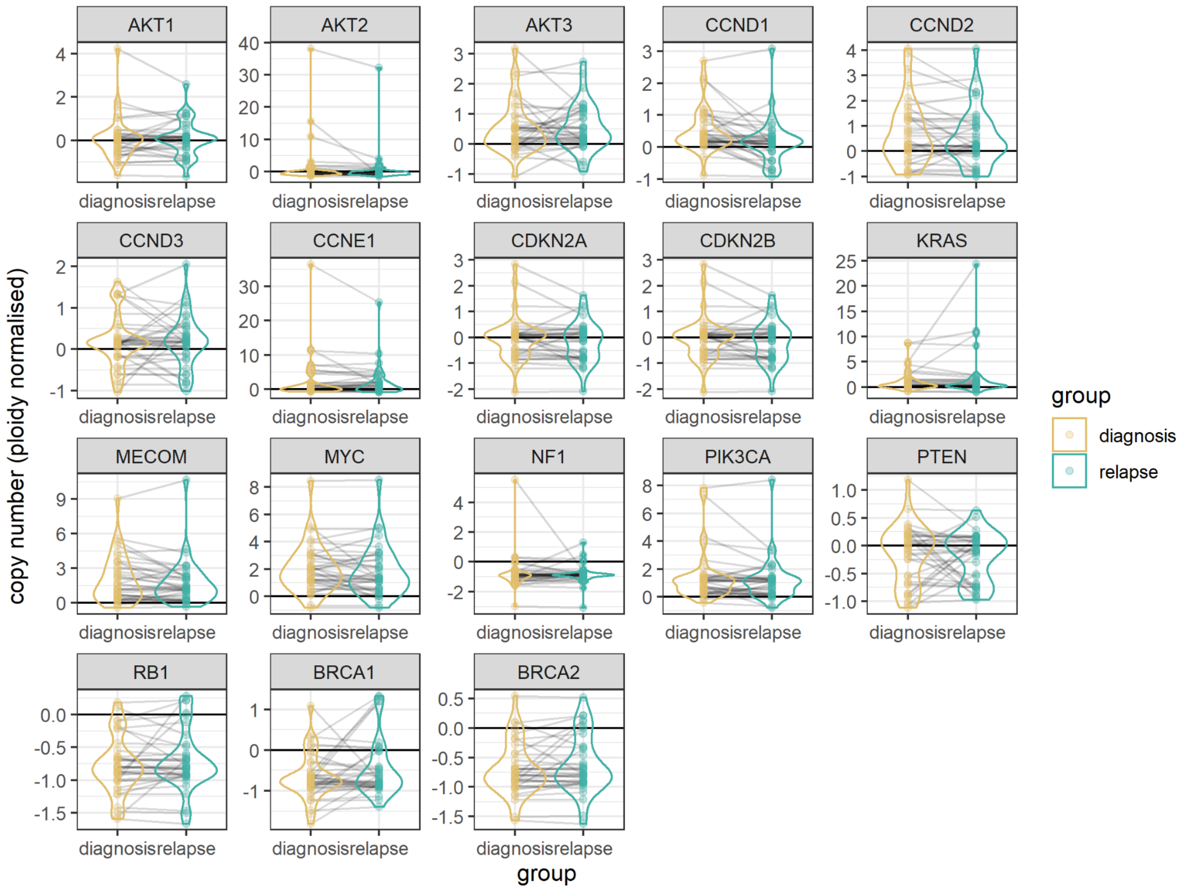

Figure S23. Ploidy normalised copy number change across frequently altered genes

Copy number states of frequently altered / clinically relevant genes after ploidy normalisation (gene copy number - sample group ploidy), stratified by diagnosis and relapse groups. Violin plots visualise the distribution of copy number values between diagnosis and relapse groups, grey lines between points indicate the copy number change and direction of change between the diagnosis and relapse groups for a given patient. Black horizontal line indicates the zero change point where points at this line have gene copy numbers identical to the sample ploidy (n =38 patients).

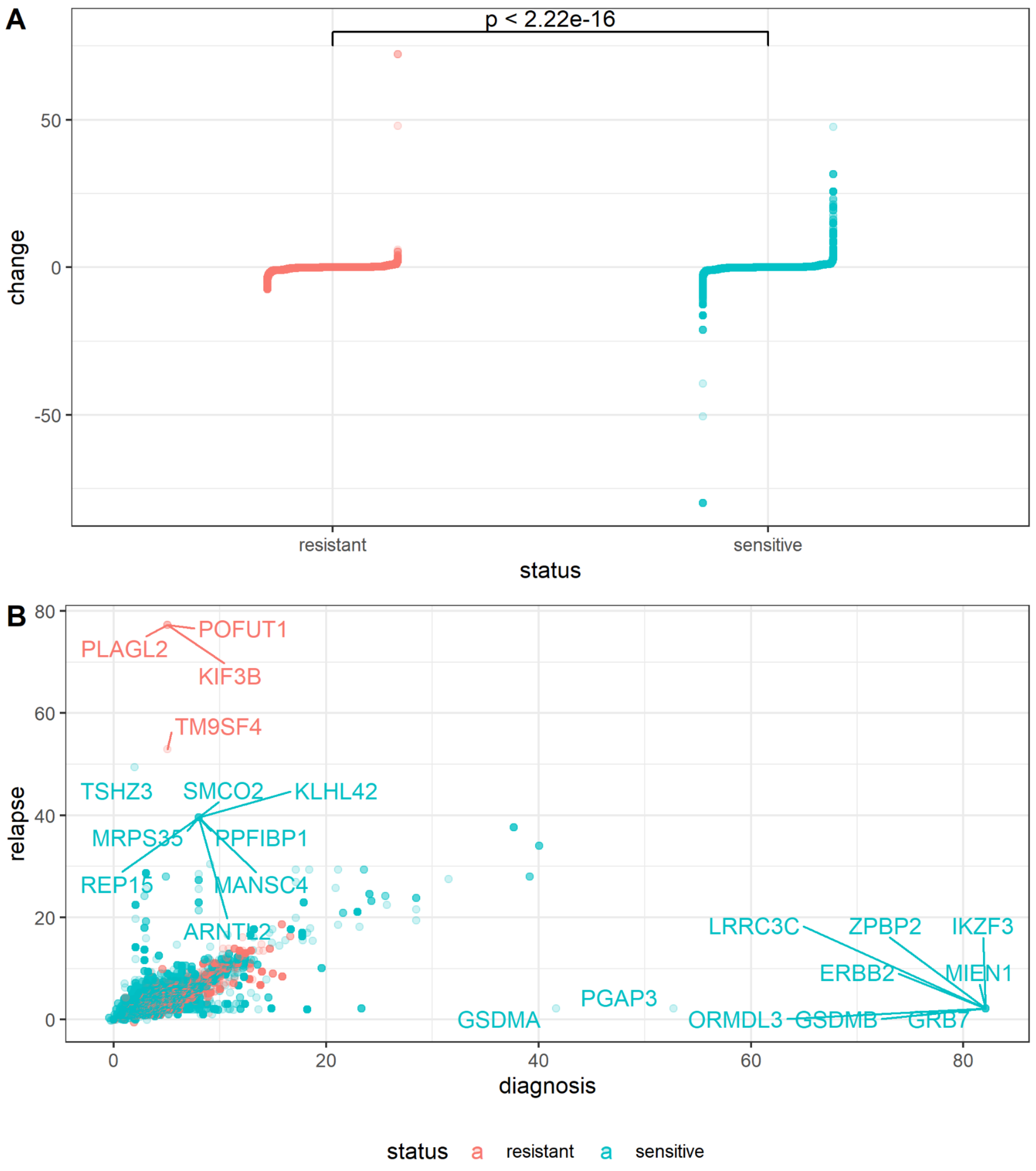

Figure S24. Copy number change by response to platinum-based therapeutics spread

Plots demonstrating the increased number of 'extreme' copy number focal changes between resistant and sensitive HGSC patients (n = 9 & n = 29, resistant and sensitive patients, respectively).

***A** - Jittered point plot of gene change between diagnosis and relapse patients stratified by response to platinum-based chemotherapeutics showing that sensitive patients have a wider distribution, corresponding to a greater number of extreme changes. Statistics shown is a two-sided Mann-Whitney U test (p-value = 2.22e-16).*

***B** - Scatter plot of diagnosis versus relapse values for all gene loci coloured by response to platinum-based chemotherapeutics showing the wider spread of values above and below the diagonal.*

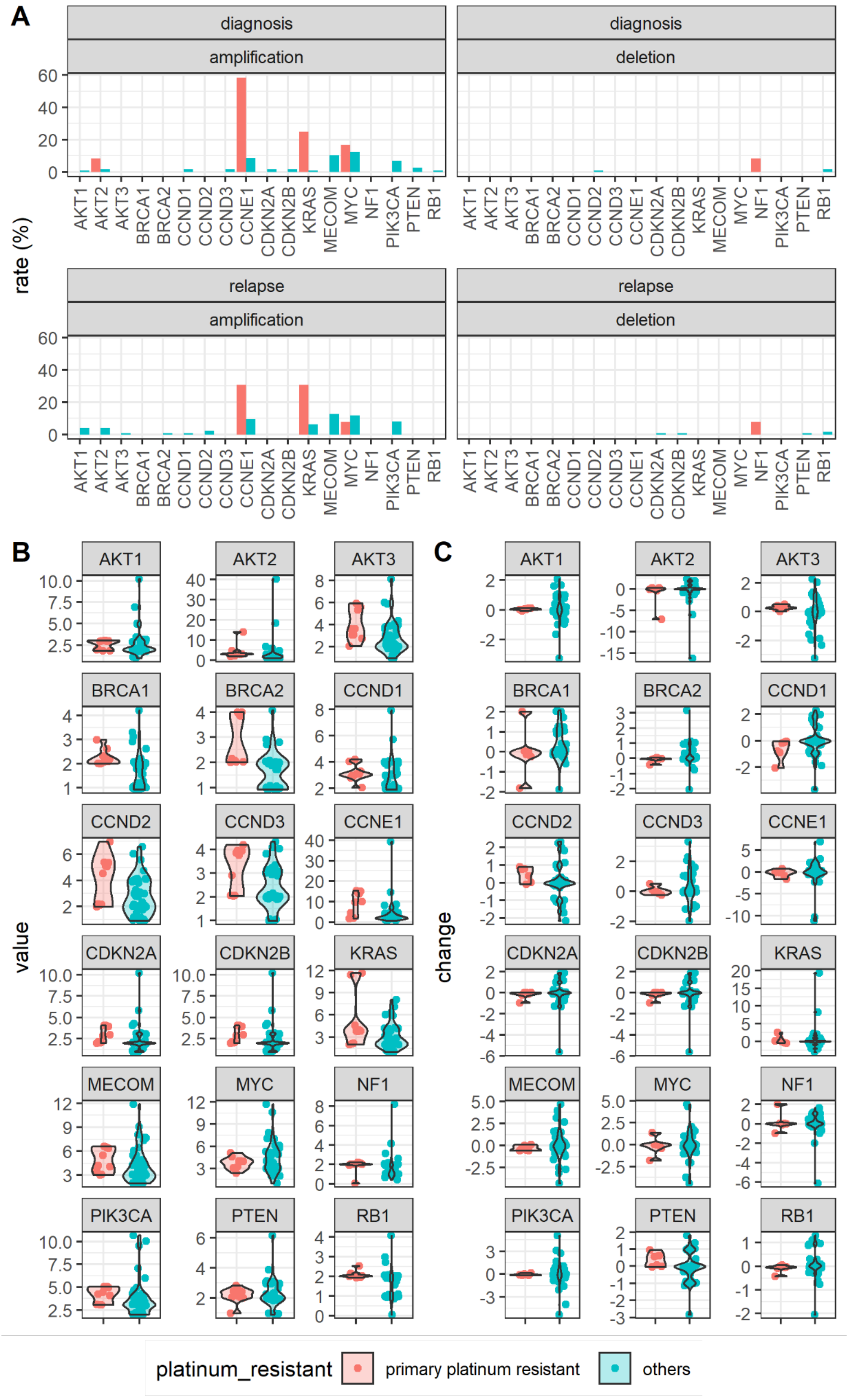

Figure S25. Copy number alteration rates between primary platinum resistant samples

***A** - Computed copy number alteration rate for clinically relevant / frequently altered genes. Faceted bar plot compares the copy number rate between primary platinum resistant samples against all others, stratified by tumour time point and copy number event type (n = 114, n = 12, n = 126 & n = 13; diagnosis-others, diagnosis-primary platinum resistant, relapse-others, relapse-primary platinum resistant, respectively).*

***B** - Copy number state violin distribution for clinically relevant / frequently altered genes comparing primary platinum resistant diagnosis samples versus other diagnosis samples (n = 10 & n = 48, respectively).*

***C** - Copy number violin change distribution for paired samples showing the changing copy number state for clinically relevant / frequently altered genes comparing primary platinum resistant patients versus other patients (n = 6 & n = 41, respectively).*

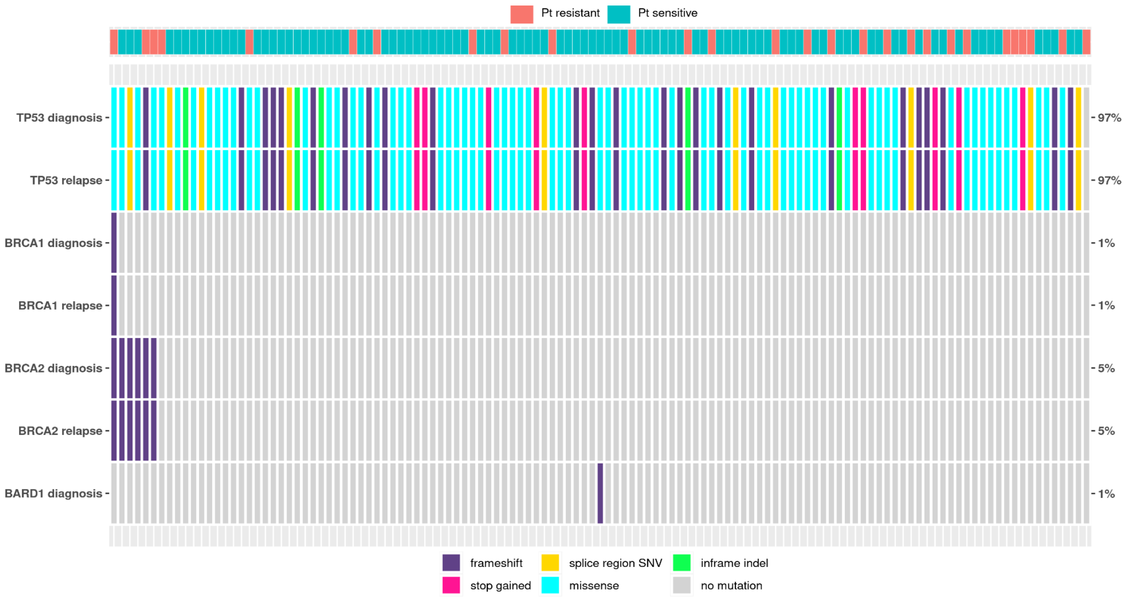

Figure S26. Changes in detected somatic mutations from diagnosis to relapse (matched and paired analysis)
Patients with both diagnosis and relapse tumour samples and also non-tumour samples were examined for the gain or loss of variants from diagnosis to relapse. Inclusion of matched non-tumour samples allowed the confident classification of variants as germline or somatic. TP53 was not matched for the non-tumour sample. Variants with full opacity represent somatic variants whereas variants with reduced opacity represent germline variants. RAD51B, RAD51D, FANCM, BRIP1 and PALB2 were tested but no mutations were identified.

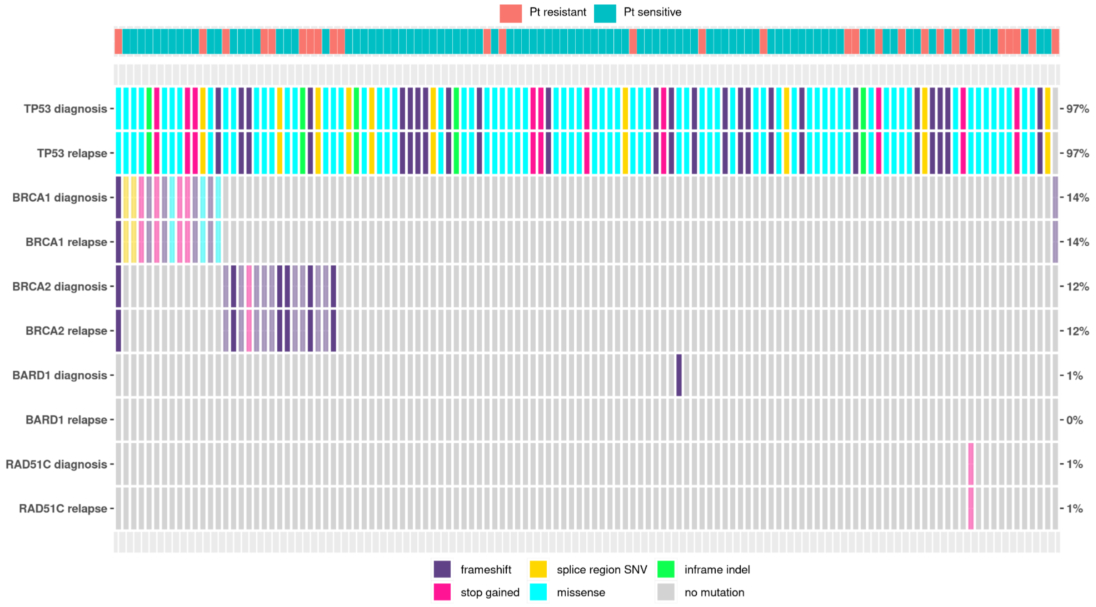

Figure S27. Changes in detected somatic mutations from diagnosis to relapse (matched and paired analysis)
As in supplementary Methods Figure 1 with somatic and germline mutations displayed for this figure.

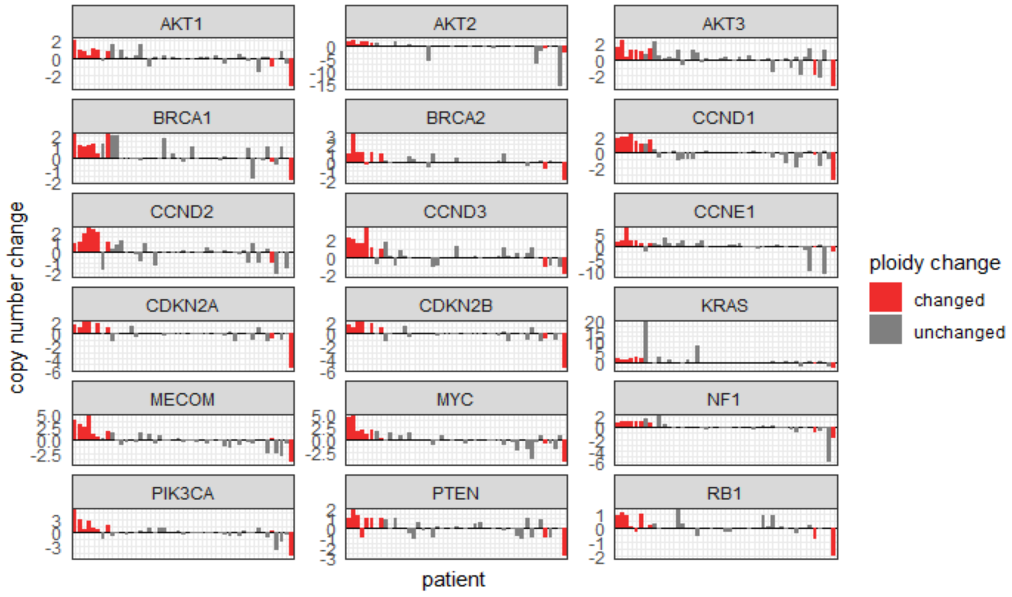

Figure S28. Gene change bar plot

A faceted bar plot showing the copy number changes at frequently altered or clinically relevant genes (n = 47). Plots show the net gain or loss of gene copies between diagnosis and relapse tumours, where a median was taken for time points with multiple samples. A positive value indicates a net gain of gene copies and a negative value a net loss of gene copies, between diagnosis and relapse. Patients with identified ploidy change between diagnosis and relapse are coloured in red.

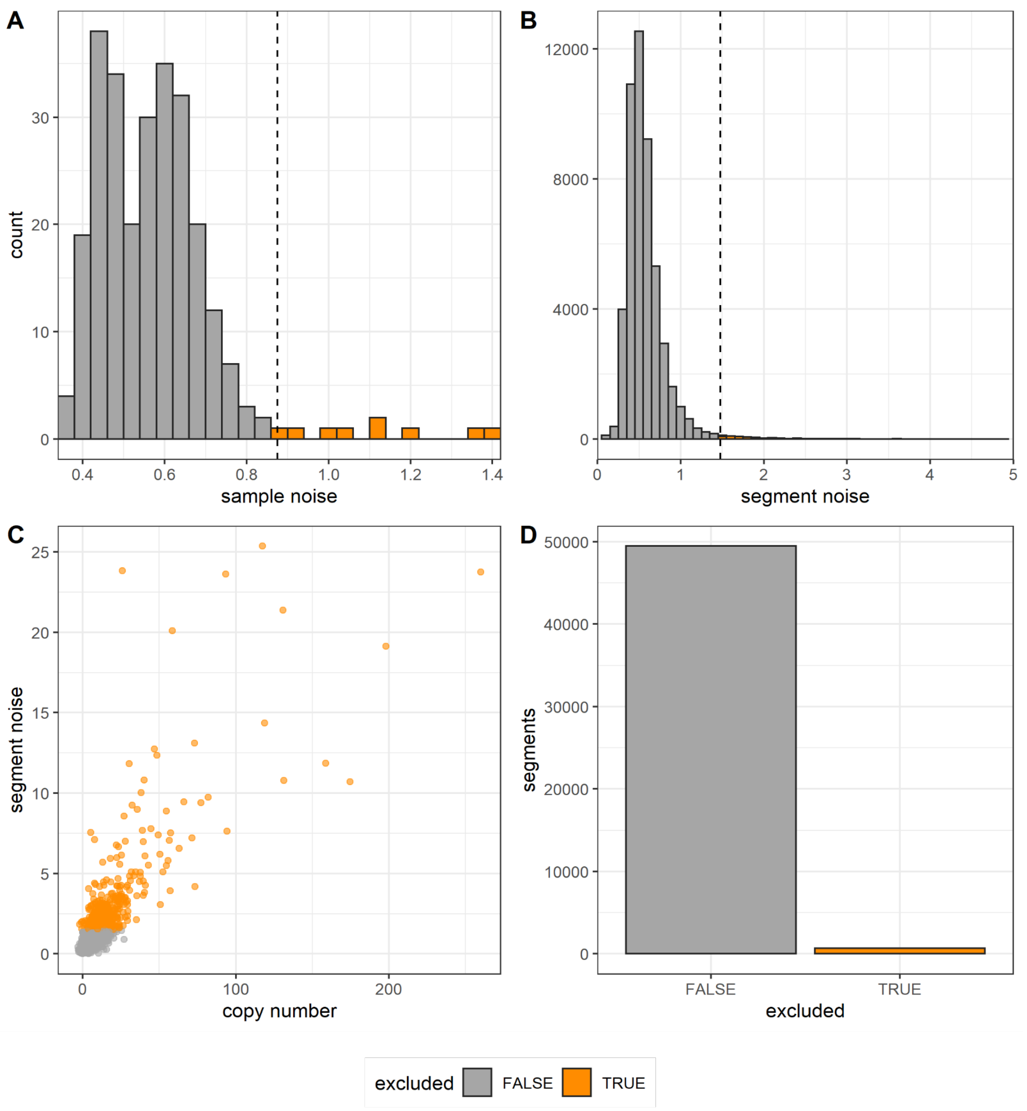

Figure S29. Intra-tumoural heterogeneity filtering
A *- Histogram of mean segment noise across each sample. Vertical dashed line indicates a value of two standard deviations above the mean. Bars highlighted in orange (right of dashed line) were removed from further ITH analysis.* ***B*** *- Histogram of segment noise. Vertical dashed line indicates a value of two standard deviations above the mean. Bars highlighted in orange (right of dashed line) were removed from further ITH analysis.* ***C*** *- Plot visualising the distribution of copy number value against segment noise. Points with orange colouration were those exceeding the segment noise cutoff.* ***D*** *- Bar plot visualising the proportion of total segments that were excluded from ITH by segment noise filtering.*

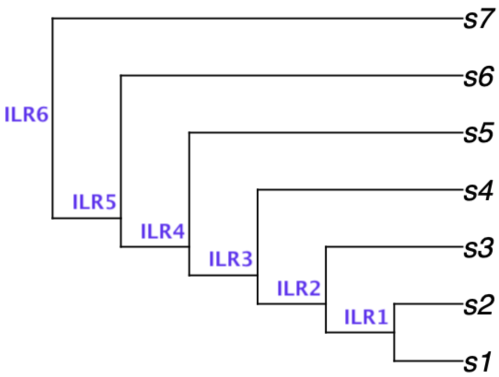

Figure S30. Visualisation of IRL pairwise comparisons within partial ILR model

Tree structure visualisation represents the pairwise comparisons of ratios between various subgroupings of transformed signatures. For example ILR1 is the ratio of s1 to s2, ILR4 is the ratio of the geometric mean of s1-s4 to s5.

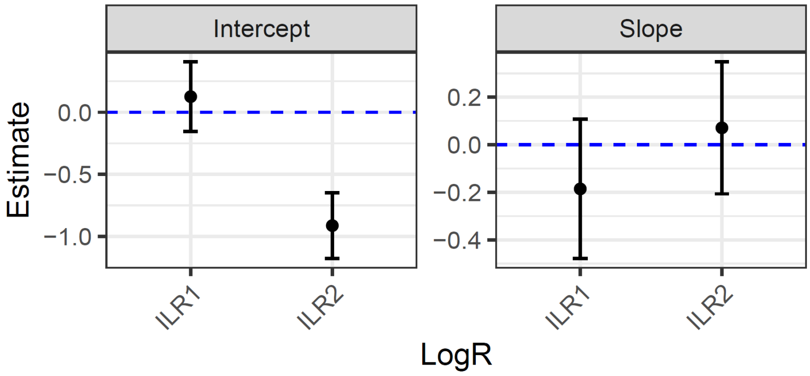

Figure S31. Example beta slope and beta intercept plot

Plot demonstrates an example outcome from a set of 3 signatures modelled using the described partial ILR transformation for two groups of simulated signatures (n = 50 & 50, group 1 and group 2,respectively). Left plot is the beta intercept for ILR1 and ILR2 which are the transformed ratios of signatures s1-s3. Right plot is the beta slope for ILR1 and ILR2 which are the transformed ratios of signatures s1-s3. Error bars represent mean ± the standard error of the mean (SEM).

Tables

Table S1. FIGO stage at time of diagnosis.

*Original FIGO stage**	***Number***	***%***
1a	*3*	*1.1*
1c	*13*	*4.7*
Stage I	***16***	***5.8***
2a	*8*	*2.9*
2b	*8*	*2.9*
2c	*7*	*2.5*
Stage II	***23***	***8.3***
3a	*8*	*2.9*
3b	*16*	*5.8*
3c	*148*	*53.6*
3NOS	*1*	*0.4*
Stage III	***173***	***62.7***
4	*58*	*21.0*
4b	*2*	*0.7*
Stage IV	***60***	***21.7***
NK	*4*	*1.4*
TOTAL	***276***	***100.0***

**As reported by recruiting site according to classification in place at time of original diagnosis (2008 or 2014)*

Table S2. Surgery undertaken during first-line treatment and extent of residual disease following first line surgery.

SURG_TYP_1	***Number***		***Residual disease at first surgery***	***Number***
Interval	*96*		*No residual - R0*	*42*
No surgery	*24*		*Optimal - R1*	*156*
Primary surgery	*152*		*Suboptimal - R2*	*38*
Salvage surgery	*3*		*Not known*	*15*
Missing	*1*		*No surgery*	*24*
Total	***276***		*Missing*	*1*
			Total	***276***

Table S3. BriTROC-1 biopsy locations by sample

Biopsy site summary	***Tissue samples***	***DNA Samples***
Peritoneum	*56*	*62*
*Lymph node**	*73*	*85*
Omentum	*28*	*33*
Colon, mesentery, small bowel, pericolic fat	*20*	*24*
Liver	*17*	*20*
Subcutaneous	*9*	*10*
Gynae organs (uterus, ovary, fallopian tube, vaginal vault)	*16*	*22*
Peri-splenic	*3*	*3*
Brain	*4*	*5*
Pelvis	*2*	*3*
Diaphragm	*2*	*2*
Chest (lung, mediastinum, trachea)	*3*	*3*
Breast	*3*	*4*
Bladder	*3*	*4*
Perinephric fat	*1*	*1*
Multiple	*8*	*11*
Total	***247***	***292***

Table S4. BriTROC-1 lymph node locations by sample

****Lymph node summary***	***Number***
PELVIC	*14*
RETROPERITONEAL	*23*
AXILLARY	*14*
CERVICAL	*1*
INGUINAL	*12*
MEDIASTINAL	*1*
RIGHT ILIAC FOSSA	*1*
SUPRA-CLAVICULAR	*7*
Total	***73***

Table S5. Response to first treatment following study entry as reported by recruiting site

Response (N)	***Overall***	***Sensitive***	***Resistant***
Complete Response	*36*	*35*	*1*
Partial Response	*78*	*67*	*11*
Stable Disease	*50*	*36*	*14*
Progressive Disease	*63*	*35*	*28*
Not Evaluable	*7*	*5*	*2*
Not Known	*32*	*27*	*5*
No treatment	*10*	*4*	*6*
Total	***276***	***209***	***67***
Response rate (N)	*114*	*102*	*12*
Response Rate (CR+PR) (%)	*48.7*	*57.3*	*21.4*

Table S6. List of clinically relevant and/or frequently altered genes in HGSC

Gene name	*Chromosome*	*Gene start (bp)*	*Gene end (bp)*	*Cyto*	*expected*	*ensembl*
AKT1	*14*	*105235686*	*105262088*	*14q32.33*	*AMP*	*ENSG00000142208*
AKT2	*19*	*40736224*	*40791443*	*19q13.2*	*AMP*	*ENSG00000105221*
AKT3	*1*	*243651535*	*244014381*	*1q44*	*AMP*	*ENSG00000117020*
CCND1	*11*	*69455855*	*69469242*	*11q13.3*	*AMP*	*ENSG00000110092*
CCND2	*12*	*4382938*	*4414516*	*12p13.32*	*AMP*	*ENSG00000118971*
CCND3	*6*	*41902671*	*42018095*	*6p21.1*	*AMP*	*ENSG00000112576*
CCNE1	*19*	*30302805*	*30315215*	*19q12*	*AMP*	*ENSG00000105173*
CDKN2A	*9*	*21967751*	*21995300*	*9p21.3*	*DEL*	*ENSG00000147889*
CDKN2B	*9*	*22002902*	*22009362*	*9p21.3*	*DEL*	*ENSG00000147883*
KRAS	*12*	*25357723*	*25403870*	*12p12.1*	*AMP*	*ENSG00000133703*
MECOM	*3*	*168801287*	*169381406*	*3q26.2*	*AMP*	*ENSG00000085276*
MYC	*8*	*128747680*	*128753674*	*8q24.21*	*AMP*	*ENSG00000136997*
NF1	*17*	*29421945*	*29709134*	*17q11.2*	*DEL*	*ENSG00000196712*
PIK3CA	*3*	*178865902*	*178957881*	*3q26.32*	*AMP*	*ENSG00000121879*
PTEN	*10*	*89622870*	*89731687*	*10q23.31*	*DEL*	*ENSG00000171862*
RB1	*13*	*48877887*	*49056122*	*13q14.2*	*DEL*	*ENSG00000139687*

Table S7. Ploidy change scoring

patient	***samples***	***category 1***	***category 2***	***category 3***	***star rating***
BRITROC-209	*IM_295, JBLAB-4960*	*TRUE*	*FALSE*	*TRUE*	**
BRITROC-216	*IM_336, IM_337, IM_338, IM_339, IM_340, IM_341, IM_342, JBLAB-4965*	*TRUE*	*FALSE*	*FALSE*	*
BRITROC-23	*IM_56, JBLAB-4128, JBLAB-4967*	*TRUE*	*FALSE*	*TRUE*	**
BRITROC-241	*IM_423,JBLAB-4996*	*TRUE*	*TRUE*	*FALSE*	**
BRITROC-248	*IM_403, JBLAB-19302, JBLAB-19303*	*TRUE*	*FALSE*	*TRUE*	**
BRITROC-267	*IM_383, JBLAB-19330*	*TRUE*	*TRUE*	*FALSE*	**
BRITROC-274	*IM_395, IM_396, IM_397, JBLAB-19337, JBLAB-19338*	*TRUE*	*FALSE*	*FALSE*	*
BRITROC-67	*IM_115, JBLAB-4179*	*TRUE*	*TRUE*	*FALSE*	**
BRITROC-74	*IM_124, JBLAB-4186, JBLAB-4187, JBLAB-4188, JBLAB-4189*	*TRUE*	*TRUE*	*TRUE*	***

NB - Details of the ploidy change rating and designation can be found in the supplemental methods

Table S8. Sample stratification per analysis composition table.

**CNA analysis (focal, broad & signatures)**		no stratification	platinum resistant	platinum sensitive	primary platinum resistant	primary platinum sensitive	prior lines (1,2,3,4+)
Unpaired	diagnosis	126	30	96	12	114	89,29,4,4
	relapse	139	36	103	13	126	87,38,9,5
paired	diagnosis	58	15	43	10	48	42,14,1,1
	relapse	68	15	53	10	58	47,19,1,1
Patient (paired)		47	10	37	6	41	34,11,1,1
**Intra-tumour heterogeneity (ITH) analysis**							
Unpaired	diagnosis	119	29	90	11	108	84,27,4,4
	relapse	137	35	102	13	124	86,37,9,5
paired	diagnosis	57	15	42	10	47	41,14,1,1
	relapse	68	15	53	10	58	47,19,1,1
**Immune analysis**							
Unpaired	diagnosis	92	15	77	7	85	68,19,3,2
	relapse	0	0	0	0	0	0
paired	diagnosis	47	9	38	6	41	34,11,1,1
	relapse	0	0	0	0	0	0

Supplementary References

1. McKenna A*, et al.* The Genome Analysis Toolkit: a MapReduce framework for analyzing next-generation DNA sequencing data. *Genome research* **20**, 1297-1303 (2010).

2. Poplin R*, et al.* A universal SNP and small-indel variant caller using deep neural networks. *Nature biotechnology* **36**, 983-987 (2018).

3. Cooke DP, Wedge DC, Lunter G. A unified haplotype-based method for accurate and comprehensive variant calling. *Nature biotechnology* **39**, 885-892 (2021).

4. Macintyre G*, et al.* Copy-number signatures and mutational processes in ovarian carcinoma. *Nat Genet* **50**, 1262-1270 (2018).

5. Goranova T*, et al.* Safety and utility of image-guided research biopsies in relapsed high-grade serous ovarian carcinoma-experience of the BriTROC consortium. *Br J Cancer* **116**, 1294-1301 (2017).

6. Piskorz AM*, et al.* Methanol-based fixation is superior to buffered formalin for next-generation sequencing of DNA from clinical cancer samples. *Ann Oncol* **27**, 532-539 (2016).

7. Ahmed AA*, et al.* Driver mutations in TP53 are ubiquitous in high grade serous carcinoma of the ovary. *J Pathol* **221**, 49-56 (2010).

8. Williams C*, et al.* A high frequency of sequence alterations is due to formalin fixation of archival specimens. *Am J Pathol* **155**, 1467-1471 (1999).

9. Wong SQ*, et al.* Sequence artefacts in a prospective series of formalin-fixed tumours tested for mutations in hotspot regions by massively parallel sequencing. *BMC medical genomics* **7**, 23 (2014).

10. McLaren W*, et al.* The Ensembl Variant Effect Predictor. *Genome biology* **17**, 122 (2016).

11. Tamborero D*, et al.* The Molecular Tumor Board Portal supports clinical decisions and automated reporting for precision oncology. *Nat Cancer* **3**, 251-261 (2022).

12. Schröder J, Corbin V, Papenfuss AT. HYSYS: have you swapped your samples? *Bioinformatics (Oxford, England)* **33**, 596-598 (2017).

13. Van Loo P*, et al.* Allele-specific copy number analysis of tumors. *Proc Natl Acad Sci U S A* **107**, 16910-16915 (2010).

14. van Dijk E*, et al.* Chromosomal copy number heterogeneity predicts survival rates across cancers. *Nature Communications* **12**, 3188 (2021).

15. Aitchison J. The Statistical Analysis of Compositional Data. *Journal of the Royal Statistical Society Series B (Methodological)* **44**, 139-177 (1982).

16. Cheng Z*, et al.* The Genomic Landscape of Early-Stage Ovarian High-Grade Serous Carcinoma. *Clin Cancer Res* **28**, 2911-2922 (2022).

17. Egozcue JJ, Pawlowsky-Glahn V, Mateu-Figueras G, Barceló-Vidal C. Isometric Logratio Transformations for Compositional Data Analysis. *Mathematical Geology* **35**, 279-300 (2003).

18. Kristensen K, Nielsen A, Berg CW, Skaug H, Bell BM. TMB: Automatic Differentiation and Laplace Approximation. *Journal of Statistical Software* **70**, 1 - 21 (2016).
