# Supplementary material for: The copy number and mutational landscape of recurrent ovarian high-grade serous carcinoma
Source: Nat Commun. 2023 Jul 20;14:4387. doi: 10.1038/s41467-023-39867-7 (PMC10359414; doi:10.1038/s41467-023-39867-7)
Supplement: Supplementary file 3 — Description of Additional Supplementary Files [file 41467_2023_39867_MOESM3_ESM.docx]

Inventory of Supplementary Information

### Supplementary methods

- 1. Patient inclusion and exclusion criteria
     1. Patient & sample subsetting
  2. Tagged Amplicon Sequencing
     1. Read alignment
     2. Read alignment post-processing and QC
  3. Germline Variant Calling
     1. HaplotypeCaller/ampliconseq
     2. Octopus
     3. Germline variant post-processing
  4. Tumour Sample Variant Calling
     1. *TP53* somatic variant calling
     2. Non-*TP53* somatic variant calling
     3. Unmatched and unpaired analyses
     4. Unmatched and paired analyses
     5. Matched and unpaired analyses
     6. Matched and paired analyses
  5. Fixation artefact correction
  6. Variant annotation
  7. Inference of sample mislabelling events
  8. Copy number fitting
     1. Profile fitting
  9. Copy number analysis
     1. Copy number event calling
     2. Ploidy changes
     3. Purity differences
  10. Intra-tumour heterogeneity
  11. Copy number signature abundance modelling
      1. Model interpretability
  12. Immune environment from copy number signatures
      1. Sample preparation
      2. Automated Staining
      3. Image analyses
      4. Quantification of tumour immunohistochemistry markers

### Supplementary figures and tables

- 1. Figure S1. BriTROC study REMARK diagrams
  2. Figure S2. Germline SNVs and short indels identified in key homologous recombination pathway genes
  3. Figure S3. Whole cohort-level detection of SNVs and short indels in key cancer related genes (unpaired)
  4. Figure S4. Genome-wide copy number alteration frequency plot
  5. Figure S5: Purity distributions for diagnosis and relapse tumours
  6. Figure S6. Ploidy distributions for diagnosis and relapse tumours
  7. Figure S7. Segment distributions for primary and relapse tumours
  8. Figure S8. Copy number events
  9. Figure S9. Copy number features
  10. Figure S10: Cytoband and chromosome arm alteration rates
  11. Figure S11. Total gene copies
  12. Figure S12. Copy number focal changes in frequently altered genes stratified by prior lines of therapy
  13. Figure S13. Stratified copy number count by prior lines and tumour timepoint
  14. Figure S14. Stratified copy number count by platinum status and tumour timepoint
  15. Figure S15. Intra-tumoural heterogeneity
  16. Figure S16. Intra-tumoural heterogeneity change
  17. Figure S17. Copy number signature correlations
  18. Figure S18. Copy number features by tissue site of origin
  19. Figure S19. Copy number features stratified by diagnosis and relapse across tissue site of origin
  20. Figure S20. Gene change heatmap and correlation matrix
  21. Figure S21. Copy number change correlations in frequently altered genes
  22. Figure S22. Copy number change matrix clustering
  23. Figure S23. Ploidy normalised copy number change across frequently altered genes
  24. Figure S24. Copy number change by response to platinum-based therapeutics
  25. Figure S25. Copy number alteration rates between primary platinum resistant samples
  26. Figure S26. Changes in detected somatic mutations from diagnosis to relapse (matched and paired analysis)
  27. Figure S27. Changes in detected somatic mutations from diagnosis to relapse (matched and paired analysis)
  28. Figure S28. Gene change bar plot
  29. Figure S29. Intra-tumoural heterogeneity filtering
  30. Figure S30. Visualisation of IRL pairwise comparisons within partial ILR model
  31. Figure S31. Example beta slope and beta intercept plot
  32. Table S1. FIGO stage at time of diagnosis.
  33. Table S2. Surgery undertaken during first-line treatment and extent of residual disease following first line surgery.
  34. Table S3. BriTROC-1 biopsy locations by sample
  35. Table S4. BriTROC-1 lymph node locations by sample
  36. Table S5. Response to first treatment following study entry as reported by recruiting site
  37. Table S6. List of clinically relevant and/or frequently altered genes in HGSC
  38. Table S7. Ploidy change scoring
  39. Table S8. Sample stratification per analysis composition table
  40. Supplementary references

### Supplementary data

- 1. Supplementary data 1 BriTROC-1 chemotherapy lines before study entry
  2. Supplementary data 2 BriTROC-1_chemotherapy drugs before study entry
  3. Supplementary data 3 BriTROC-1 short variants
  4. Supplementary data 4 Individual patient vignettes.
  5. Supplementary data 5 BriTROC-1 amplicon data.
