# Supplementary material for: The copy number and mutational landscape of recurrent ovarian high-grade serous carcinoma
Source: Nat Commun. 2023 Jul 20;14:4387. doi: 10.1038/s41467-023-39867-7 (PMC10359414; doi:10.1038/s41467-023-39867-7)

BRITROC−1

age: 81

stage: 33

platinum status: resistant

prior lines: 1

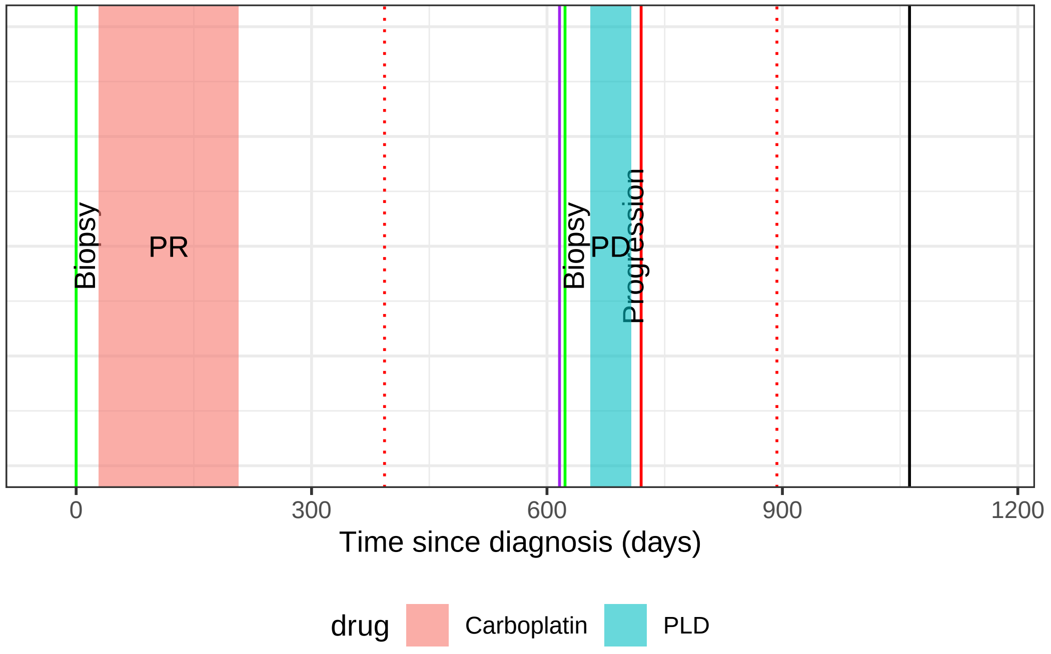

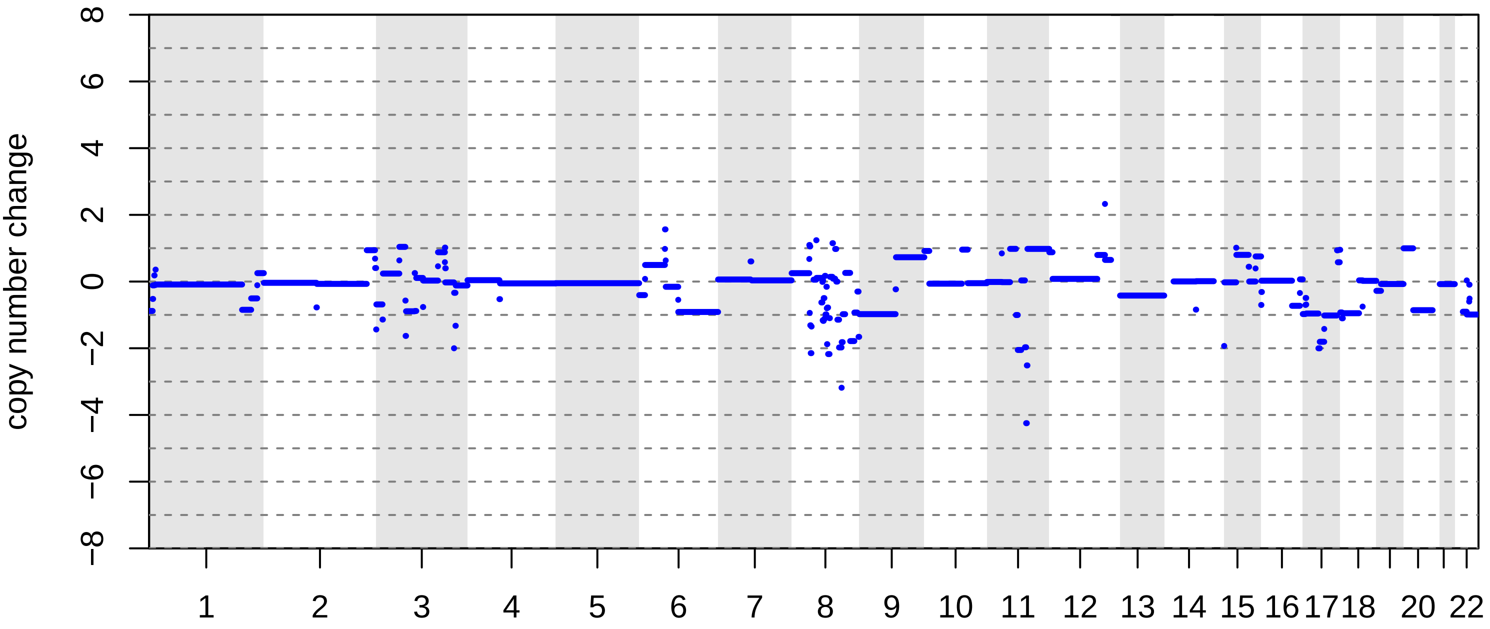

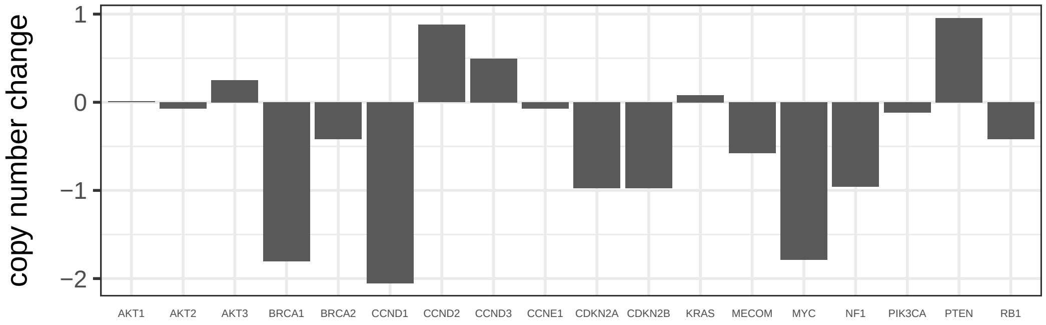

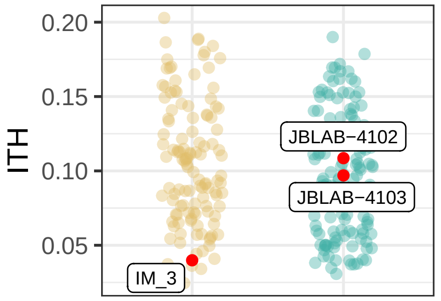

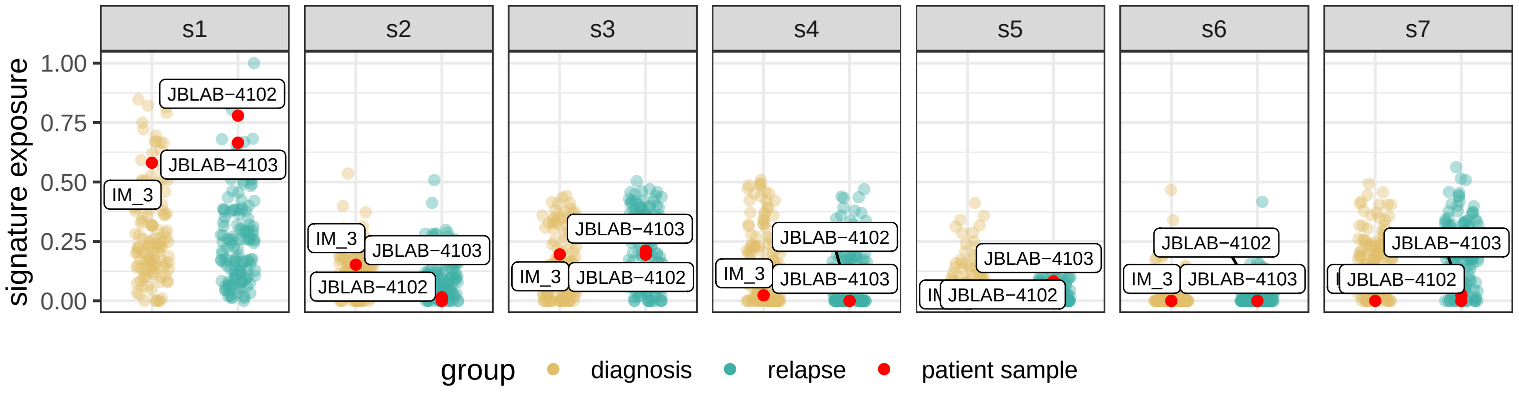

BRITROC−106

age: 66

stage: 33

platinum status: sensitive

prior lines: 2

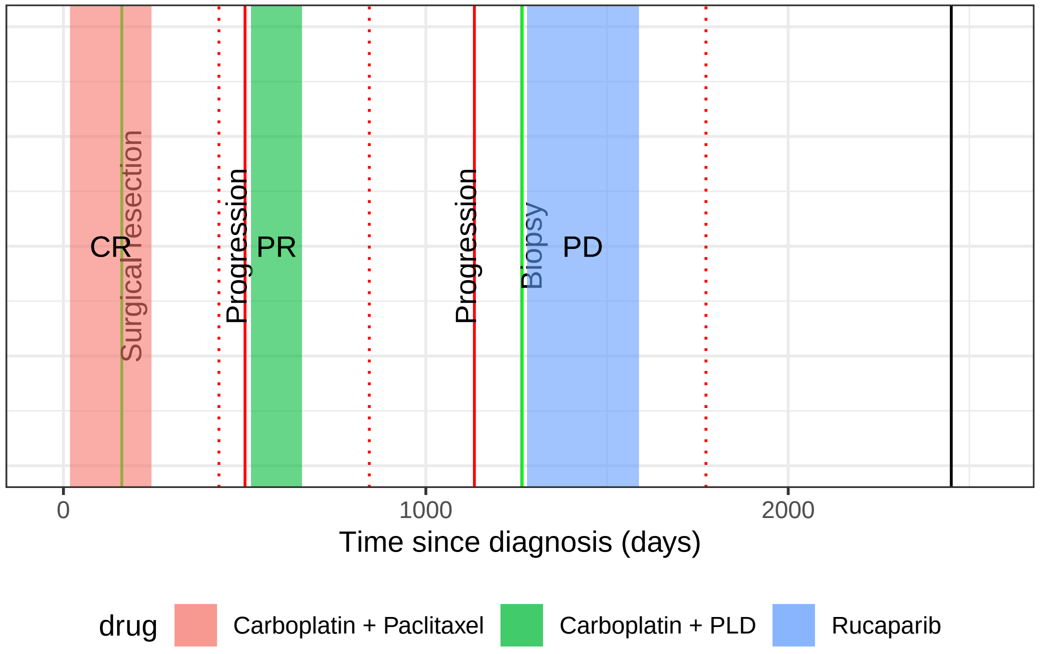

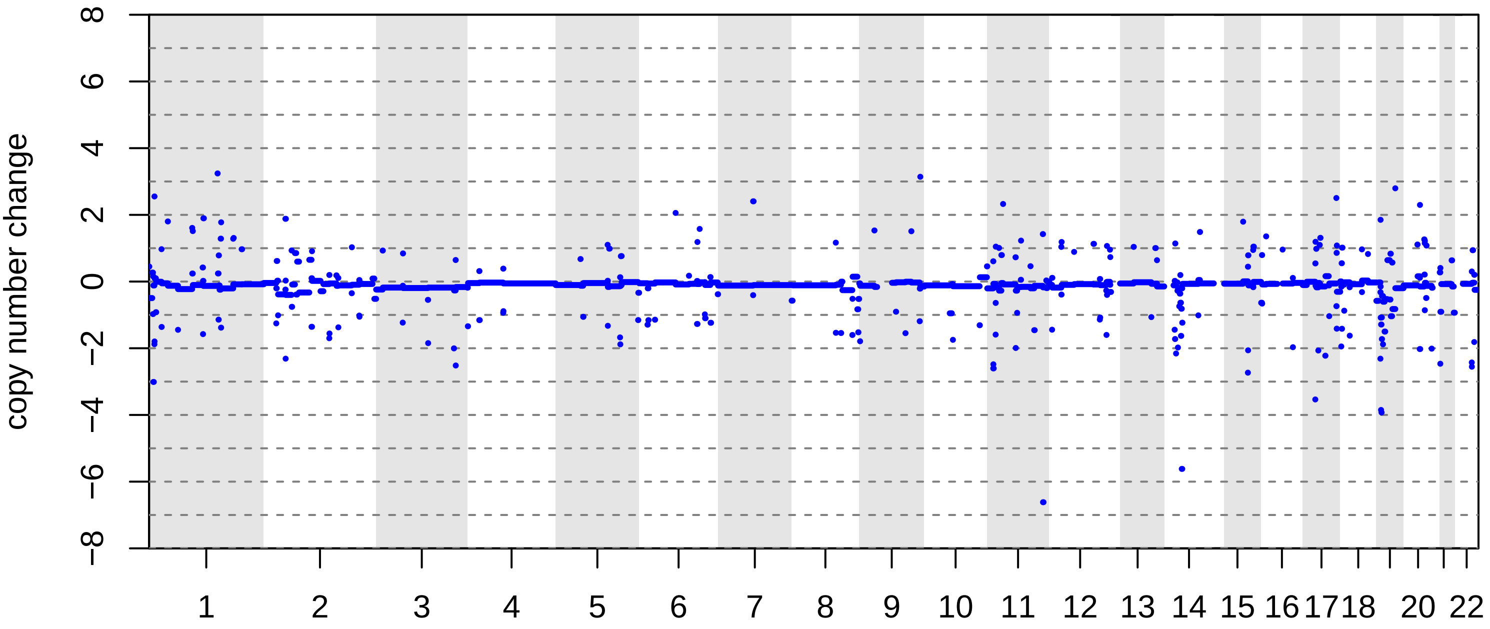

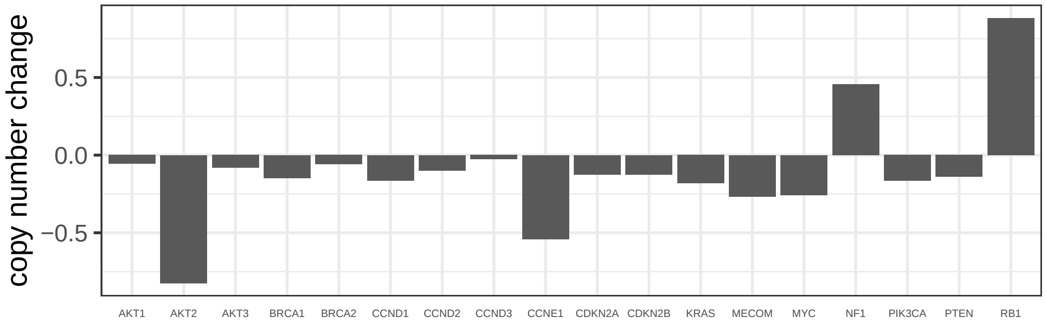

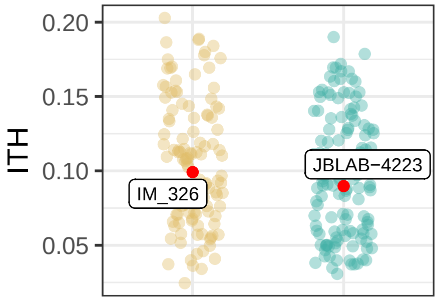

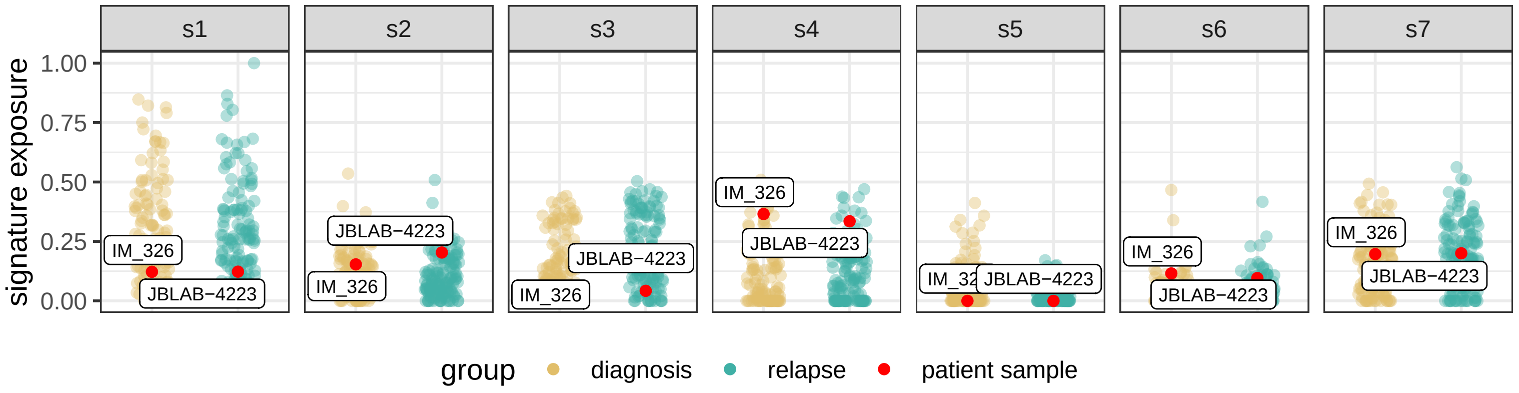

BRITROC−11

age: 74

stage: 33

platinum status: sensitive

prior lines: 1

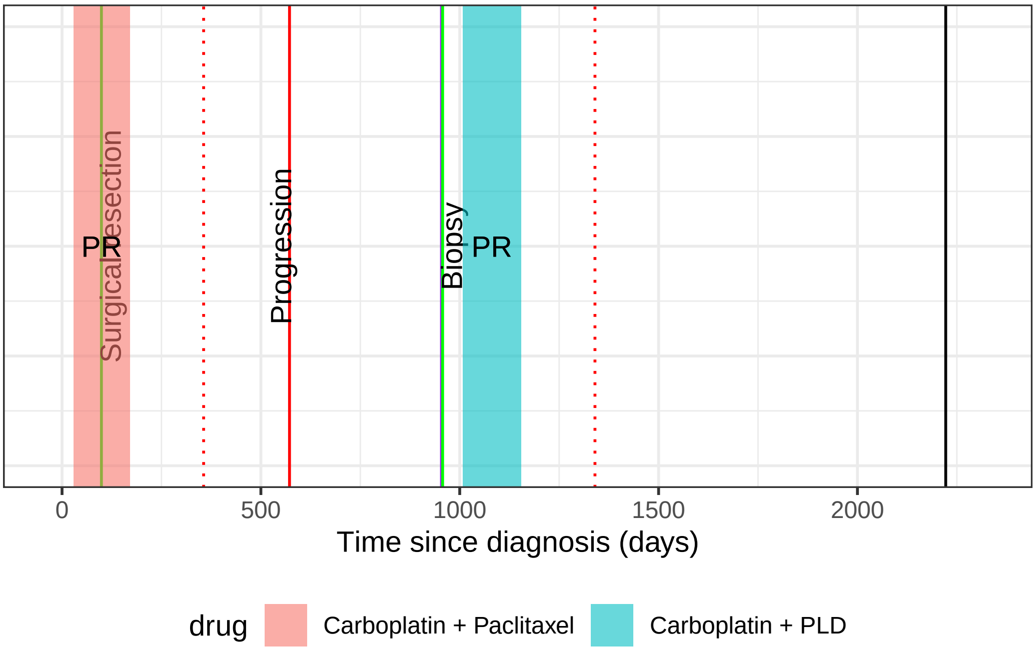

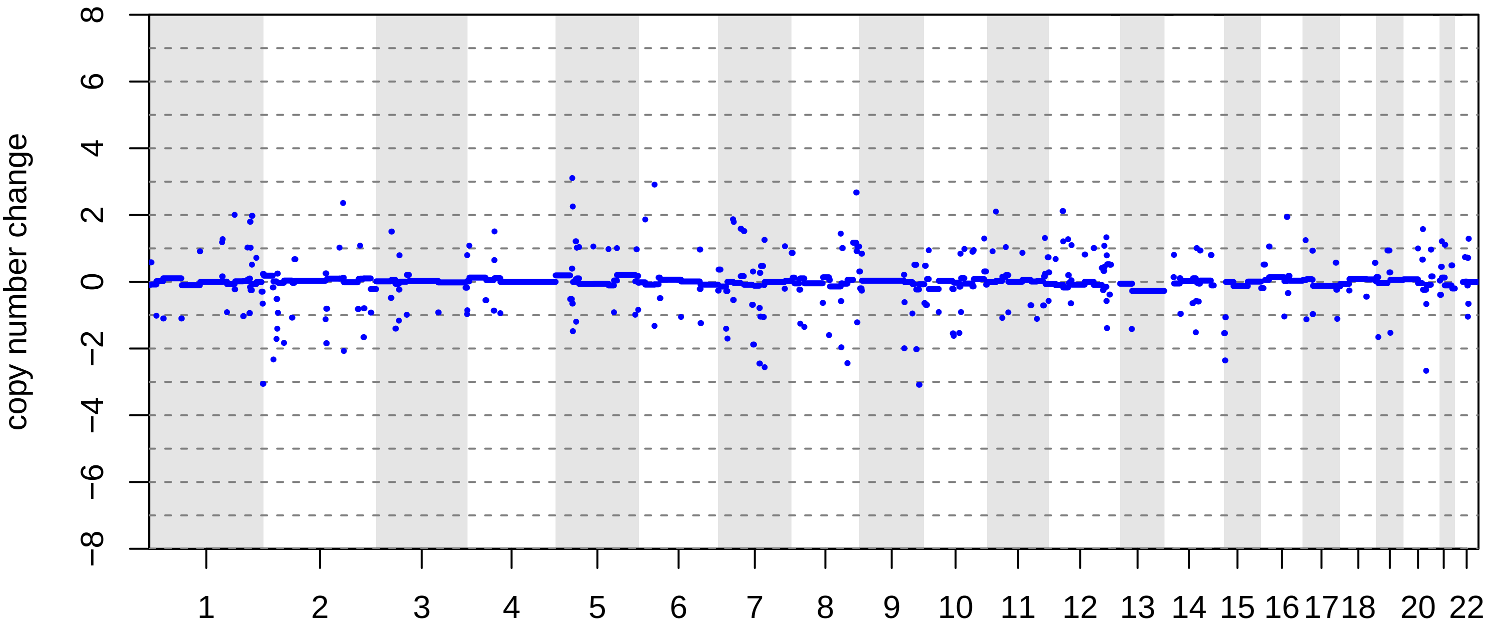

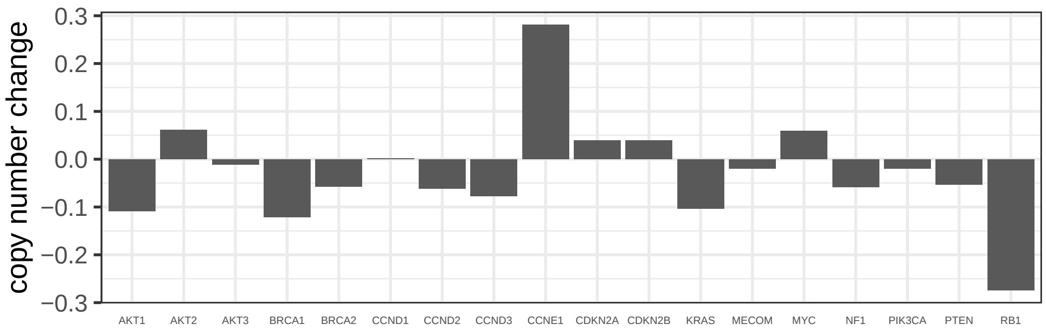

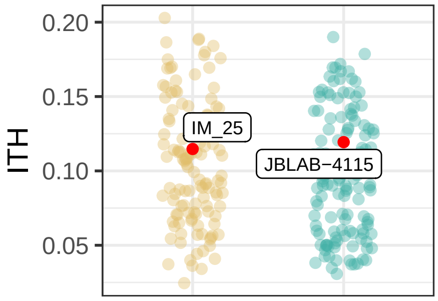

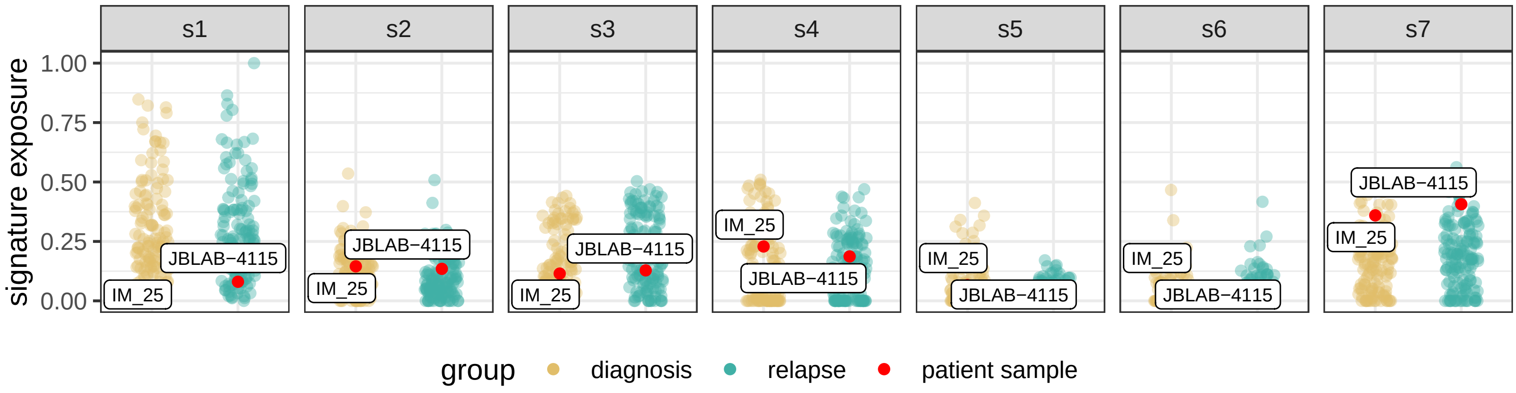

BRITROC−124

age: 63

stage: 33

platinum status: sensitive

prior lines: 1

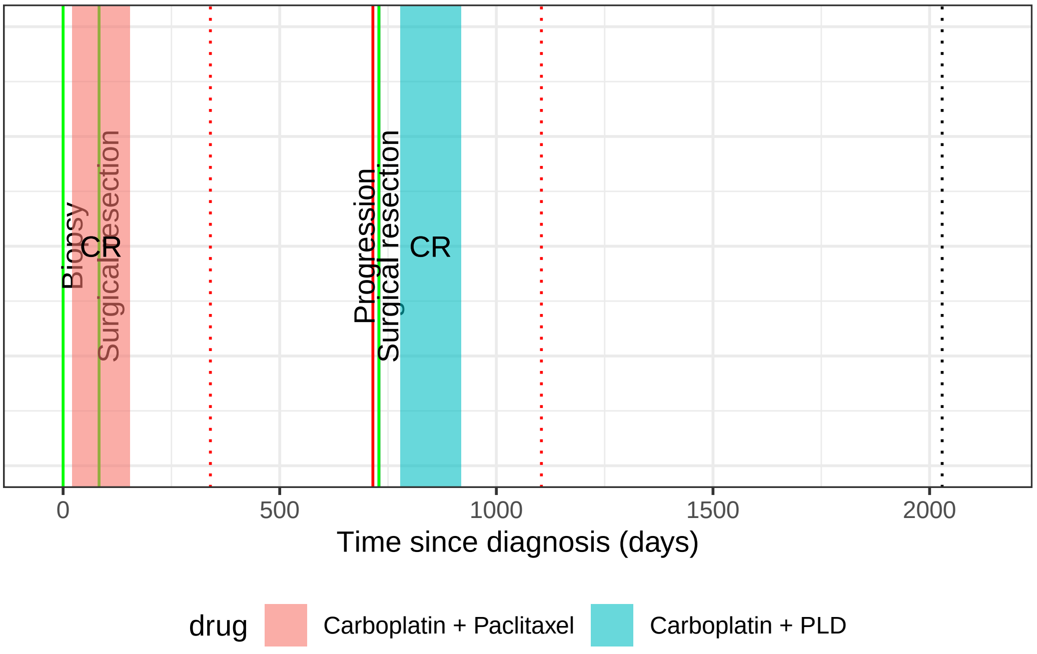

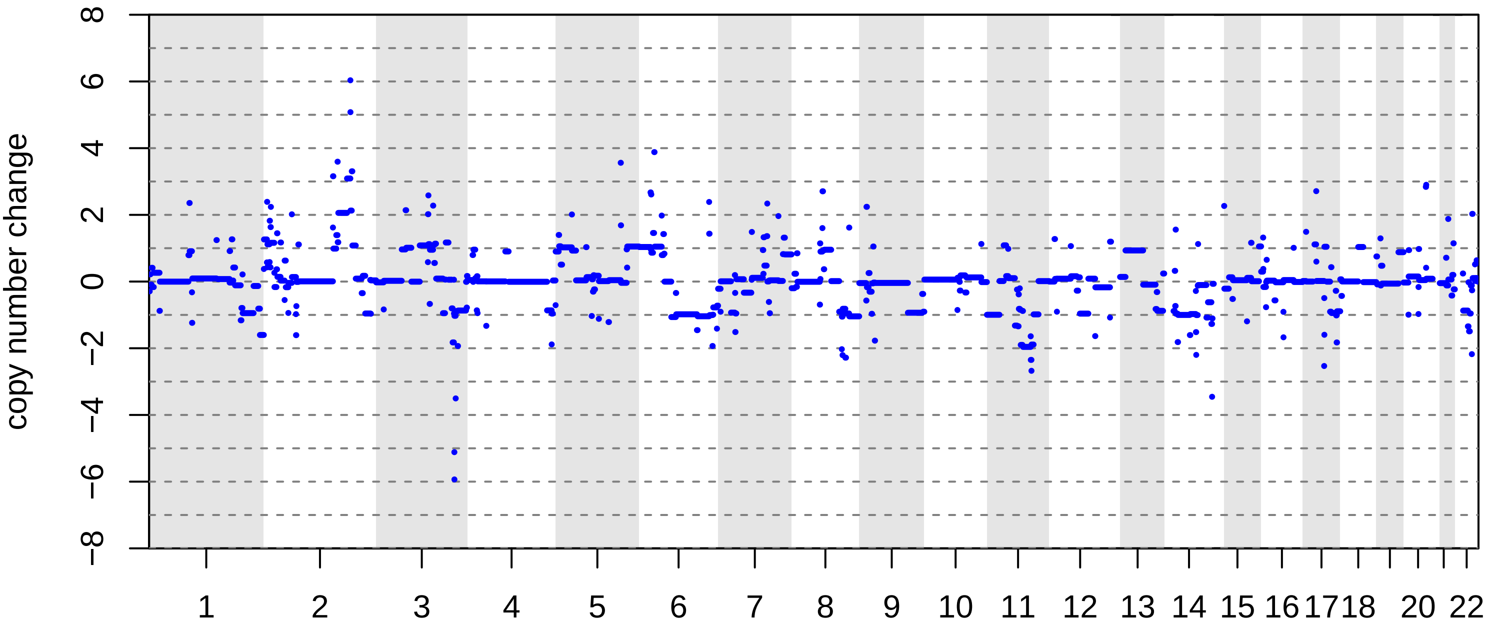

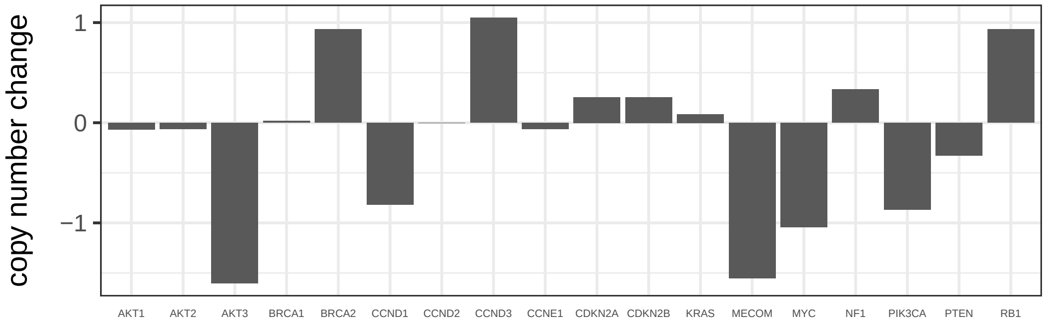

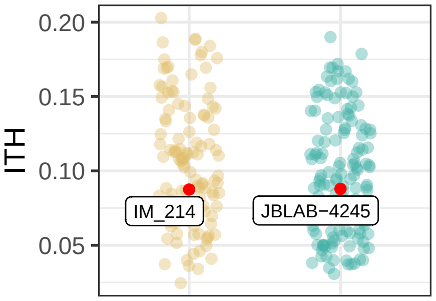

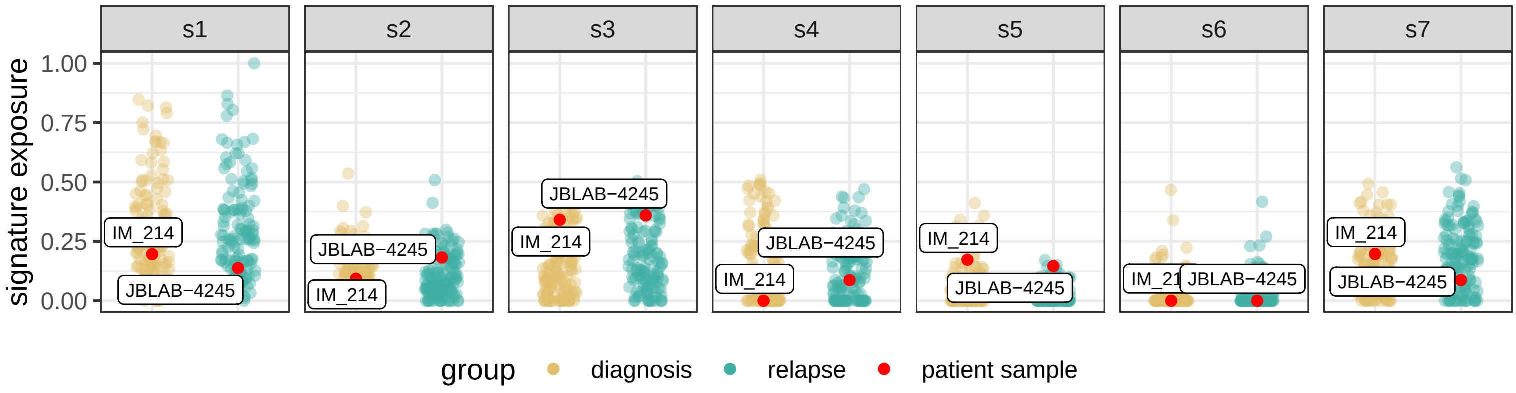

BRITROC−132

age: 68

stage: 32

platinum status: sensitive

prior lines: 1

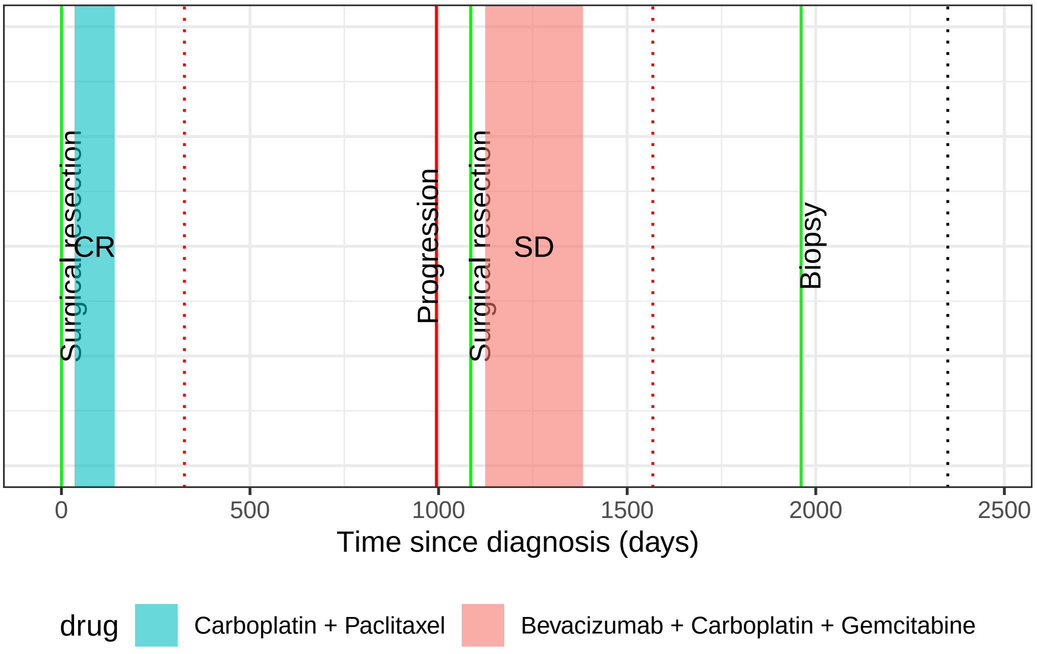

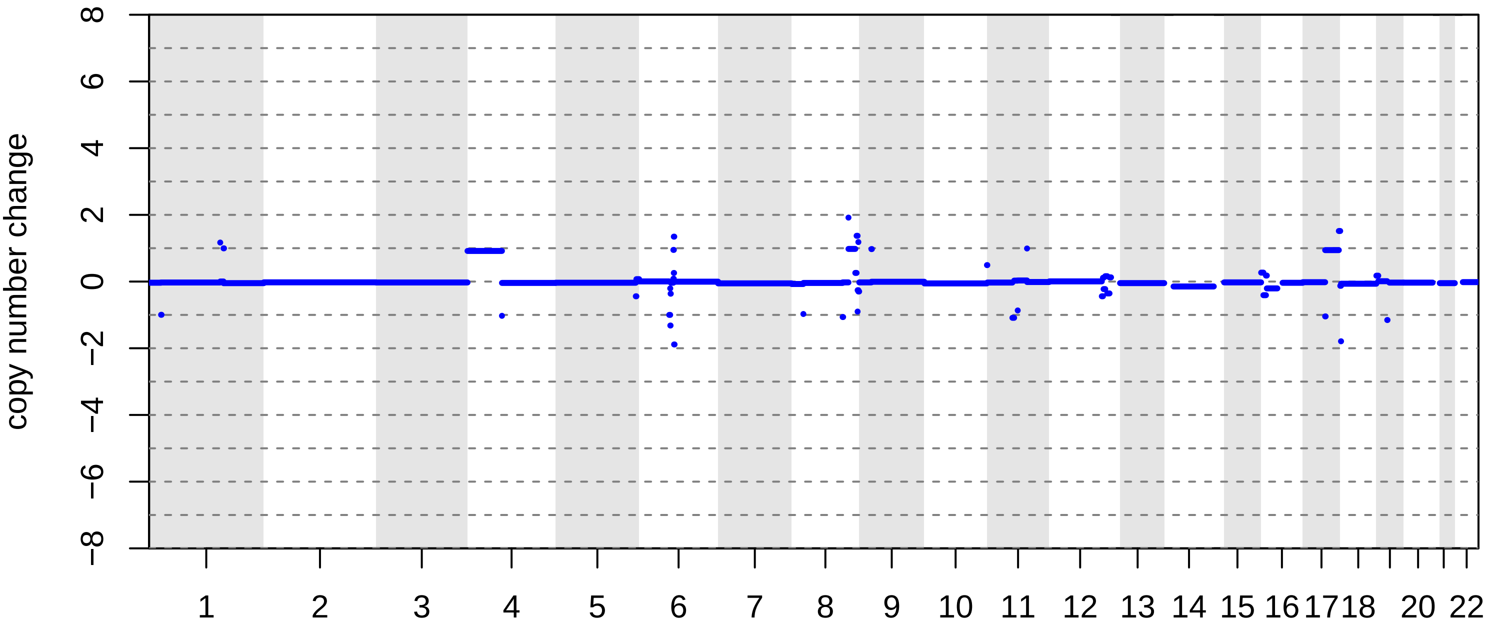

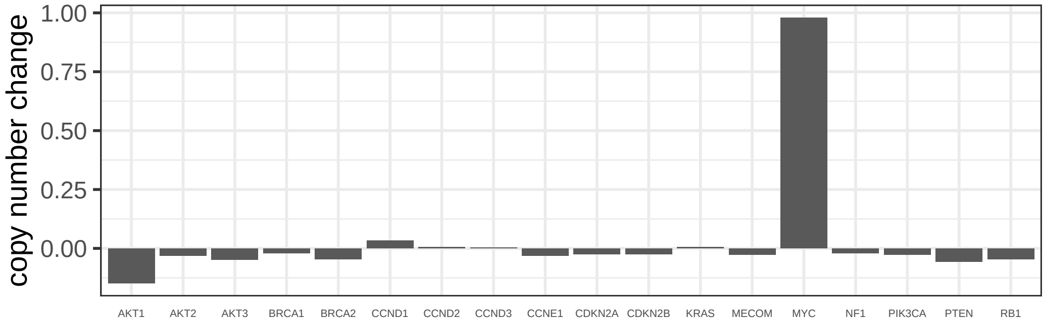

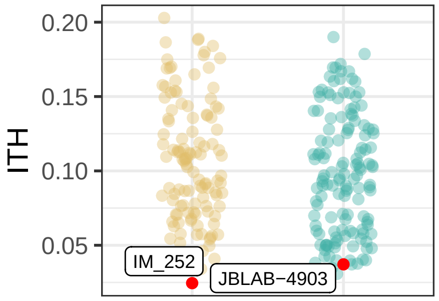

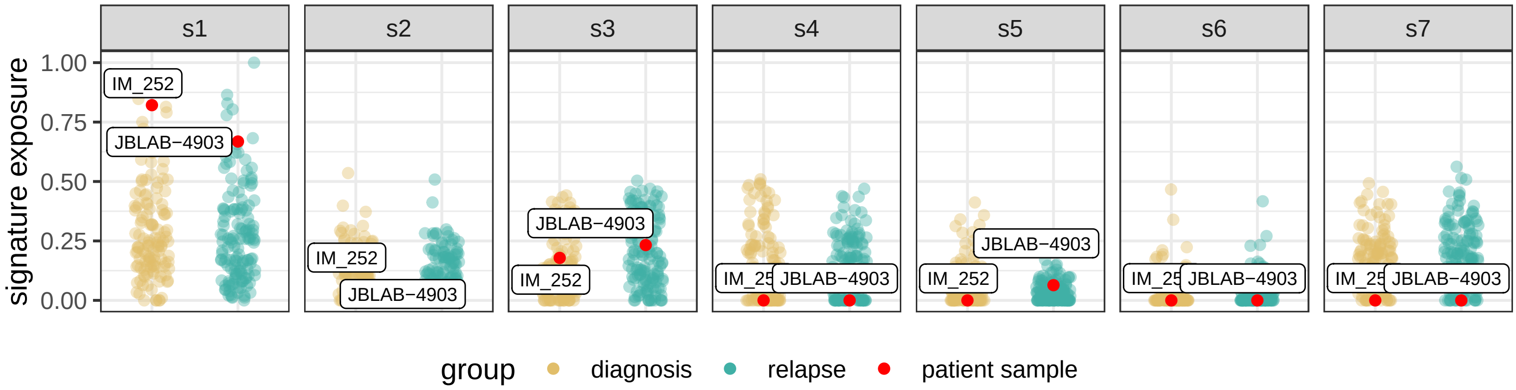

BRITROC−147

age: 57

stage: 4

platinum status: resistant

prior lines: 1

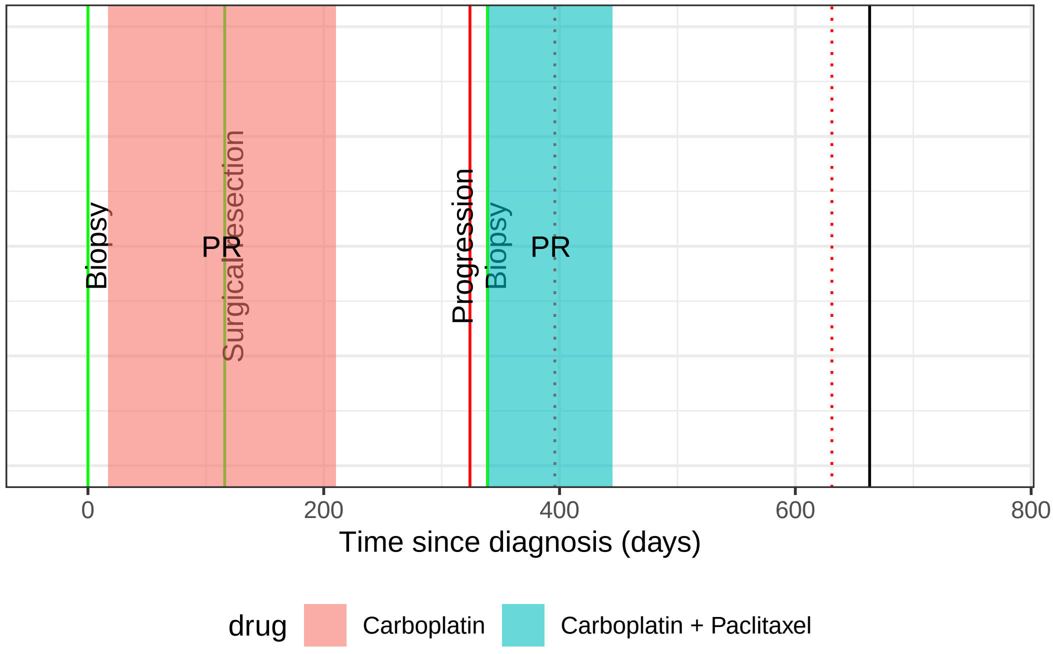

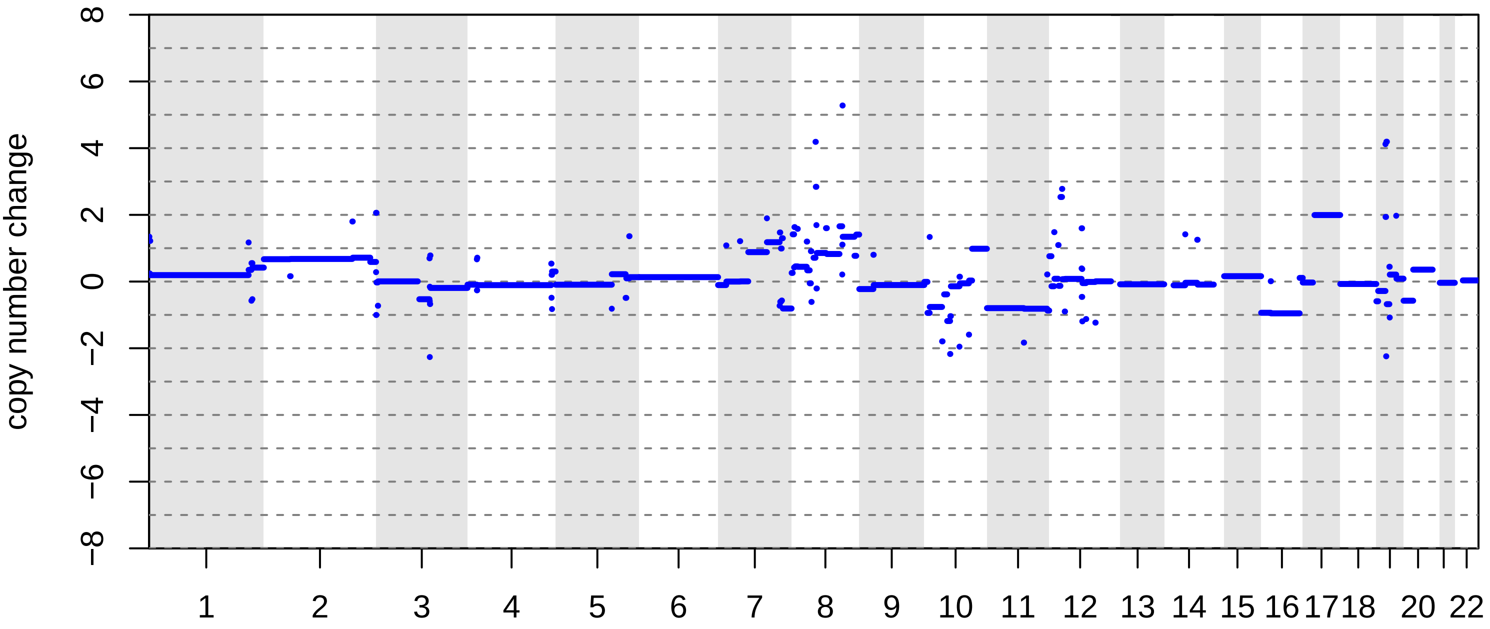

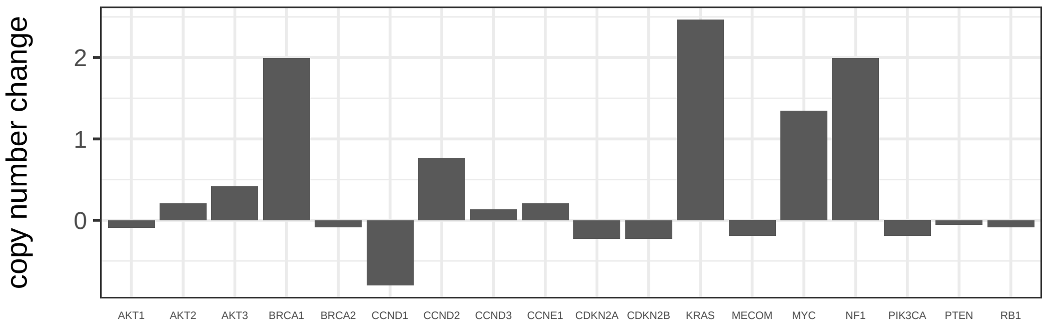

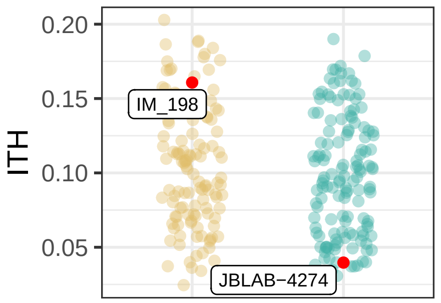

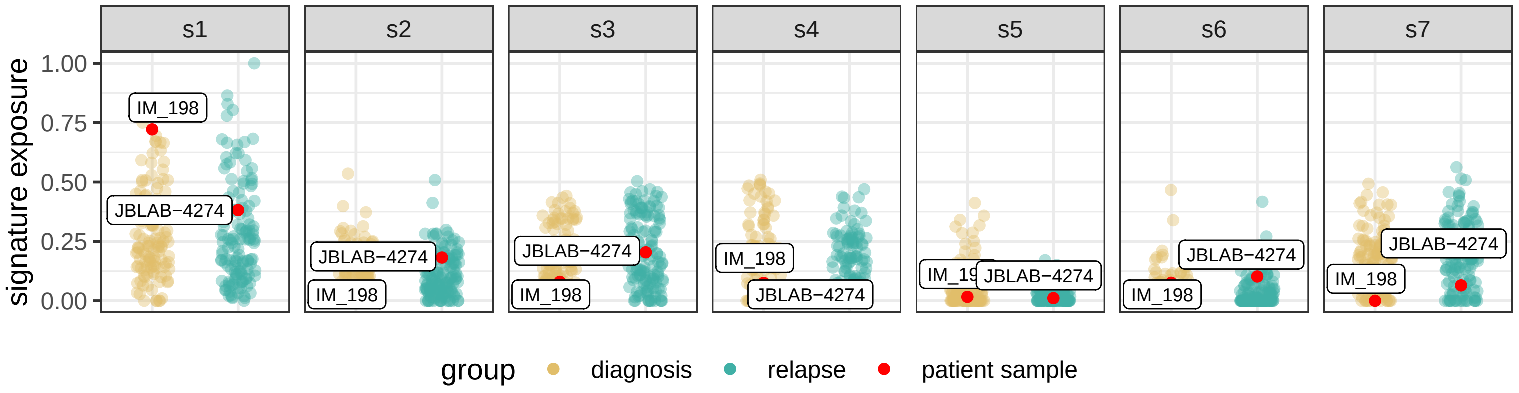

BRITROC−148

age: 74

stage: 4

platinum status: resistant

prior lines: 2

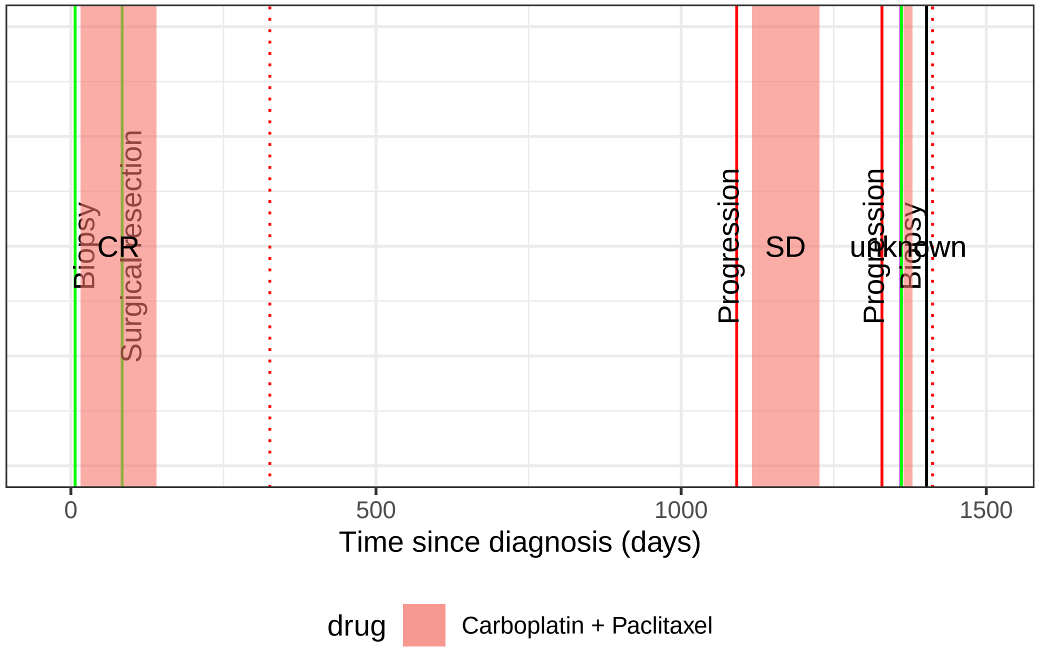

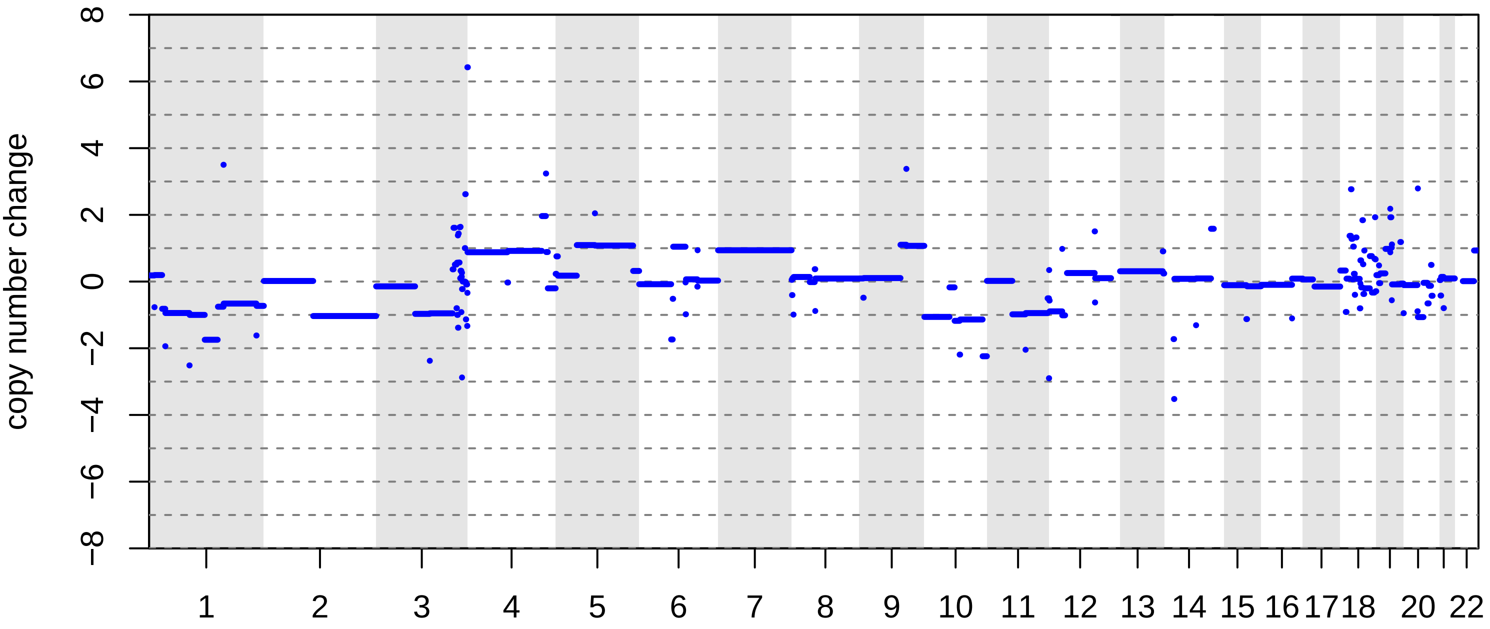

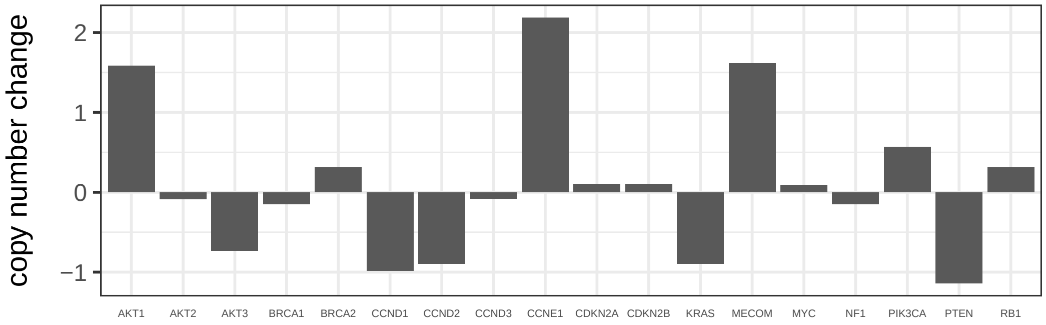

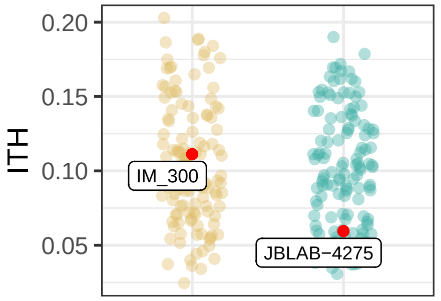

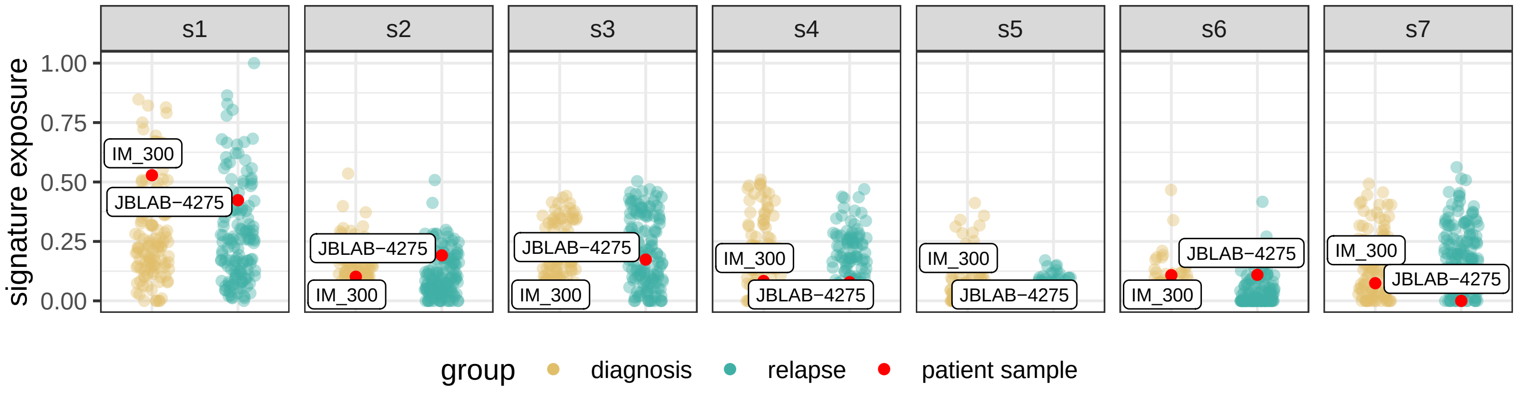

BRITROC−157

age: 67

stage: 4

platinum status: sensitive

prior lines: 1

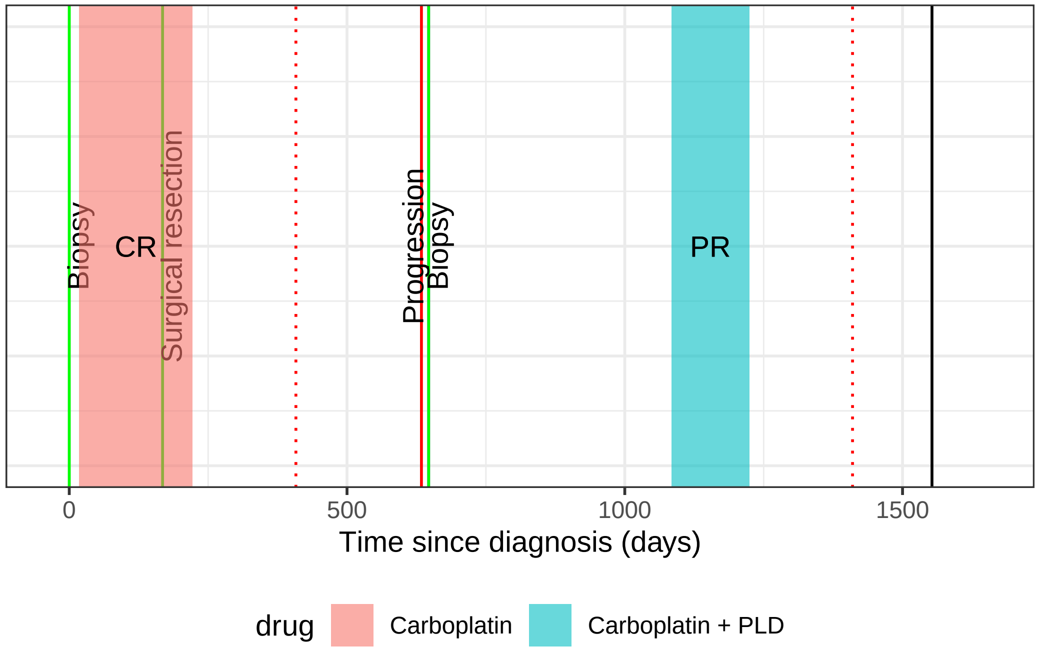

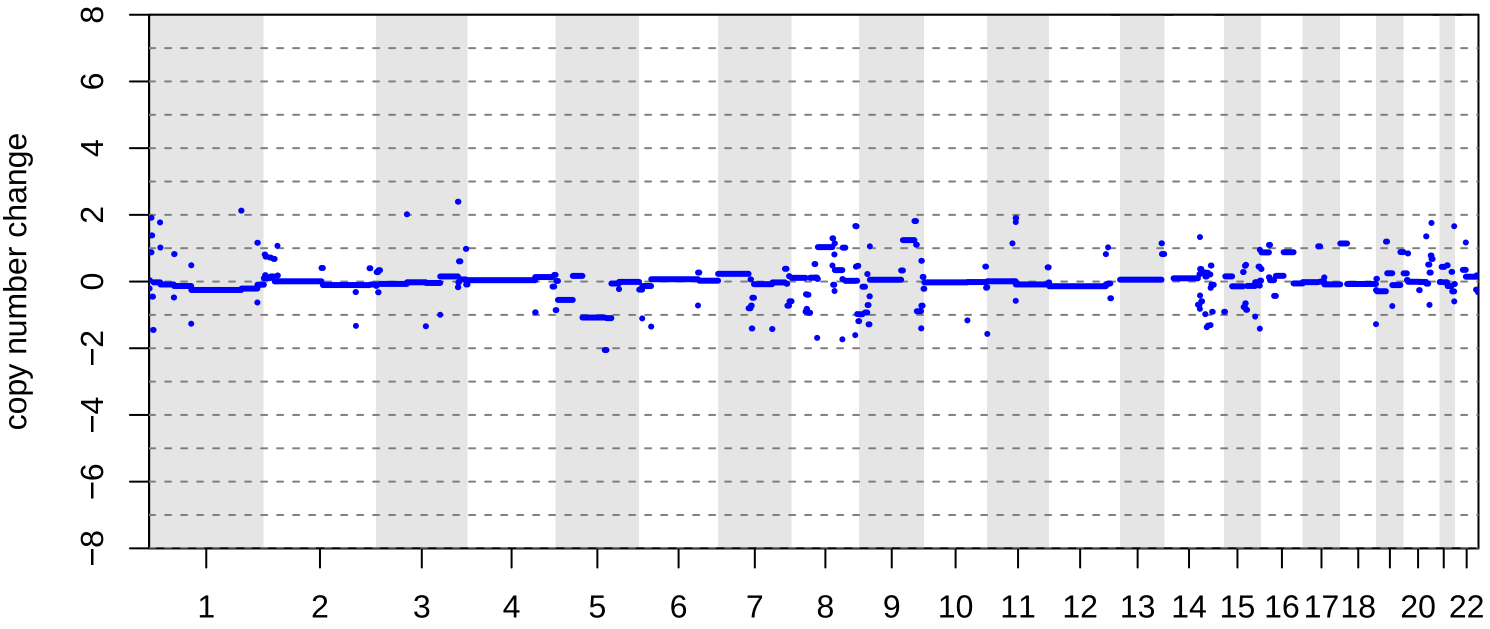

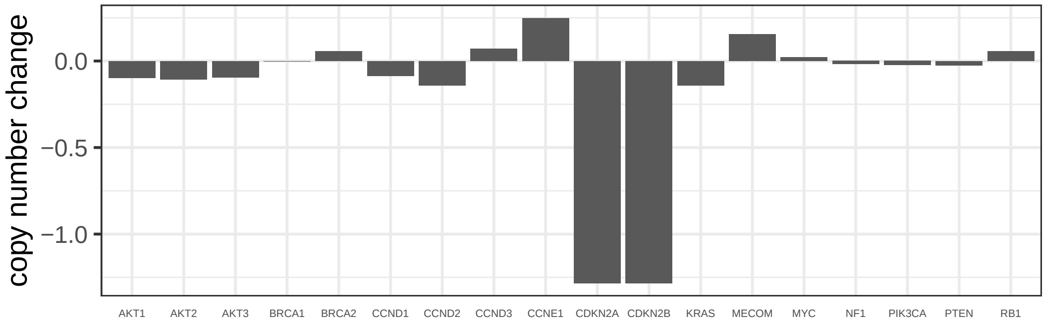

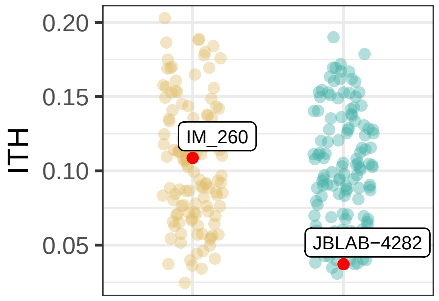

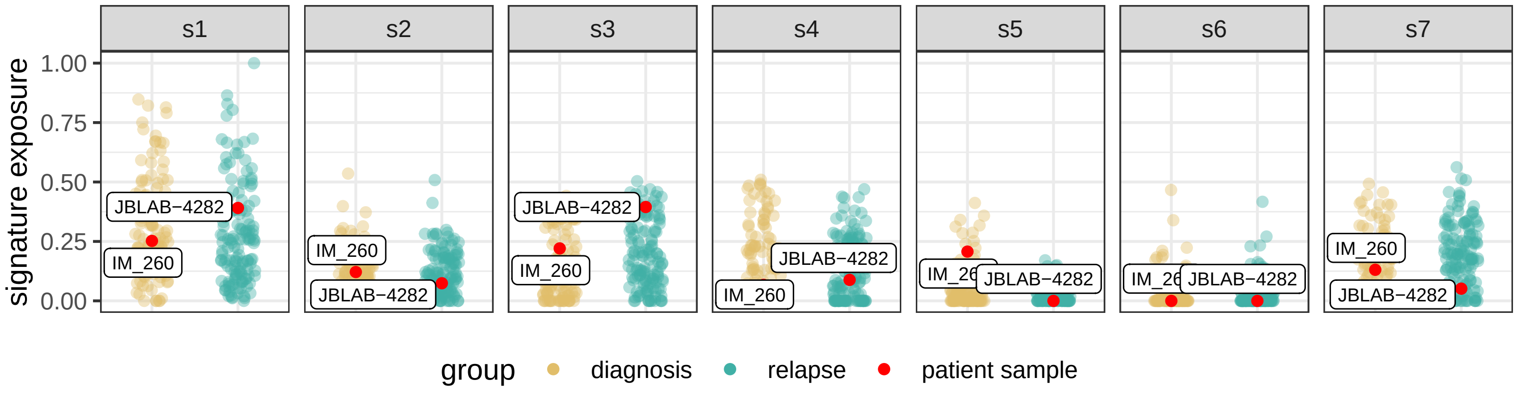

BRITROC−187

age: 77

stage: 11

platinum status: sensitive

prior lines: 1

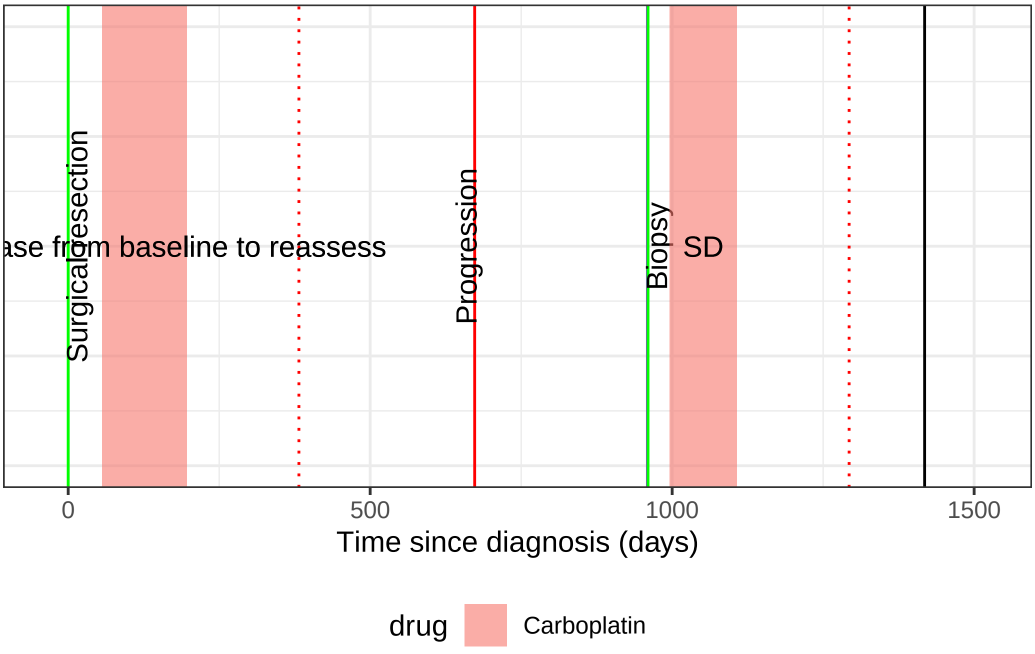

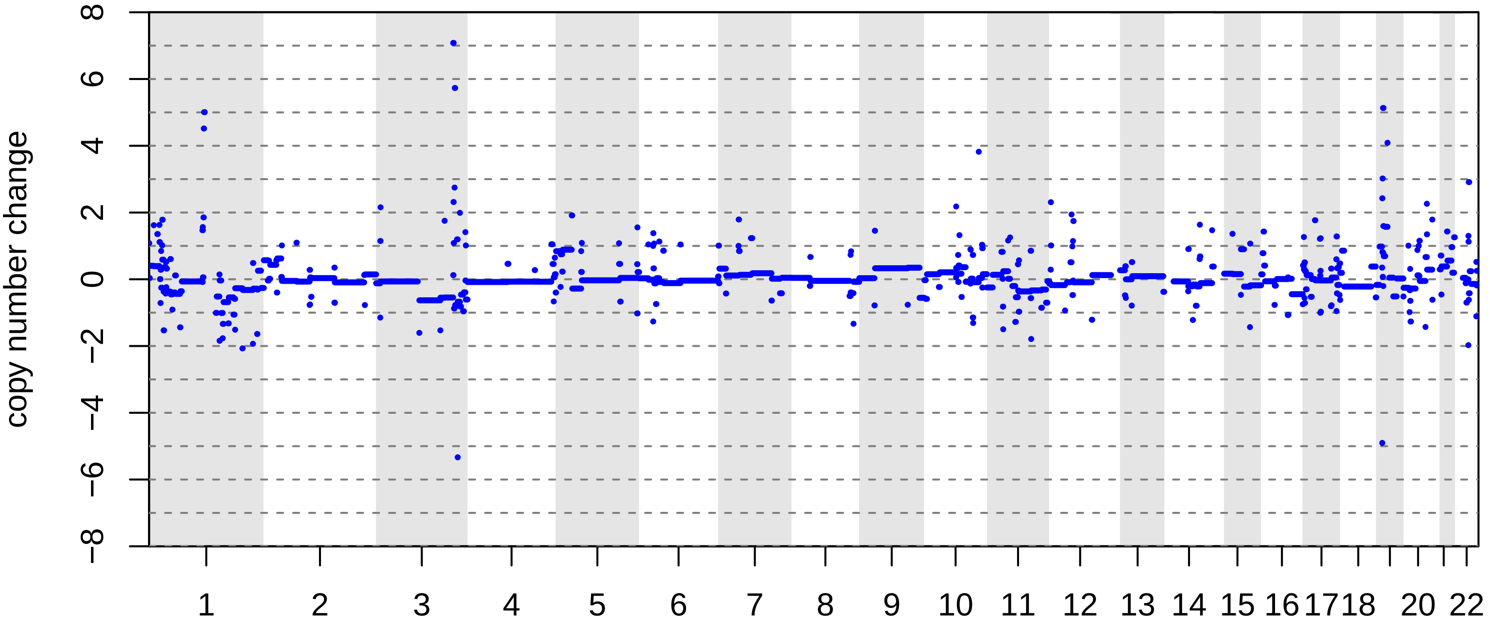

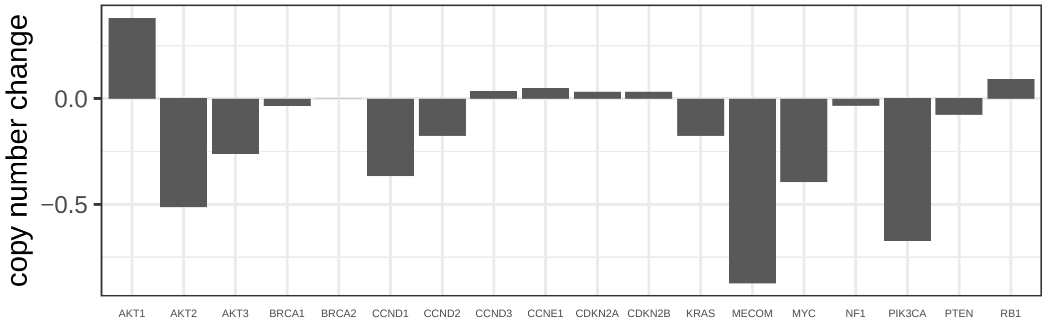

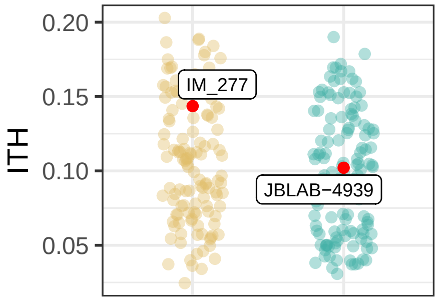

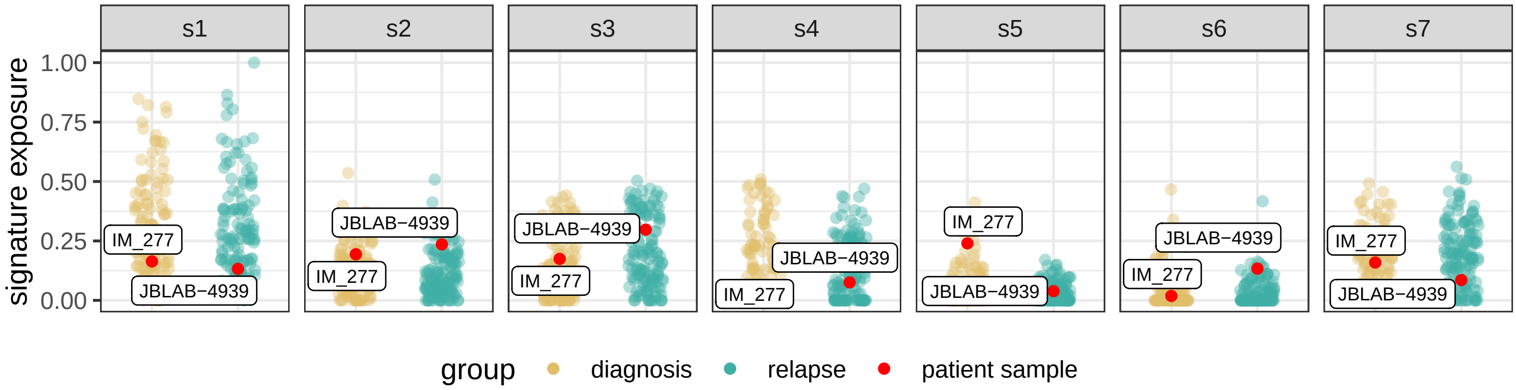

BRITROC−192

age: 48

stage: 33

platinum status: sensitive

prior lines: 1

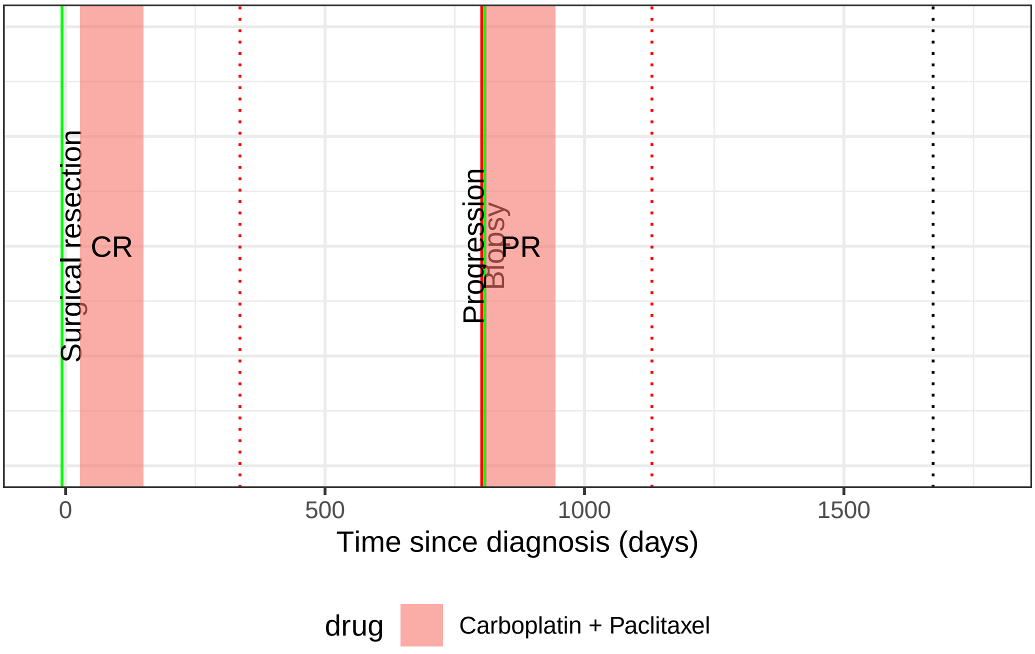

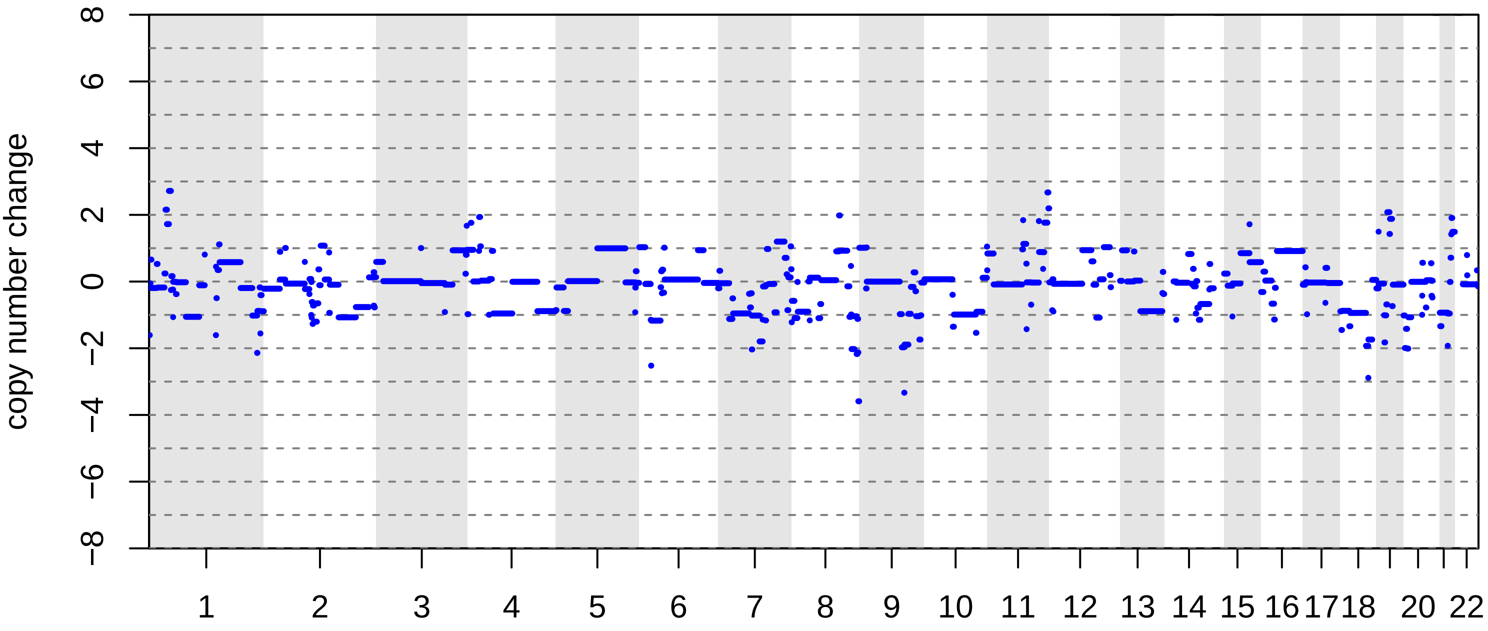

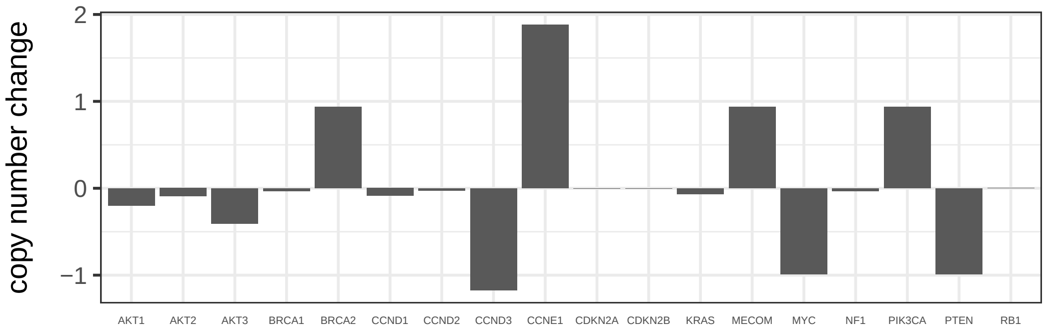

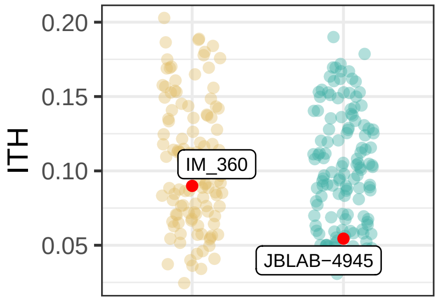

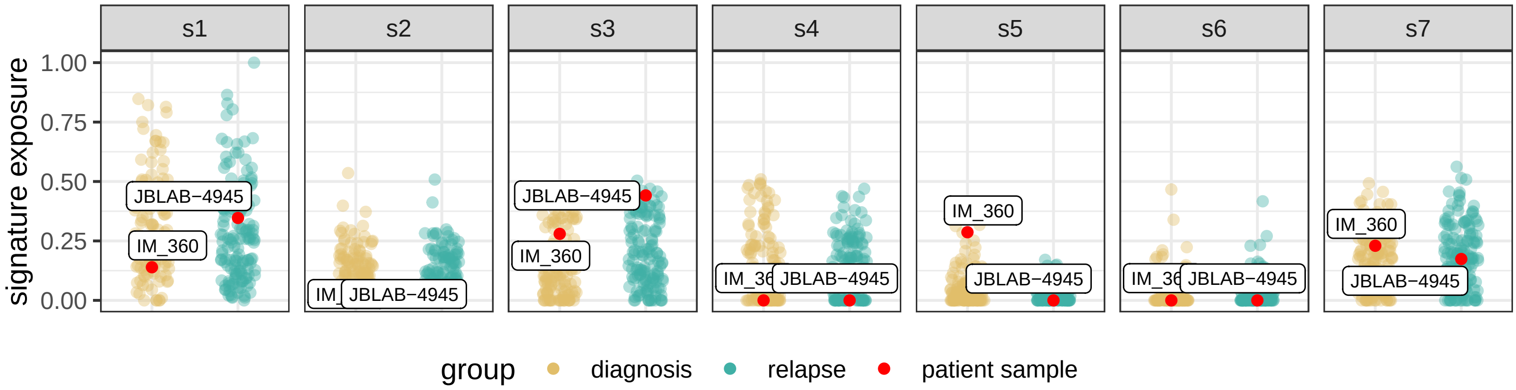

BRITROC−2

age: 73

stage: 33

platinum status: resistant

prior lines: 1

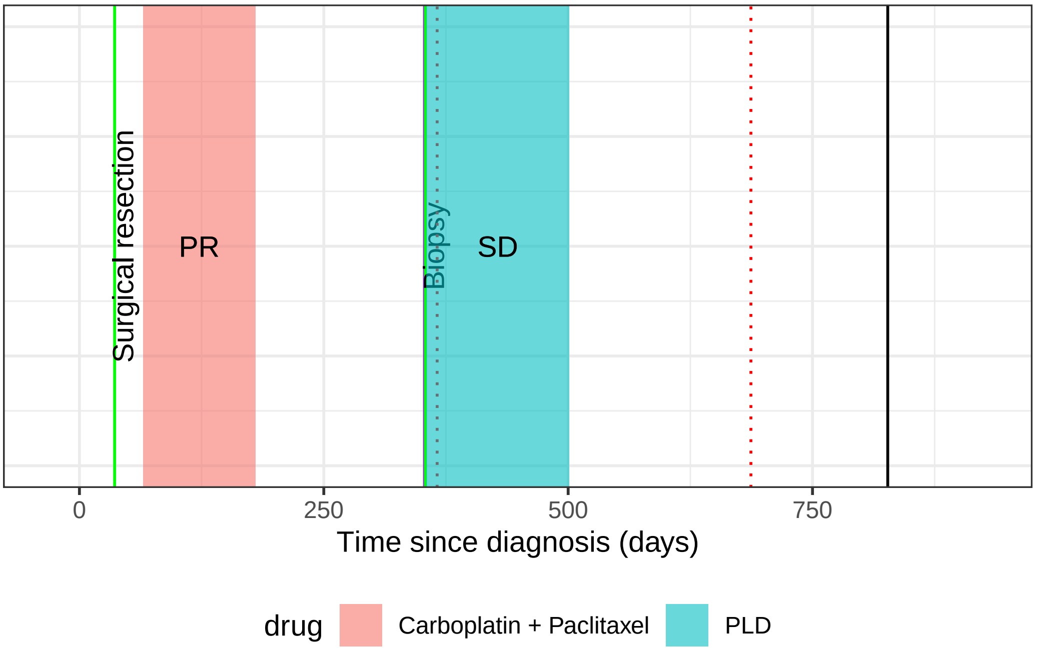

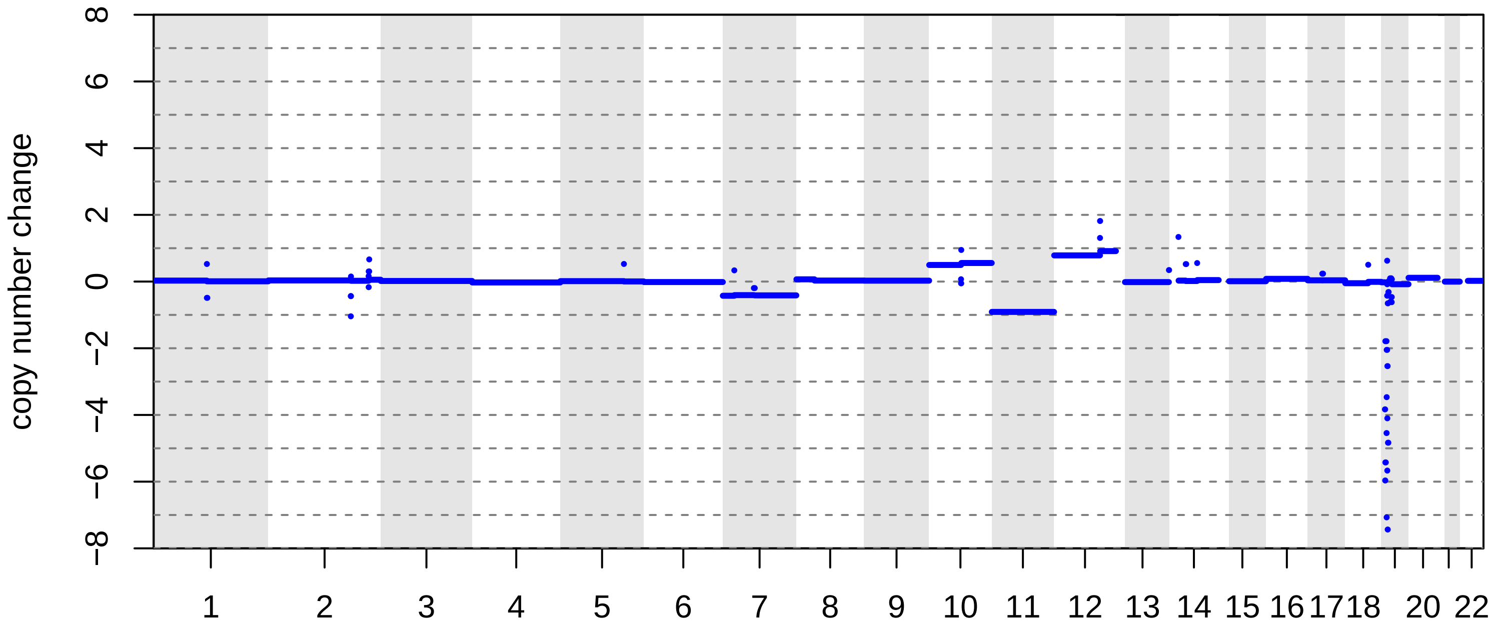

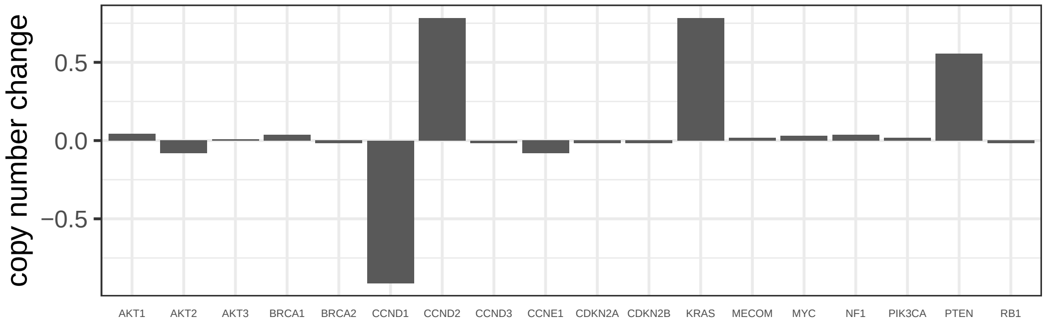

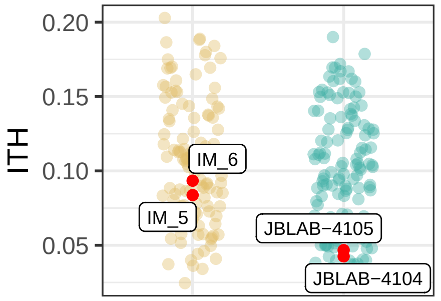

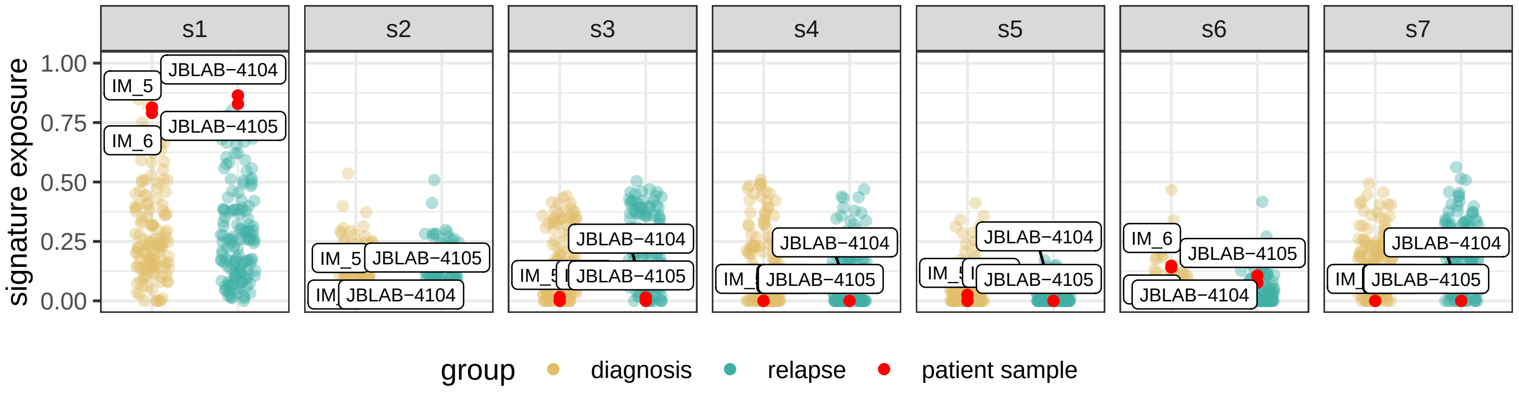

BRITROC−204

age: 50

stage: 22

platinum status: sensitive

prior lines: 1

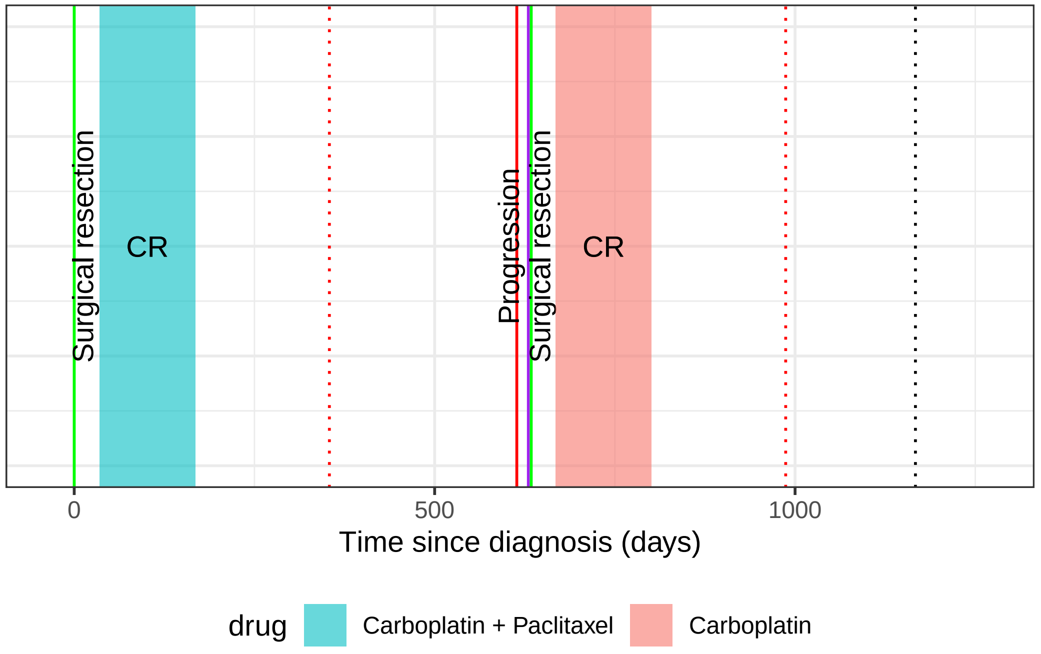

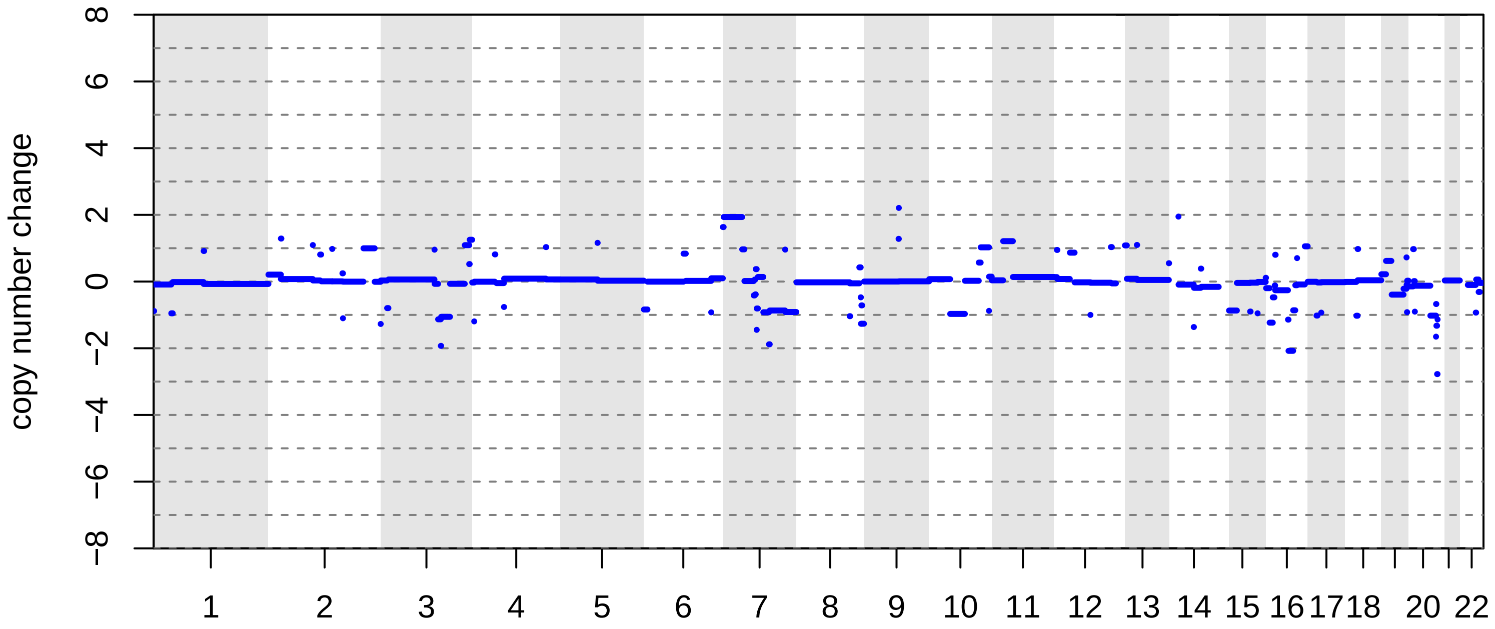

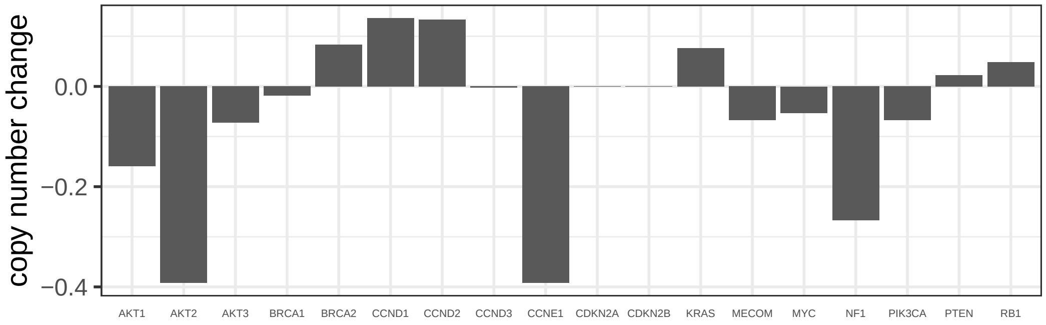

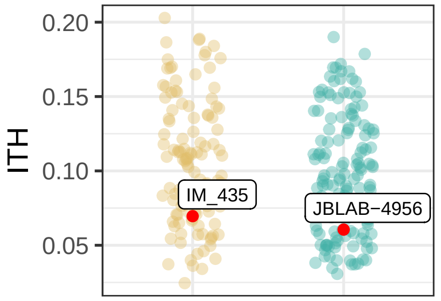

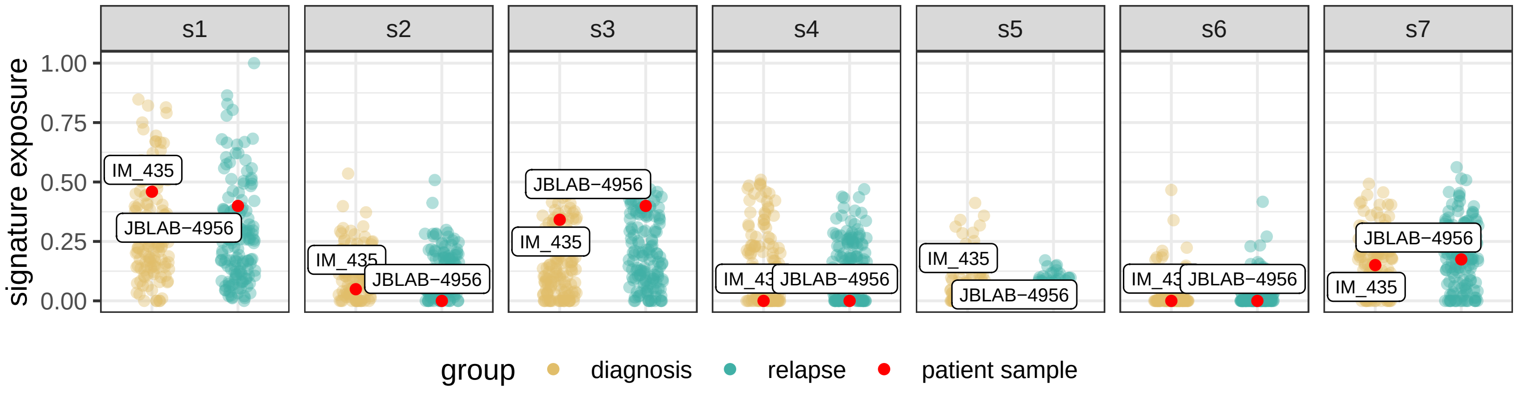

BRITROC−205

age: 53

stage: 33

platinum status: sensitive

prior lines: 1

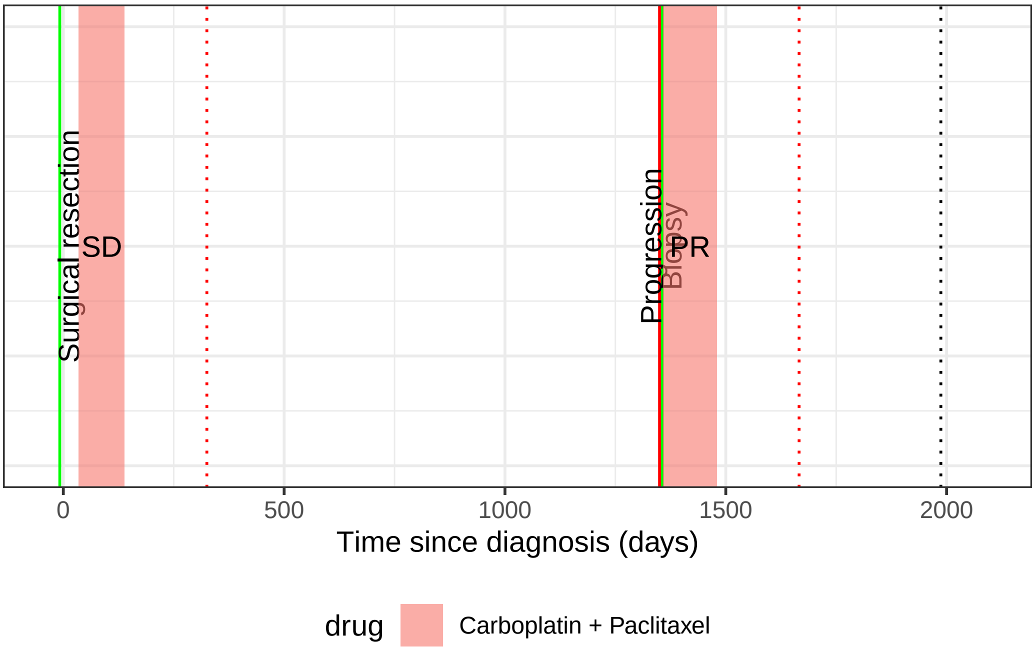

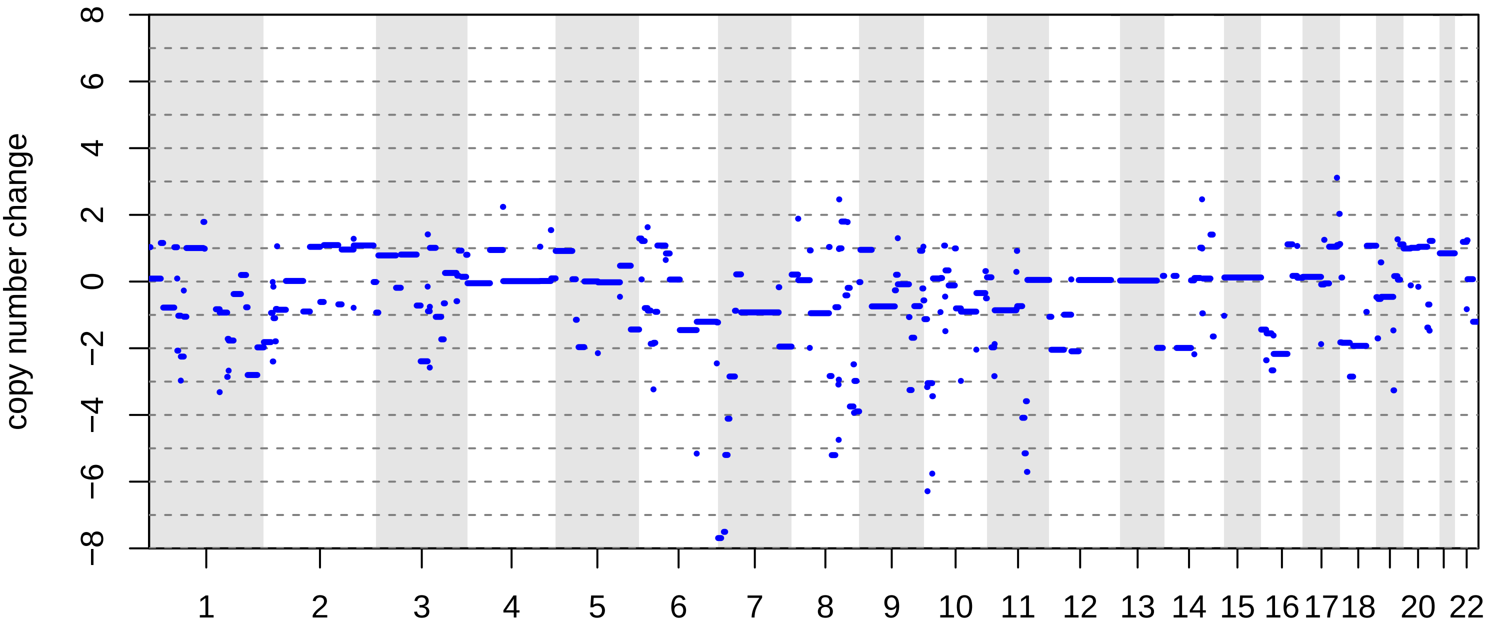

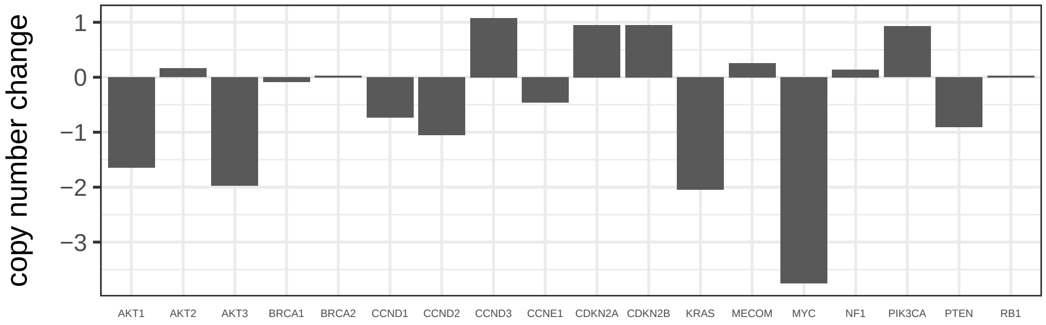

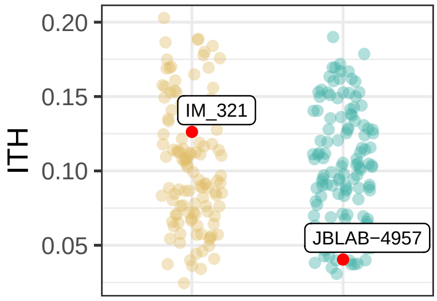

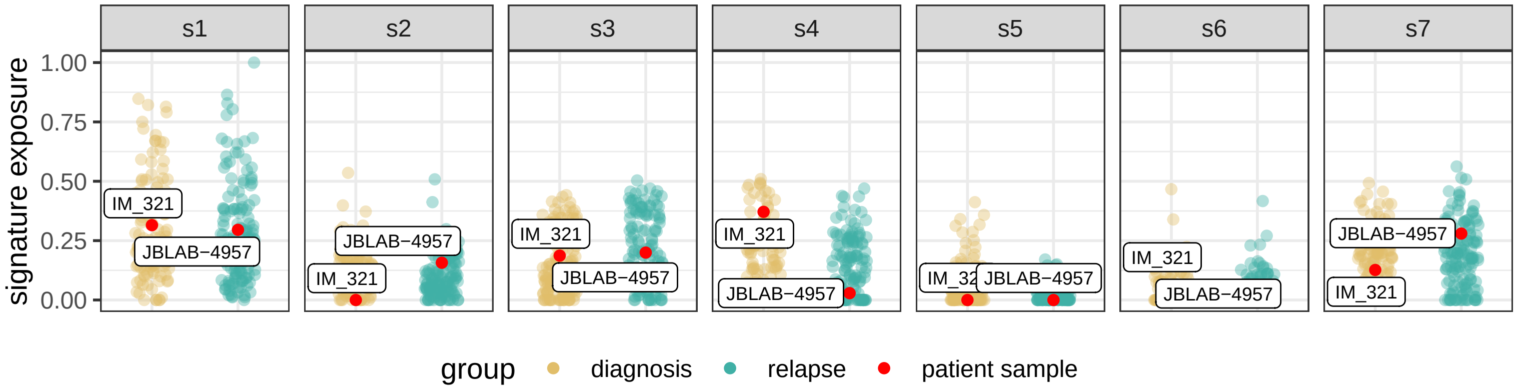

BRITROC−209

age: 42

stage: 33

platinum status: sensitive

prior lines: 1

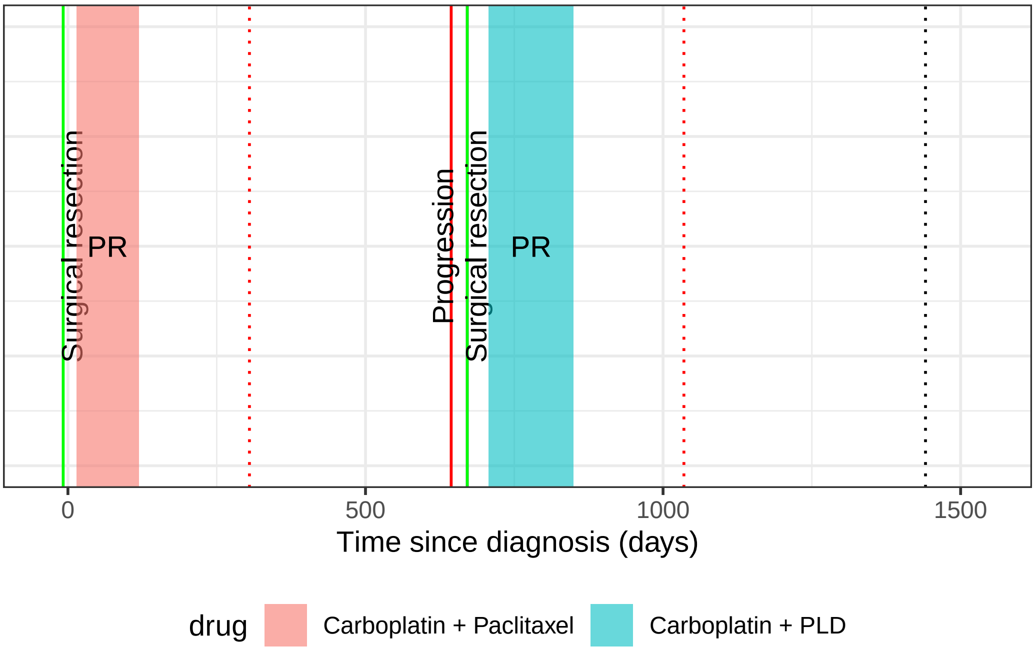

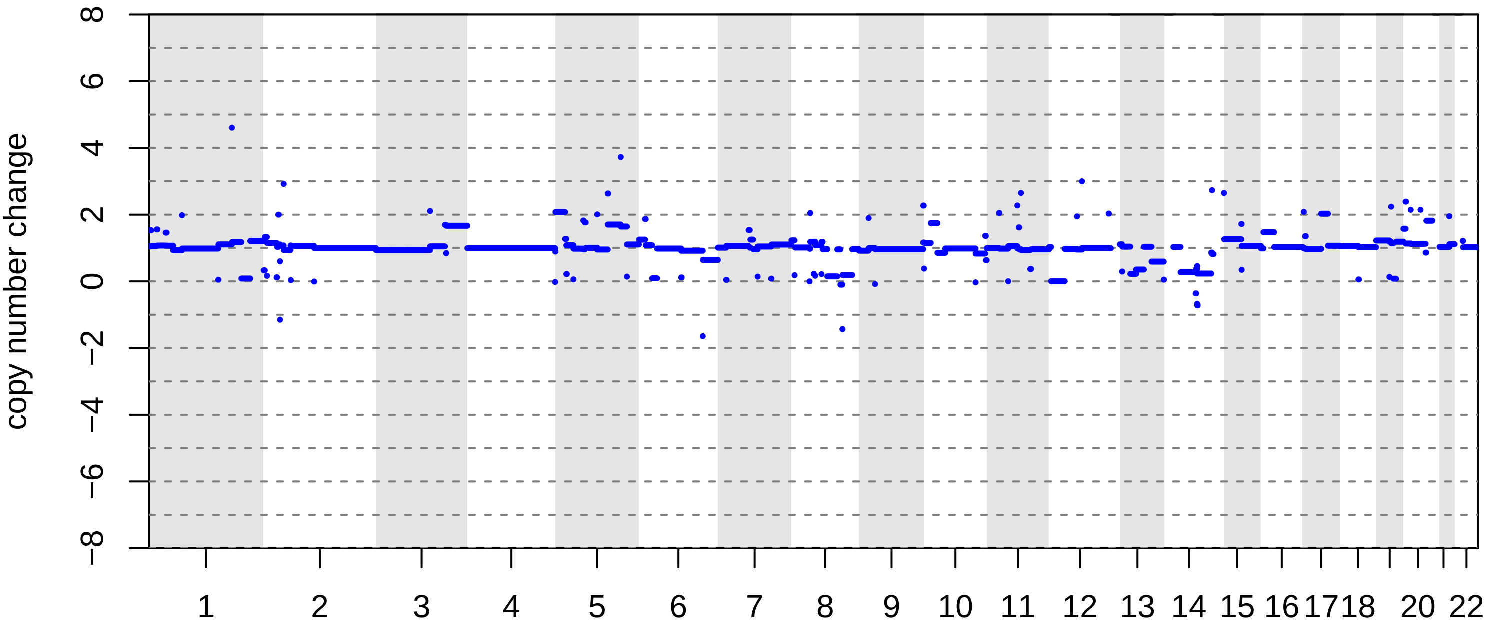

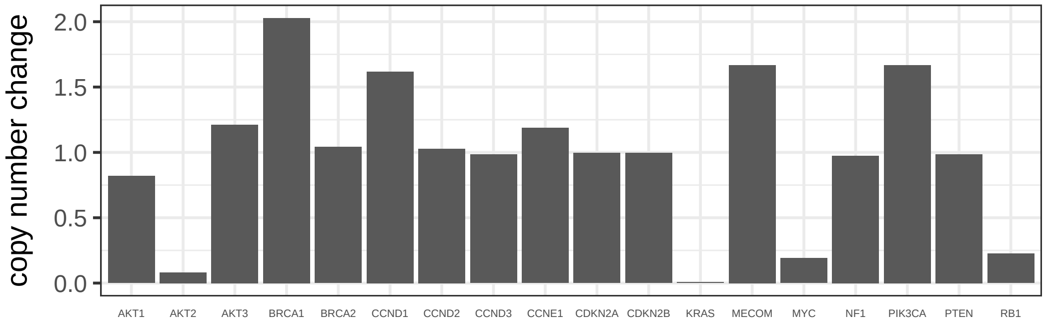

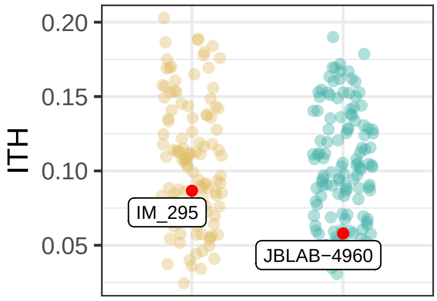

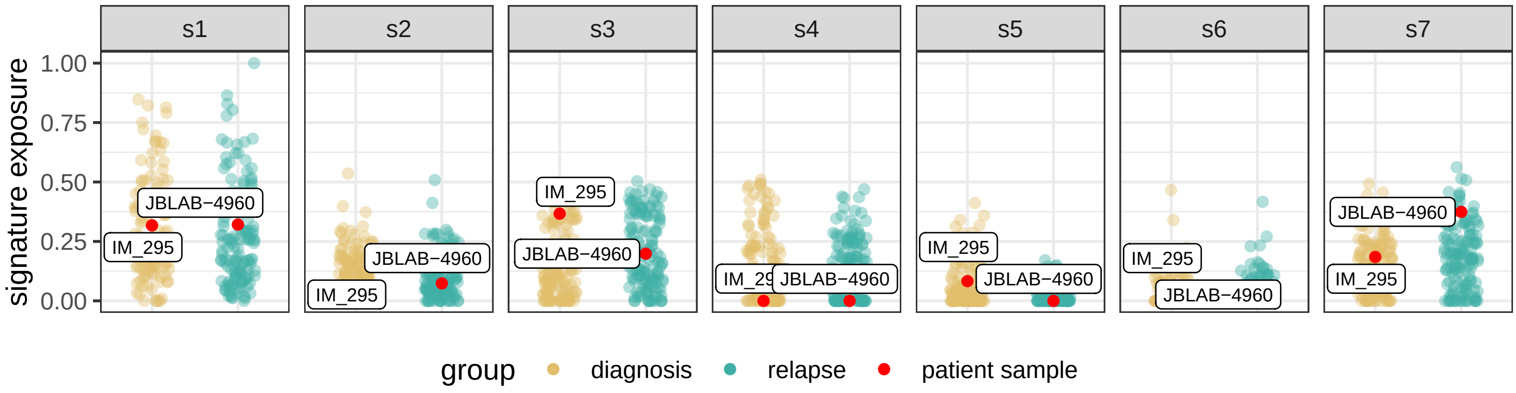

BRITROC−216

age: 70

stage: 32

platinum status: sensitive

prior lines: 1

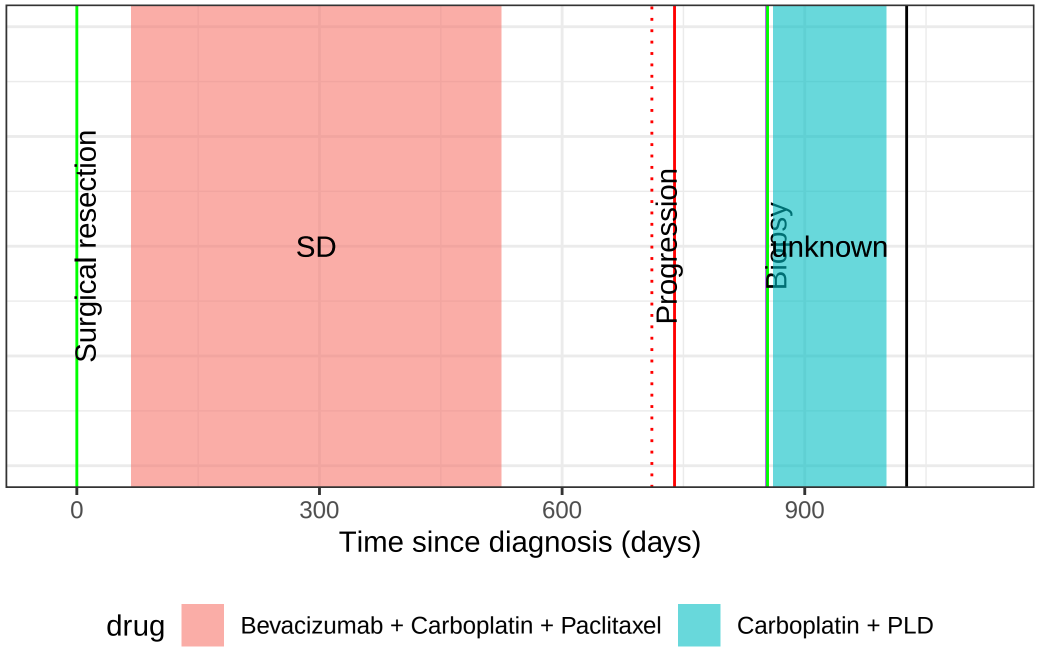

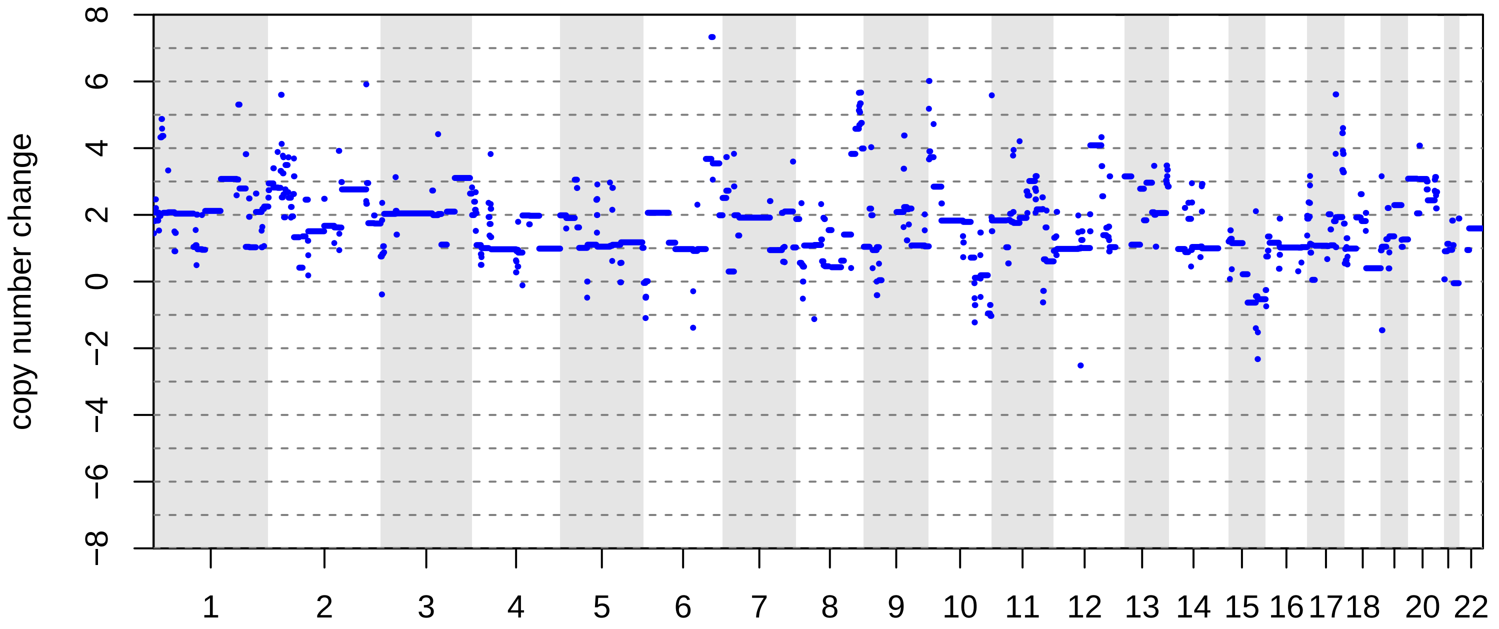

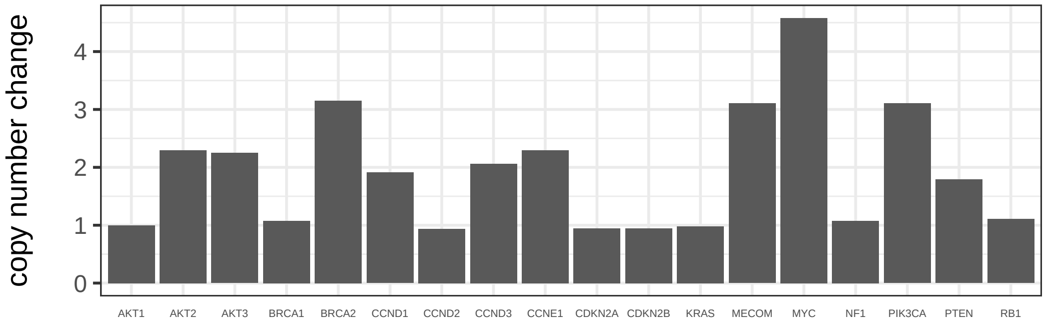

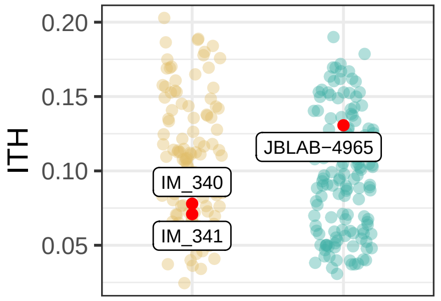

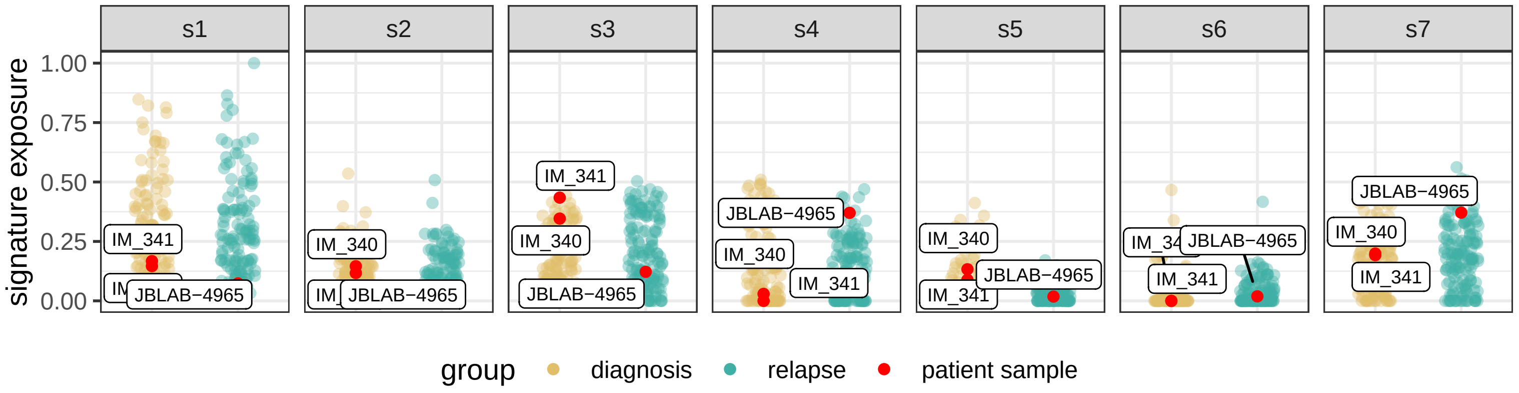

BRITROC−225

age: 46

stage: 33

platinum status: sensitive

prior lines: 1

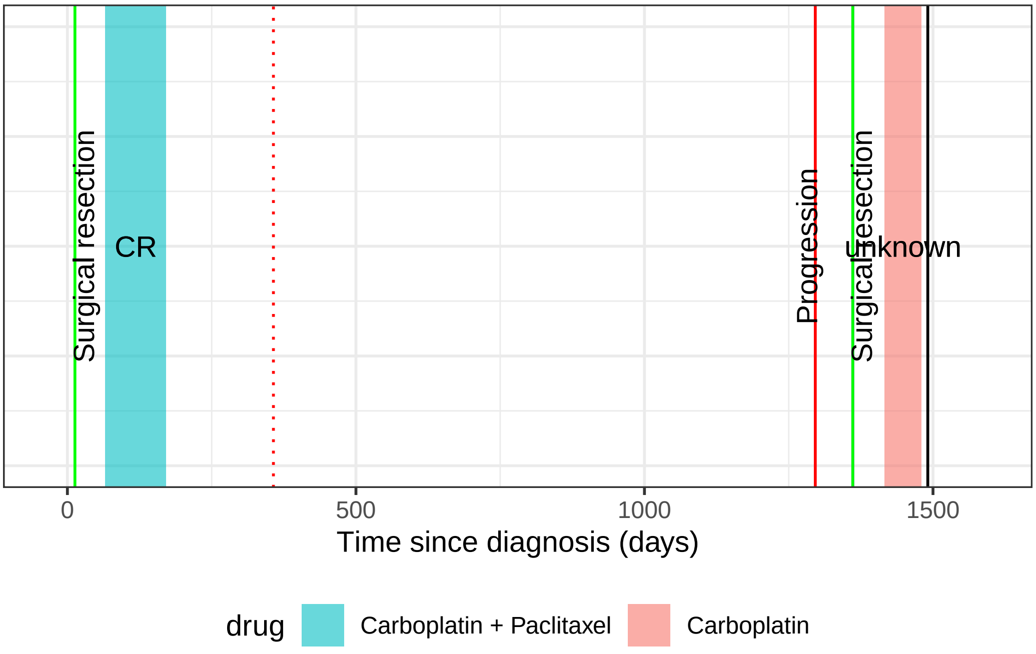

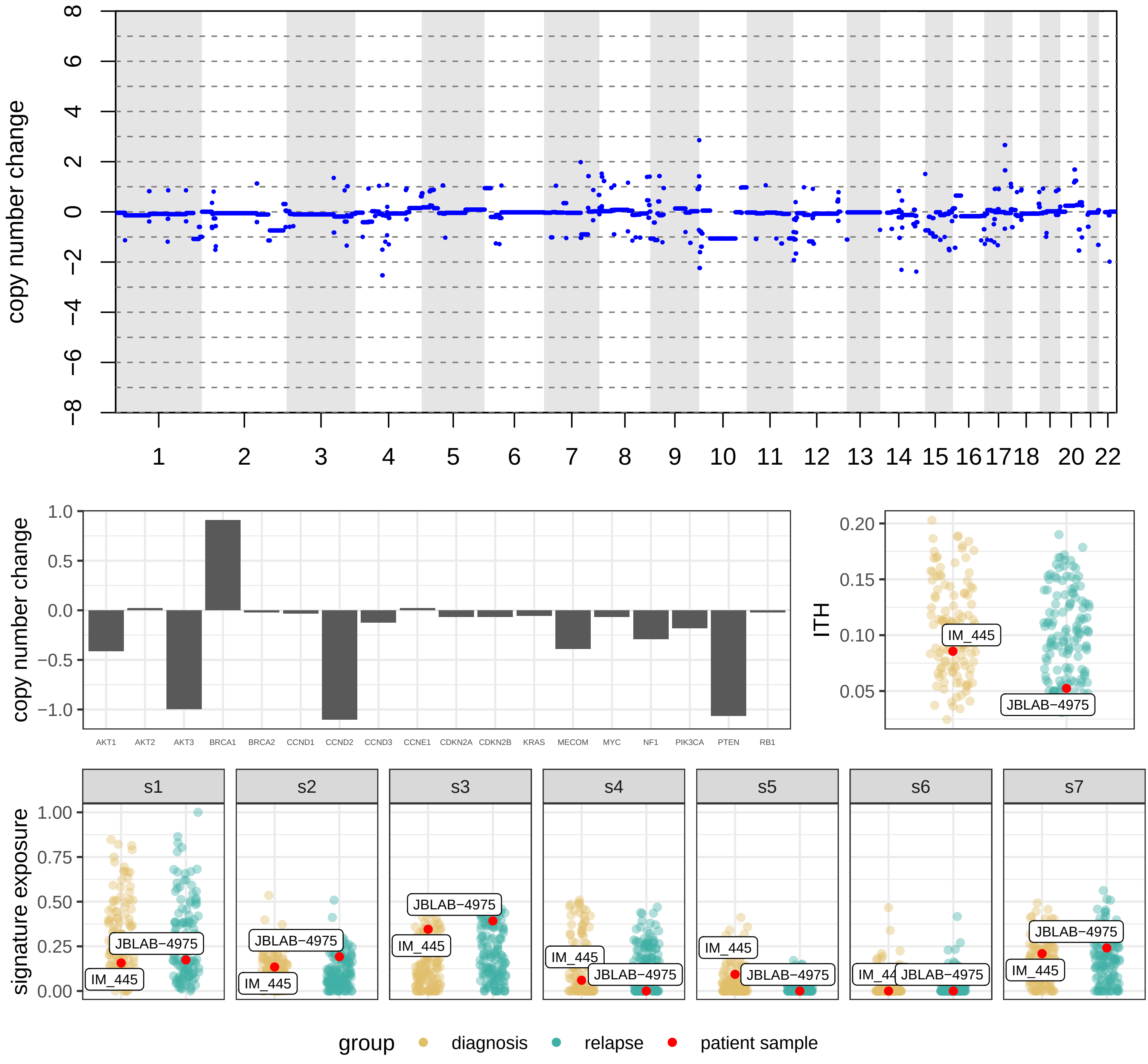

BRITROC−226

age: 70

stage: 32

platinum status: sensitive

prior lines: 1

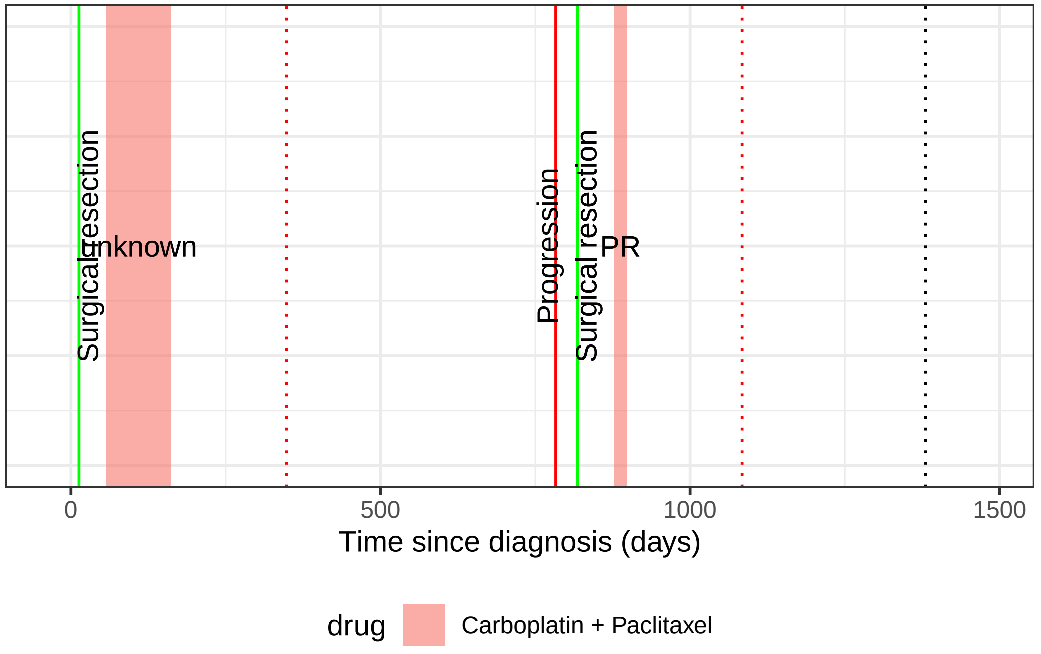

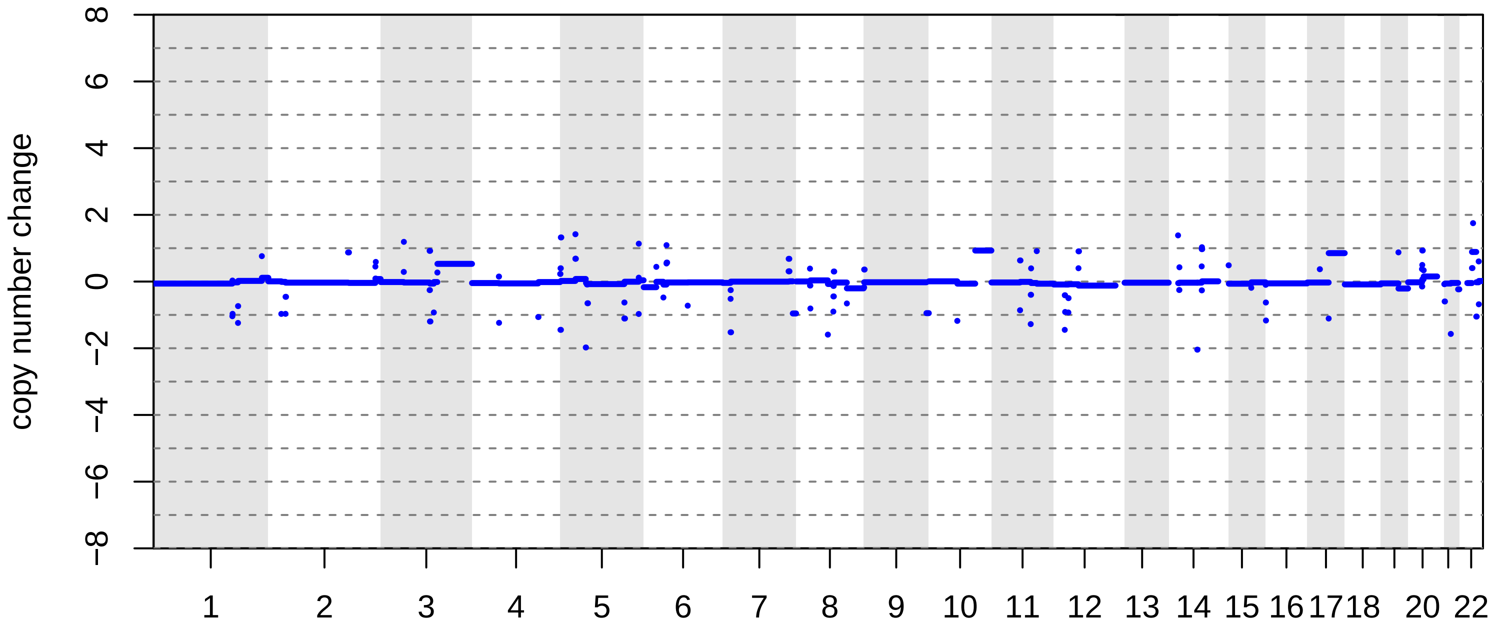

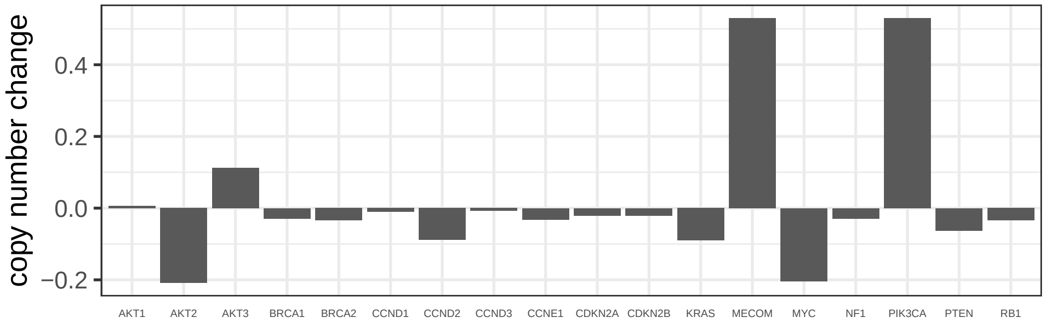

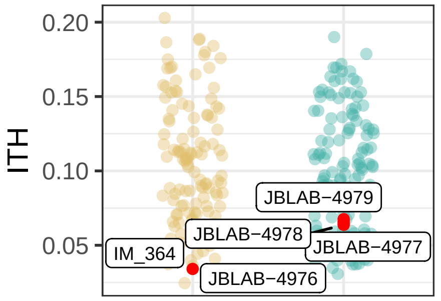

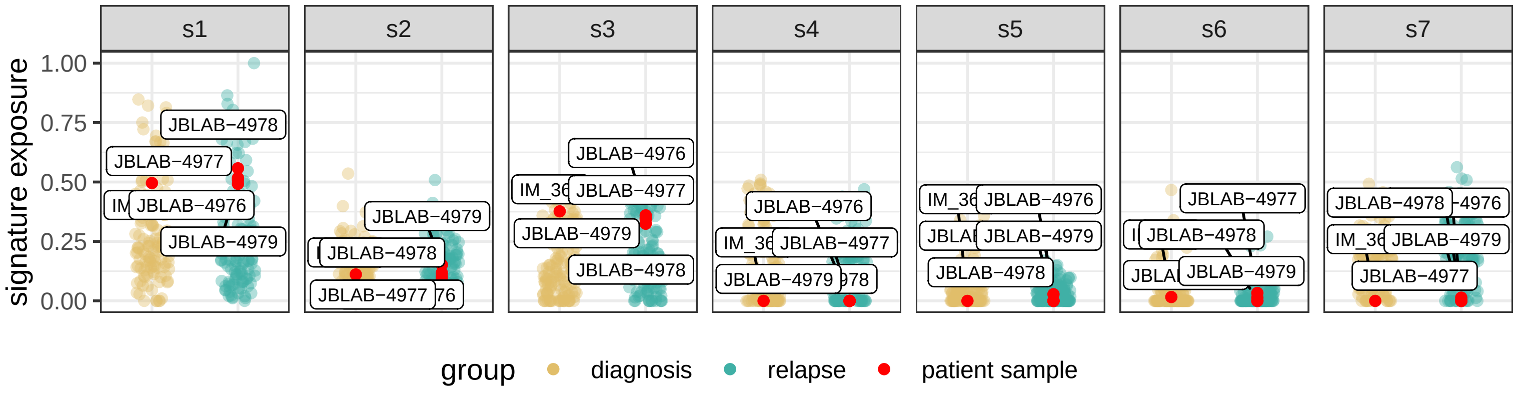

BRITROC−229

age: 75

stage: 33

platinum status: resistant

prior lines: 1

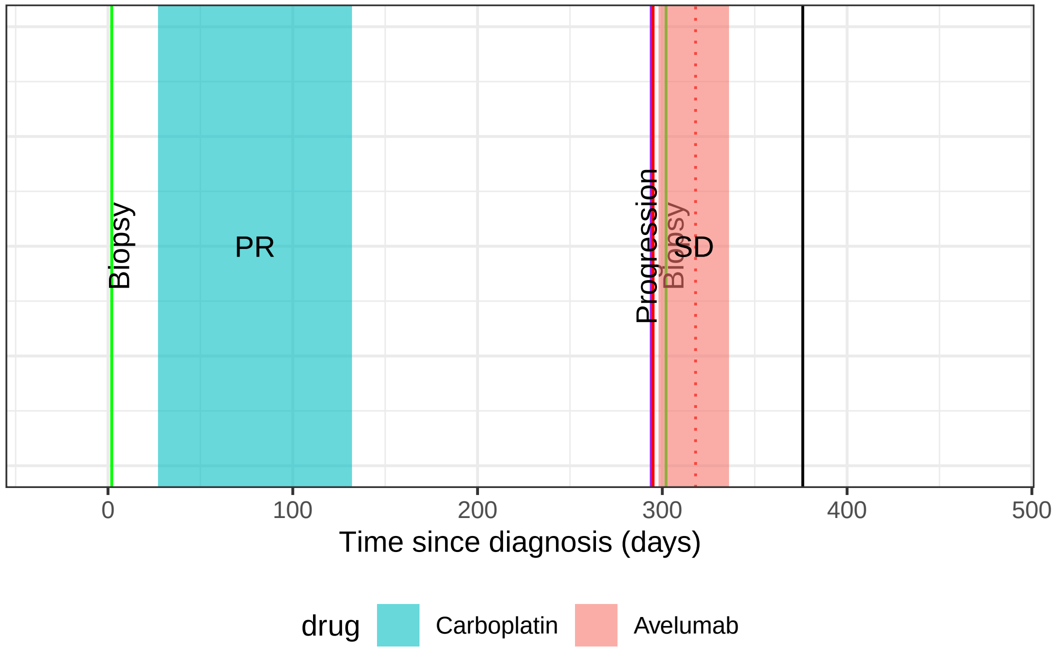

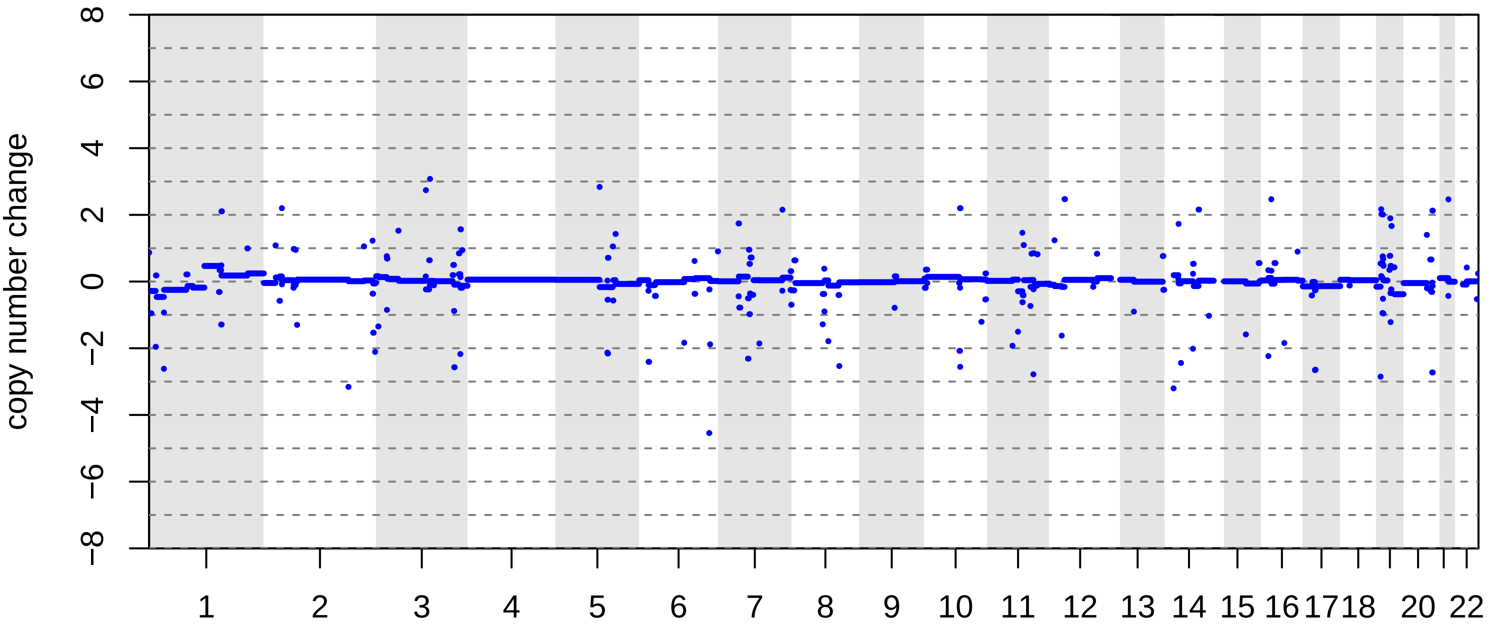

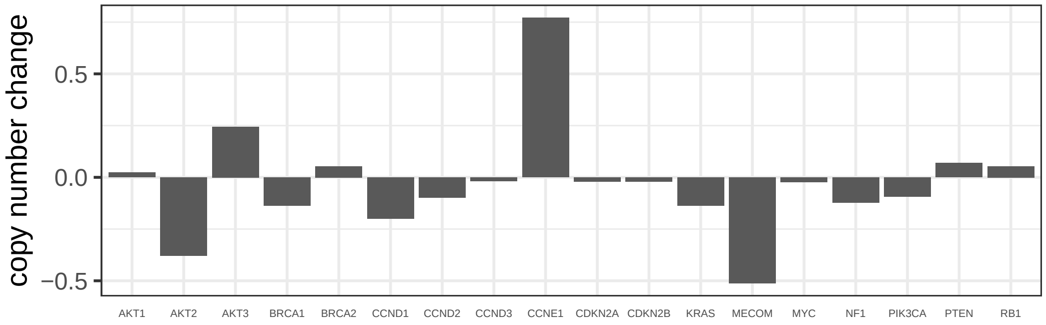

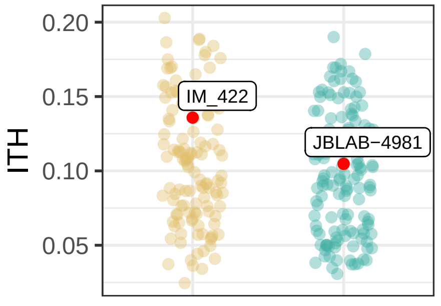

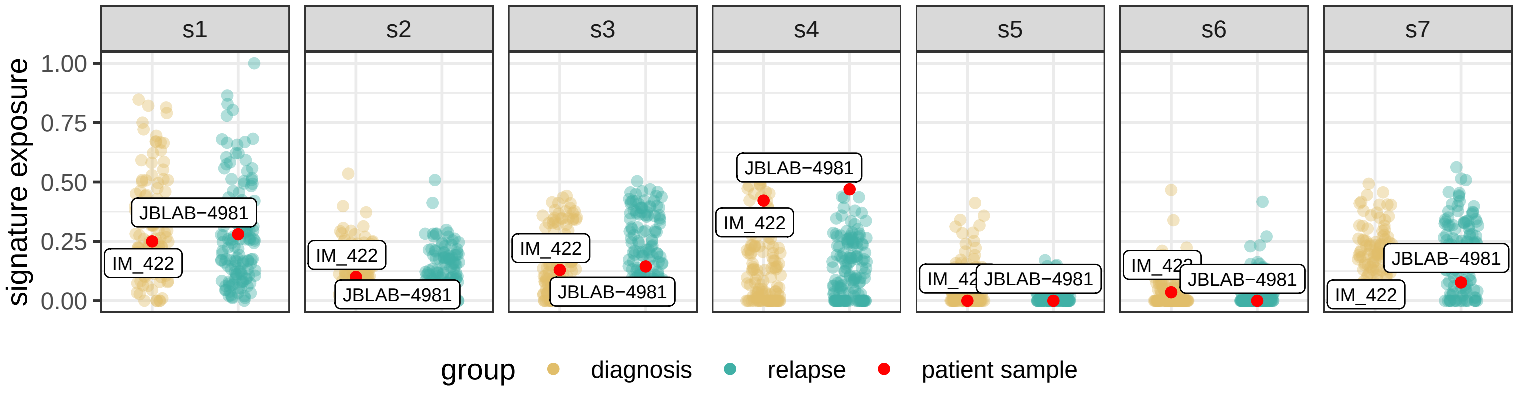

BRITROC−23

age: 47

stage: 33

platinum status: sensitive

prior lines: 2

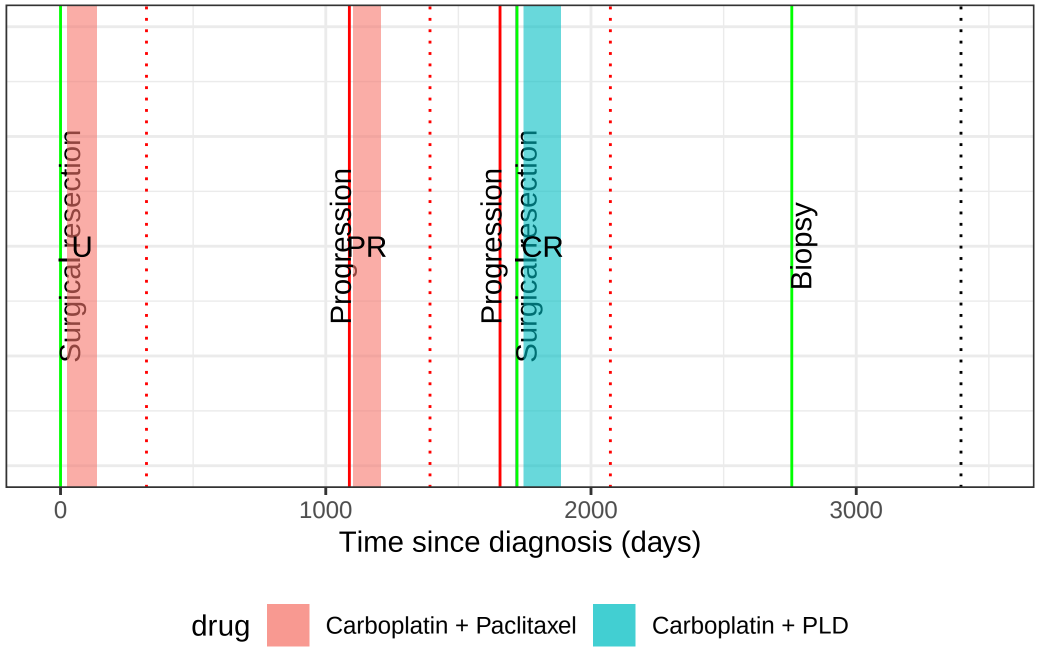

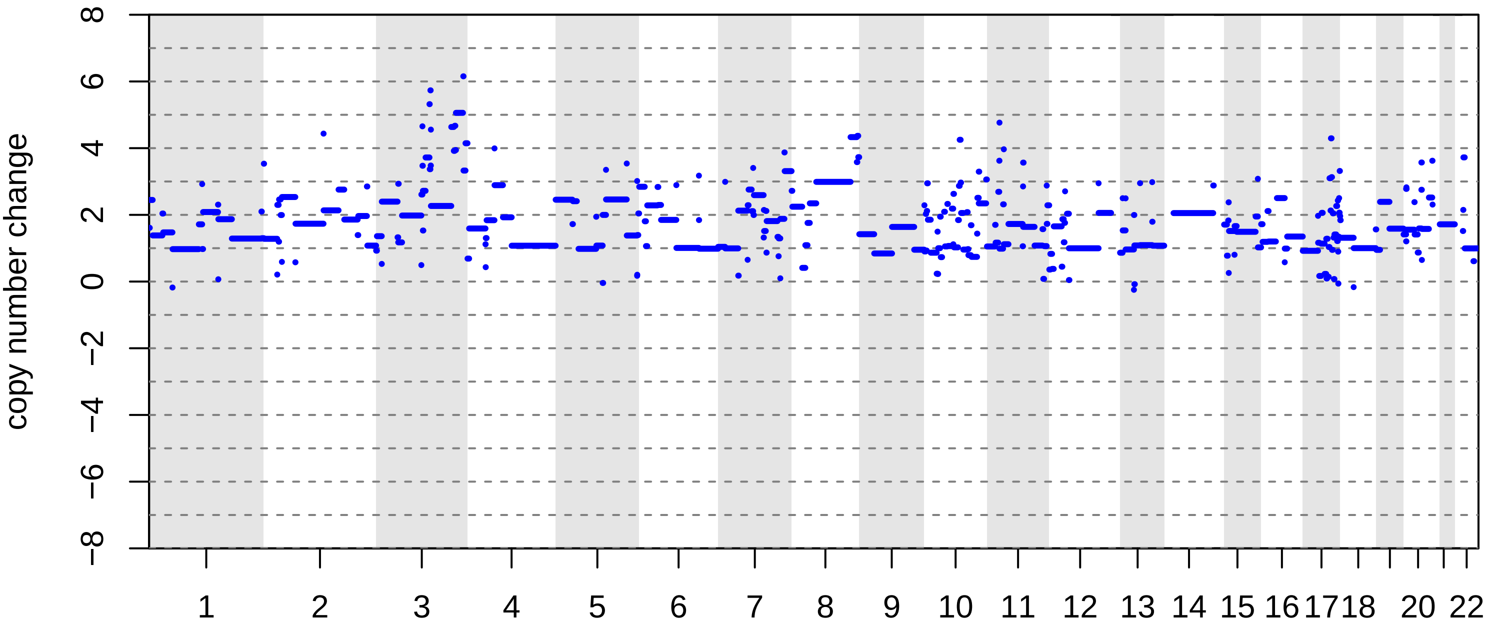

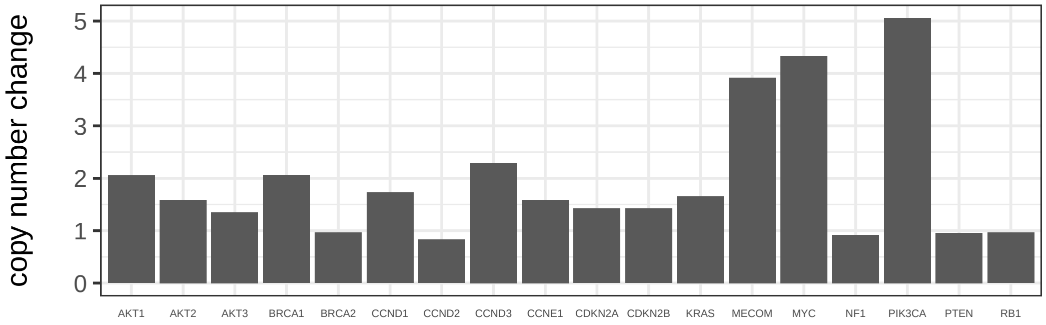

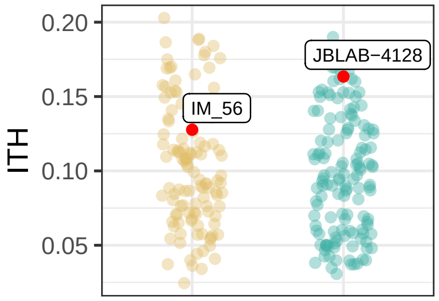

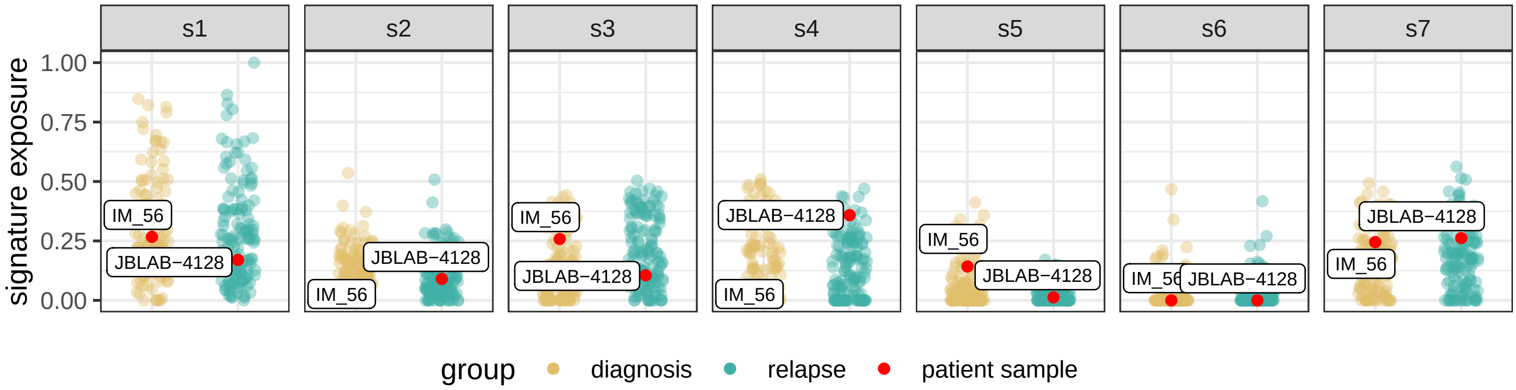

BRITROC−232

age: 63

stage: 22

platinum status: sensitive

prior lines: 1

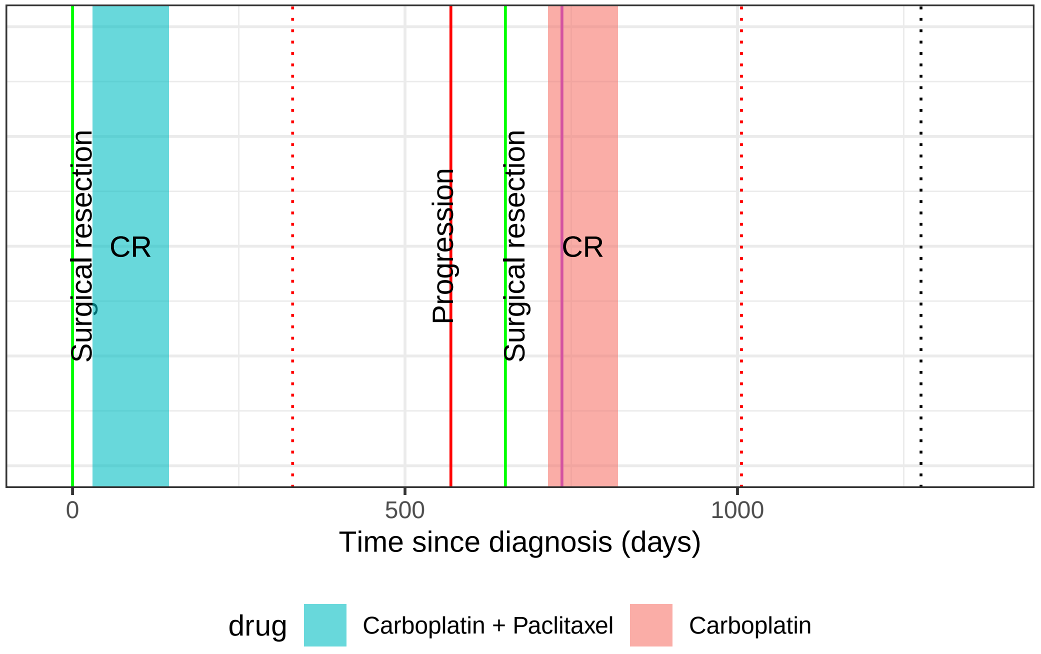

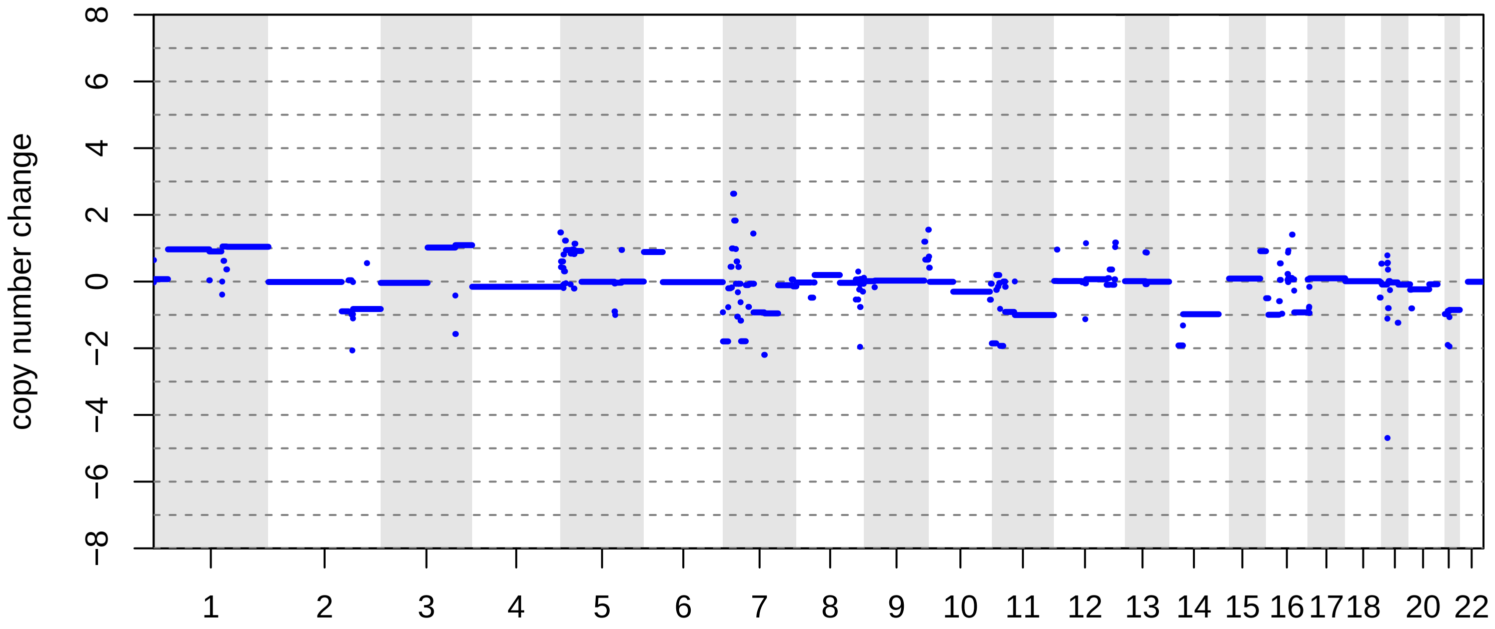

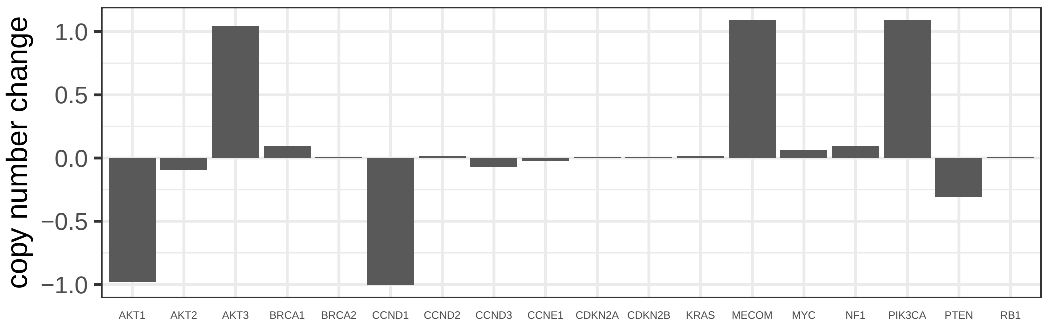

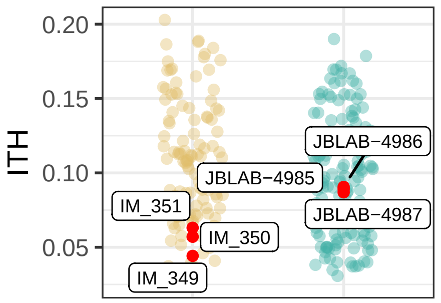

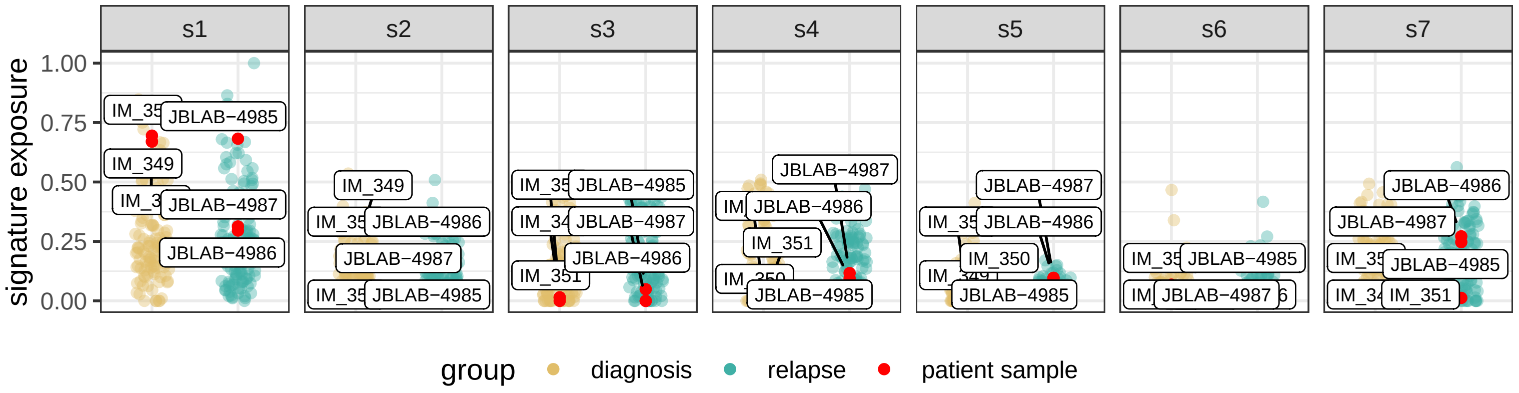

BRITROC−234

age: 63

stage: 4

platinum status: resistant

prior lines: 1

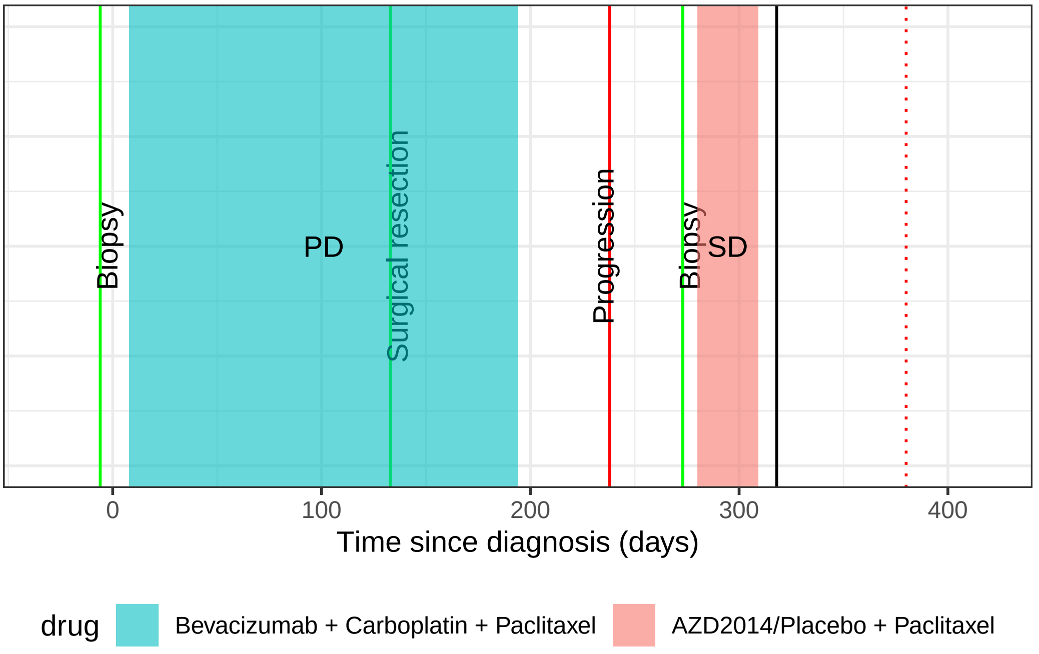

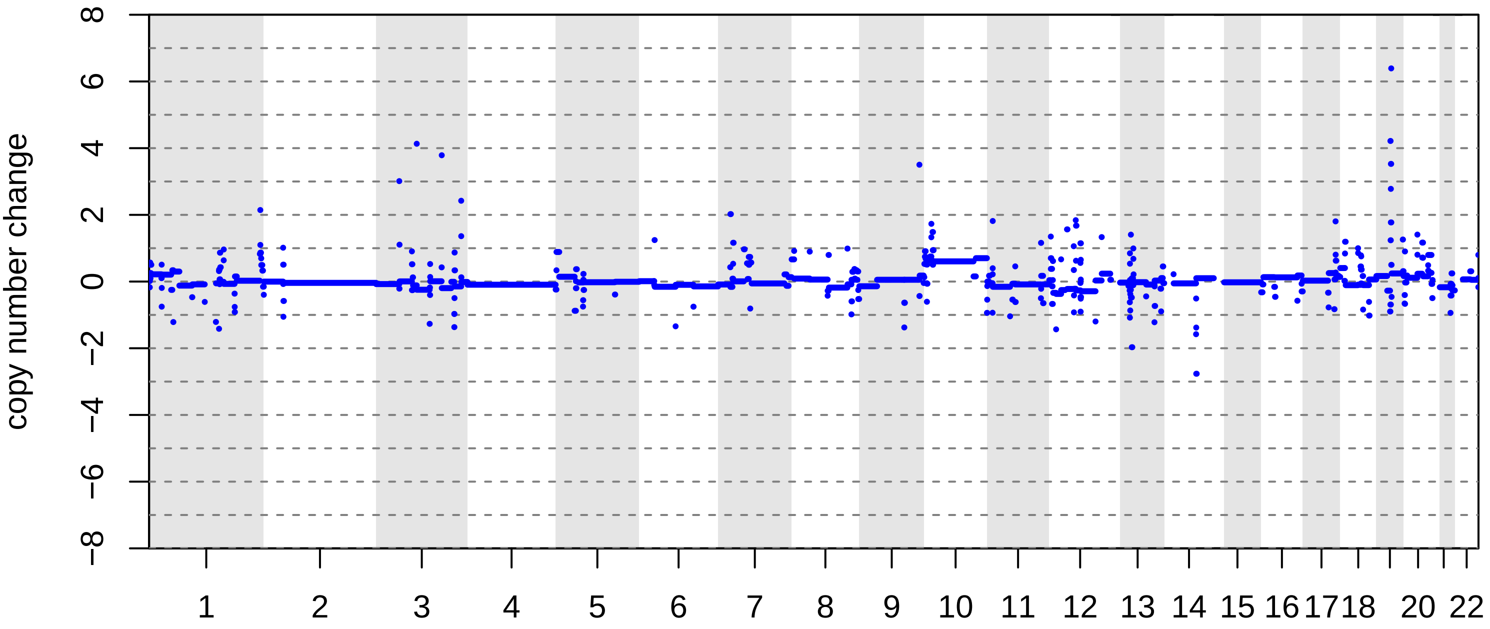

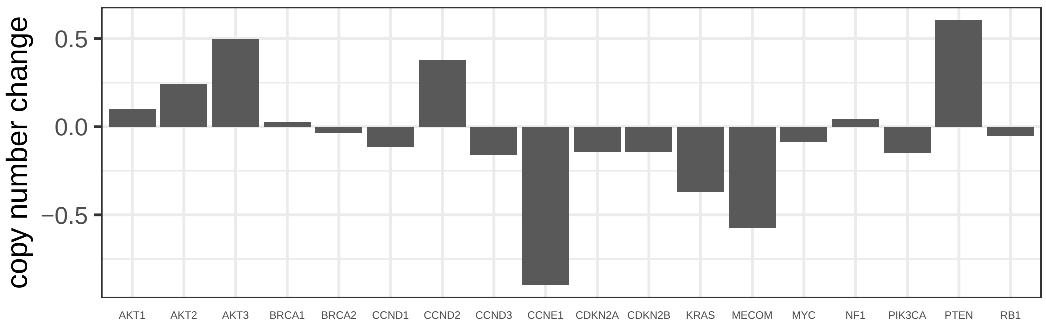

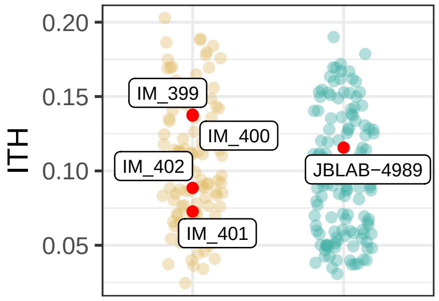

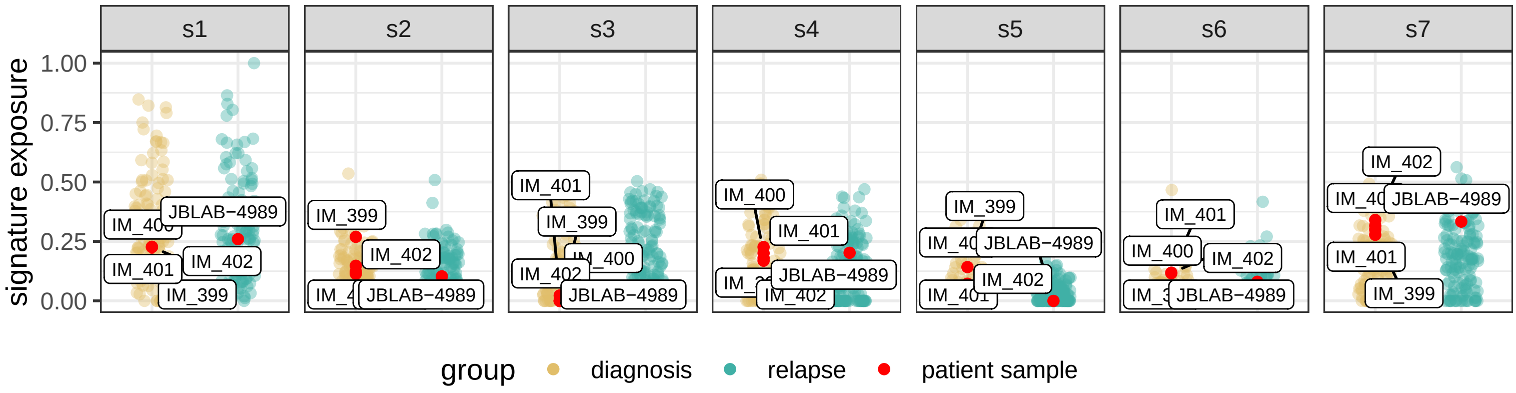

BRITROC−241

age: 53

stage: 4

platinum status: sensitive

prior lines: 3

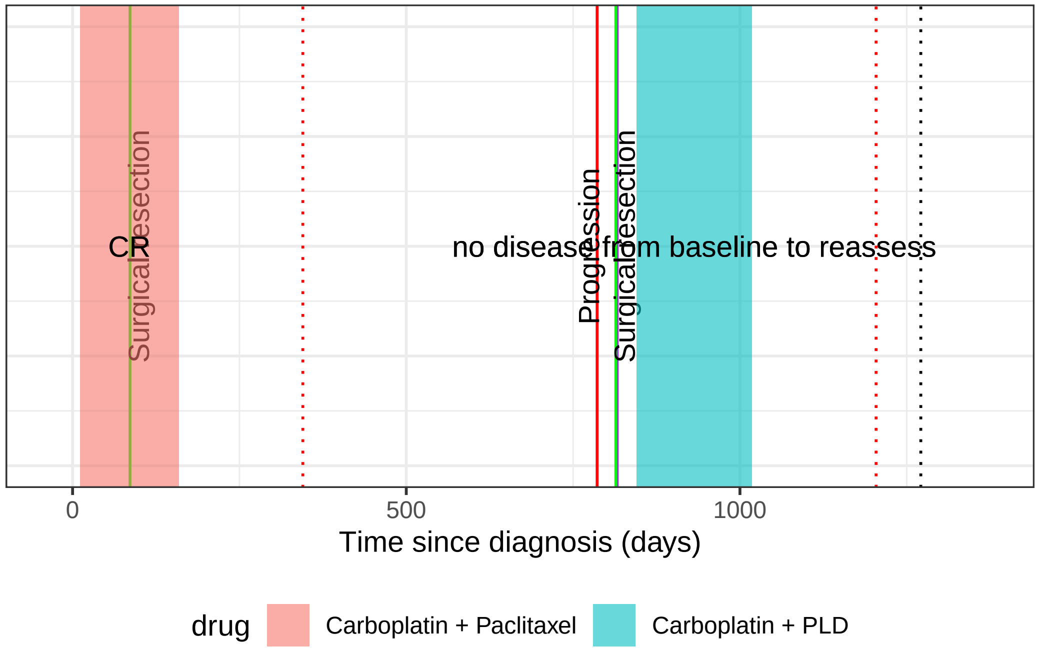

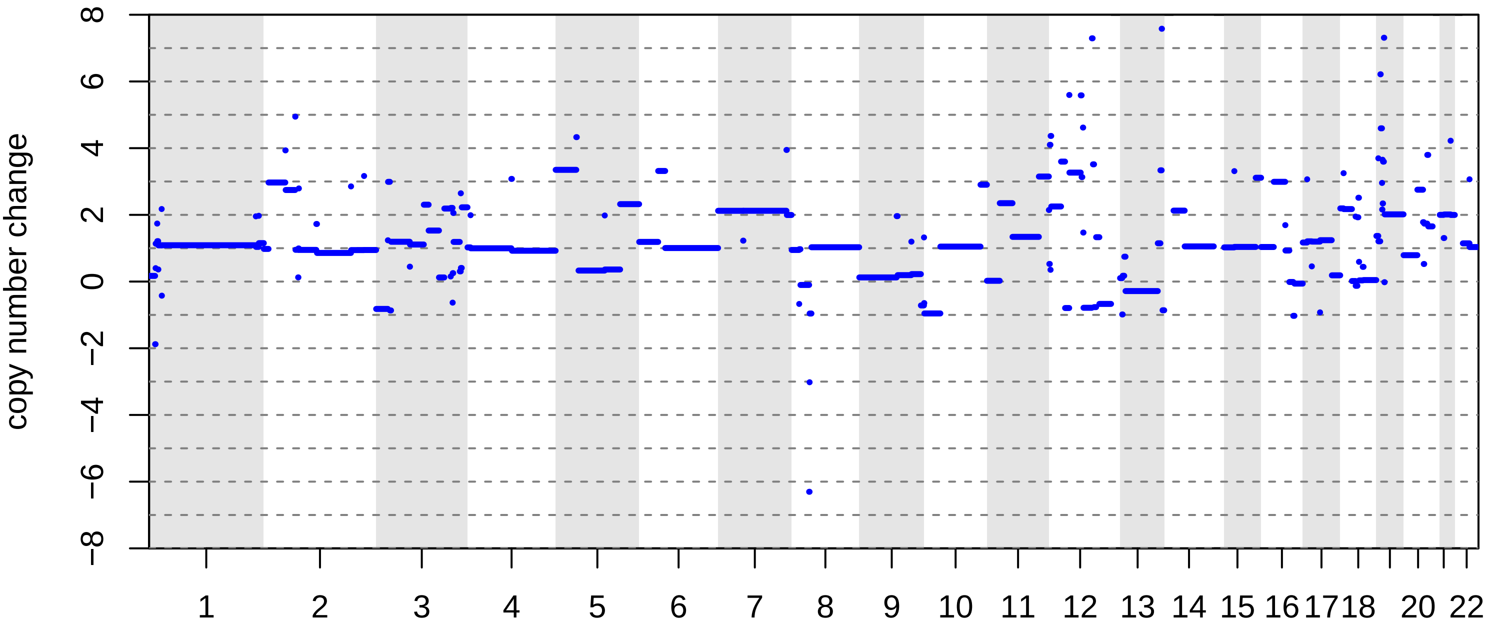

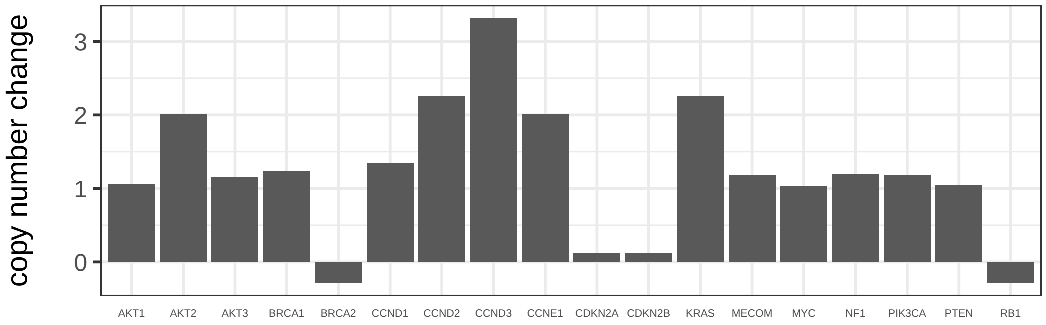

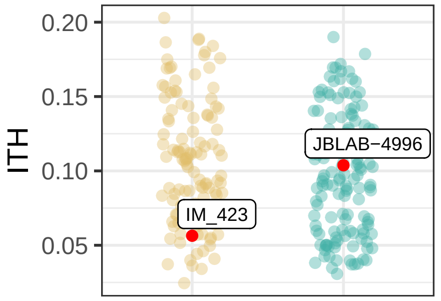

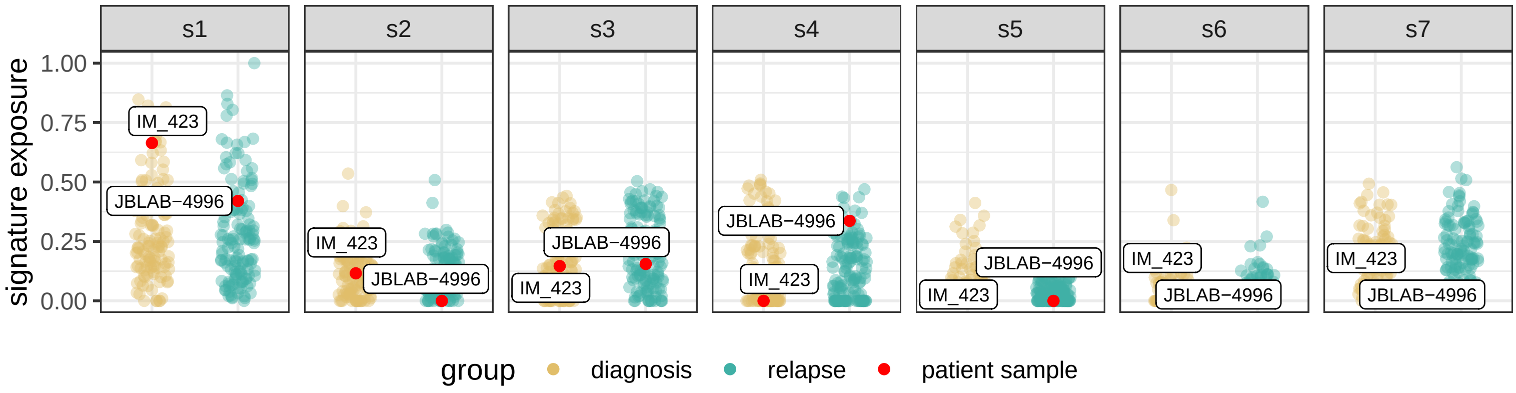

BRITROC−242

age: 78

stage: 33

platinum status: sensitive

prior lines: 1

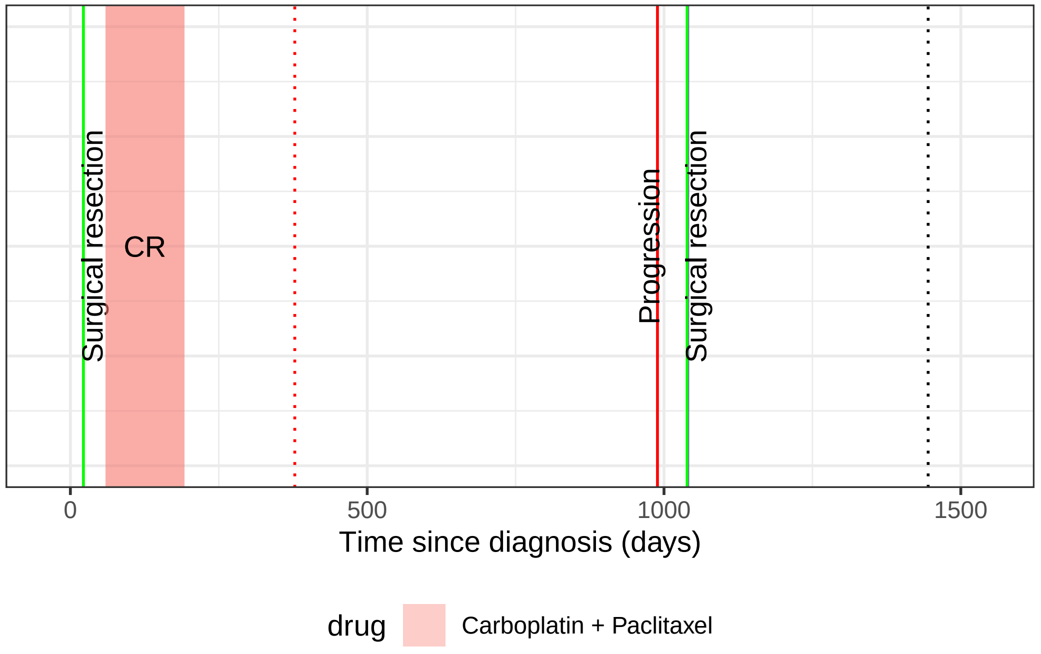

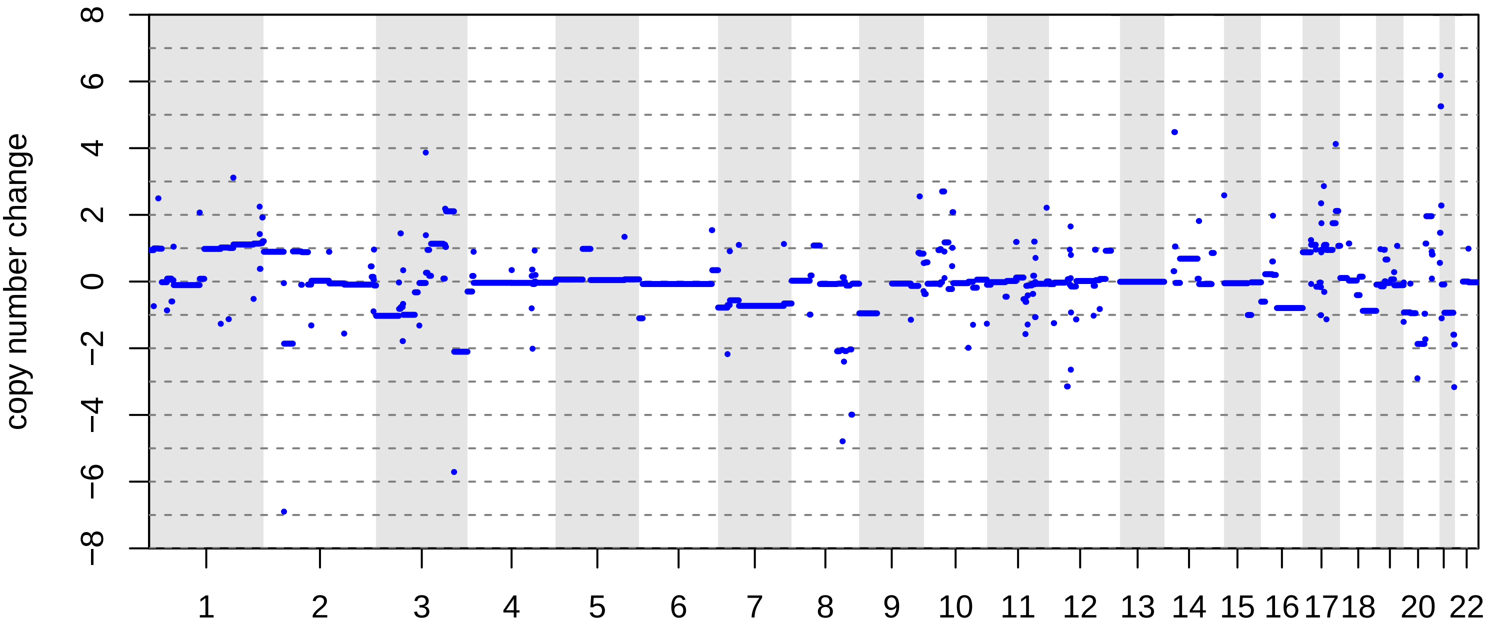

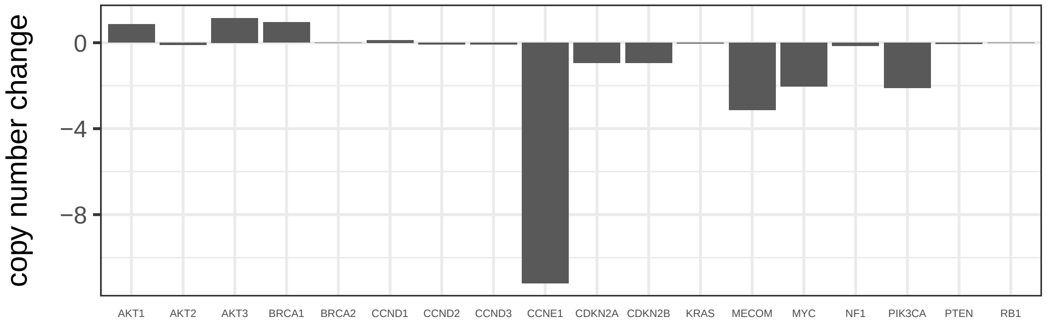

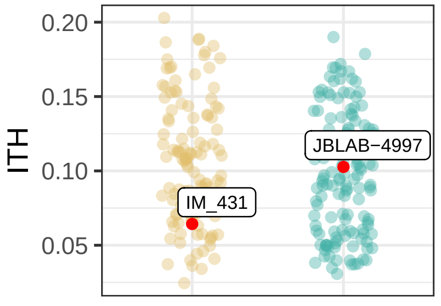

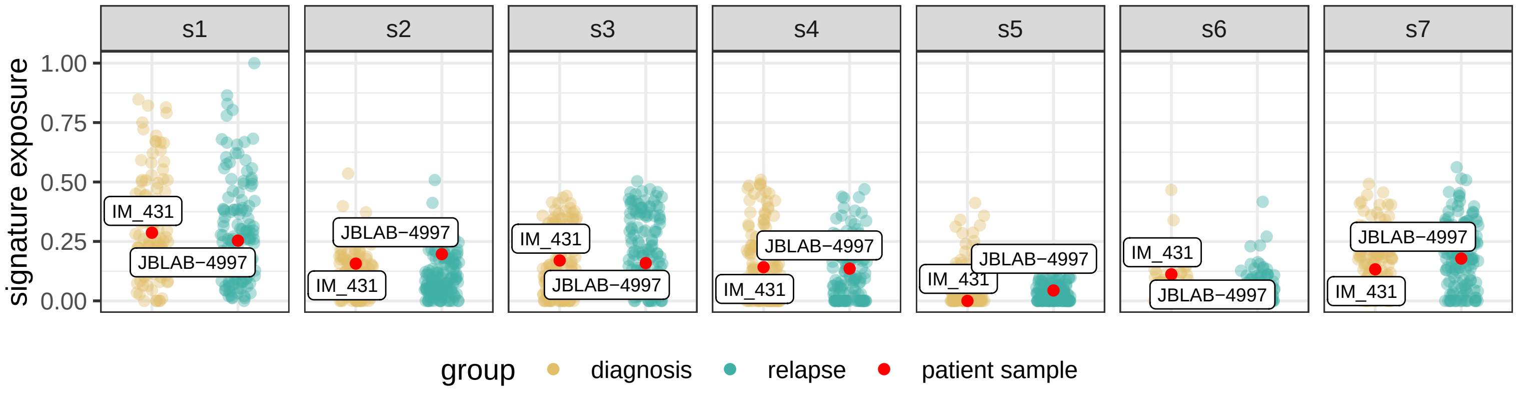

BRITROC−246

age: 61

stage: 33

platinum status: sensitive

prior lines: 1

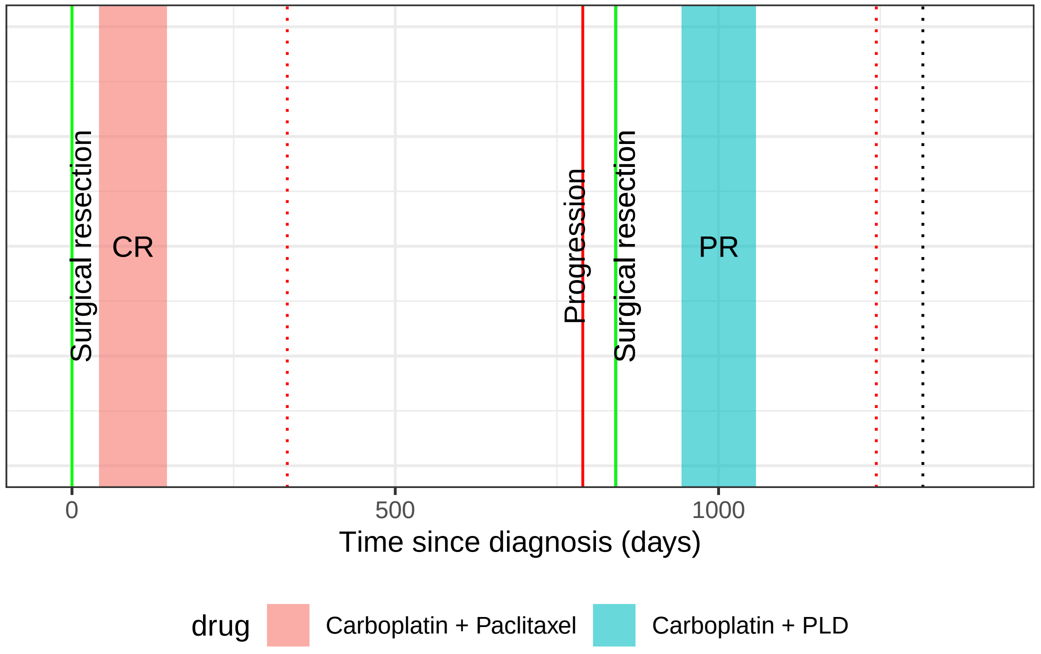

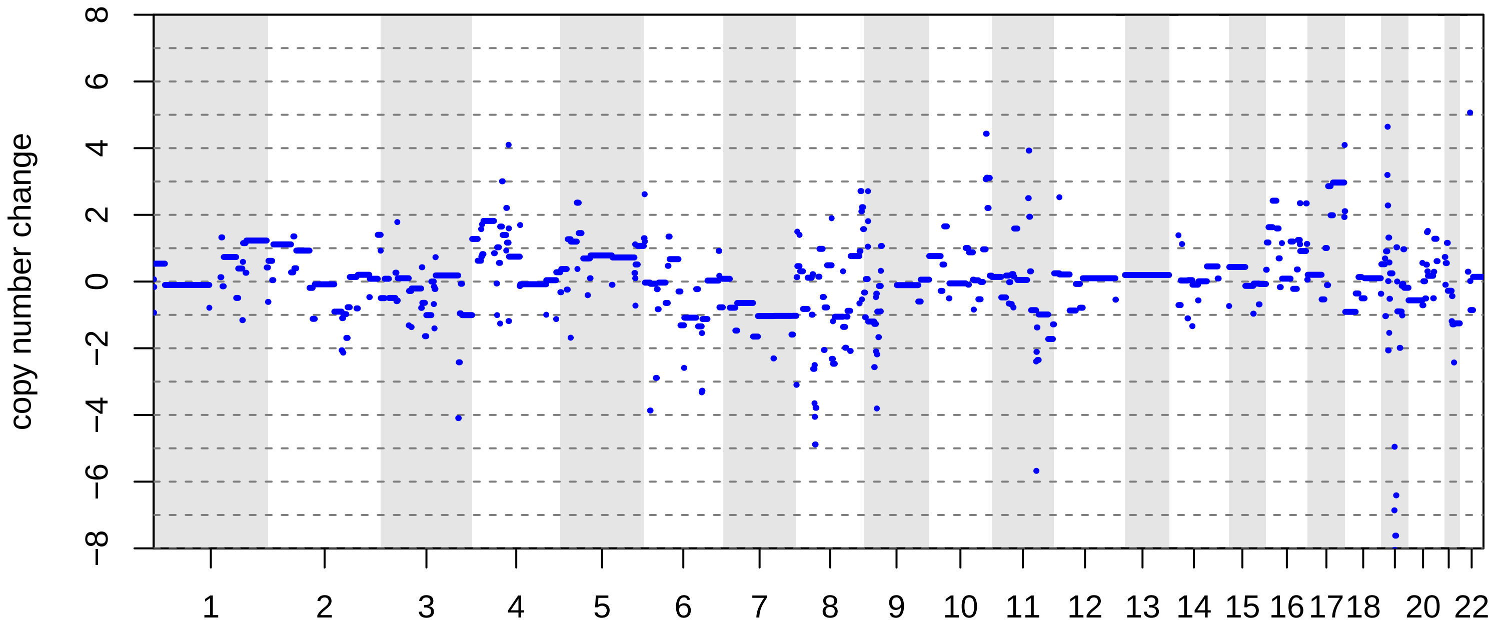

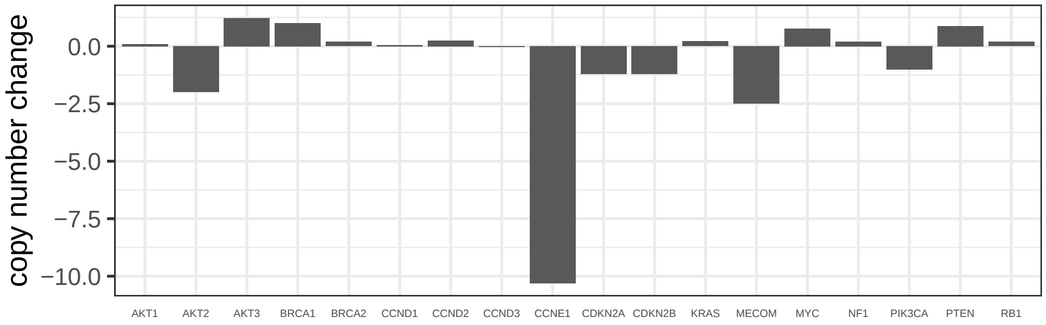

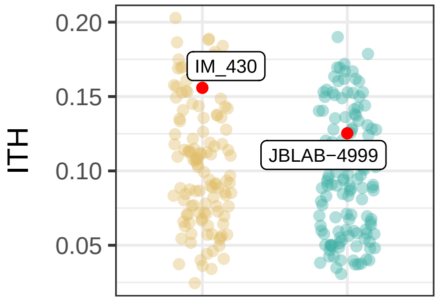

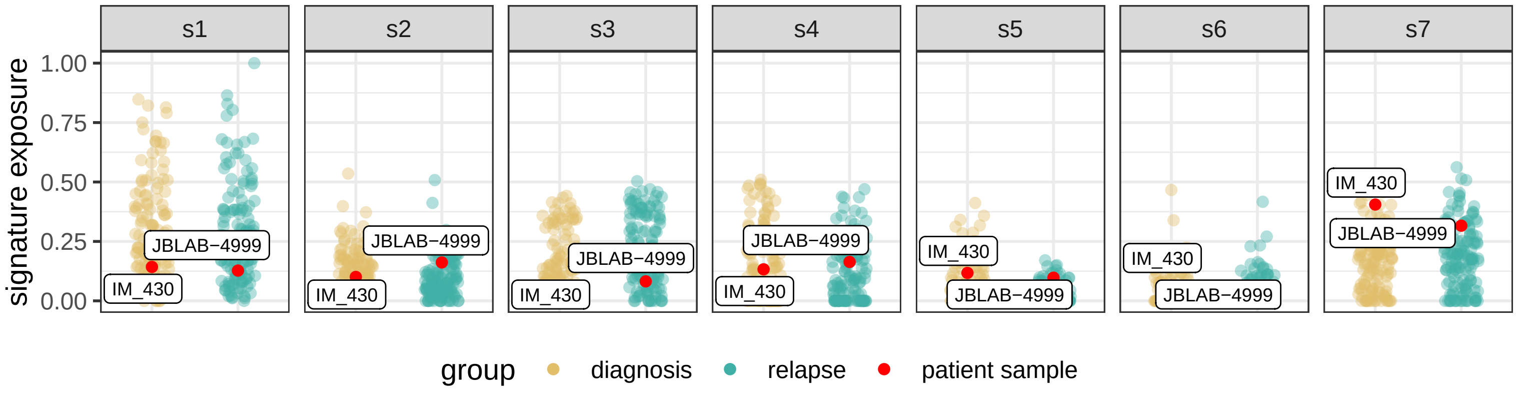

BRITROC−248

age: 56

stage: 4

platinum status: resistant

prior lines: 2

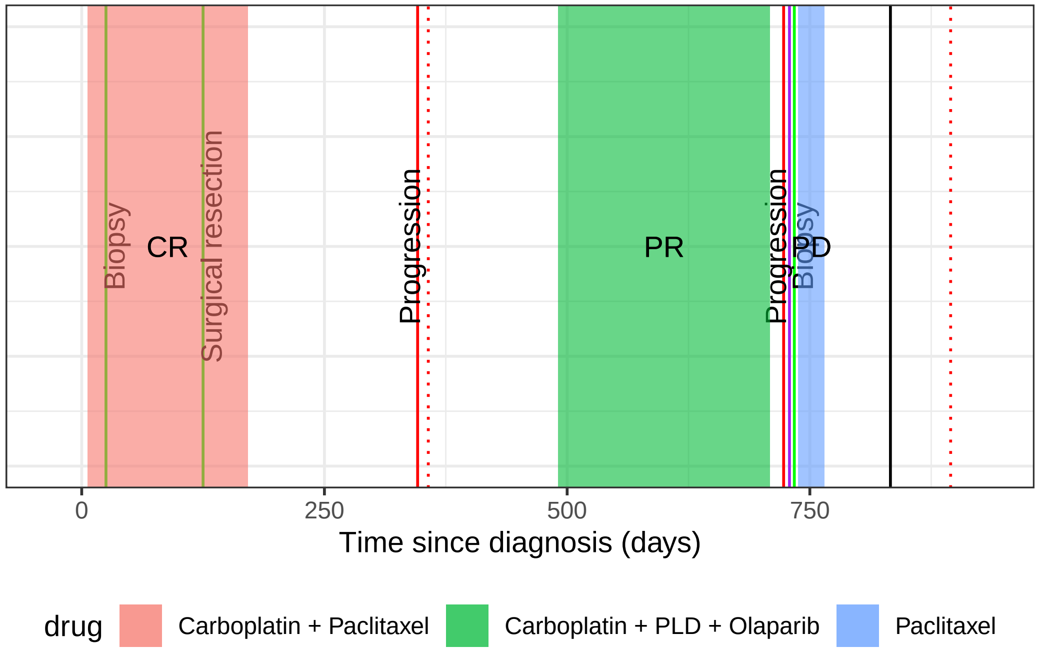

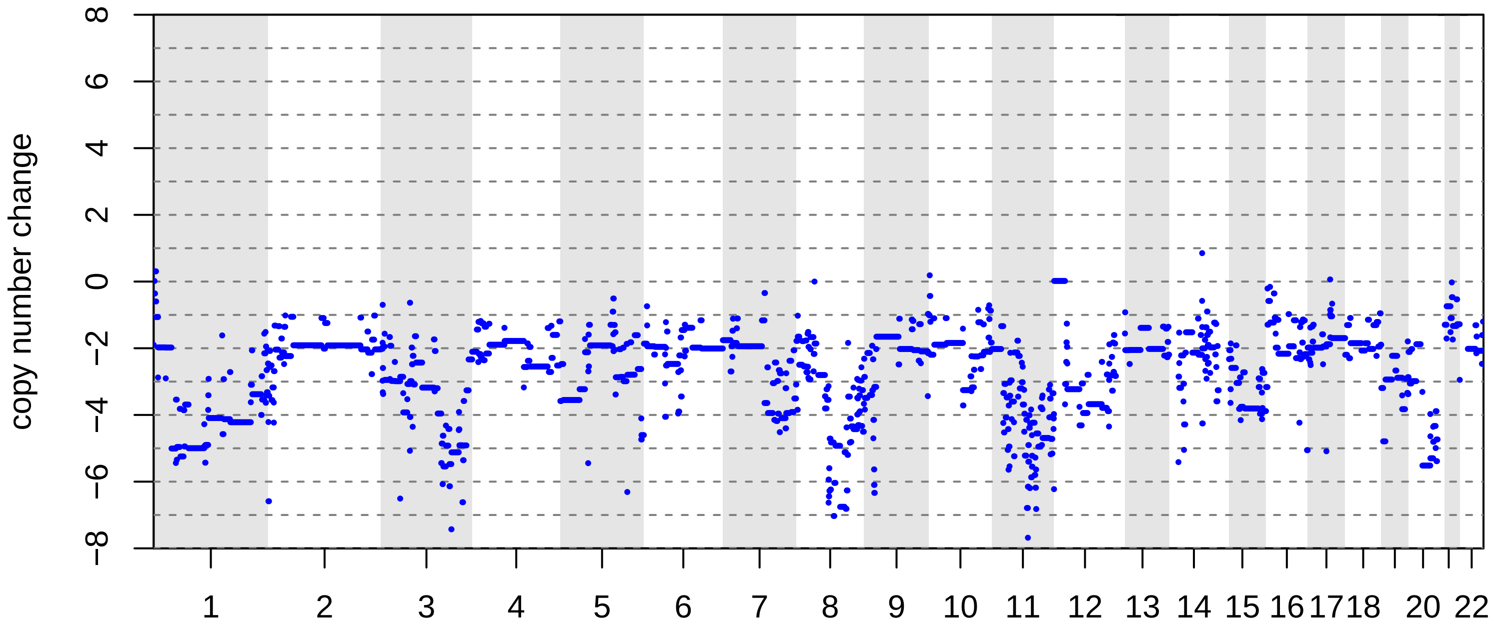

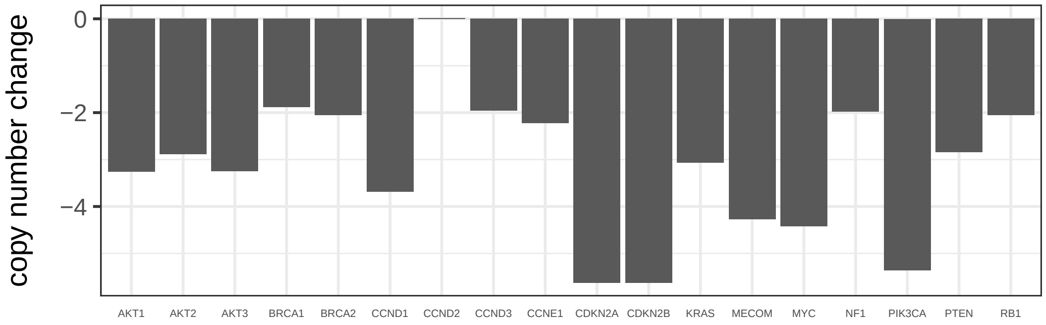

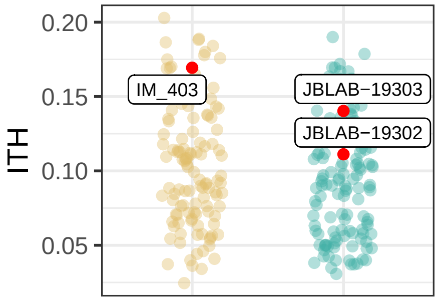

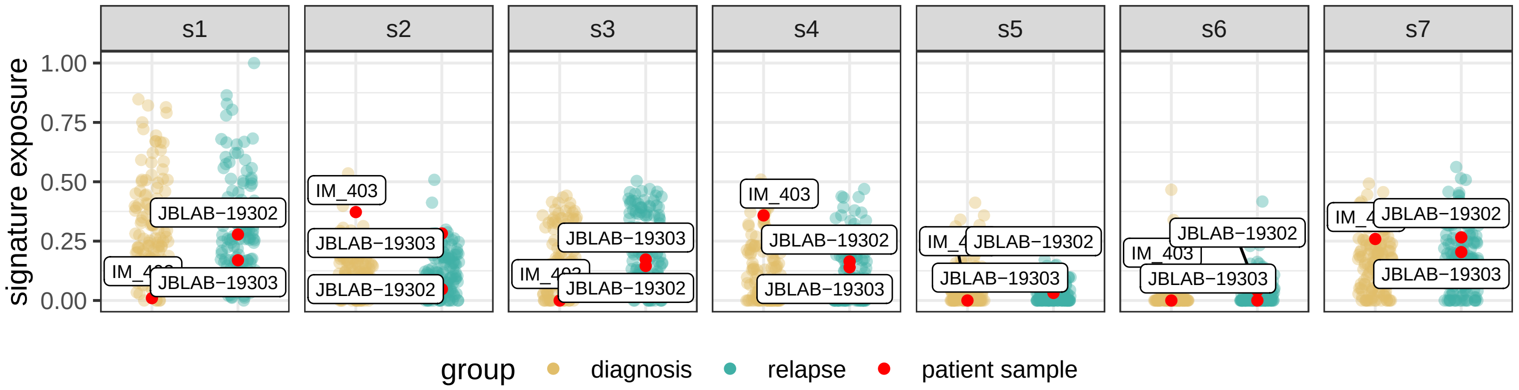

BRITROC−256

age: 63

stage: 33

platinum status: sensitive

prior lines: 2

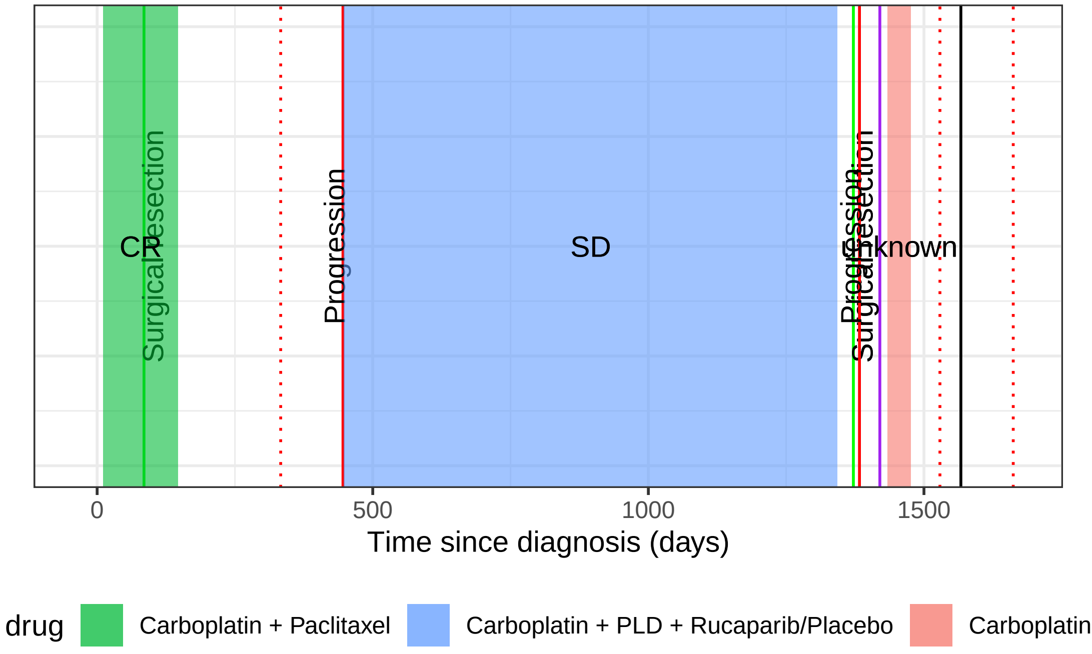

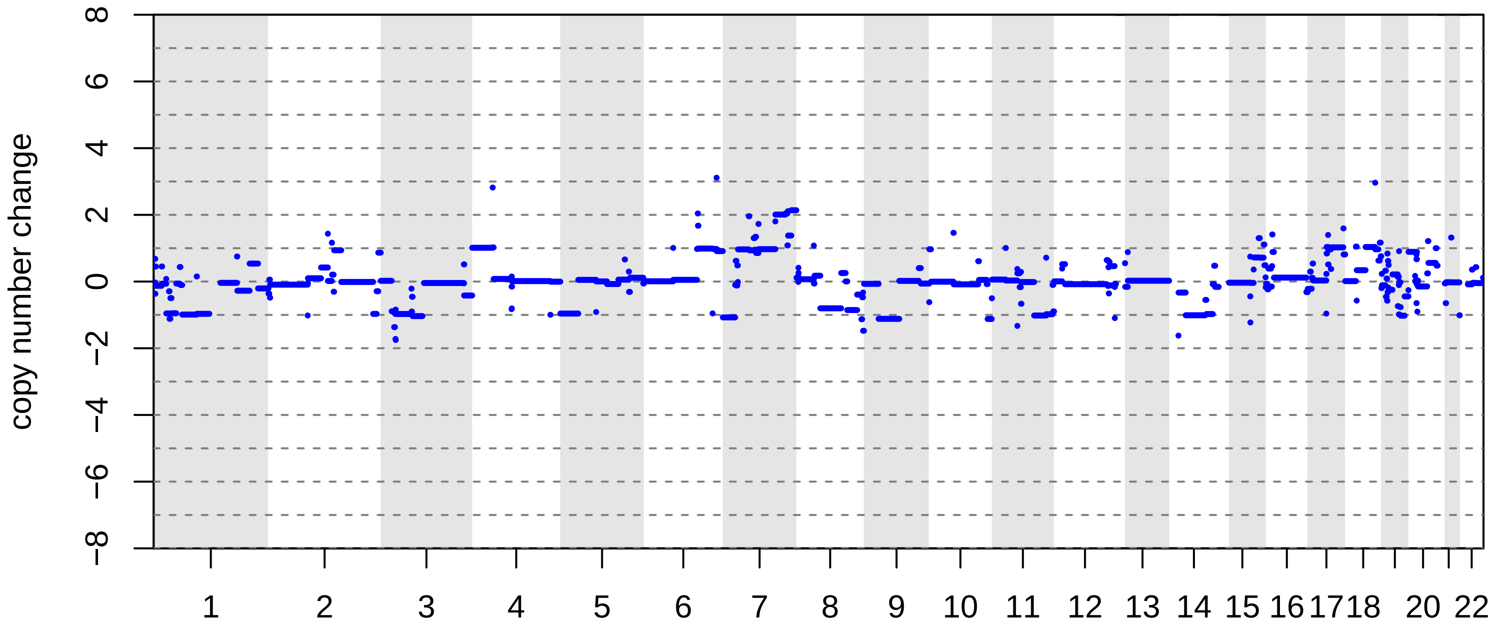

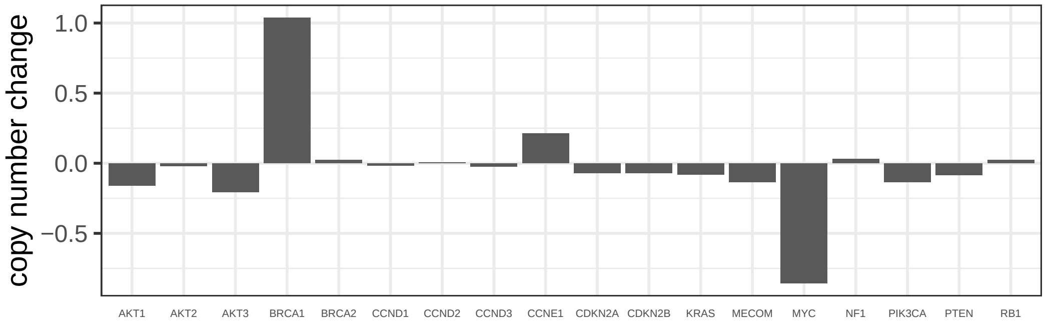

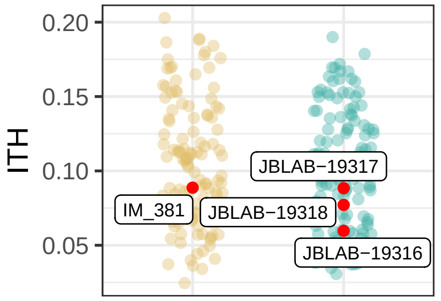

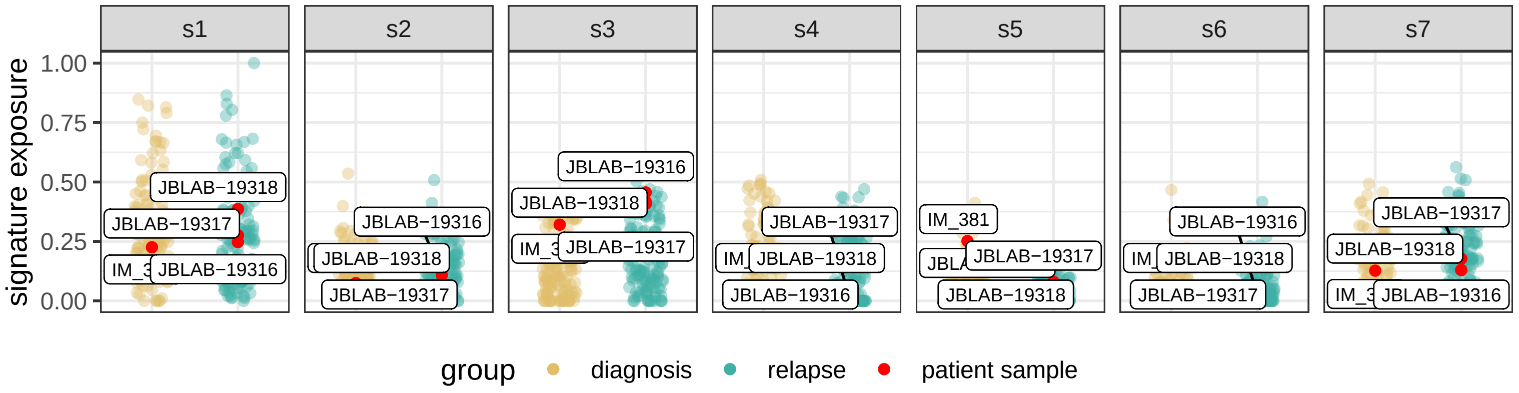

BRITROC−259

age: 52

stage: 31

platinum status: sensitive

prior lines: 2

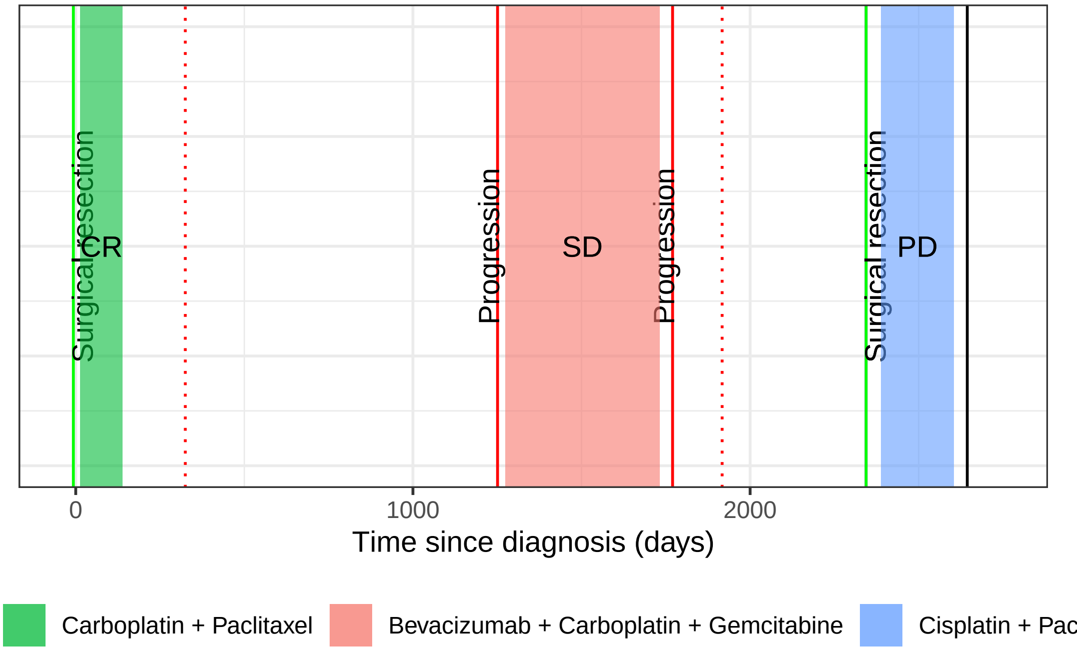

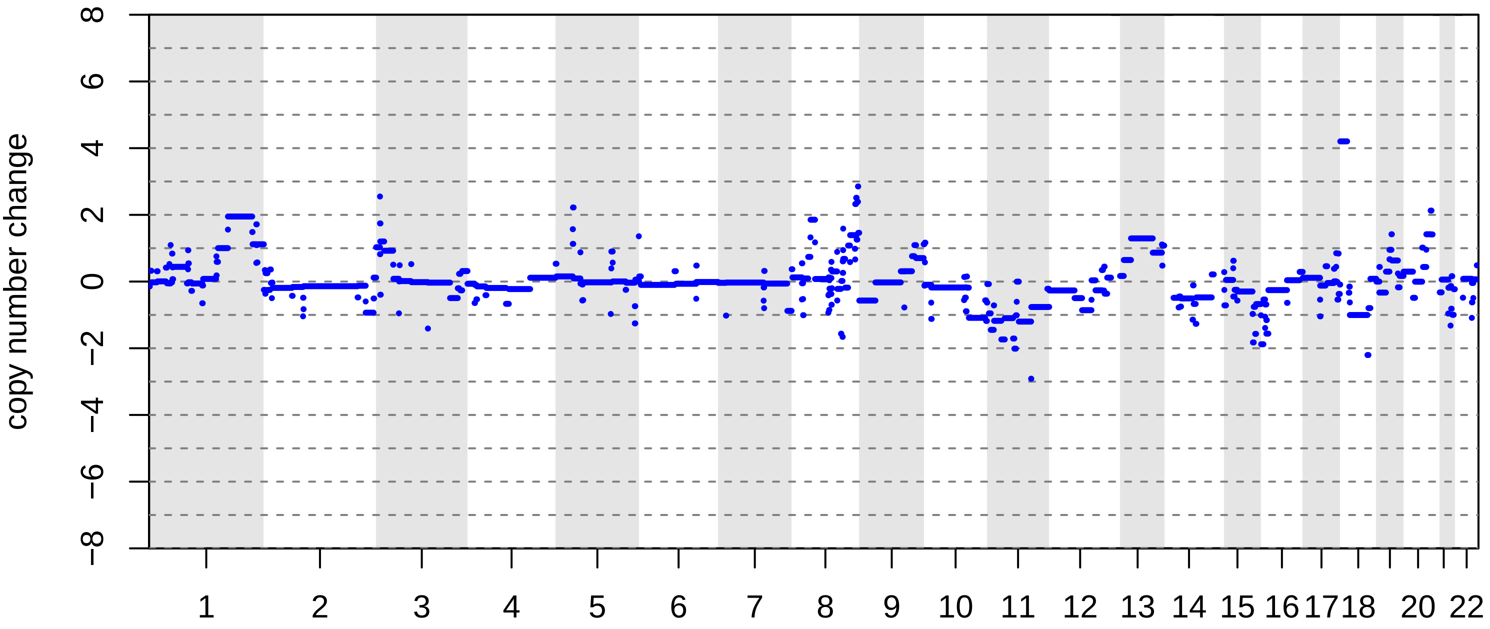

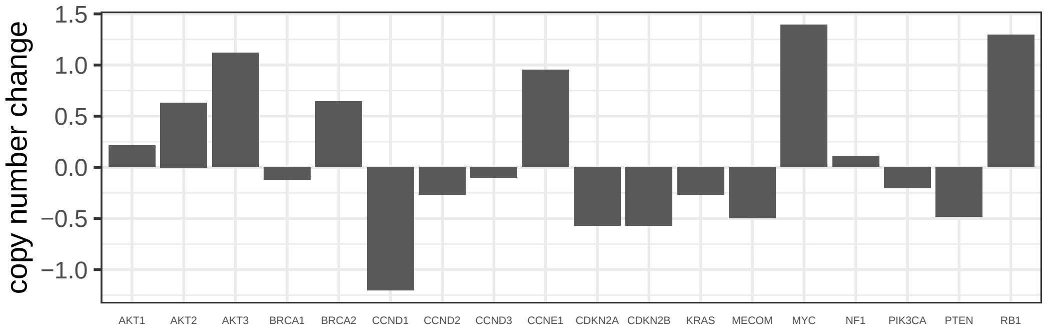

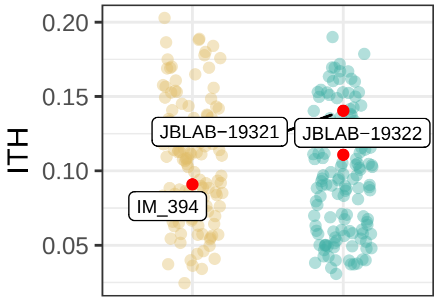

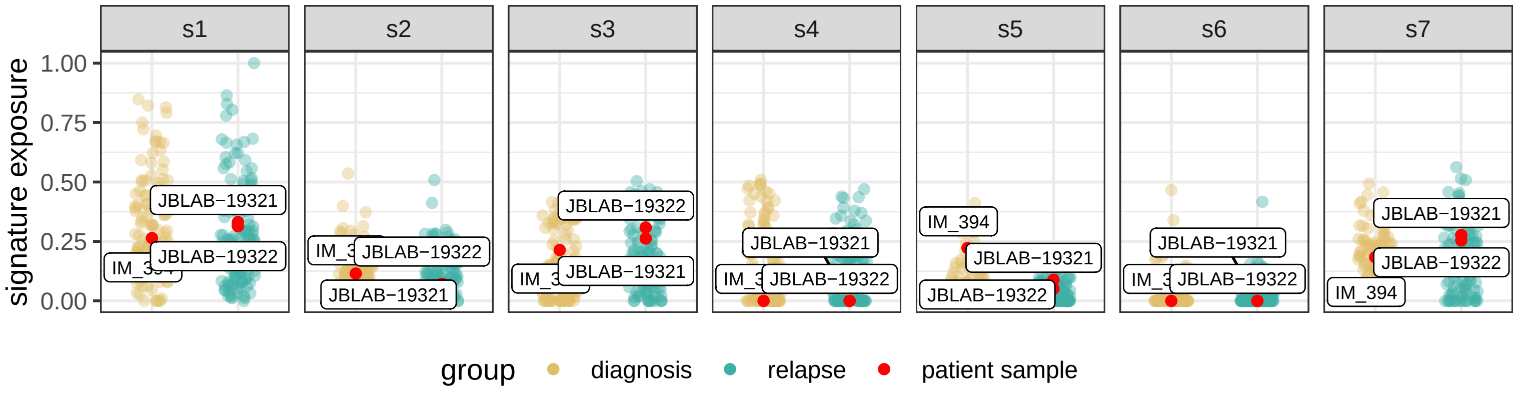

BRITROC−260

age: 73

stage: 21

platinum status: sensitive

prior lines: 1

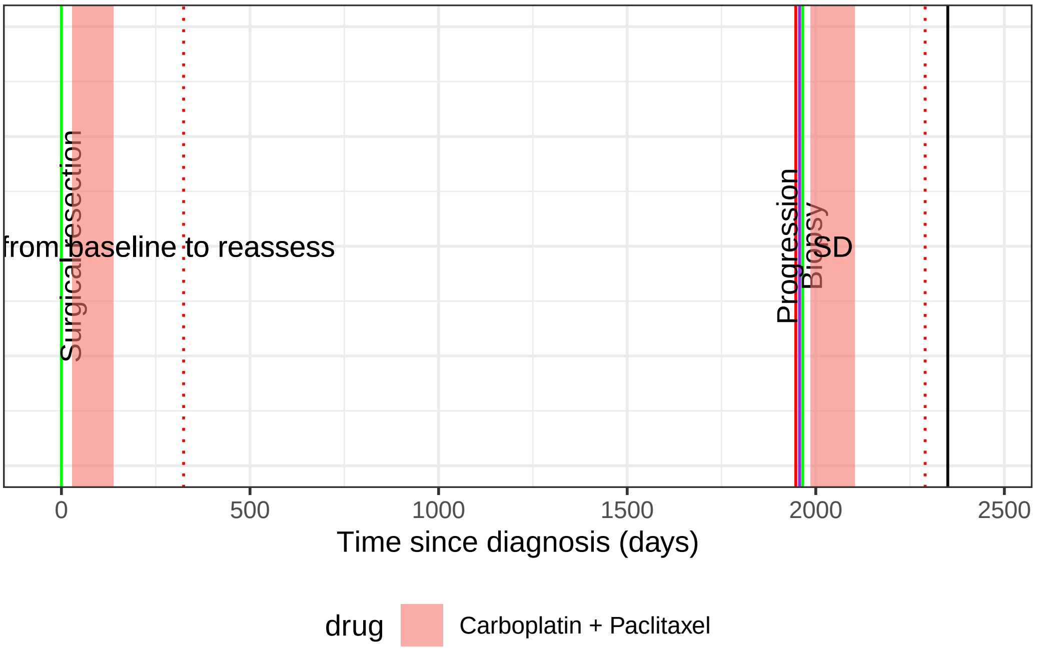

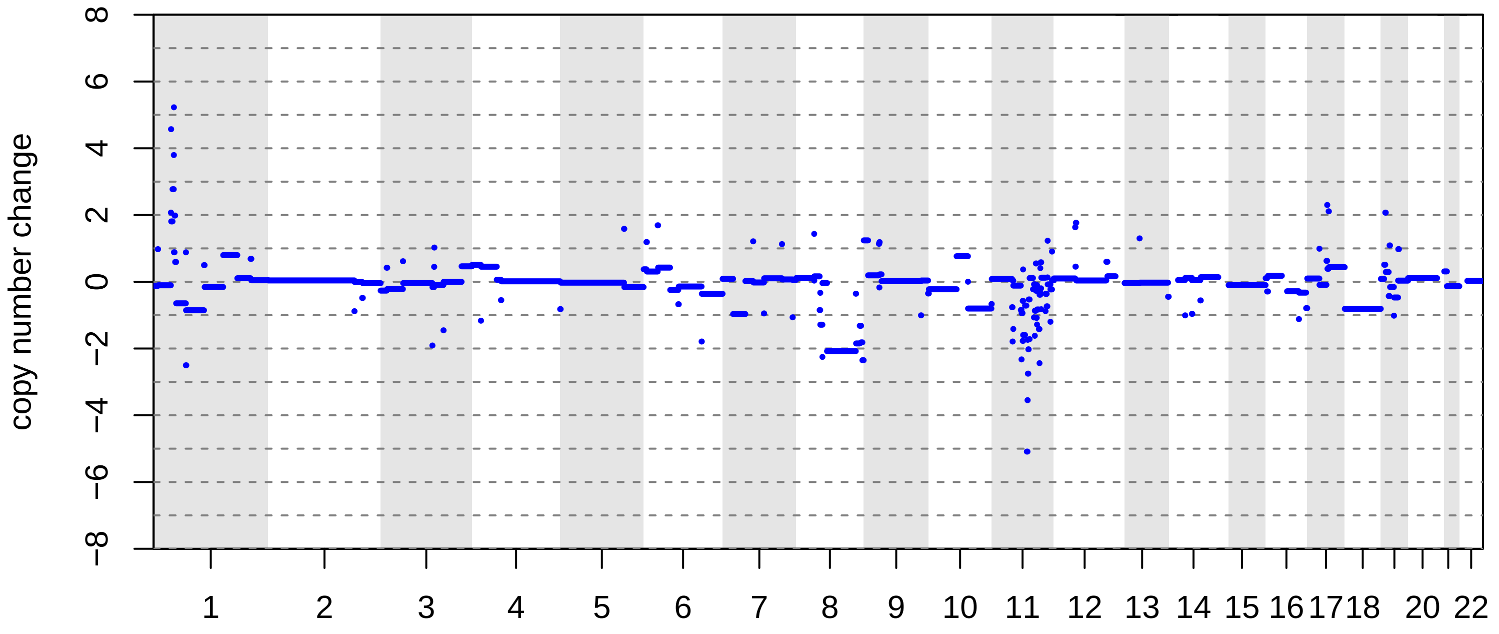

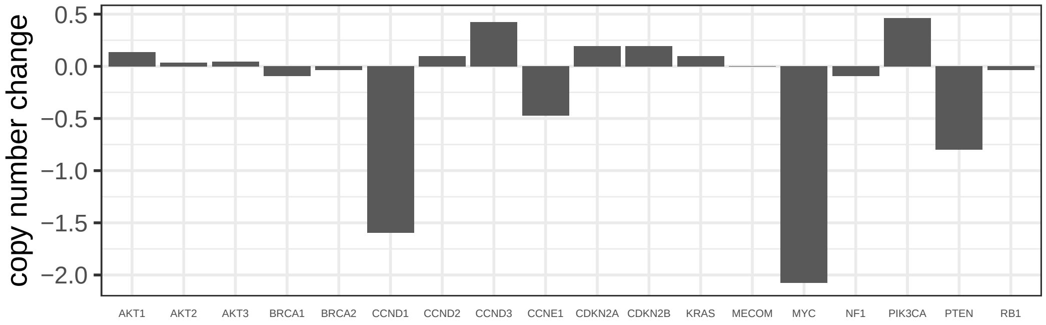

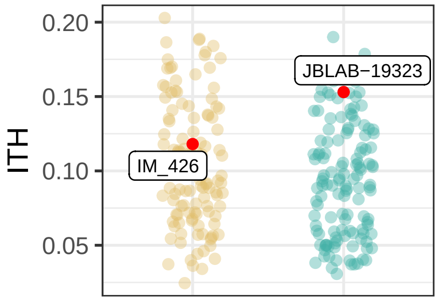

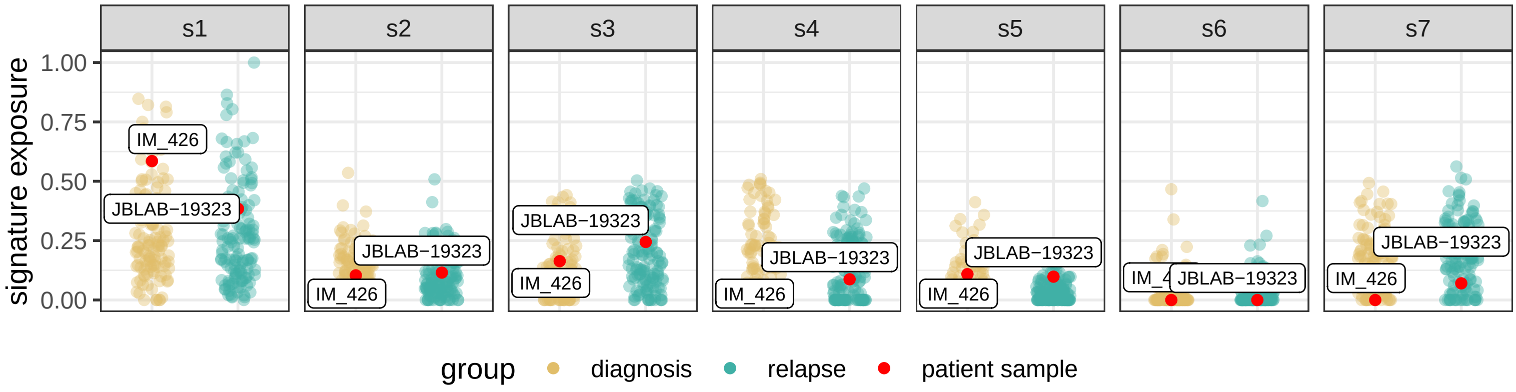

BRITROC−267

age: 57

stage: 32

platinum status: sensitive

prior lines: 1

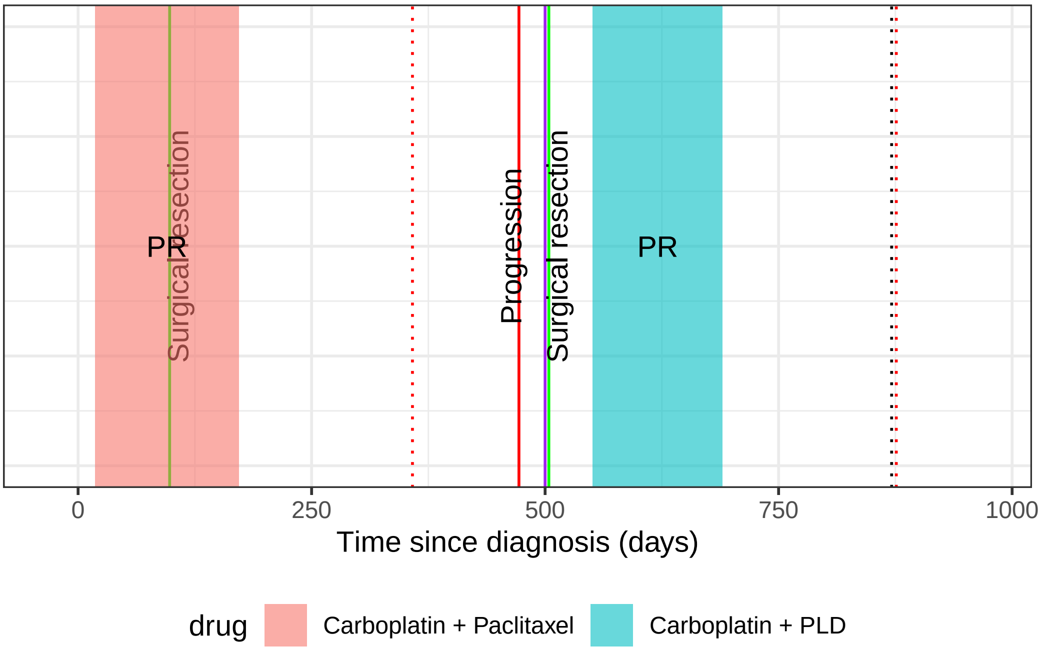

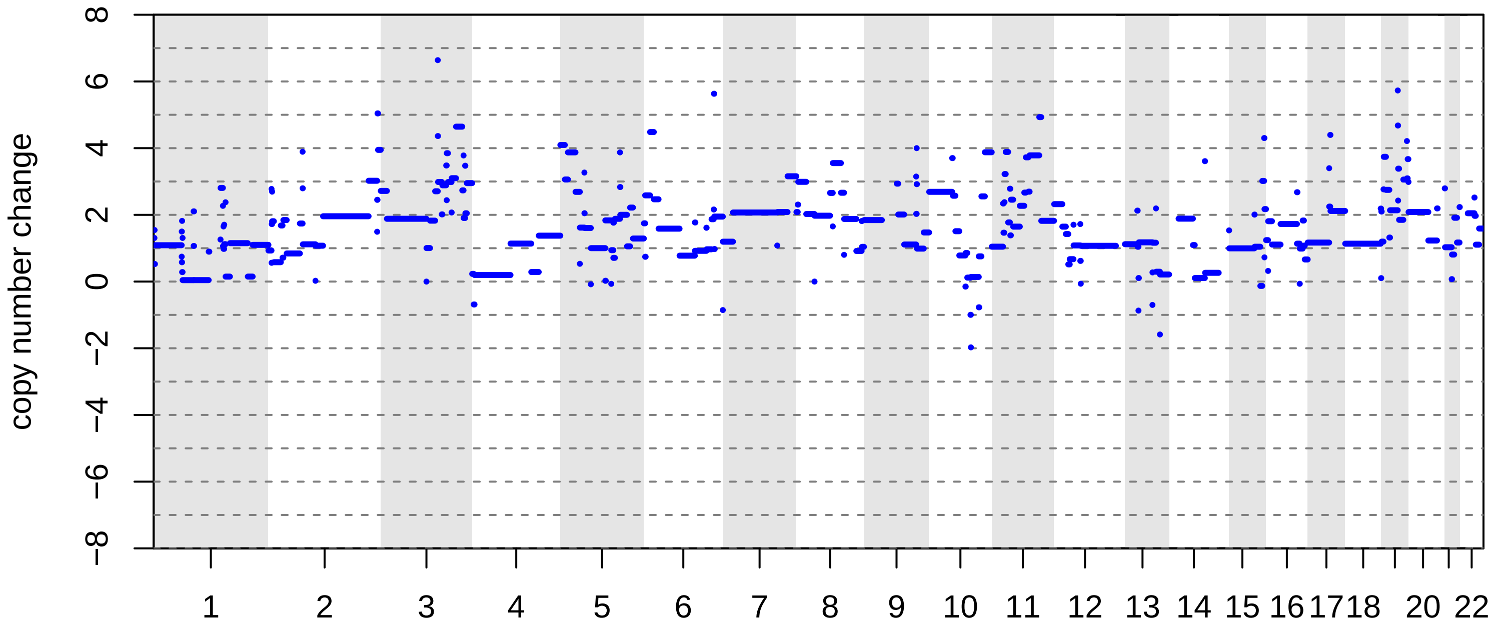

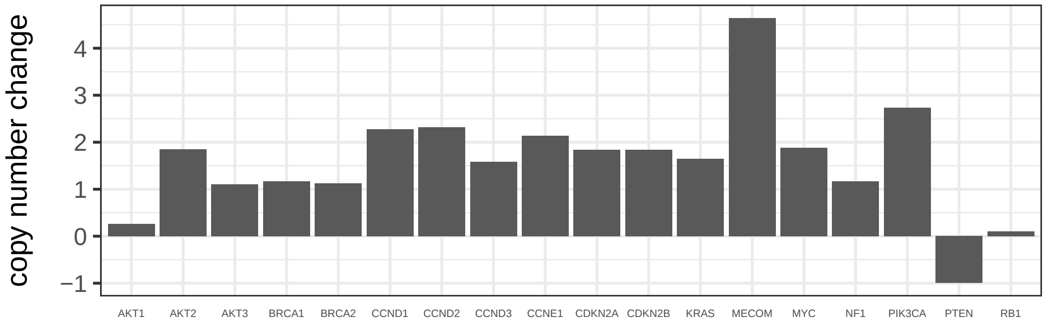

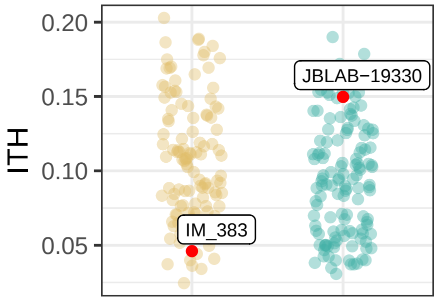

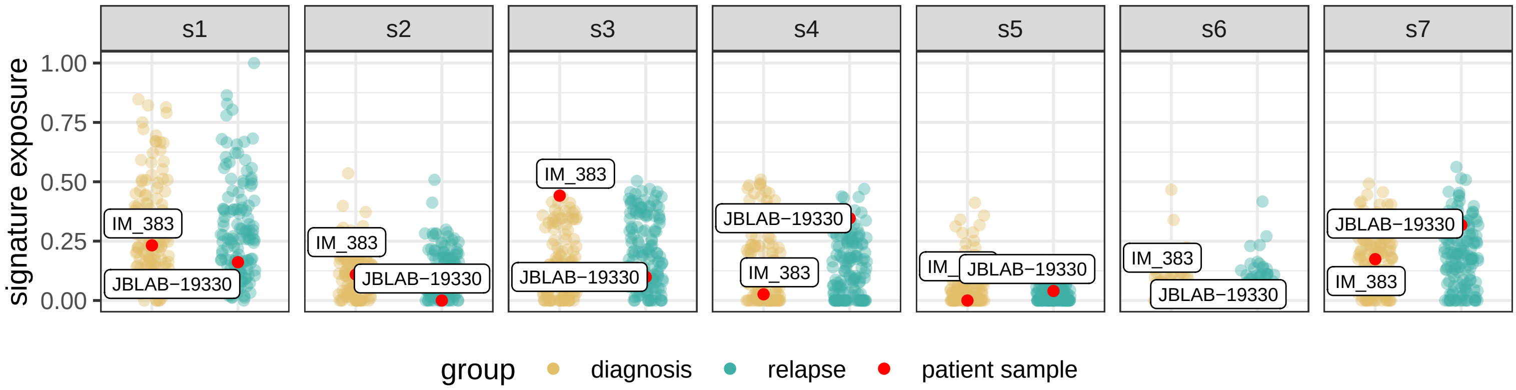

BRITROC−268

age: 60

stage: 33

platinum status: resistant

prior lines: 2

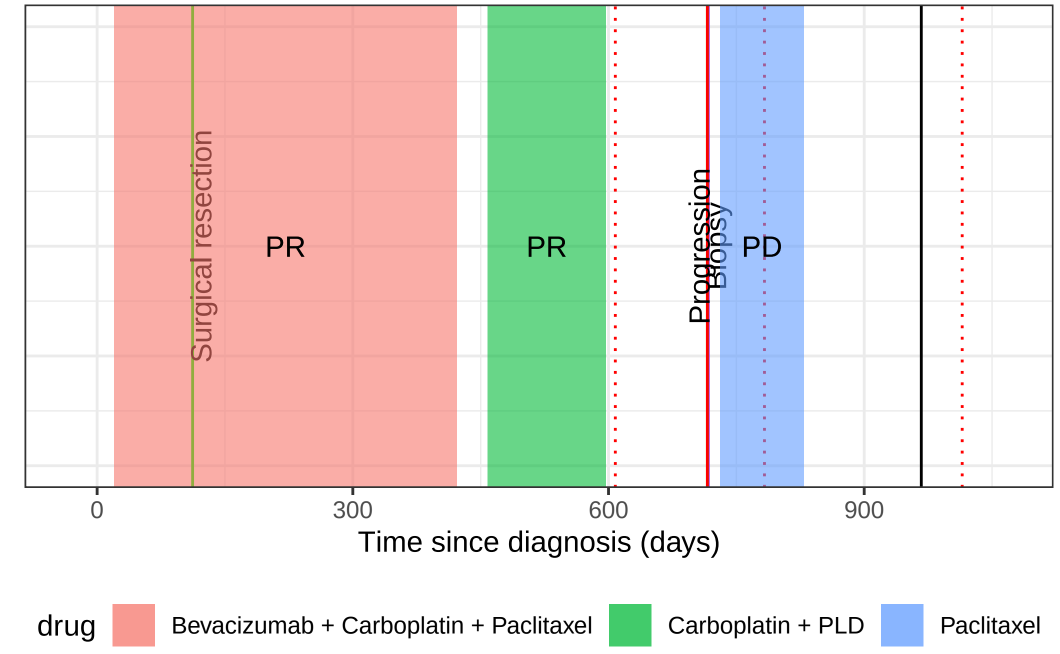

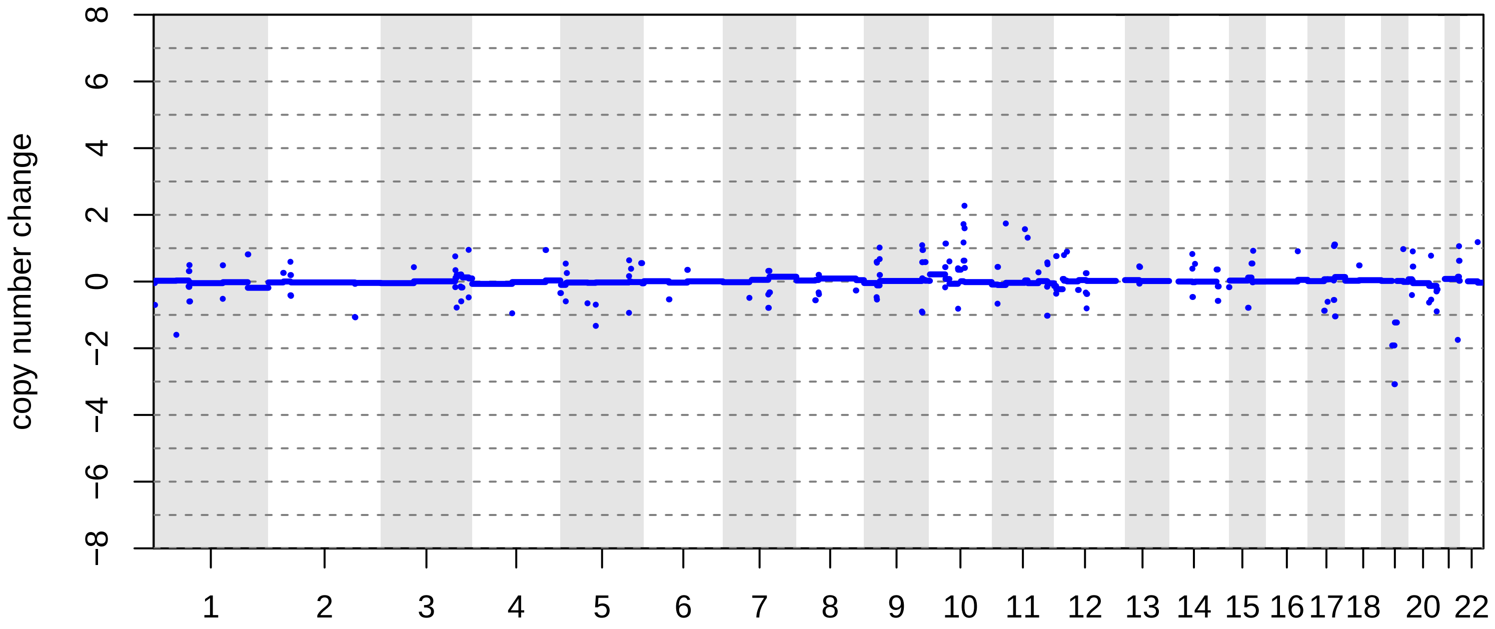

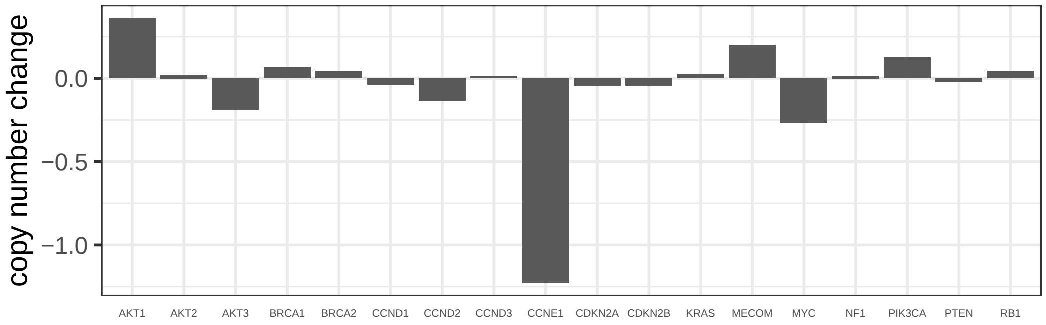

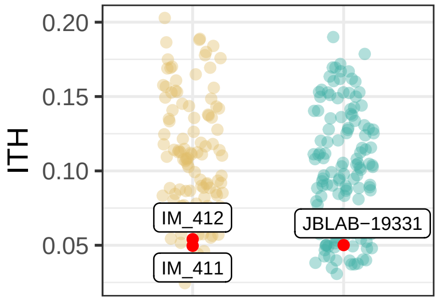

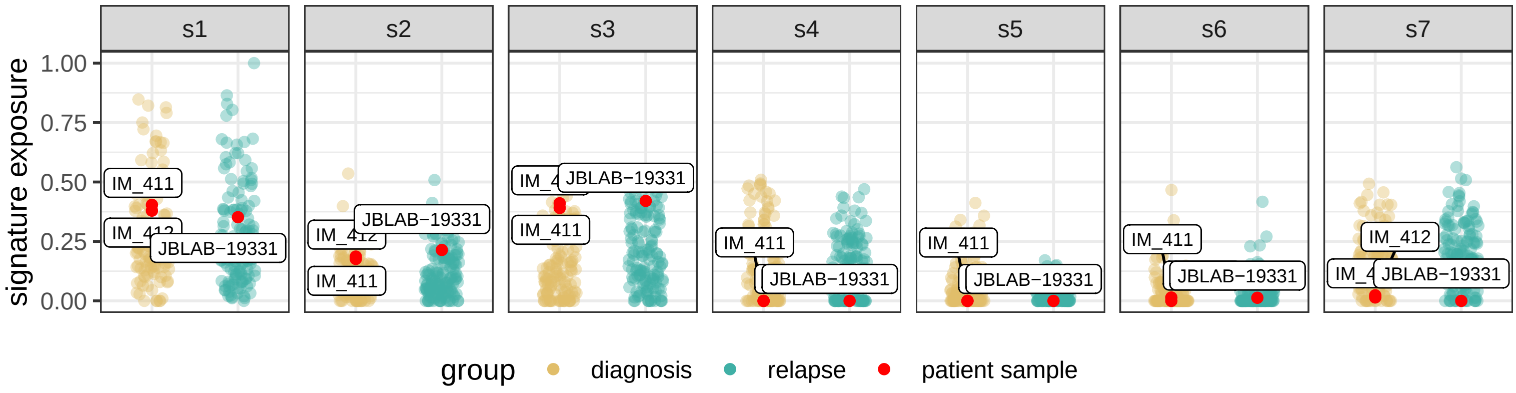

BRITROC−271

age: 70

stage: 23

platinum status: sensitive

prior lines: 1

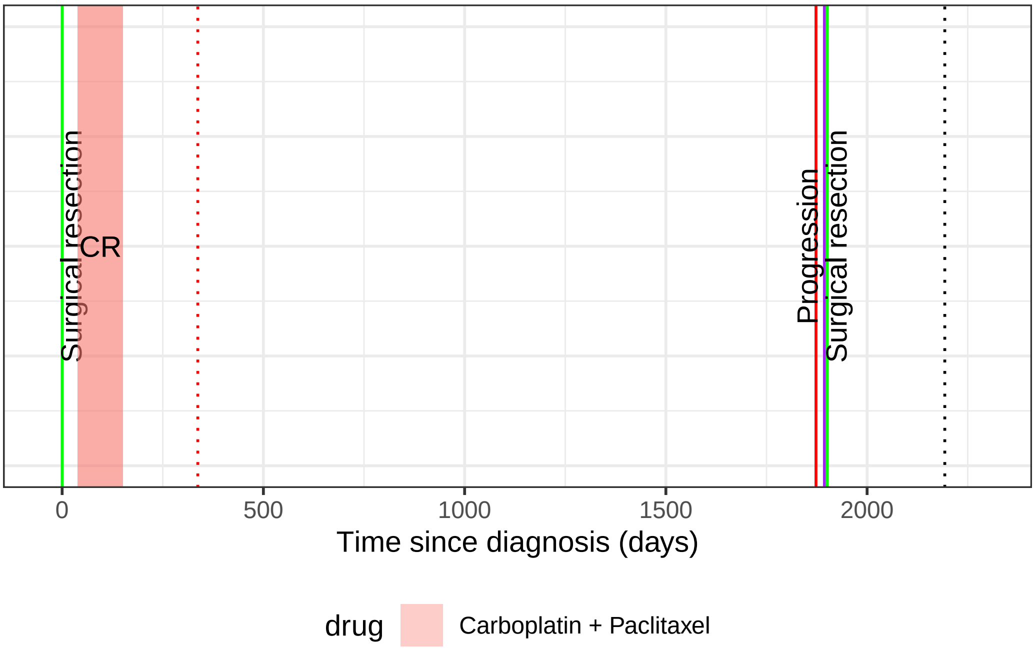

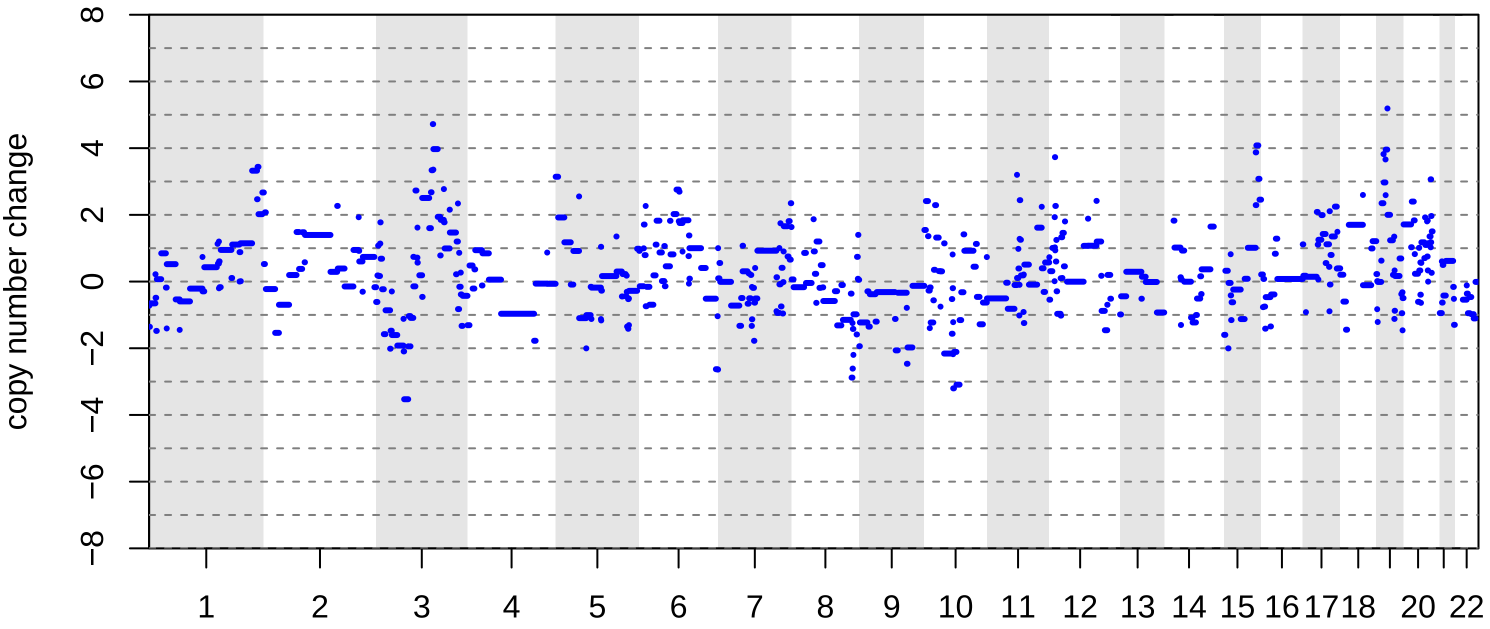

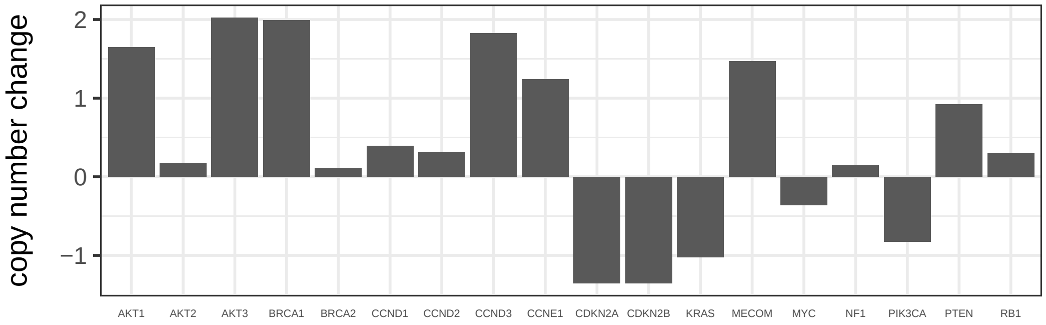

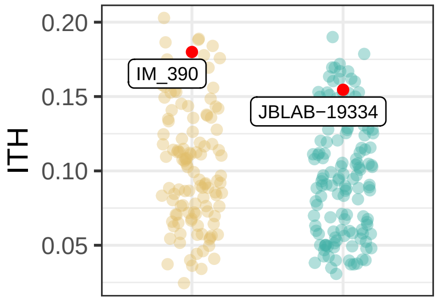

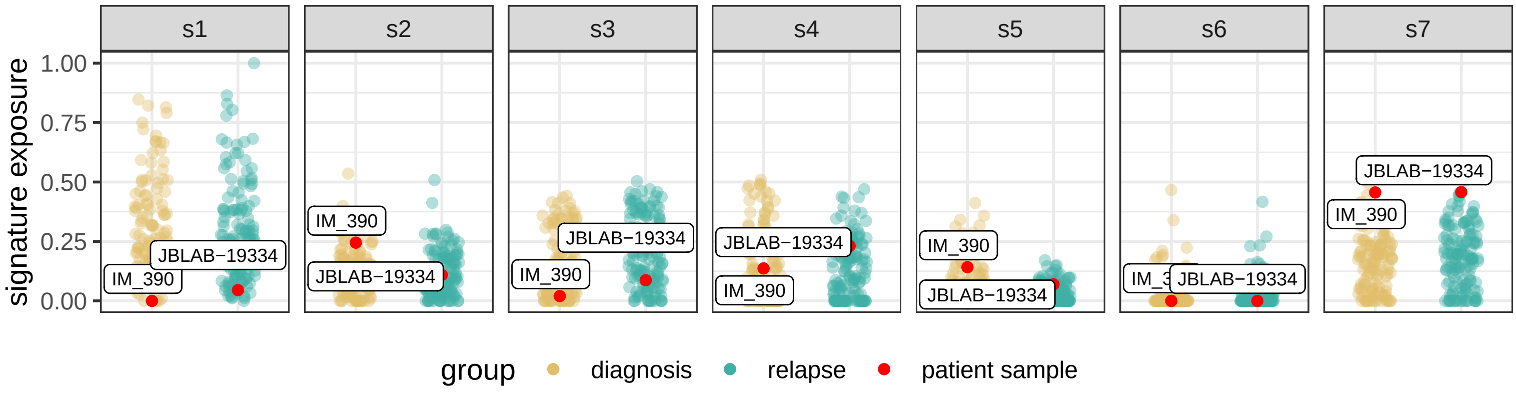

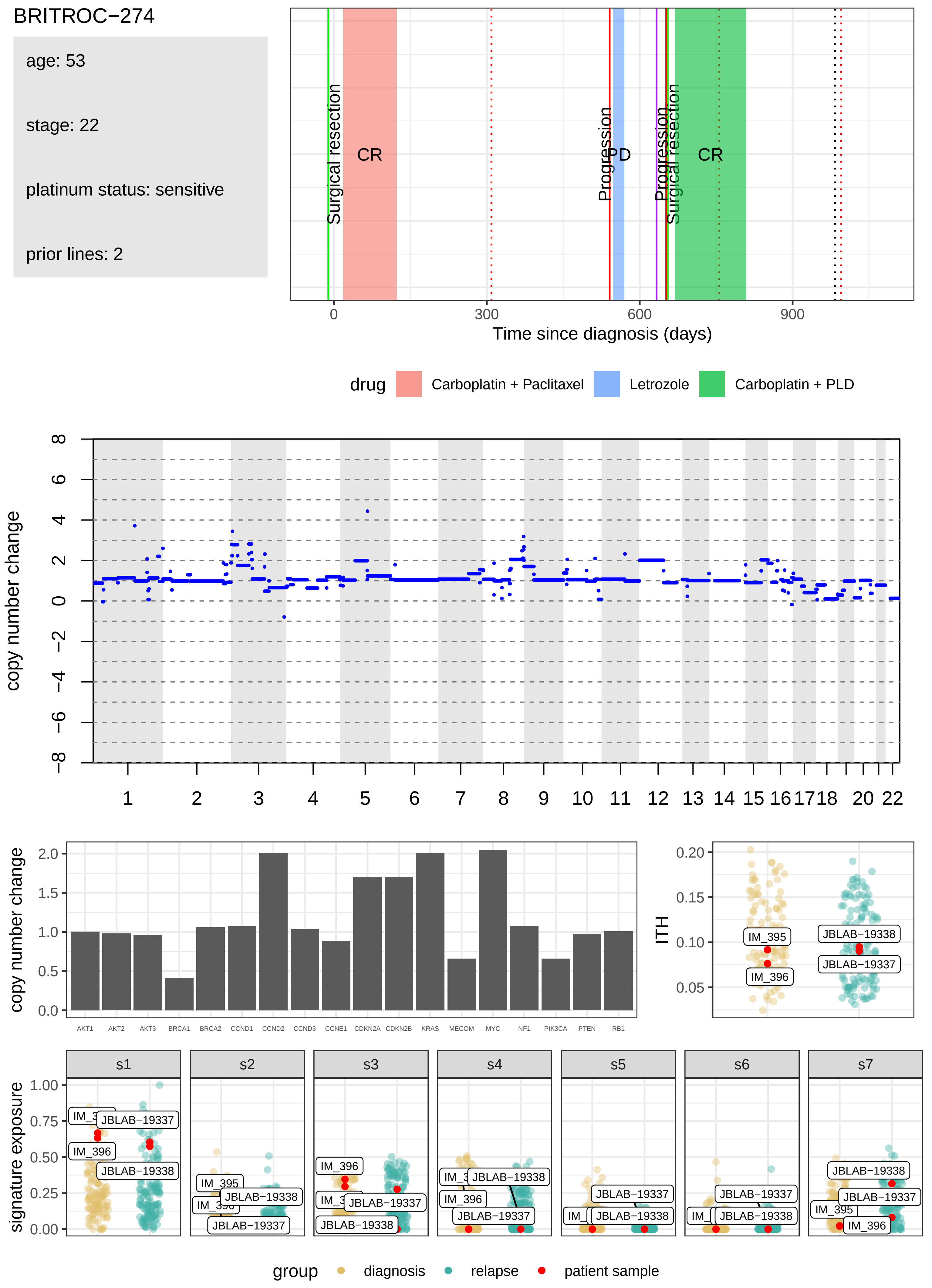
BRITROC−274
age: 53
stage: 22
platinum status: sensitive
prior lines: 2
Surgical resection
CR
Progression
PD
Progression
Surgical resection
CR
0
300
600
900
Time since diagnosis (days)
drug
Carboplatin + Paclitaxel
Letrozole
Carboplatin + PLD
copy number change
AKT1 AKT2 AKT3 BRCA1 BRCA2 CCND1 CCND2 CCND3 CCNE1 CDKN2A CDKN2B KRAS MECOM MYC NF1 PIK3CA PTEN RB1
ITH
IM_395
IM_396
JBLAB−19338
JBLAB−19337
signature exposure
s1 s2 s3 s4 s5 s6 s7
group
diagnosis
relapse
patient sample

BRITROC−32

age: 70

stage: 33

platinum status: resistant

prior lines: 1

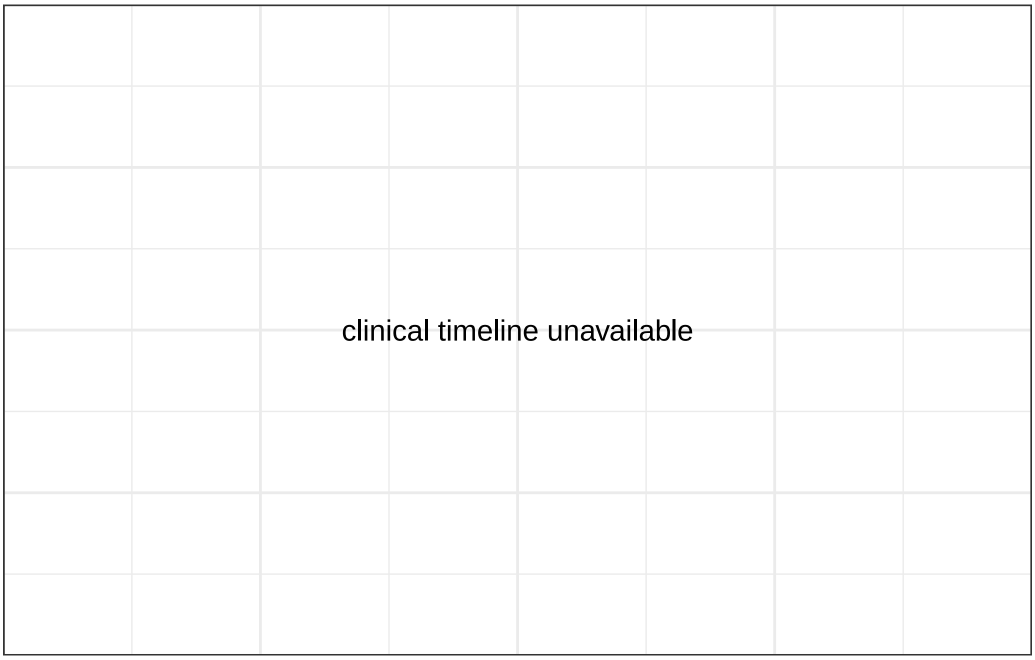

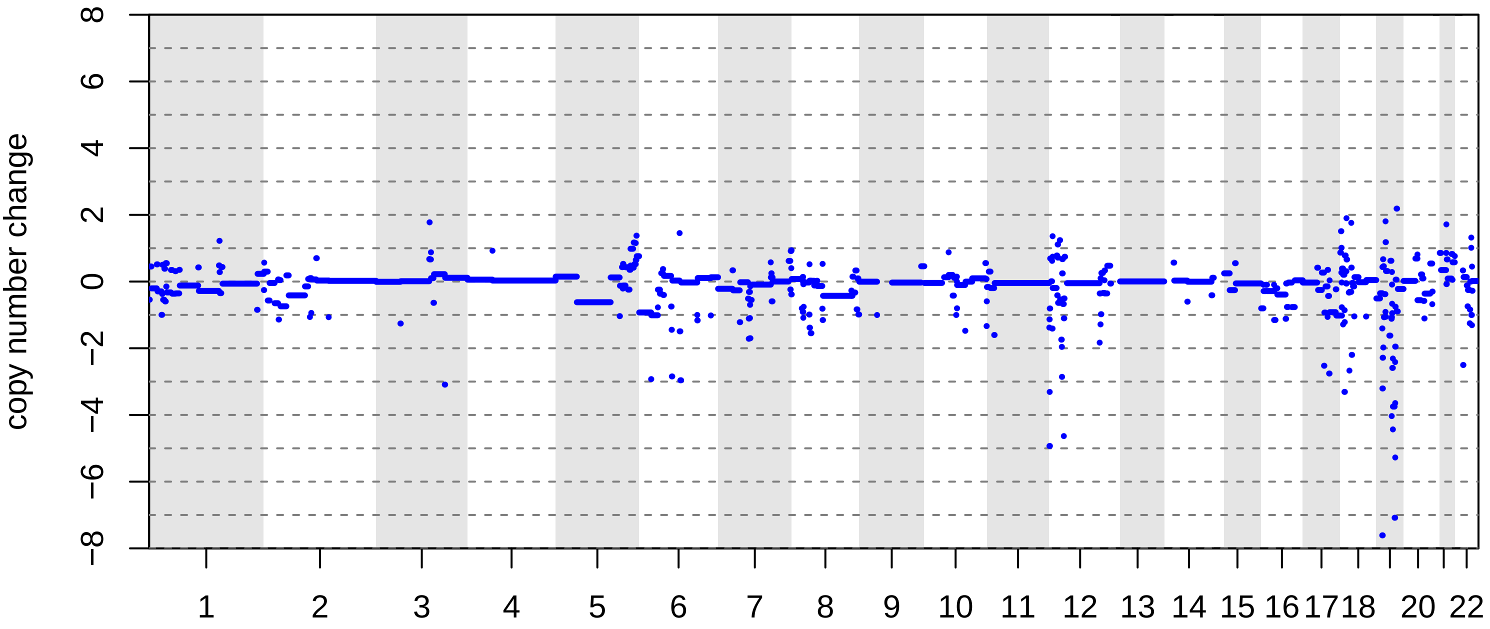

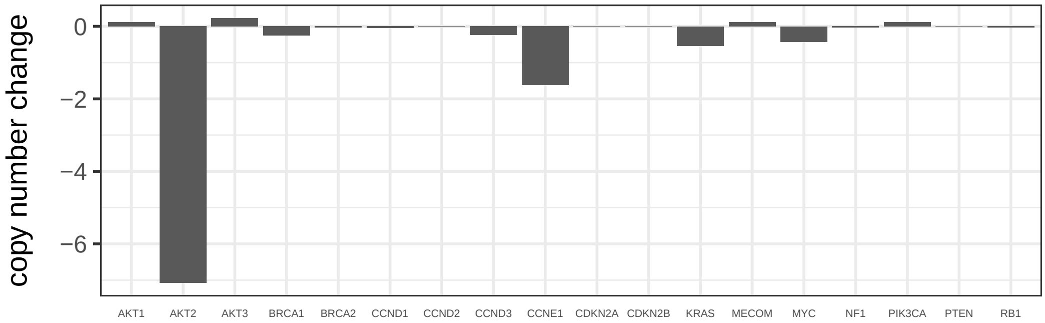

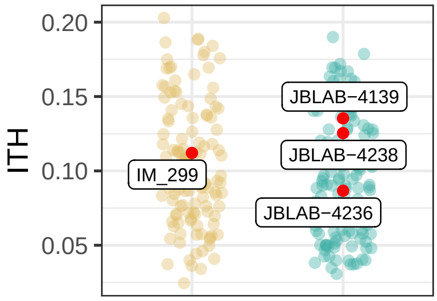

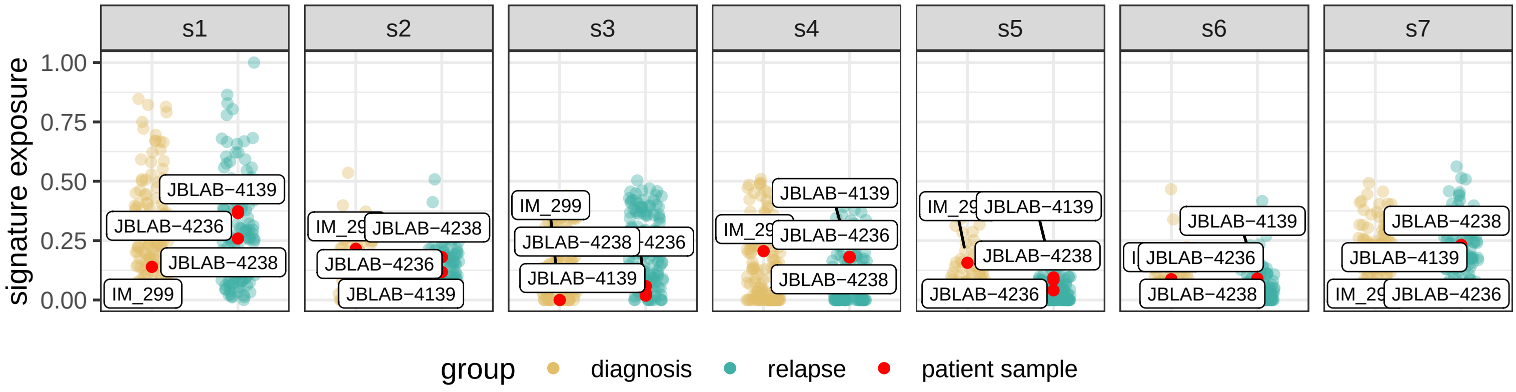

BRITROC−34

age: 75

stage: 33

platinum status: sensitive

prior lines: 1

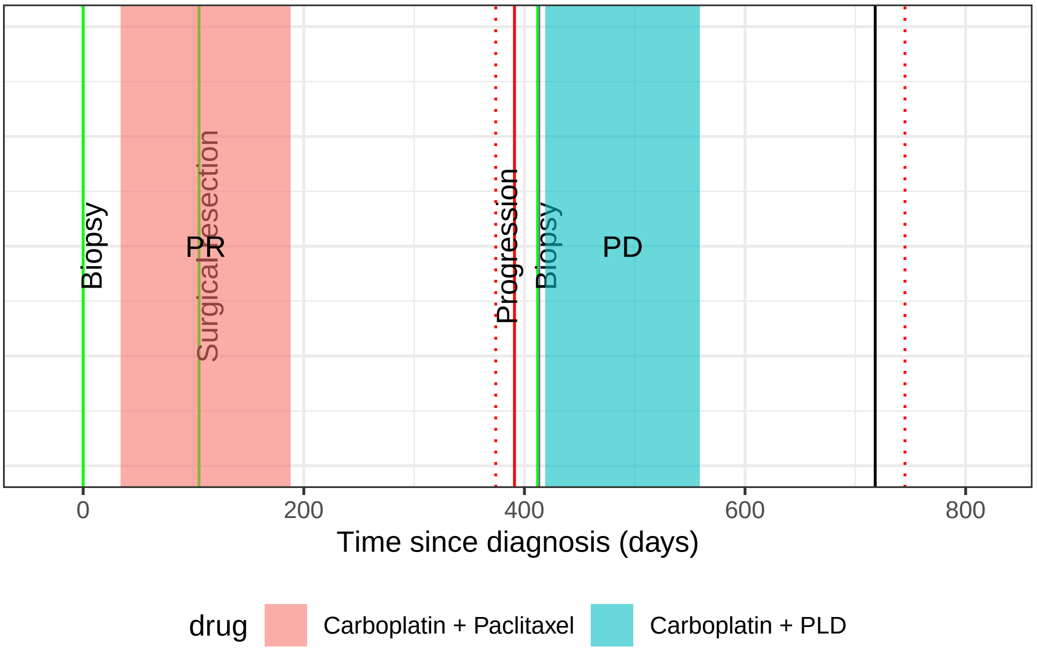

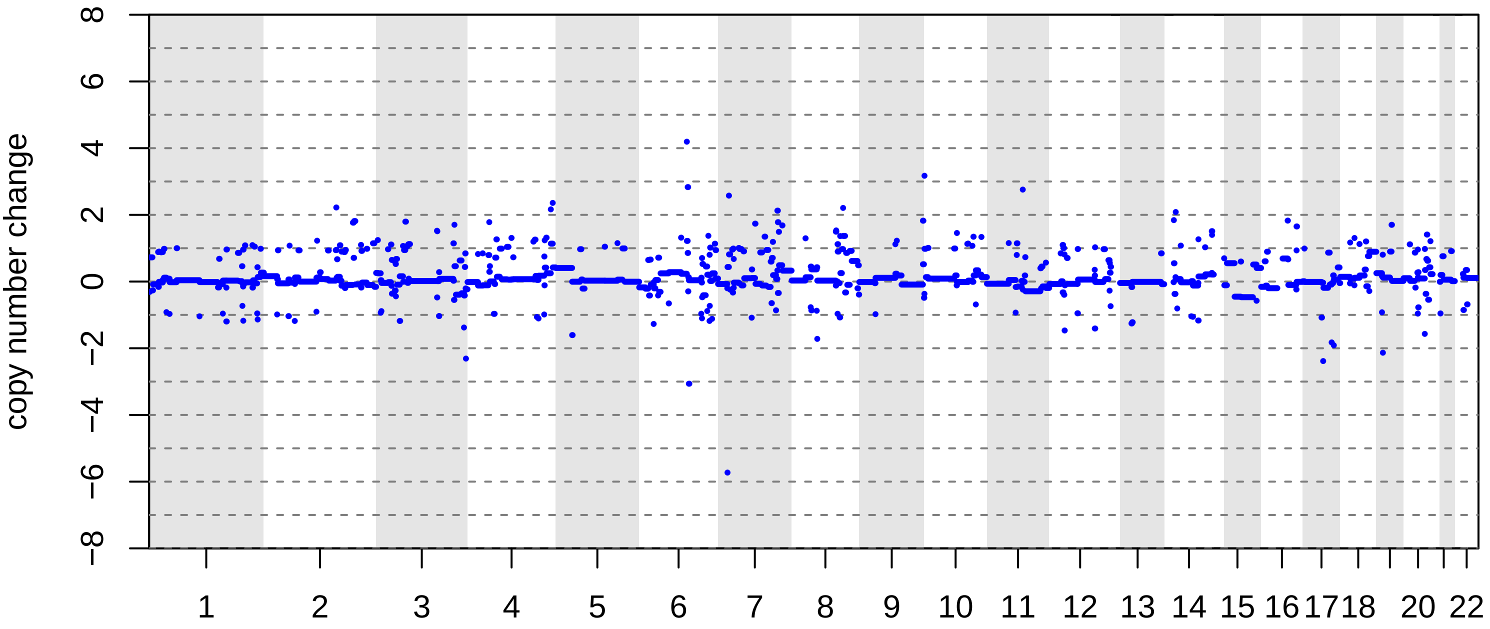

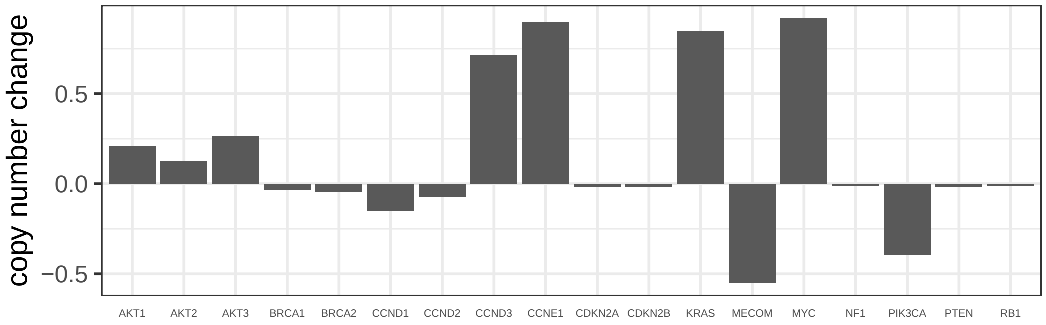

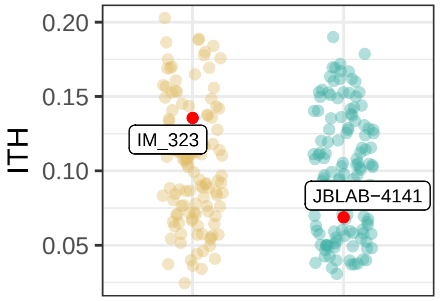

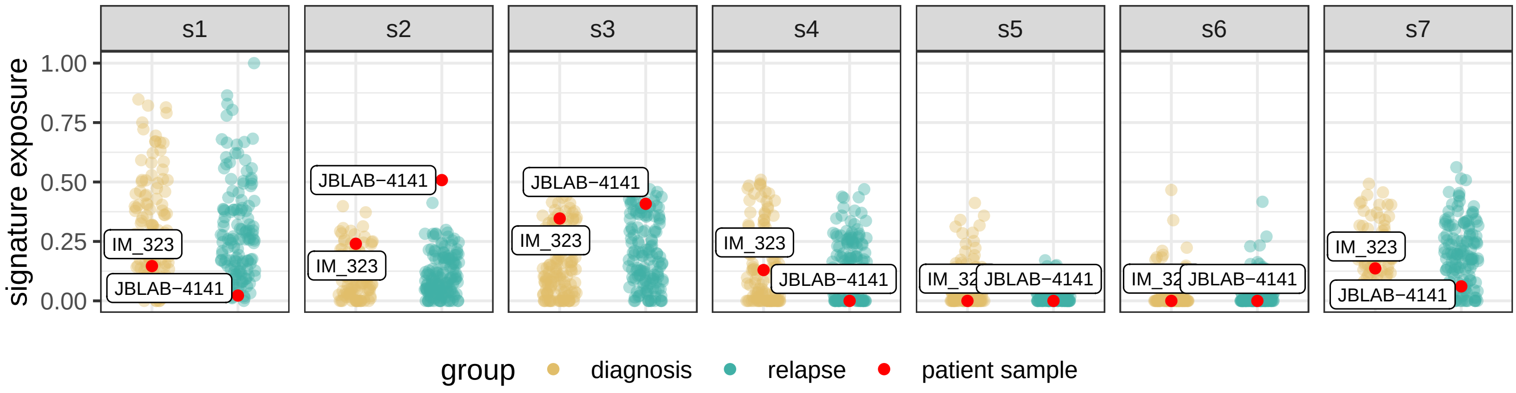

BRITROC−36

age: 66

stage: 32

platinum status: sensitive

prior lines: 1

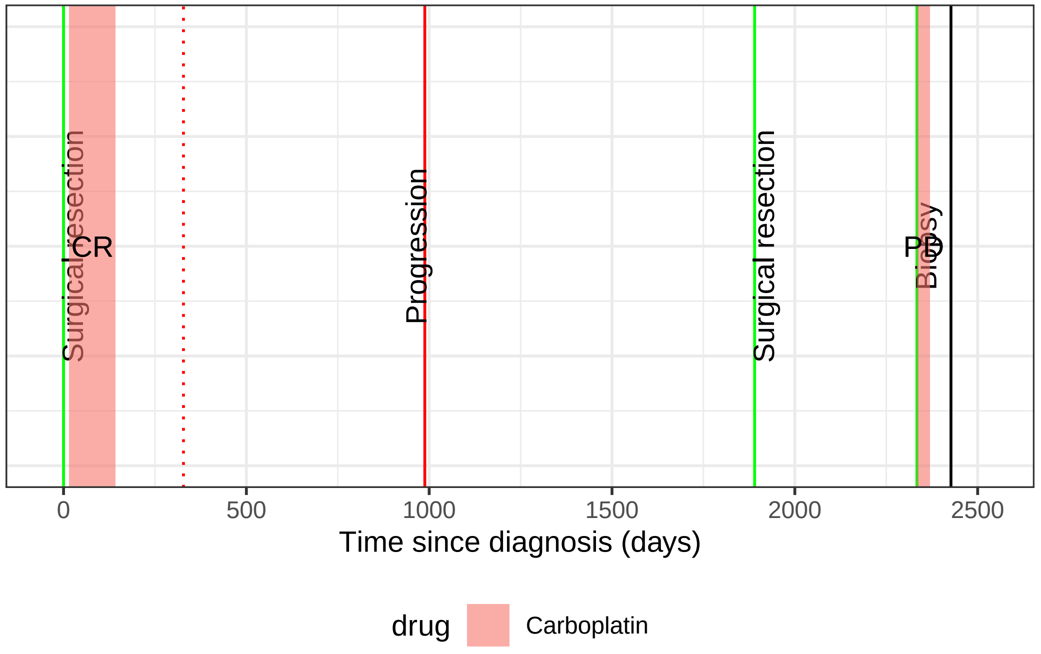

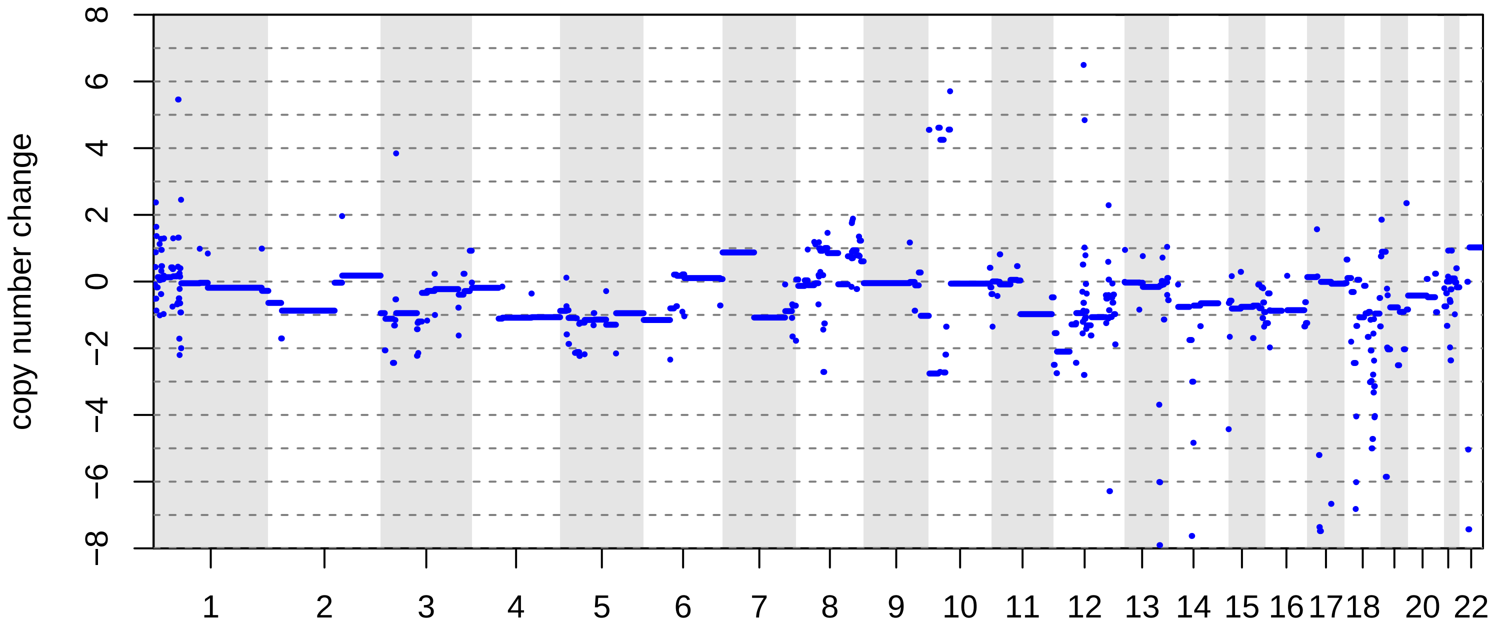

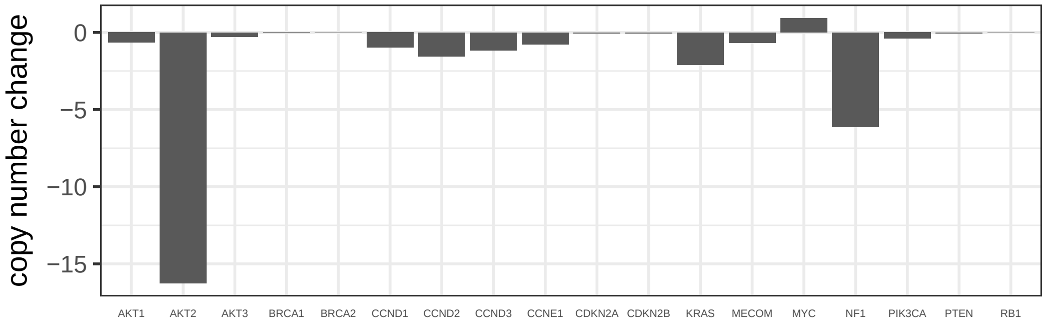

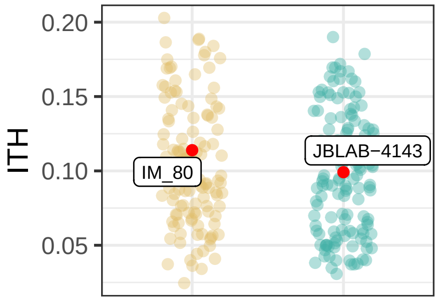

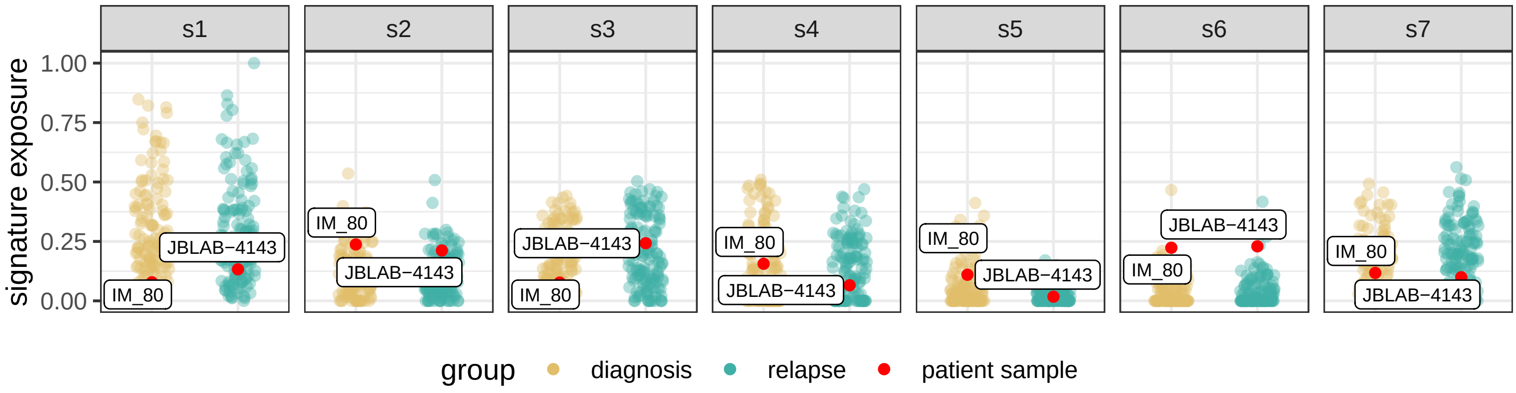

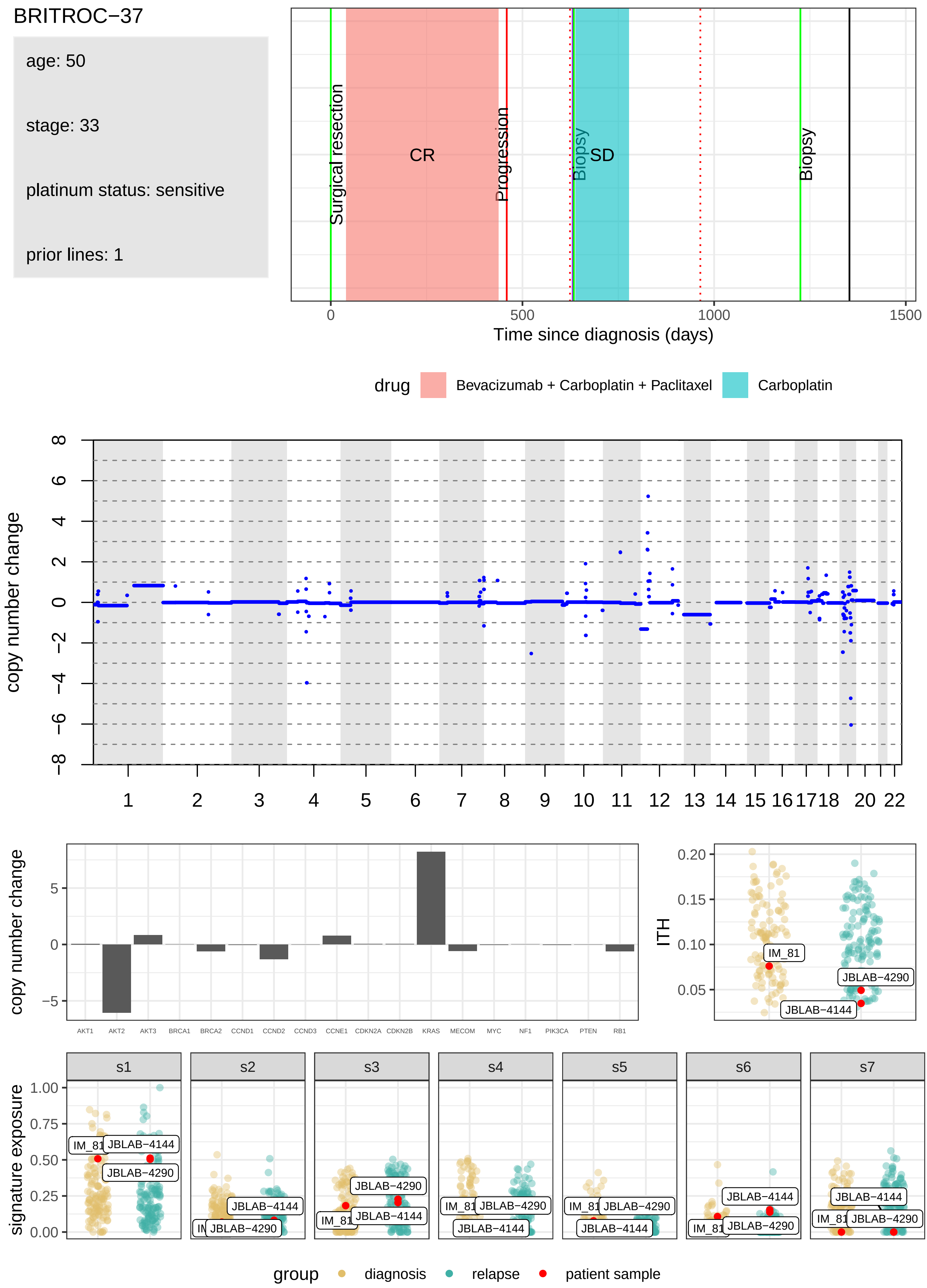
BRITROC−37
age: 50
stage: 33
platinum status: sensitive
prior lines: 1
Surgical resection
CR
Progression
Biopsy
SD
Biopsy
Time since diagnosis (days)
drug
Bevacizumab + Carboplatin + Paclitaxel
Carboplatin
copy number change
copy number change
ITH
signature exposure
s1
s2
s3
s4
s5
s6
s7
group
diagnosis
relapse
patient sample

BRITROC−39

age: 64

stage: 31

platinum status: sensitive

prior lines: 1

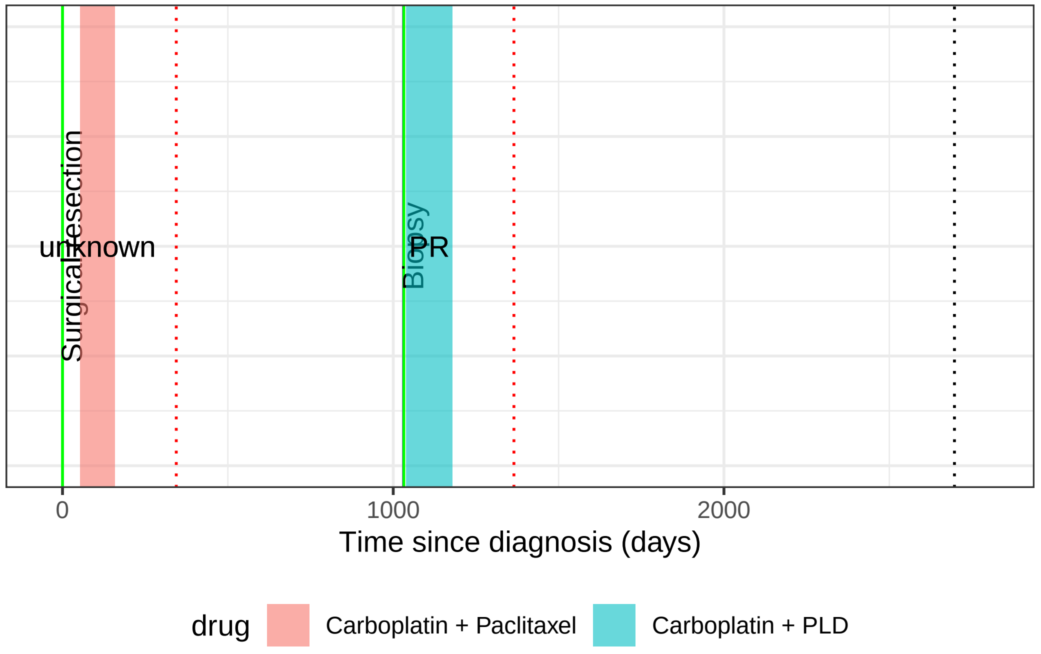

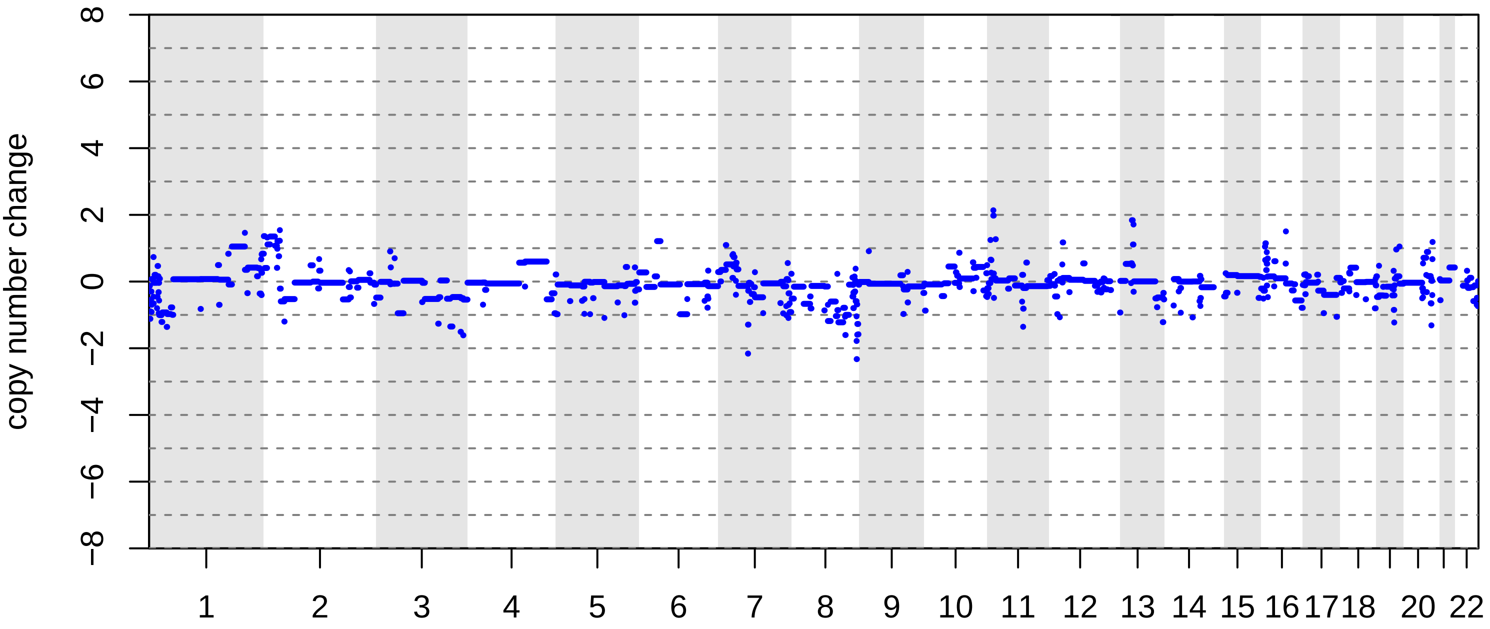

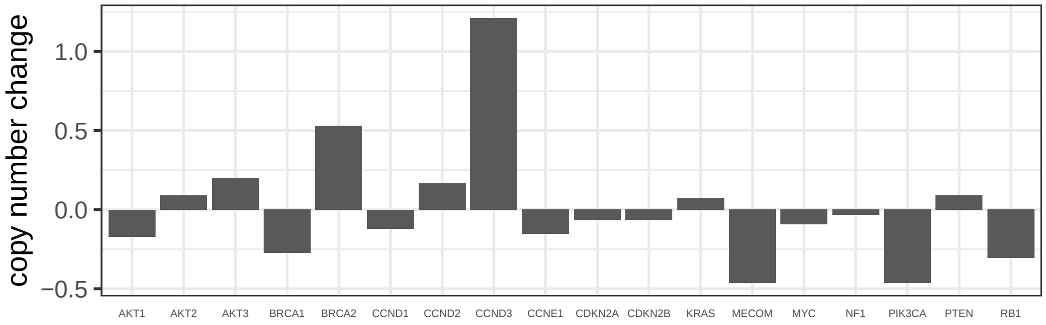

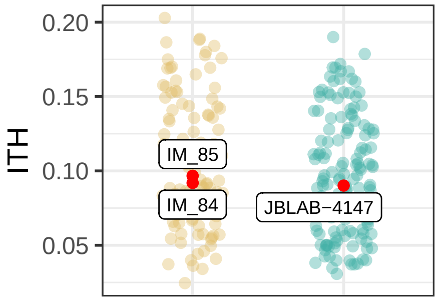

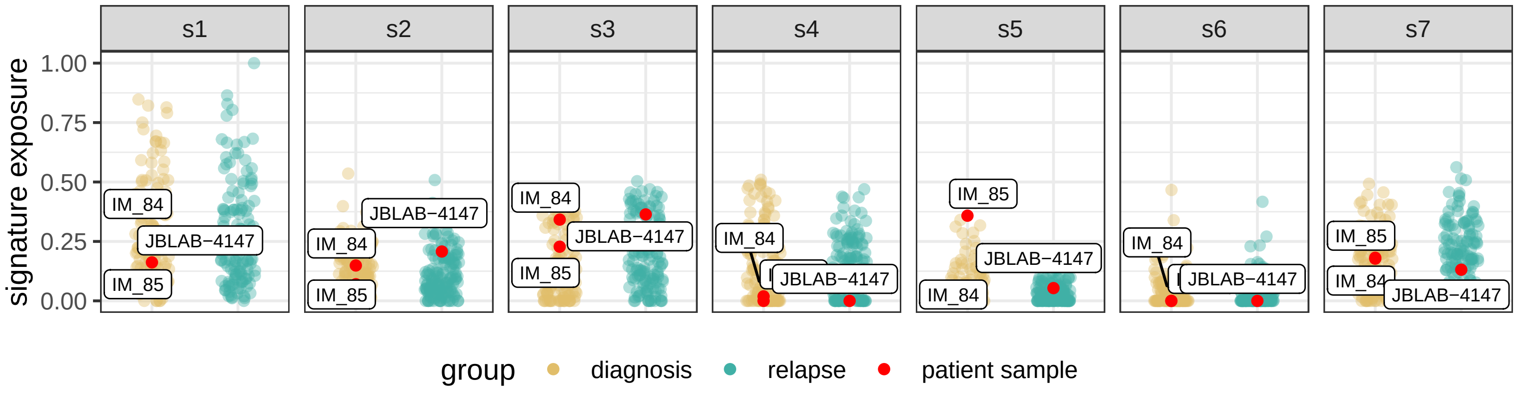

BRITROC−5

age: 49

stage: 33

platinum status: sensitive

prior lines: 2

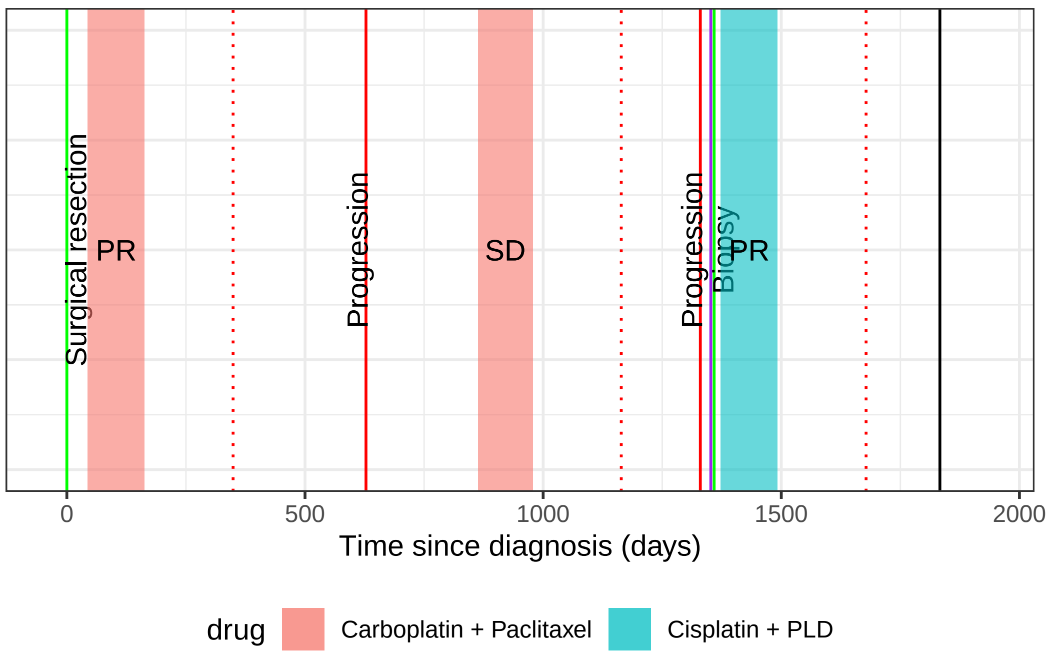

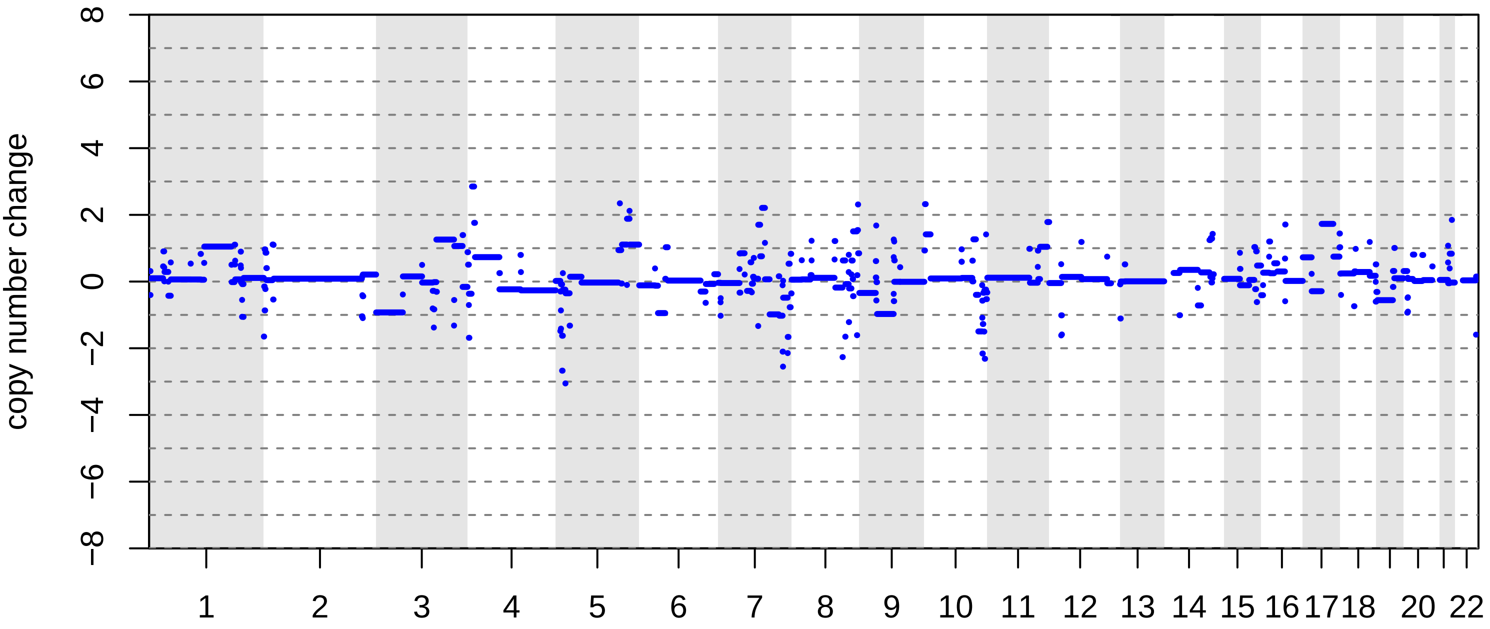

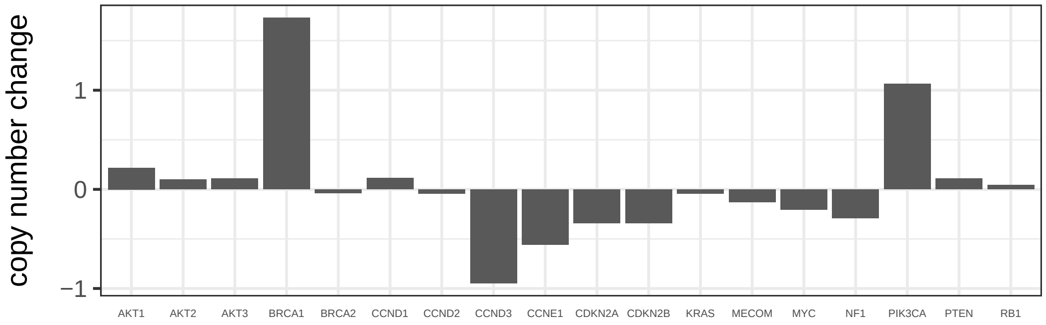

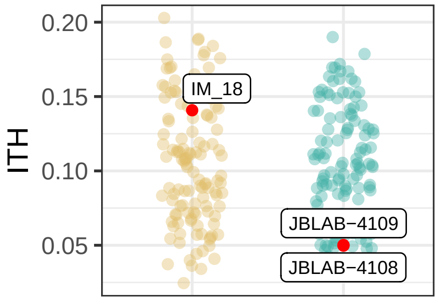

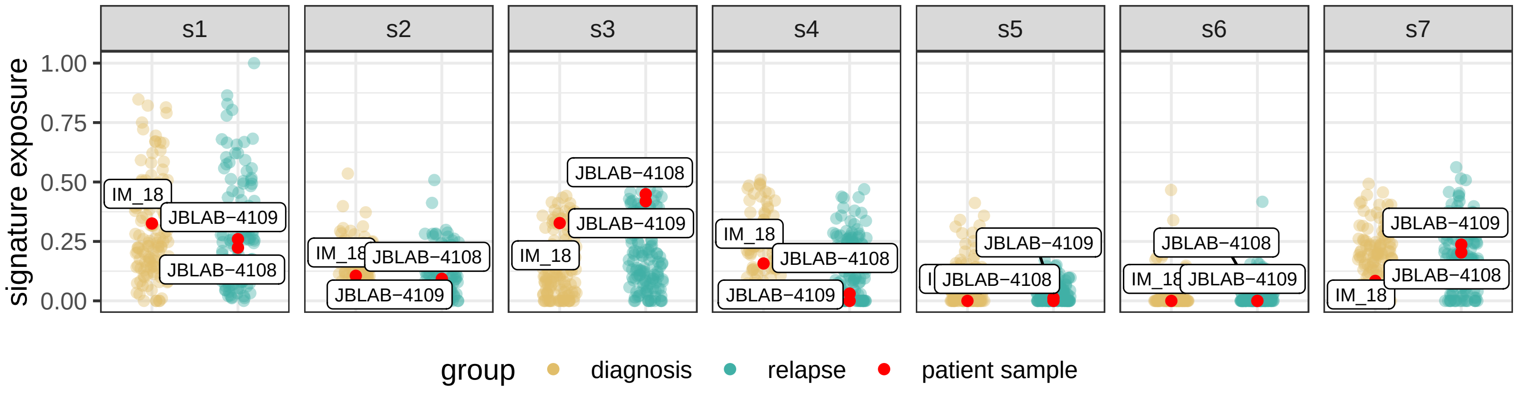

BRITROC−55

age: 58

stage: 13

platinum status: sensitive

prior lines: 2

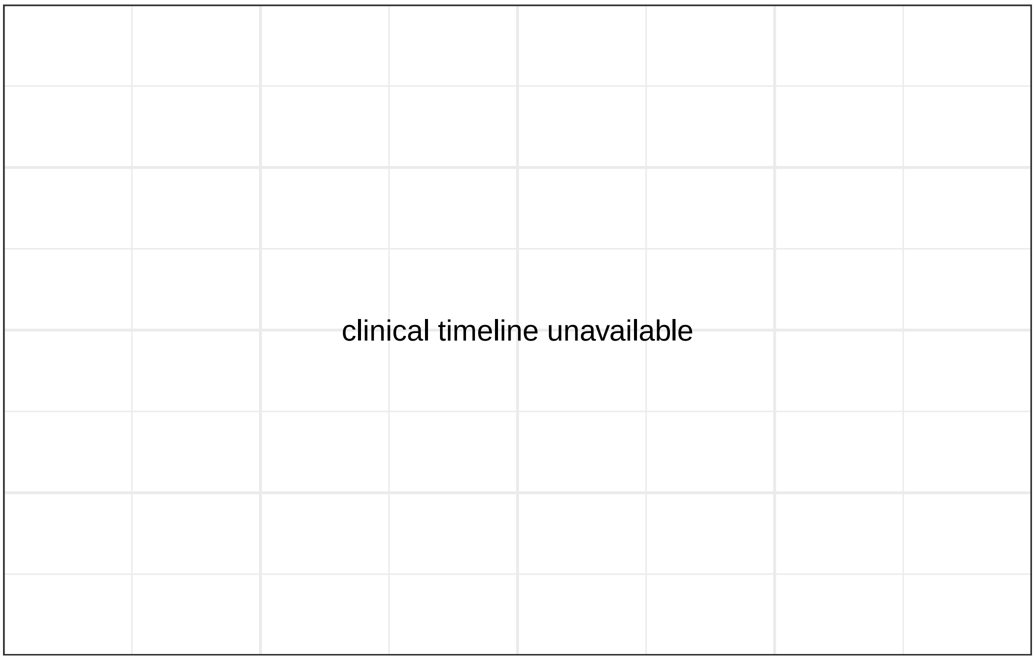

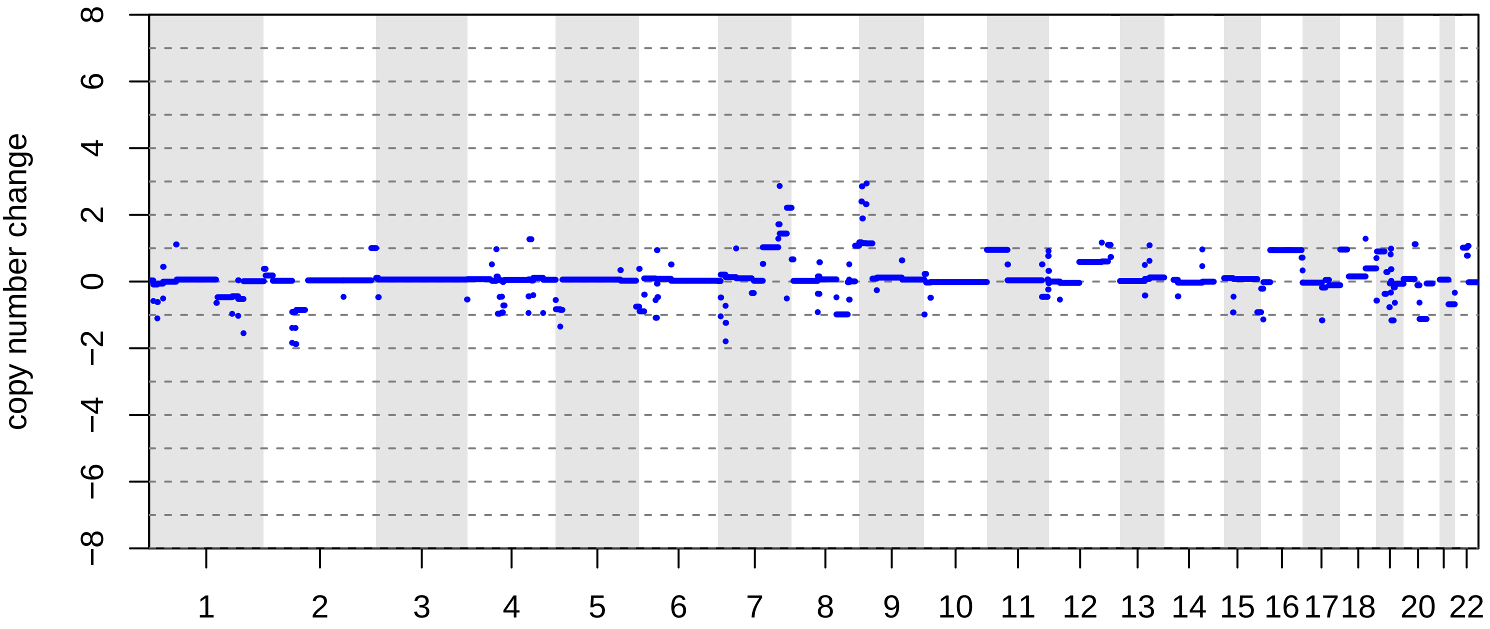

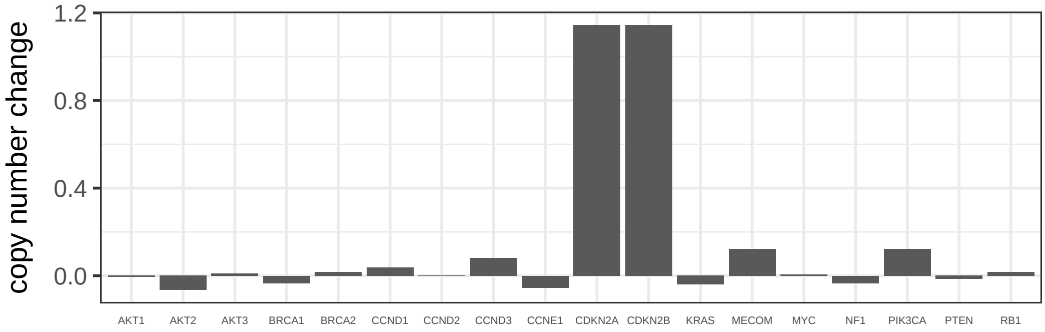

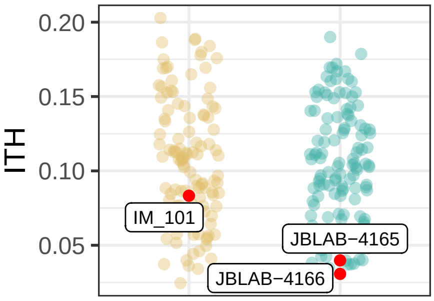

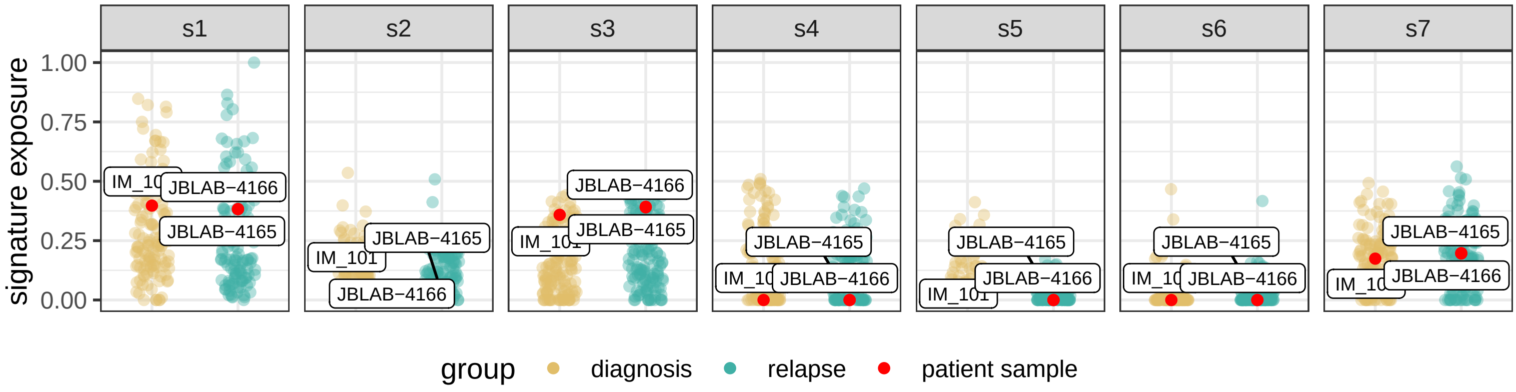

BRITROC−65

age: 70

stage: 33

platinum status: sensitive

prior lines: 1

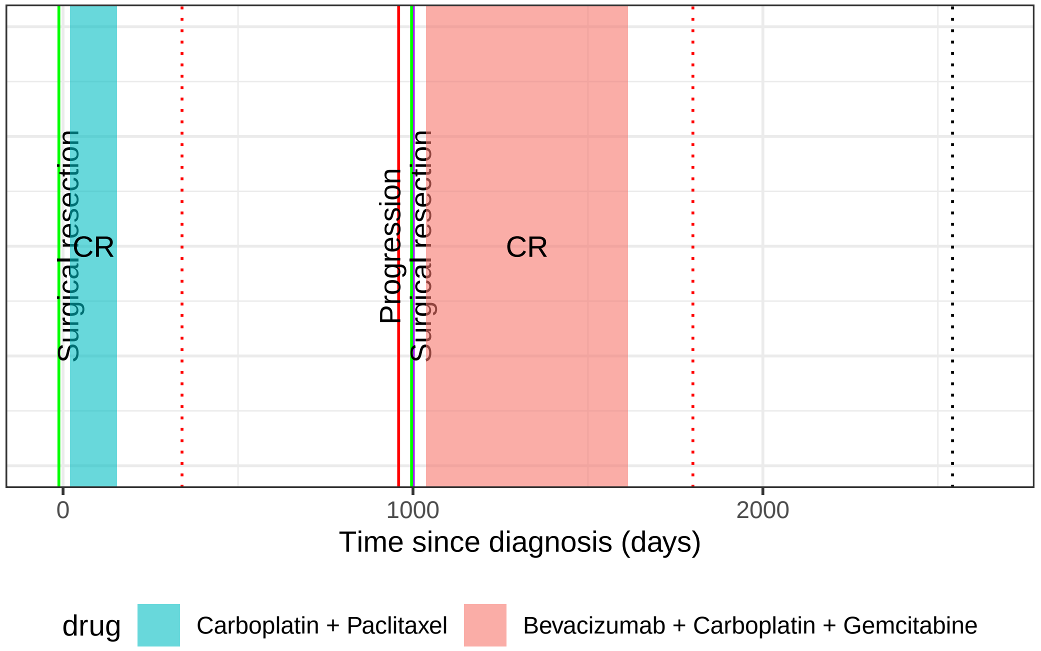

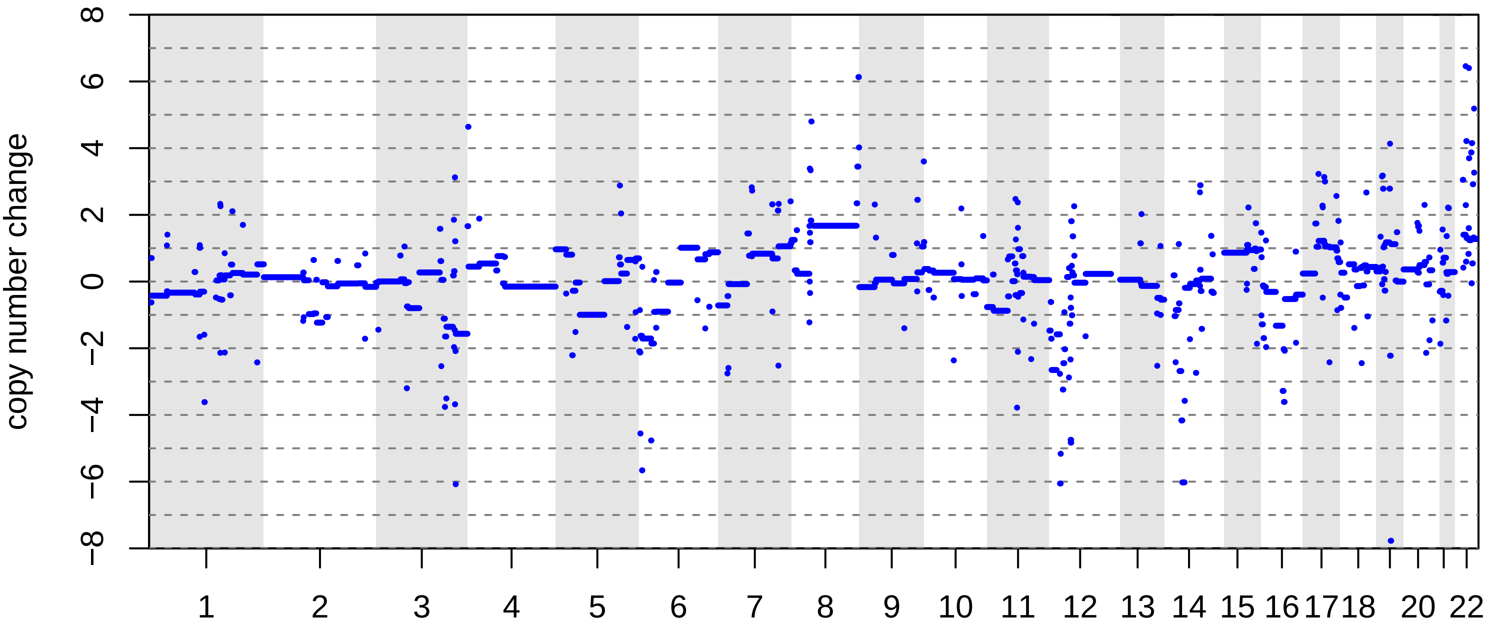

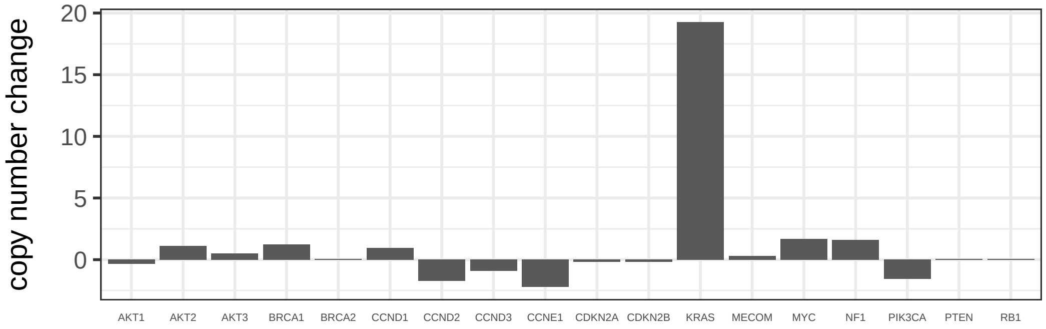

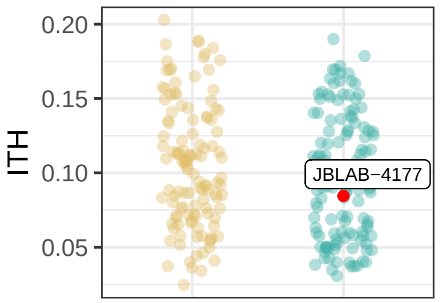

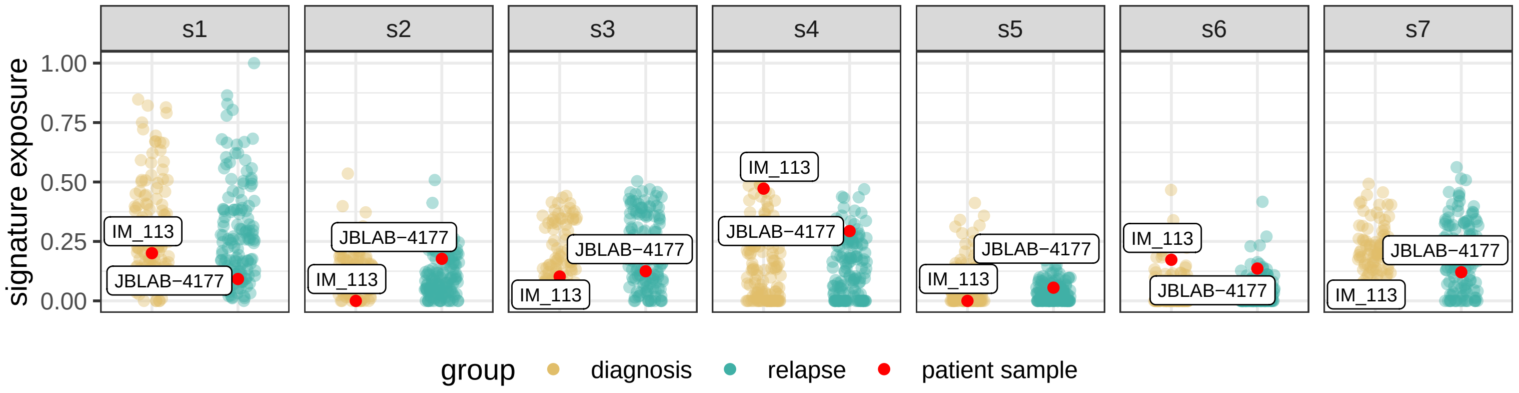

BRITROC−67

age: 68

stage: 33

platinum status: sensitive

prior lines: 1

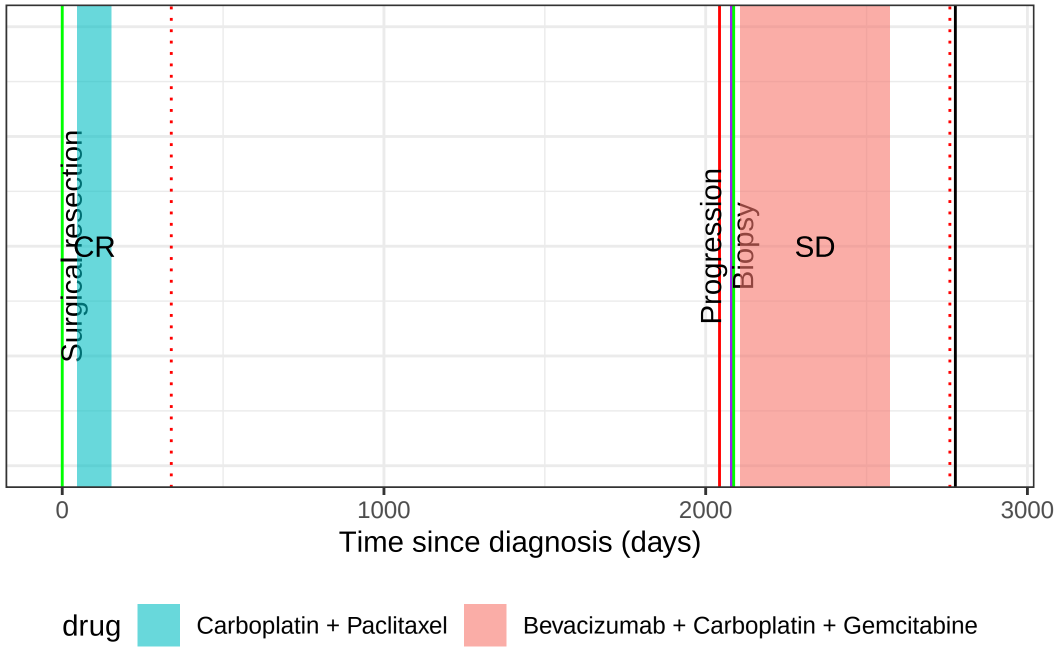

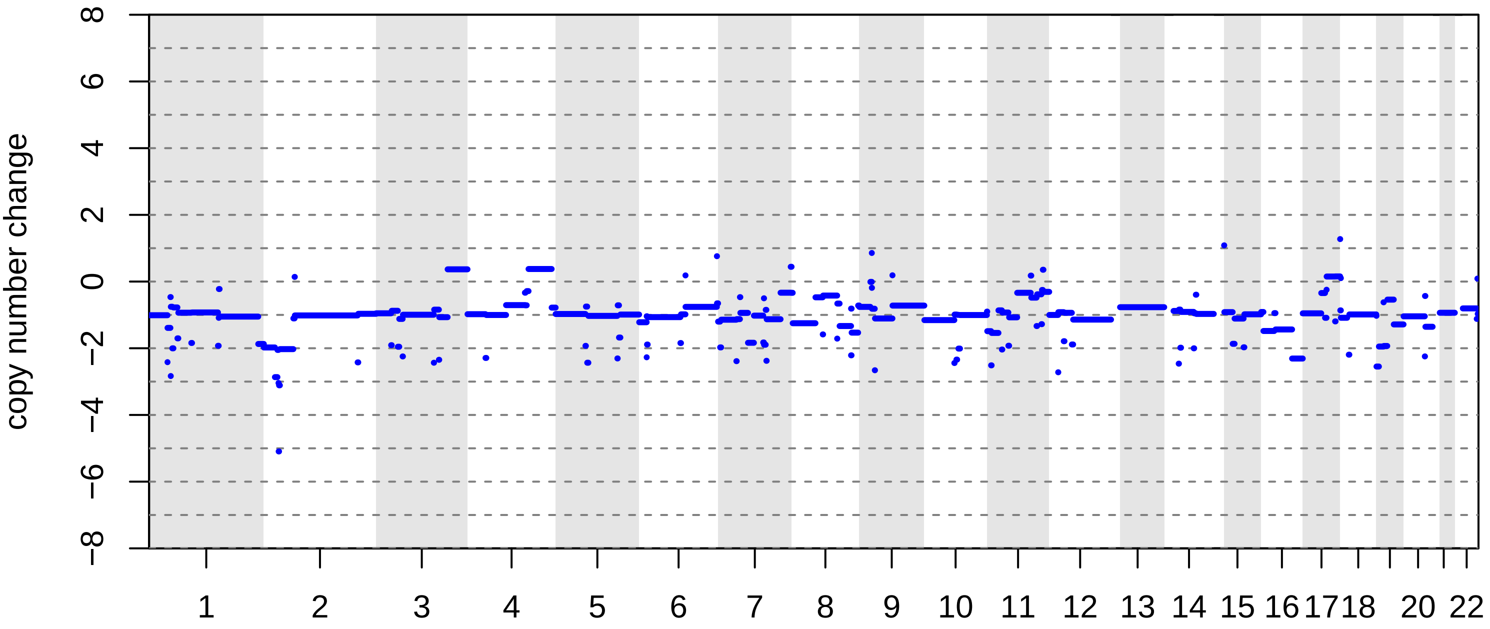

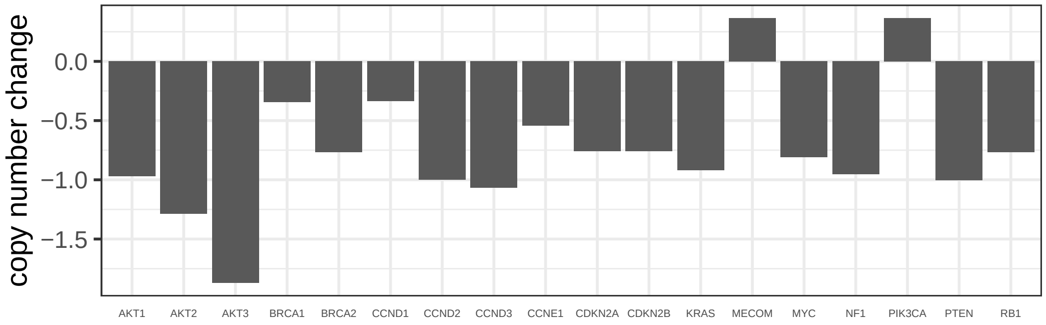

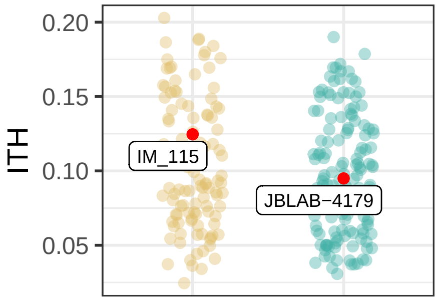

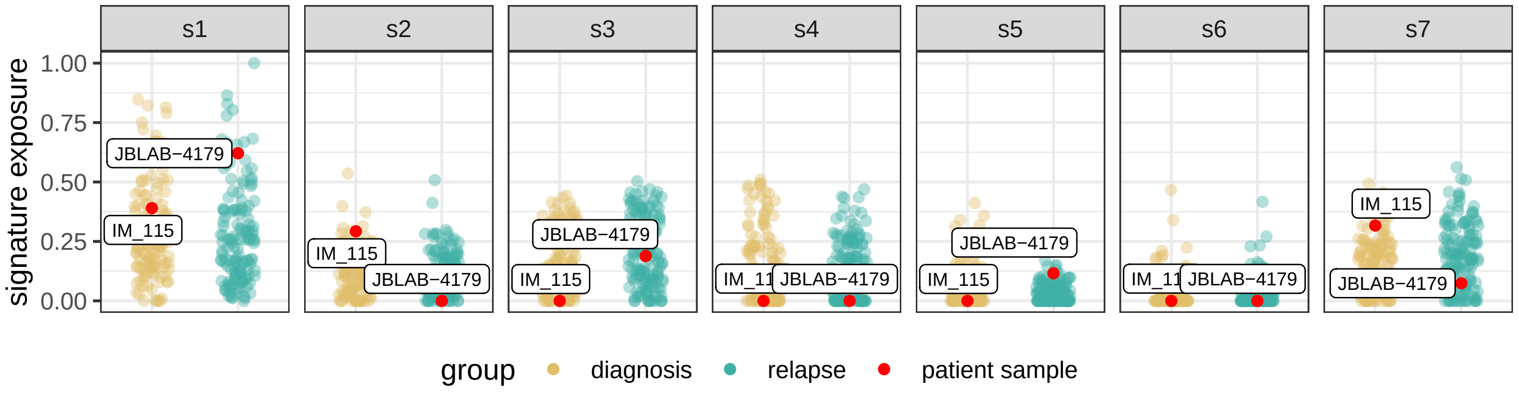

BRITROC−74

age: 64

stage: 33

platinum status: sensitive

prior lines: 1

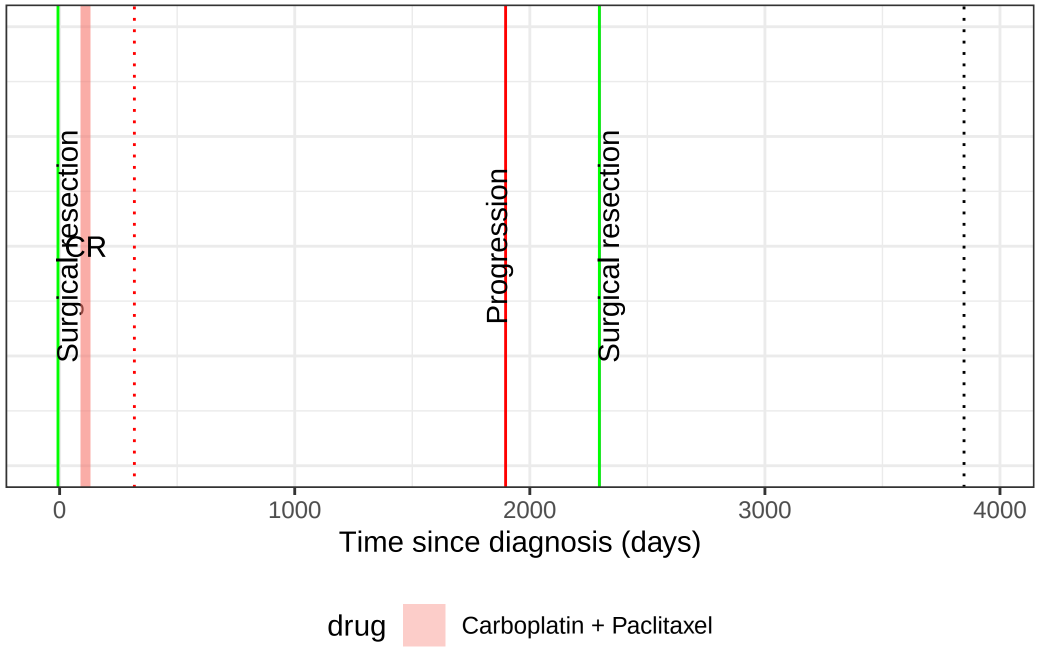

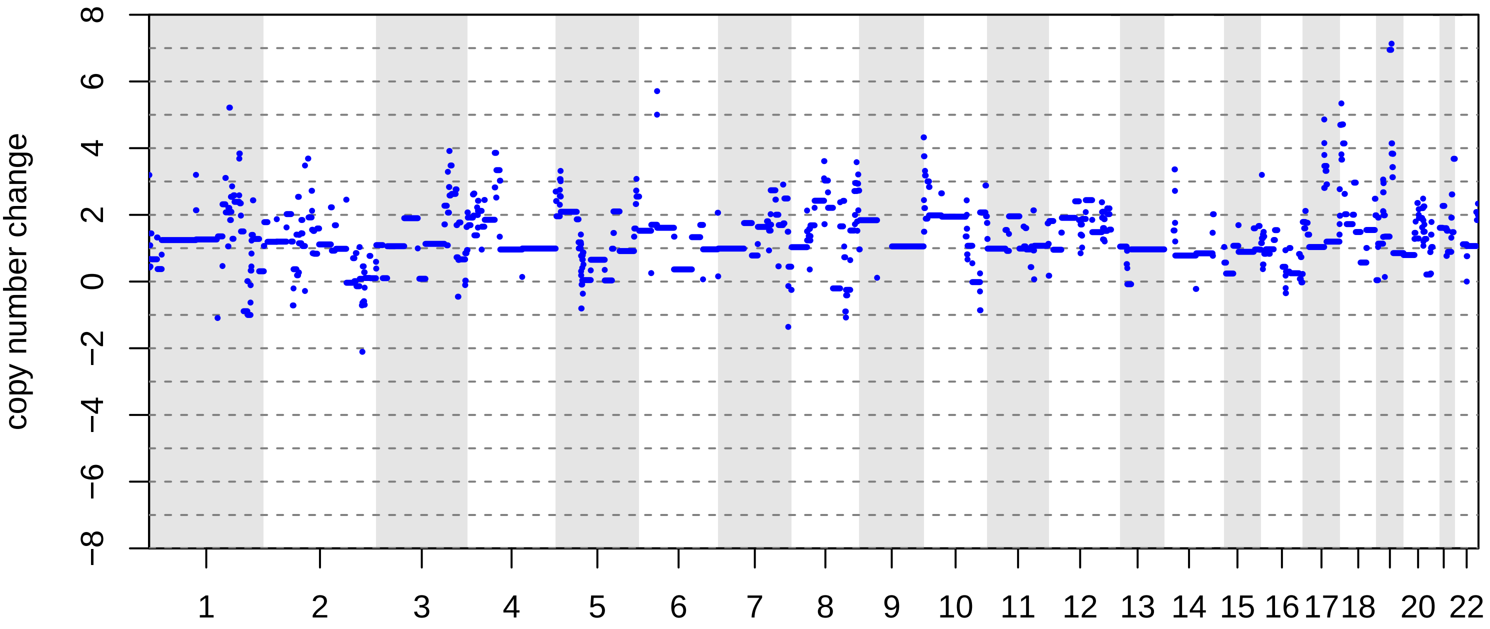

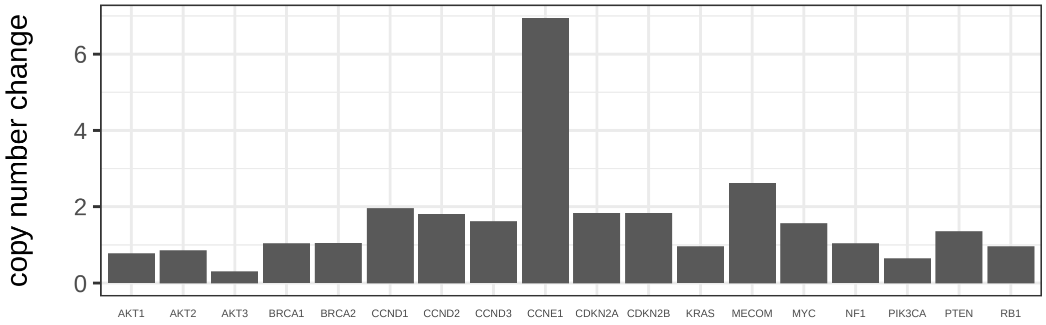

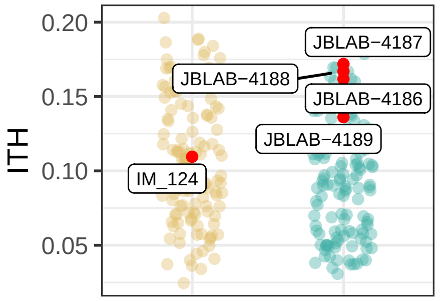

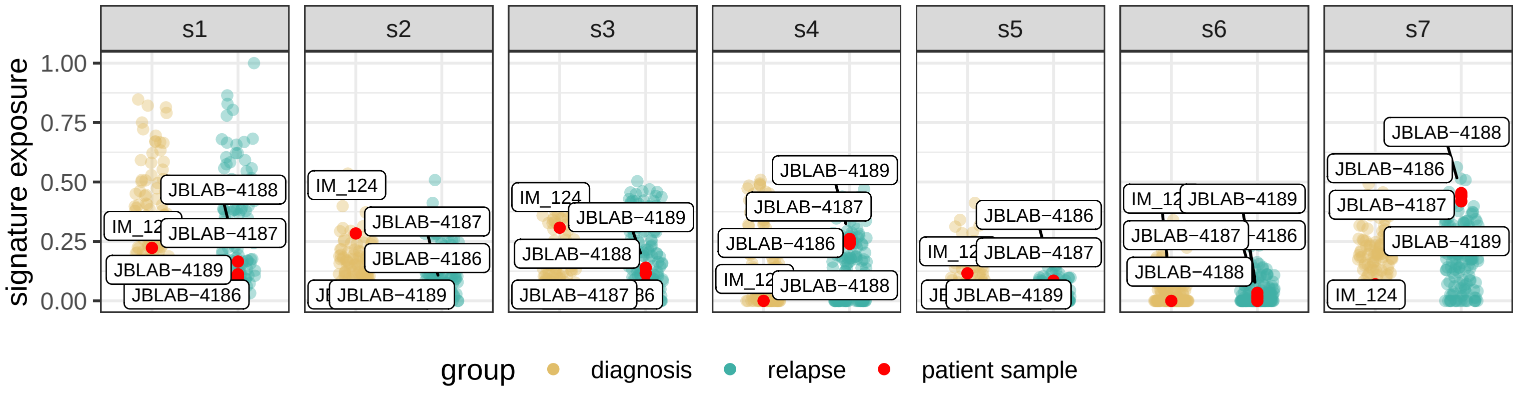

BRITROC−8

age: 60

stage: 22

platinum status: sensitive

prior lines: 1

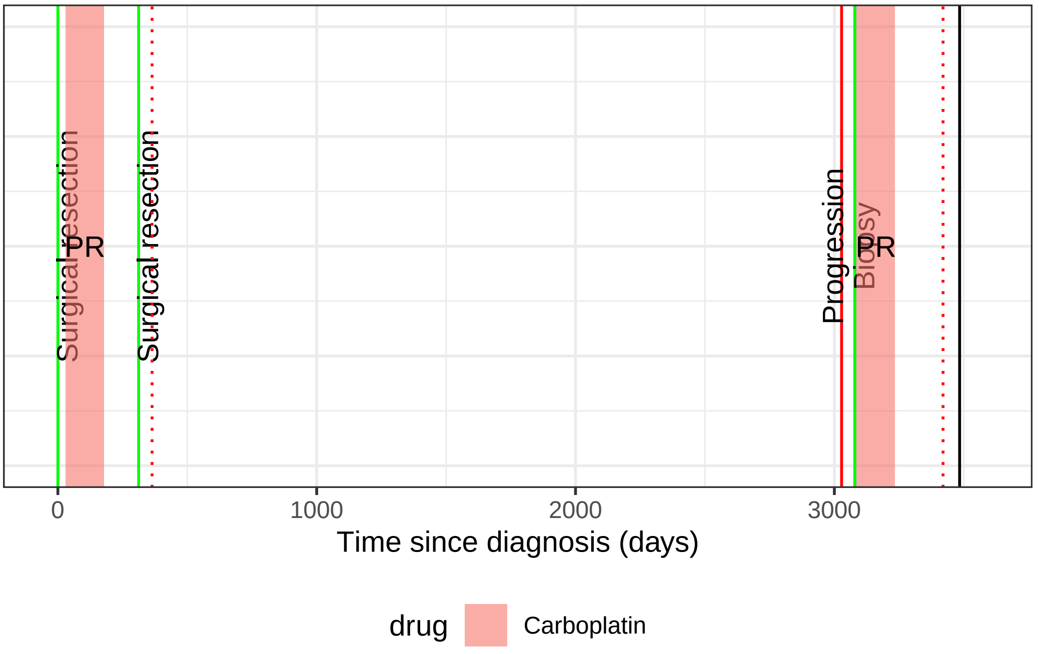

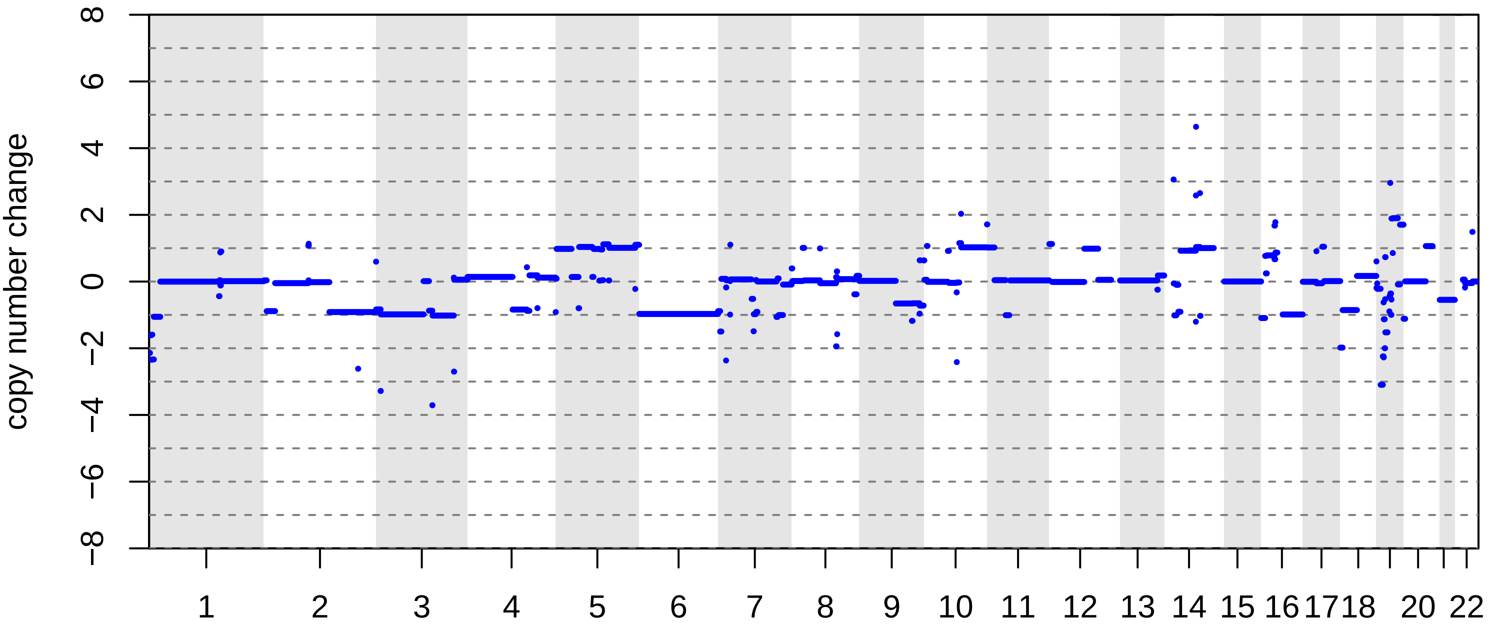

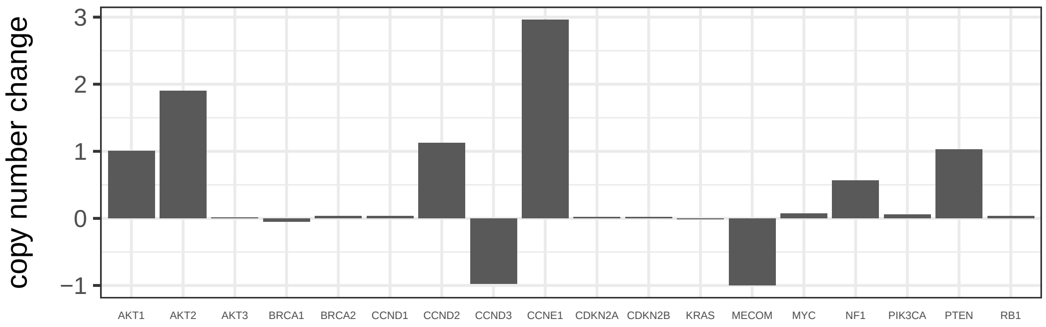

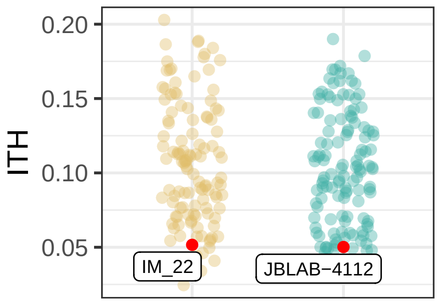

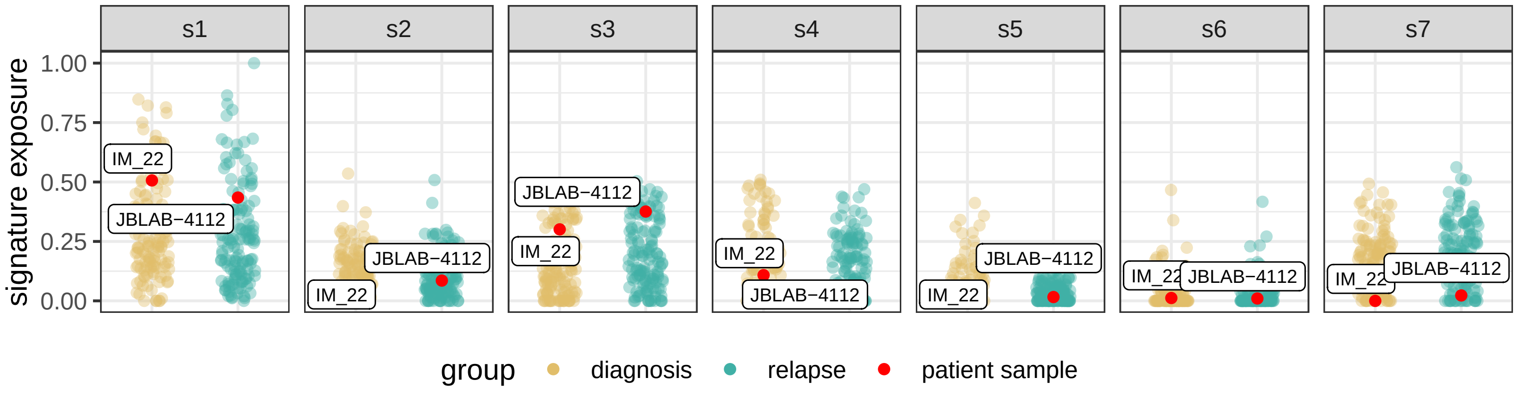

BRITROC−9

age: 47

stage: 33

platinum status: sensitive

prior lines: 1

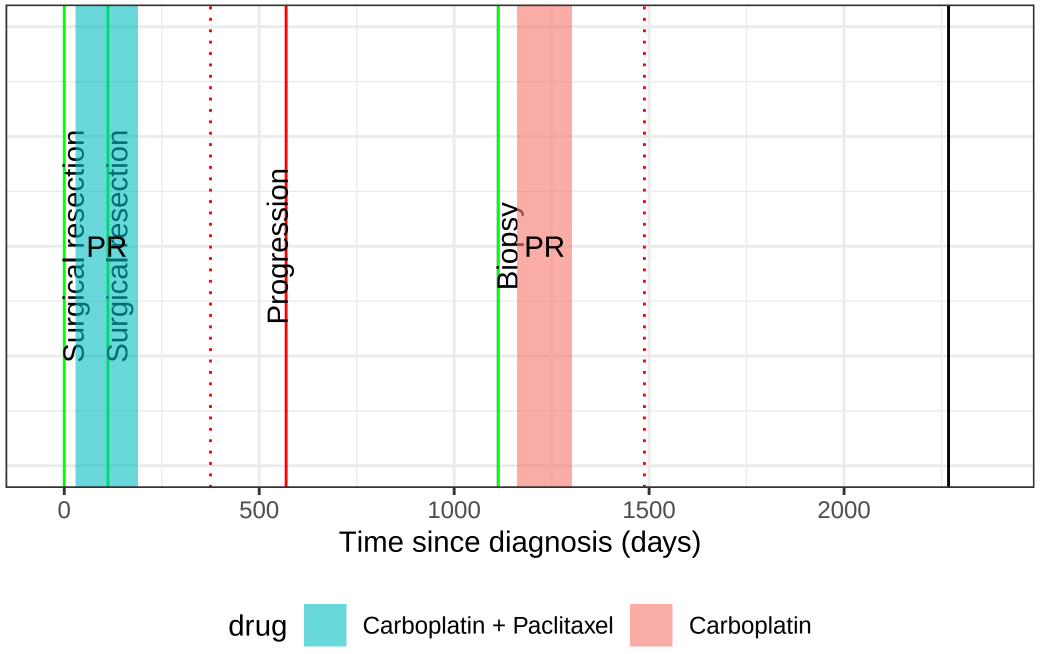

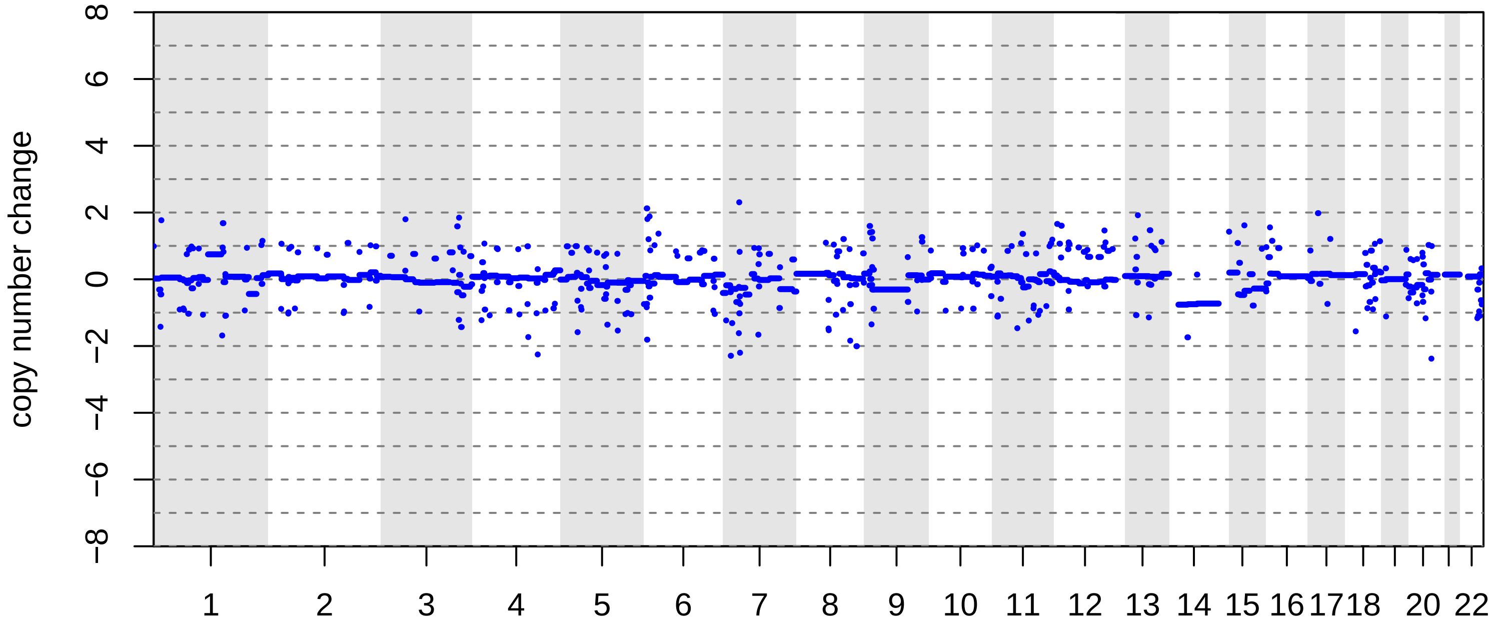

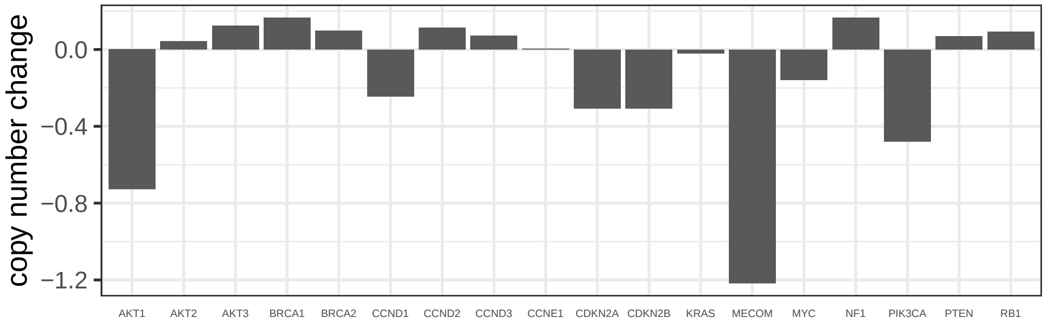

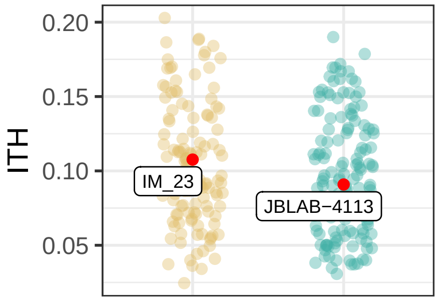

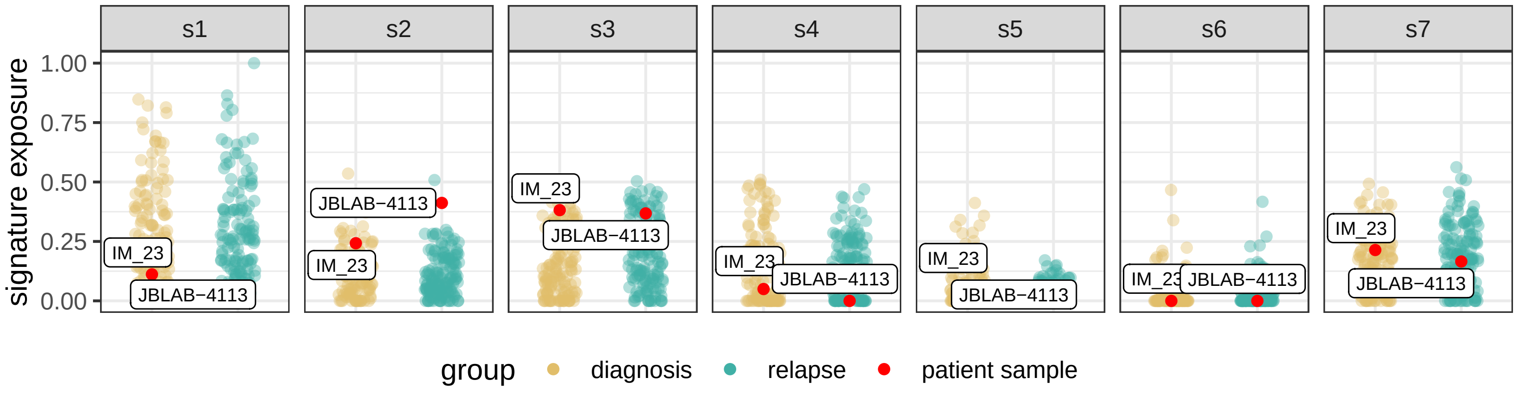

BRITROC−94

age: 71

stage: 22

platinum status: sensitive

prior lines: 2

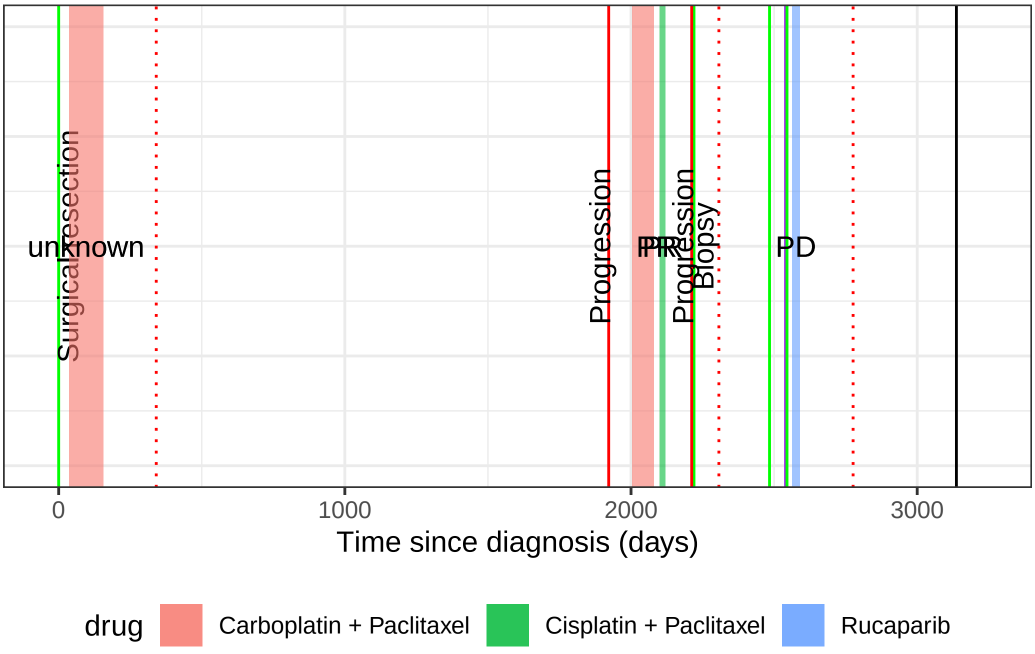

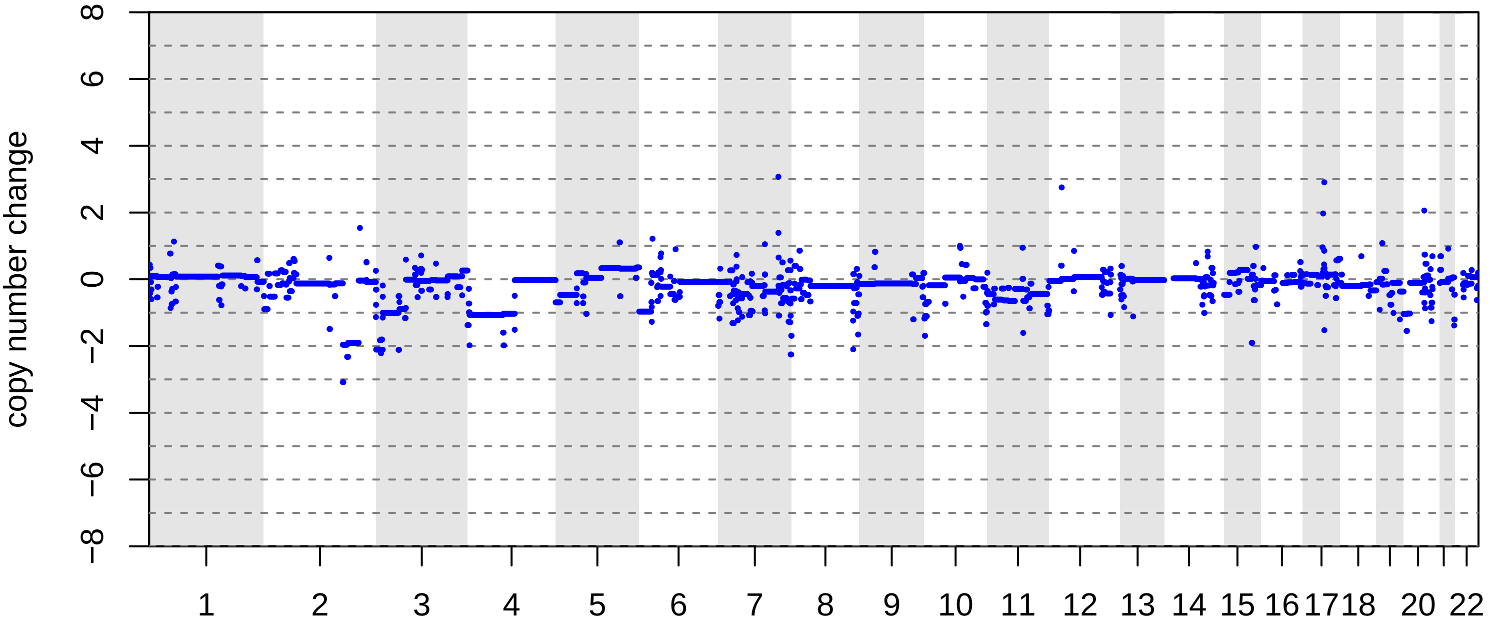

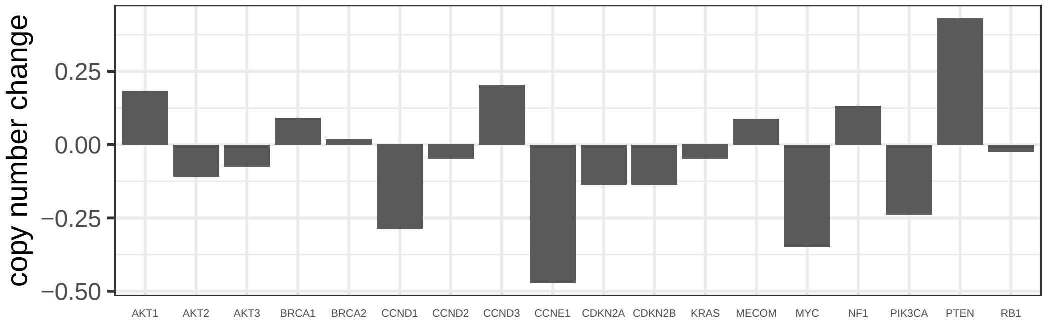

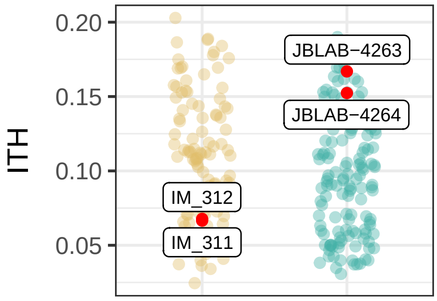

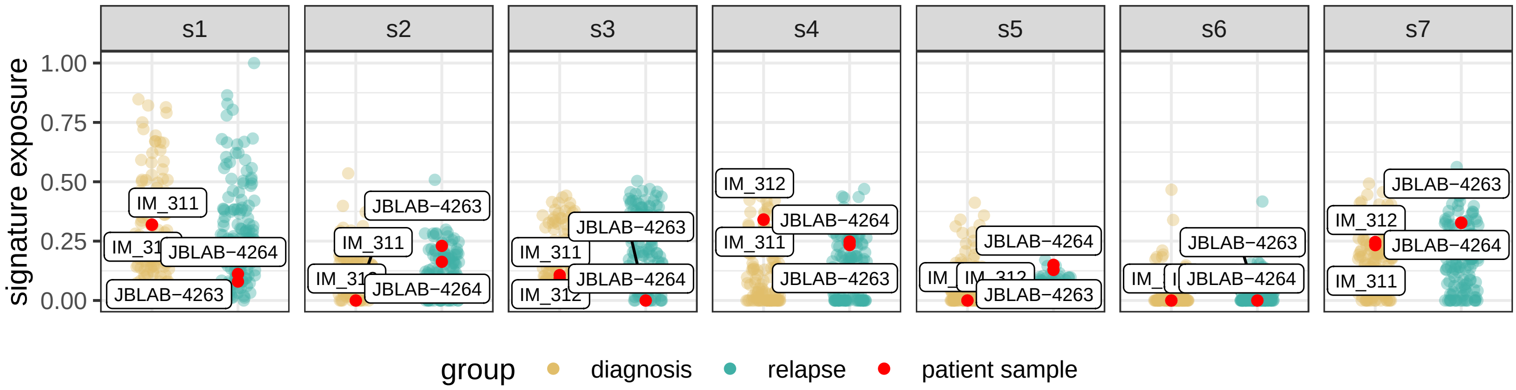

BRITROC−96

age: 64

stage: 32

platinum status: resistant

prior lines: 4

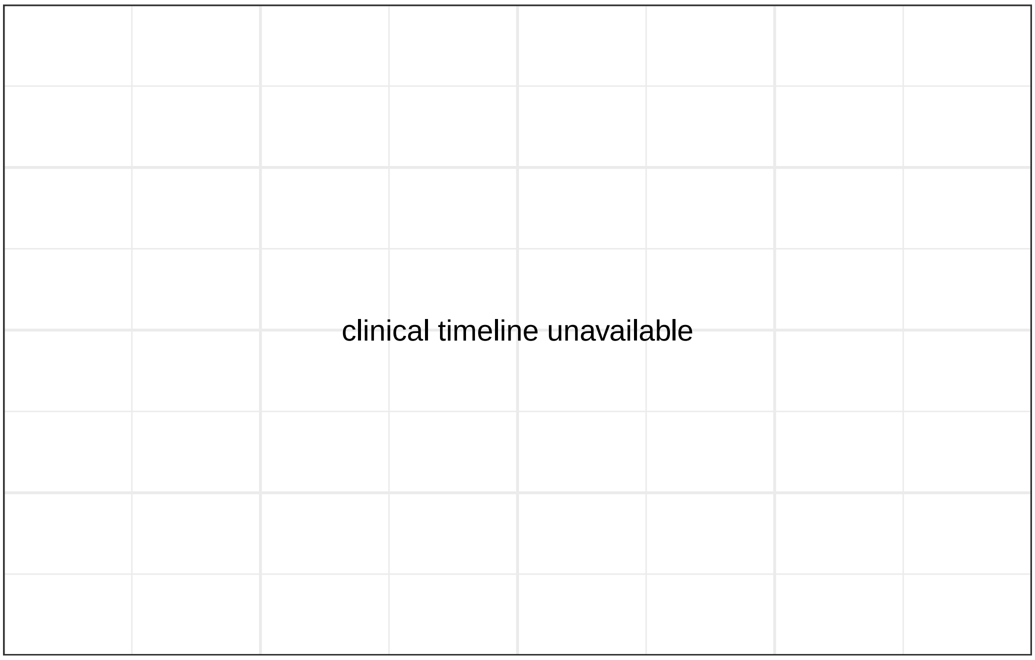

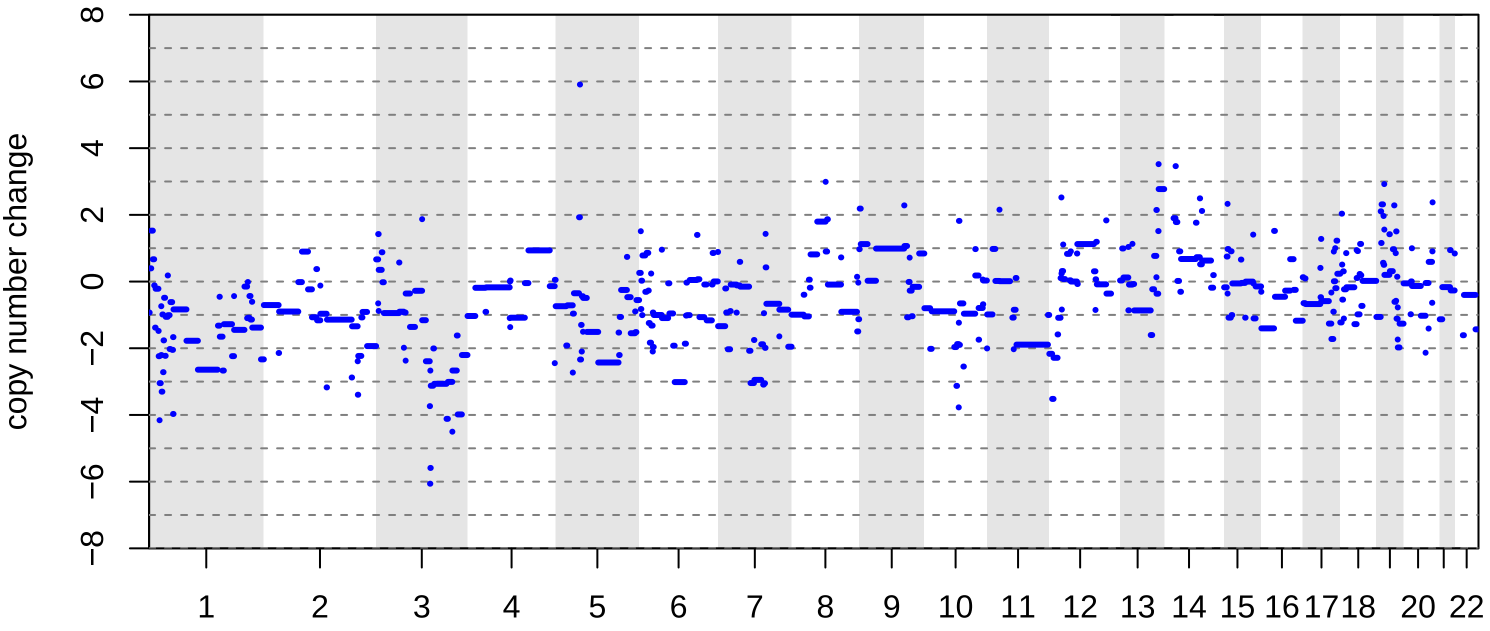

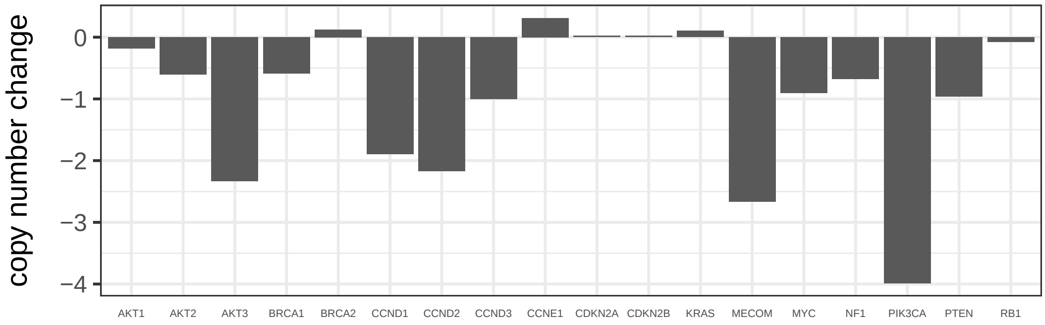

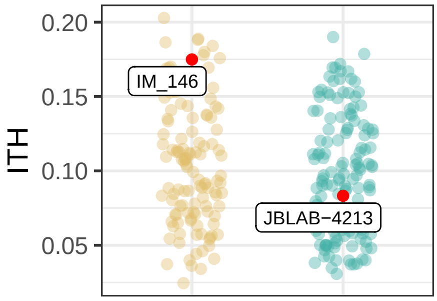

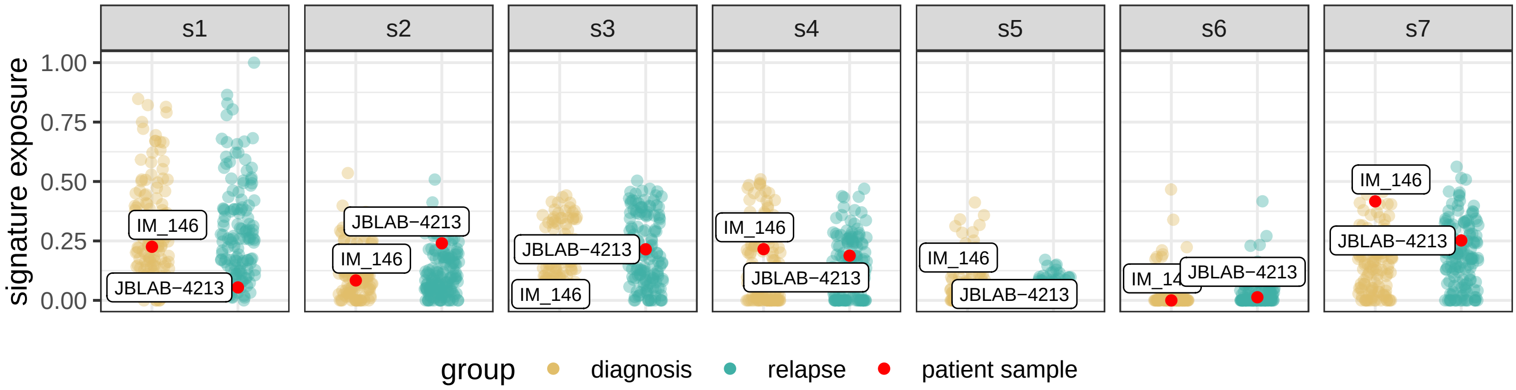

BRITROC−99

age: 63

stage: 4

platinum status: sensitive

prior lines: 1

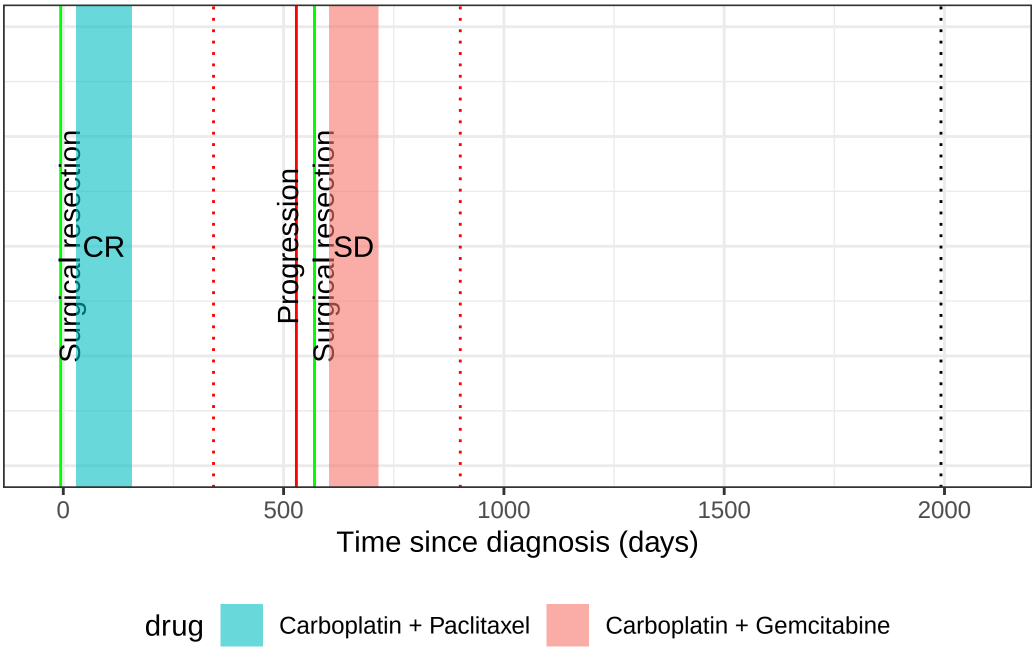

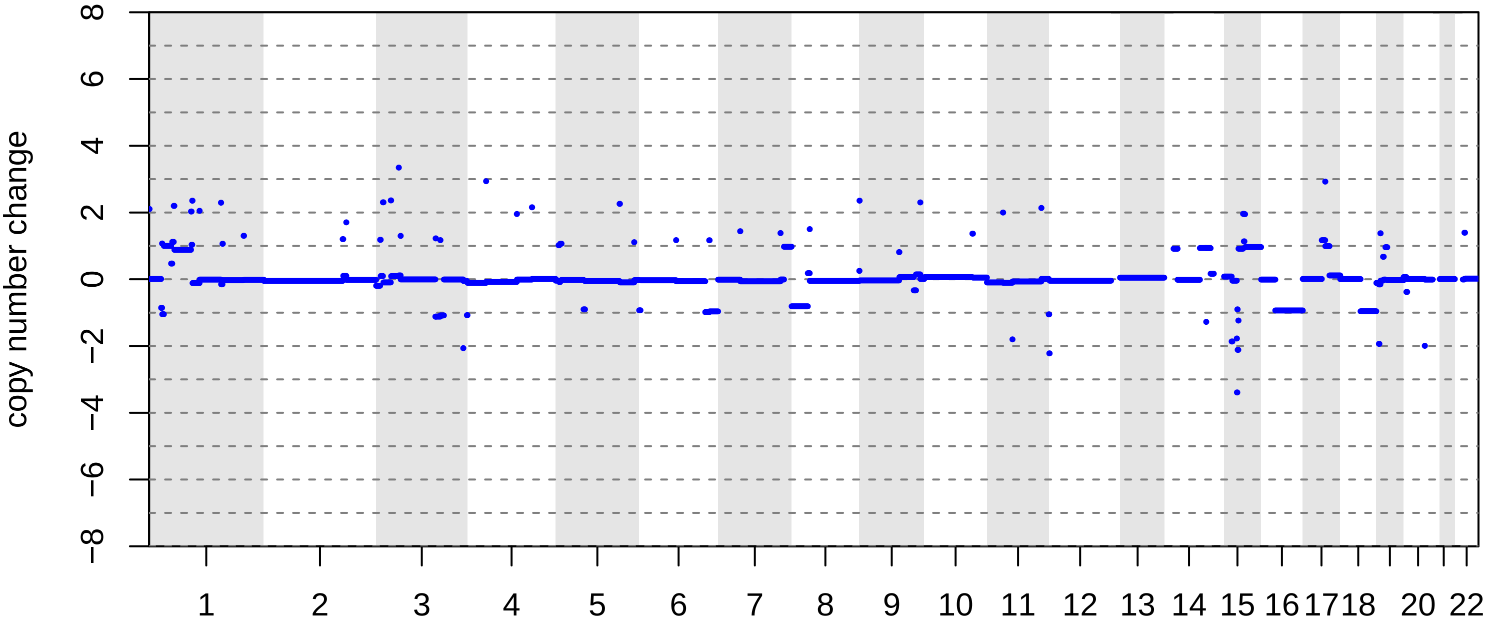

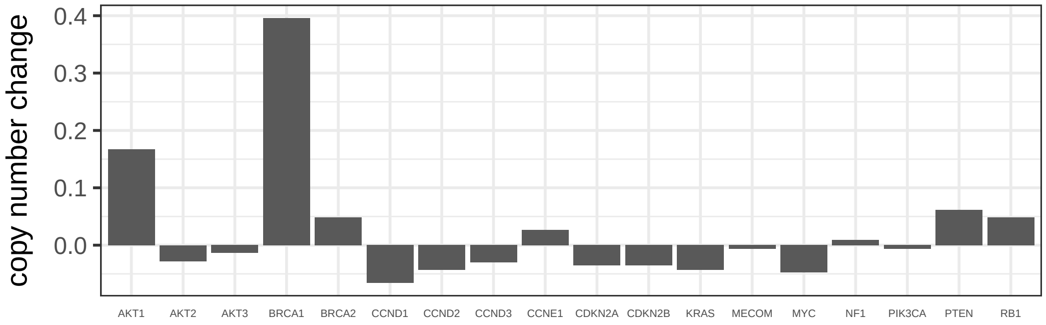

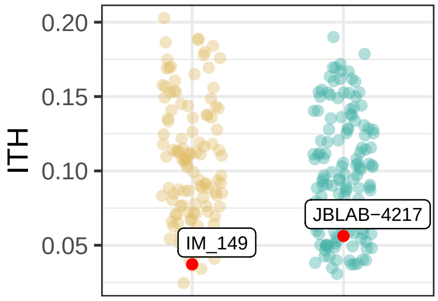

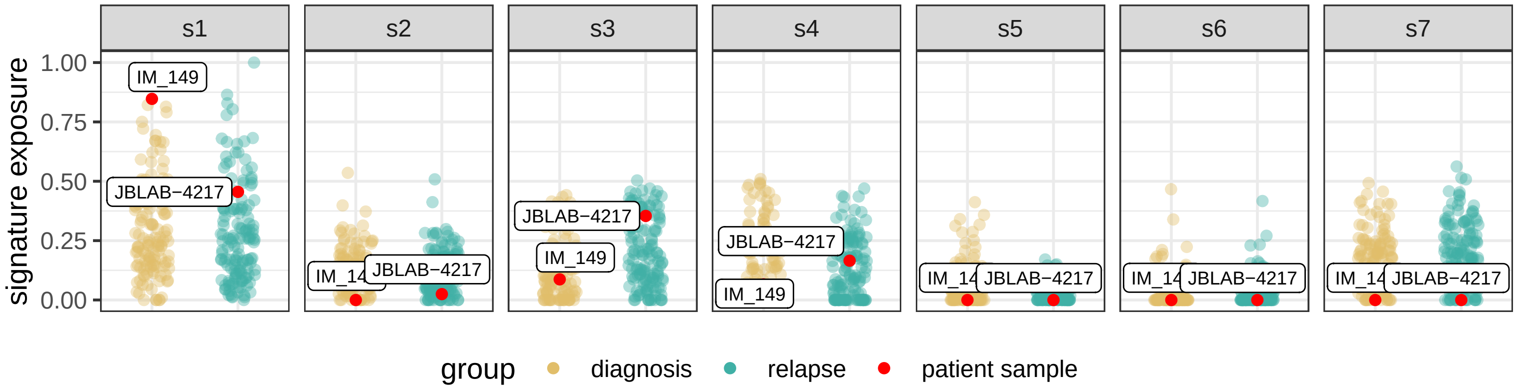

Supplement: Supplementary file 7 — Supplementary Dataset 4 [file 41467_2023_39867_MOESM7_ESM.pdf]
