# Supplementary material for: The copy number and mutational landscape of recurrent ovarian high-grade serous carcinoma
Source: Nat Commun. 2023 Jul 20;14:4387. doi: 10.1038/s41467-023-39867-7 (PMC10359414; doi:10.1038/s41467-023-39867-7)
Supplement: Supplementary file 9 — Reporting Summary [file 41467_2023_39867_MOESM9_ESM.pdf]

nature portfolio

Corresponding author(s): Professor Iain McNeish

Last updated by author(s): 2022/10/21

Reporting Summary

Nature Portfolio wishes to improve the reproducibility of the work that we publish. This form provides structure for consistency and transparency in reporting. For further information on Nature Portfolio policies, see our Editorial Policies and the Editorial Policy Checklist.

Statistics

For all statistical analyses, confirm that the following items are present in the figure legend, table legend, main text, or Methods section.

n/a	Confirmed	
☐	☒	The exact sample size (*n*) for each experimental group/condition, given as a discrete number and unit of measurement
☐	☒	A statement on whether measurements were taken from distinct samples or whether the same sample was measured repeatedly
☐	☒	The statistical test(s) used AND whether they are one- or two-sided<br>*Only common tests should be described solely by name; describe more complex techniques in the Methods section.*
☐	☒	A description of all covariates tested
☐	☒	A description of any assumptions or corrections, such as tests of normality and adjustment for multiple comparisons
☐	☒	A full description of the statistical parameters including central tendency (e.g. means) or other basic estimates (e.g. regression coefficient) AND variation (e.g. standard deviation) or associated estimates of uncertainty (e.g. confidence intervals)
☐	☒	For null hypothesis testing, the test statistic (e.g. *F*, *t*, *r*) with confidence intervals, effect sizes, degrees of freedom and *P* value noted<br>*Give P values as exact values whenever suitable.*
☒	☐	For Bayesian analysis, information on the choice of priors and Markov chain Monte Carlo settings
☒	☐	For hierarchical and complex designs, identification of the appropriate level for tests and full reporting of outcomes
☐	☒	Estimates of effect sizes (e.g. Cohen's *d*, Pearson's *r*), indicating how they were calculated

Our web collection on statistics for biologists contains articles on many of the points above.

Software and code

Policy information about availability of computer code

Data collection	Raw clinical data were collected and stored on a bespoke Oracle database generated in the Cancer Research UK Clinical Trials Unit in Glasgow
Data analysis	All code required to reproduce the analysis in this paper is freely available and linked at https://github.com/BRITROC/britroc-1-HGSOC-landscape which details the utilised analysis pipelines, copy number fitting pipelines, and data access links. This repository is citable using the DOI 10.5281/zenodo.7942206 via Zenodo release tracking.

For manuscripts utilizing custom algorithms or software that are central to the research but not yet described in published literature, software must be made available to editors and reviewers. We strongly encourage code deposition in a community repository (e.g. GitHub). See the Nature Portfolio guidelines for submitting code & software for further information.

Data

Policy information about availability of data

All manuscripts must include a data availability statement. This statement should provide the following information, where applicable:

- Accession codes, unique identifiers, or web links for publicly available datasets
- A description of any restrictions on data availability
- For clinical datasets or third party data, please ensure that the statement adheres to our policy

Anonymised clinical data will be made available upon publication via request to Trial Management Group via the CRUK Clinical Trials Unit Glasgow

EGA submission for BAM files

Human research participants

Policy information about studies involving human research participants and Sex and Gender in Research.

Reporting on sex and gender	This study utilised samples of relapsed ovarian high grade serous carcinoma. Thus all participants were female.
Population characteristics	Women with recurrent histologically-proven ovarian cancer, primary peritoneal carcinoma or fallopian tube cancer of high grade serous and high grade endometrioid subtypes. Patients who had a diagnosis of ovarian cancer with a known germline mutation in BRCA1 or BRCA2 were also eligible for inclusion regardless of histological subtype. All participants had to be aged at least 18 years of age, with no upper age limit. All participants must have received at least one line of platinum-containing chemotherapy and have disease deemed suitable for imaging-guided biopsy (ultrasound or CT) by an experienced radiologist or suitable for intra-operative biopsy during secondary debulking surgery as determined by an experienced gynaecological oncology surgeon.
Recruitment	Patients were recruited from 14 UK gynaecological cancer centres: patients were identified by treating oncologists according to the inclusion and exclusion criteria that are detailed in Supplementary Methods. As stated above, all patients had to have disease deemed suitable for imaging-guided biopsy or suitable for intra-operative biopsy during secondary debulking surgery. This excluded patients with very small volume disease but we do not believe that this will alter the outcome. Patients had to be well enough to undergo biopsy - this will exclude patients who needed to start chemotherapy rapidly and thus could potentially mean that the study population was of good prognosis and better performance status. However, the overall survival data suggest that the recruited population were representative of those who have participated in clinical trials of chemotherapy in recurrent ovarian cancer.
Ethics oversight	Ethics/IRB approval was given by Cambridge Central Research Ethics Committee (Reference 12/EE/0349). All patients provided written informed consent

Note that full information on the approval of the study protocol must also be provided in the manuscript.

Field-specific reporting

Please select the one below that is the best fit for your research. If you are not sure, read the appropriate sections before making your selection.

☒ Life sciences ☐ Behavioural & social sciences ☐ Ecological, evolutionary & environmental sciences

For a reference copy of the document with all sections, see nature.com/documents/nr-reporting-summary-flat.pdf

Life sciences study design

All studies must disclose on these points even when the disclosure is negative.

Sample size	BriTROC-1 was originally powered to identify differences in the rates of defective homologous recombination in patients with platinum sensitive relapse, with a sample size 300. A total sample size of 300 (100 with platinum-resistant disease, 200 with platinum sensitive disease) provided power (>90%) to detect a 15% increase in the HRD status (based on the biopsies) in the sensitive group compared to the resistant group (assuming a 10% HRD rate in the resistant group) at the 5% two-sided level of statistical significance. The study recruited 276 patients, which reduced the power to 80%
Data exclusions	Not all biopsy samples yielded sufficient DNA or DNA of sufficient quality for analysis but no data were excluded.
Replication	This is an observational cohort study assessing genomic landscape of ovarian high grade serous carcinoma at diagnosis vs relapse. This is the first study of its kind ever performed and we are not aware of any comparable previous studies. We hope that future studies will be able to replicate our data in separate cohorts.
Randomization	There was no randomisation - all treatment was given at the discretion of treating oncologists. There was no control for co-variates such as age in the genomic analyses as each patient acted as their own control as we compared diagnosis and relapse tumour samples.
Blinding	There was no group allocation in this study. However, the platinum status (sensitive vs resistant) for each patient is a clinical definition based on time since last platinum chemotherapy (>6 vs <6 months respectively). This information was provided by the treating oncologists and was a key data point at the time of patient registration. All primary assays (e.g. focal somatic copy number alterations; SNV detection) were performed by research staff who did not have access to any clinical information (including platinum status, age, number of prior lines of therapy) at the time of primary analysis. Final analysis was then performed with reference to the relevant clinical data (especially platinum status and number of prior lines of therapy).

Reporting for specific materials, systems and methods

We require information from authors about some types of materials, experimental systems and methods used in many studies. Here, indicate whether each material, system or method listed is relevant to your study. If you are not sure if a list item applies to your research, read the appropriate section before selecting a response.

Materials & experimental systems

n/a	Involved in the study
☐	☒ Antibodies
☒	☐ Eukaryotic cell lines
☒	☐ Palaeontology and archaeology
☒	☐ Animals and other organisms
☐	☒ Clinical data
☒	☐ Dual use research of concern

Methods

n/a	Involved in the study
☒	☐ ChIP-seq
☒	☐ Flow cytometry
☒	☐ MRI-based neuroimaging

Antibodies

Antibodies used	CD3, Roche 790-4341, prediluted;<br>CD8, Spring Bioscience M5394, 0.5ug/ml;<br>Pan-Keratin, Roche 760-2135, prediluted<br>Mouse IgG, Roche 760-2014, prediluted (IgG control);<br>Rabbit IgG, Roche 760-1029, prediluted (IgG control);
Validation	The antibodies were validated in the Cancer Research UK Cambridge Institute Pathology Core by staining human lymphoid tissue and confirming appropriate staining location with a pathologist. As the markers are CD8 and CD3. A non lymphoid tissue was used as a negative control. The antibodies were further validated by multiplex staining, where the overlap between CD3 and CD8 was confirmed.

Clinical data

Policy information about clinical studies

All manuscripts should comply with the ICMJE guidelines for publication of clinical research and a completed CONSORT checklist must be included with all submissions.

Clinical trial registration	ISRCTN09180474
Study protocol	Protocol will be uploaded to the ISRCTN website - it is not yet uploaded but will be
Data collection	276 patients were recruited from 14 UK gynaecological cancer centres between 16/JAN/2013 and 05/SEP/2017
Outcomes	The primary outcome was to obtain 300 fit-for purpose tumour biopsies from women with relapsed ovarian high grade serous carcinoma – 100 from women with platinum-resistant relapse (relapse within 6 months of previous platinum-based chemotherapy) and 200 from women with platinum-sensitive relapse (relapse 6 months or more after completion of previous platinum-based chemotherapy).<br>The secondary outcomes were:<br>1. Assessment of mutations in HRD genes, BRCA1, BRCA2, RAD51C, RAD51D, BRIP1, in relapsed HGSOC samples by targeted next generation sequencing.<br>2. Comparison of allelic ratio of BRCA1 and BRCA2 in relapsed HGSOC and archival tumour samples taken at the time of diagnosis by targeted next generation sequencing<br>3. Analysis of mutations in TP53 (positive control for high grade serous pathology), PTEN, BRAF, KRAS, PIK3CA in relapsed HGSOC and archival tumour samples targeted next generation sequencing.<br>4. Assessment of germline DNA mutations in BRCA1, BRCA2, RAD51C, RAD51D, BRIP1 in women with relapsed HGSOC using targeted next generation sequencing.<br>5. Assessment of methylation of BRCA1 and BRCA2 in relapsed HGSOC and archival tumour samples taken at the time of diagnosis. We have not yet assessed this outcome and no results relating to this outcome are presented in this study, which only reports genomic data.
